# Kokumi taste perception is functional in a model carnivore, the domestic cat (*Felis catus*)

**DOI:** 10.1038/s41598-021-89558-w

**Published:** 2021-05-18

**Authors:** A. Laffitte, M. Gibbs, C. Hernangomez de Alvaro, J. Addison, Z. N. Lonsdale, M. G. Giribaldi, A. Rossignoli, T. Vennegeerts, M. Winnig, B. Klebansky, J. Skiles, D. W. Logan, S. J. McGrane

**Affiliations:** 1 WALTHAM Petcare Science Institute, Freeby Lane, Waltham on the Wolds, Melton Mowbray, Leicestershire LE14 4RT UK; 2 IMAX Discovery GmbH, Otto-Hahn-Straße 15, 44227 Dortmund, Germany; 3 AXXAM S.p.A., OpenZone, Via Meucci 3, 20091 Bresso, Milan Italy; 4 BioPredict, Inc., 4 Adele Avenue, Demarest, NJ 07627 USA; 5 Present Address: Valis Pharma, Ins., 545 Bonair Way, La Jolla, CA 92037 USA

**Keywords:** Computational biology and bioinformatics, Molecular biology, Neuroscience, Physiology

## Abstract

Kokumi taste is a well-accepted and characterised taste modality and is described as a sensation of enhancement of sweet, salty, and umami tastes. The Calcium Sensing Receptor (CaSR) has been designated as the putative kokumi taste receptor for humans, and a number of kokumi-active ligands of CaSR have been discovered recently with activity confirmed both in vivo and in vitro. Domestic cats (*Felis catus*) are obligate carnivores and accordingly, their diet is abundant in proteins, peptides, and amino acids. We hypothesised that CaSR is a key taste receptor for carnivores, due to its role in the detection of different peptides and amino acids in other species. Using in silico, in vitro and in vivo approaches, here we compare human CaSR to that of a model carnivore, the domestic cat. We found broad similarities in ligand specificity, but differences in taste sensitivity between the two species. Indeed our in vivo data shows that cats are sensitive to CaCl_2_ as a kokumi compound, but don’t show this same activity with Glutathione, whereas for humans the reverse is true. Collectively, our data suggest that kokumi is an important taste modality for carnivores that drives the palatability of meat-derived compounds such as amino acids and peptides, and that there are differences in the perception of kokumi taste between carnivores and omnivores.

## Introduction

Taste perception is generally described through the five primary taste modalities, sweet, sour, salty, bitter and umami, each recognised by specific receptors and transduction pathways^1^. In addition to these five, other “secondary” putative taste qualities such as fat taste, metallic taste, astringency and kokumi are gaining interest in the field of sensory sciences^2–5^.

Kokumi is a well-accepted taste sensation in Asian cuisine and was first characterised as a separate taste modality by Ueda and colleagues^6,7^. It is described as a sensation of enhancement of sweet, salty and umami tastes^5,8^ when associated with specific compounds, or a mouthful, thick, delicious taste^9^. Indeed, the word *kokumi* comes from the Japanese terms for *rich* (koku) and *taste* (mi). Kokumi compounds, however, are mostly described as tasteless in isolation^6,7^. Recently, the human Calcium Sensing Receptor (CaSR) has been designated as the putative kokumi taste receptor for humans^8,10^. CaSR is a member of the same receptor class as the T1R receptors for umami and sweet taste, the class C of G-Protein Coupled Receptors (GPCRs)^11^. The receptors in this class all have a similar structure comprising of a cytoplasmic tail, a heptahelical transmembrane domain (TMD), followed by a large N-terminal domain (NTD), which is sometimes referred to as the Venus-Flytrap domain (VFT). When expressed on the cell surface, CaSR is known to mainly function as a covalently-linked homodimer^12,13^. The main functional role of CaSR is to maintain calcium homeostasis in the blood^14,15^, through the modulation of Parathyroid Hormone (PTH) secretion. With its physiological importance, CaSR has been found to be expressed in most tissues involved in calcium homeostasis e.g. the parathyroid glands, kidneys, thyroid and the brain^15^, as well as the gastrointestinal tract^16^ and taste papillae^10^. It is also known to be involved in many physiological processes including, but not limited to, gastric acid secretion^17^, or insulin release from beta-cells in the pancreas^18^, promoting glucose tolerance when activated by agonist peptides^16^, but also in pathophysiological processes such as vascular calcification^19^ and osteoporosis^15^. The CaSR has at least eight binding sites, and most of these are able to only bind Ca^2+^ and other metals. However, we know that there are at least two sites able to bind larger agonists, one within the NTD and one within the TMD^15,20^.

The first study to make the link between kokumi taste and human CaSR (hCaSR), screened a large library of γ-glutamyl peptides for agonist activity against hCaSR, and showed that these specific peptides were able to activate the receptor in vitro^8^. The same γ-glutamyl peptides also elicited kokumi sensation/ taste for trained sensory assessors when mixed with umami-tasting preparations^8^. Maruyama and colleagues found that mouse CaSR (mCaSR) was expressed in the mouse lingual epithelia, in type II and type III taste cells, and co-expressed with taste cell markers Phospholipase C β2 (PLCβ2) and Neural Cell Adhesion Molecule (NCAM). The same study confirmed that mCaSR was activated by specific kokumi compounds and induced the release of intracellular Ca^2+^, similar to other Class C GPCR taste receptors such as the umami receptor^10^. Taken all together, this evidence strongly suggests that CaSR is indeed a putative receptor for kokumi taste in mammals.

Domestic cats (*Felis catus*), and other members of the *Felidae* family, are obligate carnivores. Accordingly, their diet is abundant in proteins, peptides, L-amino acids and fats, but they do not eat food that contains high amounts of sugars^21^. The different dietary habits of carnivorous and herbivorous mammals has resulted in the evolution of different taste perception systems^22^. For example, cats and humans, who are strict carnivores and omnivores, respectively, are differently responsive to a number of L-amino acids and sweet-tasting compounds^23^.

We hypothesise that CaSR is a key taste receptor for carnivores, due to its role in the detection of different peptides and amino acids in other species. There are currently no published data, to our knowledge, on carnivore kokumi taste detection. Using in silico, in vitro*,* and in vivo approaches, we compare hCaSR to that of a model carnivore, the domestic cat CaSR (cCaSR). We demonstrate broad similarities in ligand specificity, but differences in taste sensitivity between the two species. Collectively, our data suggest that kokumi is an important taste modality for carnivores that enhances the palatability of meat-derived compounds such as peptides and amino acids.

## Results

### CaSR is expressed in the cat circumvallate taste papillae

**Figure 1:**
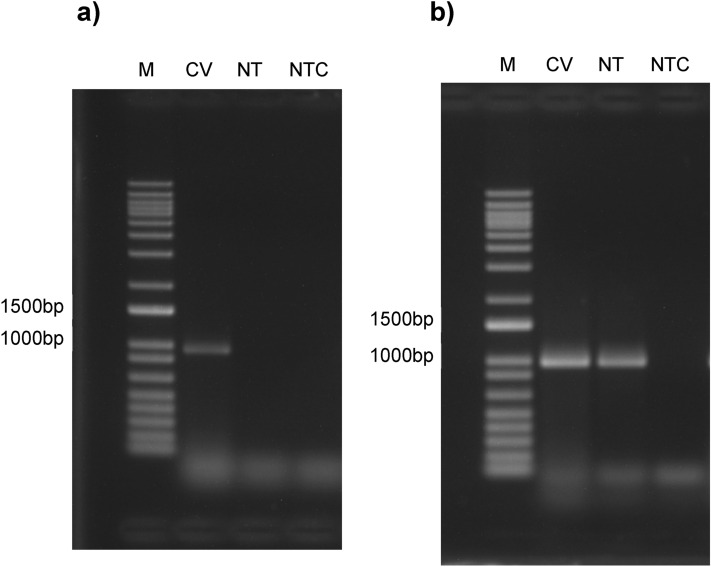
CaSR is expressed in cat circumvallate papillae (**a**). GAPDH is expressed in both circumvallate papillae and non-taste epithelial tissue (**b**). (**a**) CaSR RT-PCR for a cat circumvallate papilla (CV), non-taste epithelial tissue (NT) and no-template controls (NTC). (**b**) GAPDH RT-PCR was used as a positive control in the same tissues. Expression of CaSR was only observed in the CV tissue. M—Molecular size marker.

In order to confirm that cats express CaSR in their taste tissue, we used biopsies of cat circumvallate papillae (CV) to perform an RT-PCR analysis (Fig. 1). With this analysis we confirmed the expression of CaSR in the CV, with no expression being observed in epithelial tongue tissue without visible papillae. (See Supplementary Data, Fig. 1 for full-length gels).

### Cat and human CaSR protein sequences are homologous

**Figure 2:**
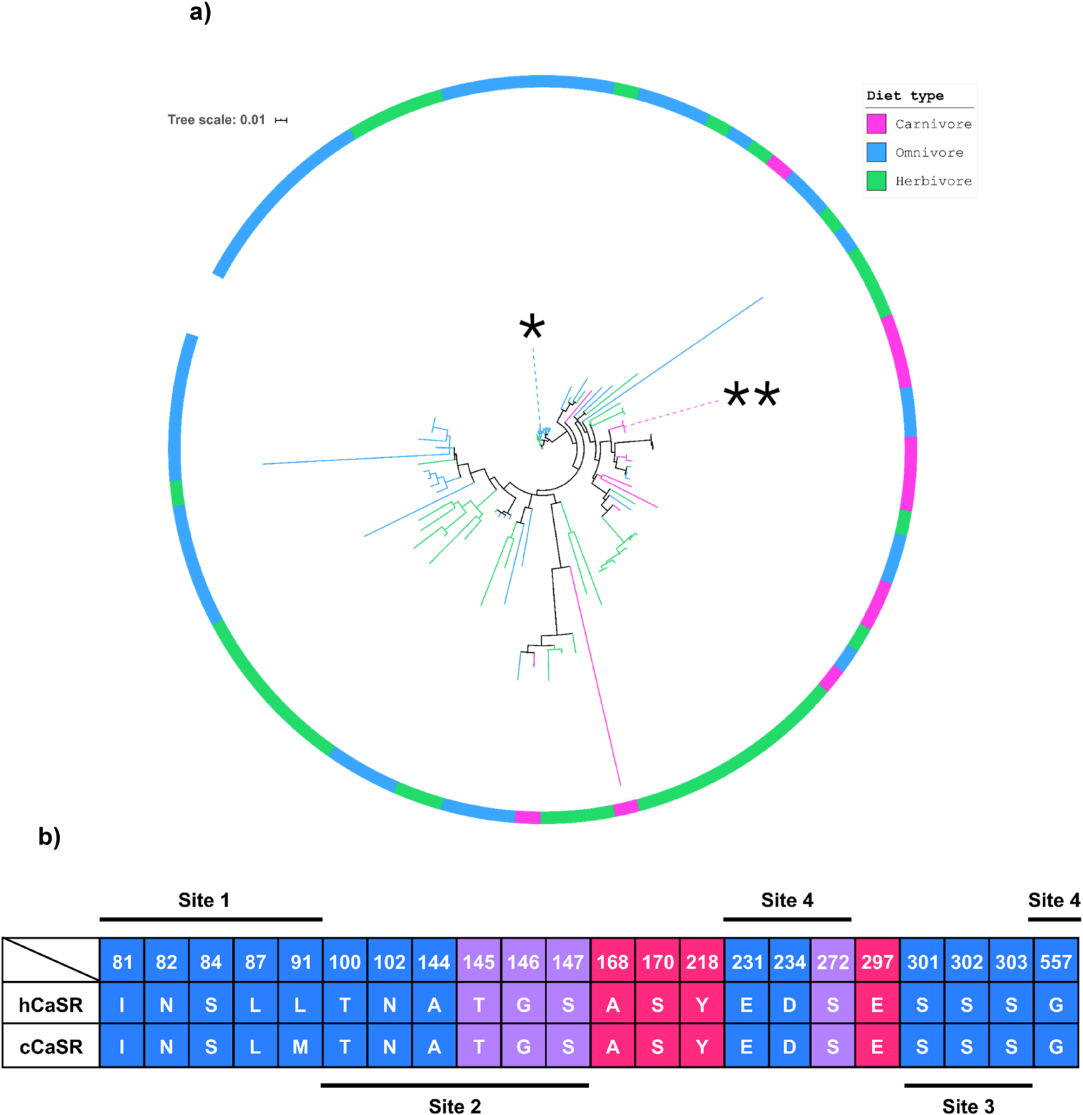
CaSR phylogenetic tree of mammalian species (**a**) and alignment of the residues from all four Ca^2+^ binding sites compared between cCaSR and hCaSR (**b**). (**a**) Labels are coloured by diet type (green = herbivore; blue = omnivore; pink = carnivore). *—Homo sapiens, **—Felis catus. (**b**) Sequence alignment comparing the residues that bind Ca^2+^ (blue), GSH (red) or both (purple) for hCaSR and cCaSR. The sites indicated, correspond to Ca^2+^ binding sites on the receptor ^20^.

**Figure 3:**
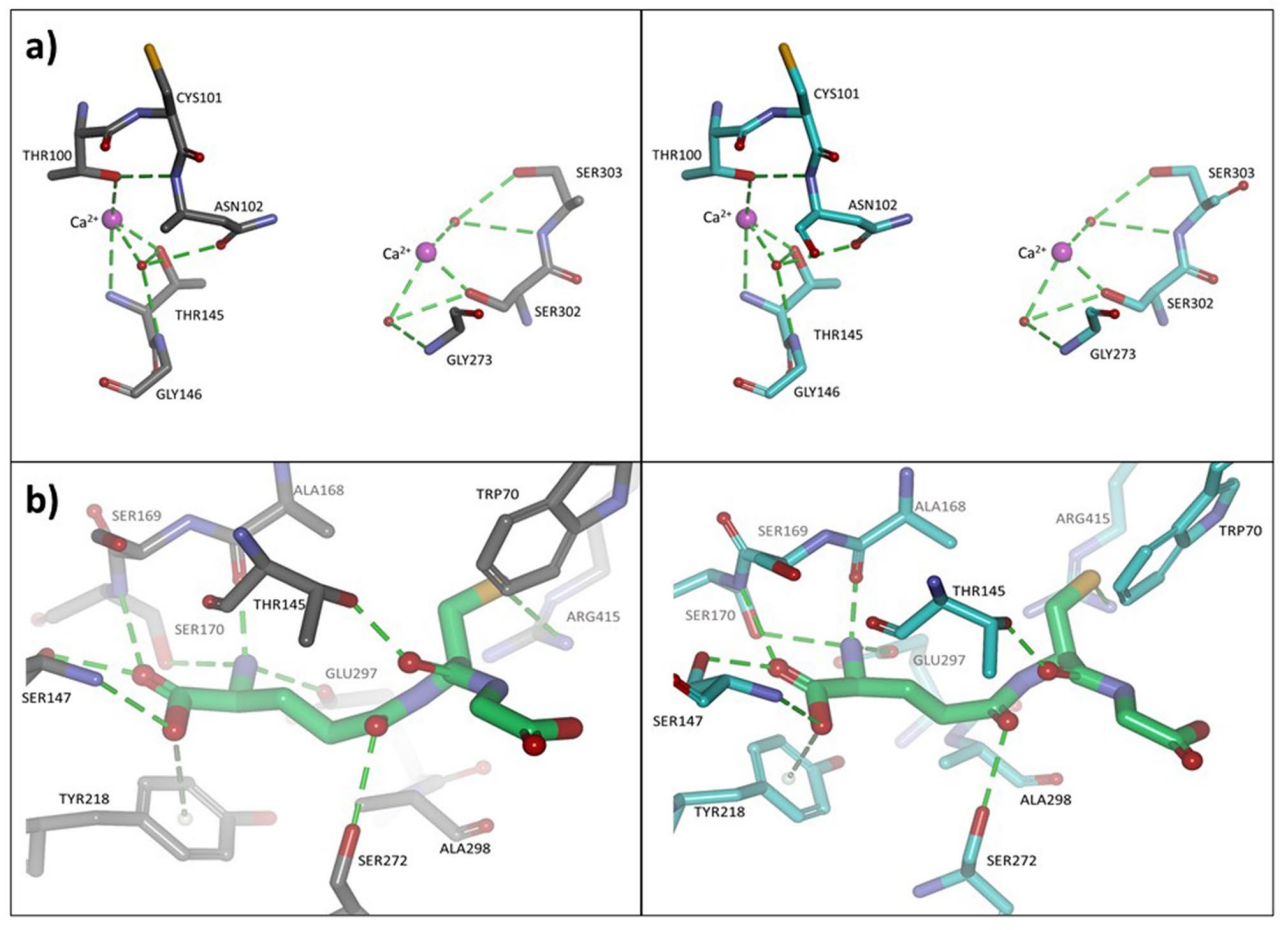
NTD with Ca^2+^ of the hCaSR crystal structure (left) and homology model of cCaSR (right) (**a**). hCaSR (left) and cCaSR (right) with GSH bound into their respective binding sites (**b**). (**a**) The Ca^2+^ binding site of hCaSR (left) and the Ca^2+^ binding site of cCaSR (right). The secondary structure of the hCaSR protein is in grey. The Ca^2+^ binding site of hCaSR (PDB structure 5fbk that was the template for cCaSR) is identical to cCaSR, including the enumeration of the amino-acids. The secondary structure of the cCaSR protein is in cyan. (**b**) The GSH (in green) binding site is also situated in the NTD of hCaSR (left) and cCaSR (right). The GSH binding site of hCaSR is identical to cCaSR, including the enumeration of the amino-acids. In both species, hydrogen bonds form with residues ALA168, THR145, SER147, SER170, SER272, GLU297; hydrophobic interaction with TYR218; charged interaction of zwitterionic nitrogen of the amino acid group to GLU297; charged interaction of the carboxyl group to Ca^2+^ (not shown for clarity of image). The images were generated using with the Discovery Studio Visualizer (BIOVIA, Dassault Systèmes).

In order to understand how similar cCaSR and hCaSR are from an evolutionary perspective, we generated a phylogenetic tree of all available mammalian CaSR sequences and found them to display high sequence identity (Fig. 2). On the phylogenetic tree, hCaSR clusters together with other primate species, and is the closest to the other *Hominidae* family members (Fig. 2a). cCaSR, on the other hand, clusters together with other felines (strict carnivores) as well as some omnivores such as the dog and the fox (See Supplementary Fig. 2 for phylogenetic tree with species details). In addition to the hCaSR and cCaSR sequences, we selected a number of model species from both omnivore and strict carnivores, to visualise possible sequence variation in the putative calcium binding site within the CaSRs NTD^24^. The main residues that interact with Ca^2+^ “Site 2” and “Site 3” (Fig. 2b), are situated in the hinge region of the CaSR NTD (Fig. 3)^20^. There are two other sites, “Site 1” (Fig. 2b) that is situated in lobe 1 of the NTD, and “Site 4” (Fig. 2b) which is included close to the cysteine rich domain^20^, but these sites only bind Ca^2+^, to the best of our knowledge, so they were of less interest to us in this work. Only one of the amino acids in the receptor sequence vary between cat and human, residue 91, which is a Leucine for humans and Methionine for cats (Fig. 2b). In our sequence alignment used to generate the phylogenetic tree (Supplementary Fig. 3), we only found minor sequence differences between mammalian species in general, confirming the highly-conserved nature of this receptor. Hence, we next investigated the ligand binding profile of cat CaSR to understand whether its function differs from the human receptor.

### From a predicted 159 ligands, 93 specifically activated cCaSR in a cellular model

**Figure 4:**
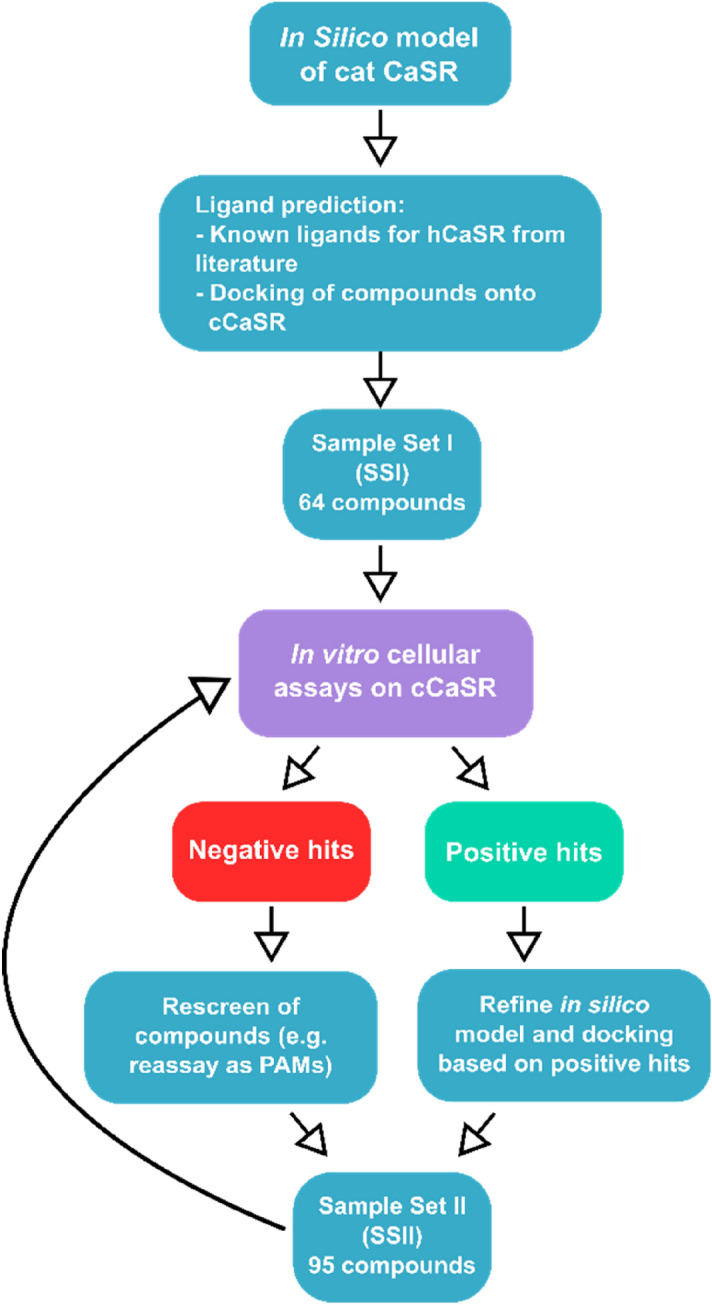
Schematic representation of the hypothesis driven, iterative approach used to predict ligand binding into cCaSR. In the process, we used information from literature on hCaSR, as well as our homology model used for docking. Between sample set I and sample set II, some compounds were repeated, and a final number of 159 unique compounds were screened on cCaSR.

In order to gain a holistic view of the ligand activity of cCaSR and to be able to compare the ligand specificity to that of hCaSR, we used a hypothesis-driven approach (Fig. 4), where we predicted possible agonists and Positive Allosteric Modulators (PAMs) of cCaSR using known ligands for hCaSR from literature, combined with docking into a homology model of cCaSR NTD built using hCaSR NTD crystal structures as templates (Fig. 3). We have only concentrated on compounds that bind within the NTD, as with our homology model all “food-related” compounds e.g. peptides, amino acids, and ions, bind within this domain. We have docked a number of compounds within the NTD, such as Ca^2+^ and glutathione (GSH) (Fig. 3), and these compounds bind within the pocket in the hinge region.

The binding site within the TMD accommodates some synthetic compounds, known allosteric modulators, that are of pharmaceutical interest, which was not the focus of this work^15^. Indeed, when we tested some of the abovementioned ligands of interest with our homology model, none of them docked successfully into the PAM site of transmembrane domain of CaSR. Overall, CaSR TMD ligands tend to be hydrophobic such as the synthetic drug Cinacalcet^15^, whereas the “food-related” ligands of interest to our work, such as GSH or the small cations, are polar and charged, and are better accommodated within the NTD binding site (Fig. 3). However, we did use a number of known PAMs from the literature, in order to evaluate the functionality of our cCaSR in vitro model in comparison to the hCaSR.

Overall, we screened 159 unique predicted agonists and/ or PAMs in the cCaSR cellular assay (Supplementary Table I). All compounds were screened in two different conditions, in the presence and absence of Ca^2+^, in order to define whether a compound is an agonist (tested in the absence of Ca^2+^) or a PAM (tested in the presence of Ca^2+^). Amongst the 159 compounds screened, we found 29 compounds to be PAMs of cCaSR. As there is no evidence that PAMs have taste activity to the best of our knowledge, we chose to focus on the potential agonists based on previously-published data for hCaSR^8,10,15,20,25,26^ and our homology model. From all the compounds screened, we found that 64 were agonists of cCaSR, giving an overall combined agonist (64) and PAM (29) hit-rate for our screen of approximately 58%. The agonist hit-rate was approximately 40% (Table 1). We could define an EC_50_ value for 25 compounds, or 39% of all the agonists identified.

**Table 1:** Activity measurement of 92 agonists on the cCaSR: All compounds listed here were screened using the HEK T-Rex/ natClytin cell line inducibly expressing cCaSR in an agonist assay. All measurements were repeated over at least three wells on the same assay plate. If the maximal response to a compound was not reached, the EC_50_ value could not be determined and is expressed as an estimation at > 0.01 M. For all agonists, the activity on cCaSR is described in a separate column: + : EC50 equal or above to 0.1 M, ++: EC50 below 0.1, +++: EC50 below 0.001. The compounds are grouped by different classes. 1–7: Cations, 8–39: Amino acids and derivatives, 40–42: Polybasic peptides, 43–47: GSH and derivatives, 48–50: Biogenic amines, 51–54: β-aspartyl peptides, 55–73: γ-glutamyl peptides, 74–80: Aminoglycoside antibiotics, 81–84: Sugars and derivatives, 85: 1,4,8,11-tetraazacyclotetradecane, 86–92: Organic acids.

No	Compound	cCaSR activity	EC_50_ (M)	No	Compound	cCaSR activity	EC_50_ (M)	No	Compound	cCaSR activity	EC_50_ (M)	No	Compound	cCaSR activity	EC_50_ (M)
1	**Ba**^**2+**^	++	**0.001172**	**24**	**γ-Carboxy-DL-glutamic acid**	+	** > *0.01***	**47**	**S-Methylglutathione**	++	**0.00442**	**70**	**γ-D-Glu-Trp**	+ +	**0.0042**
2	**Ca**^**2+**^	++	**0.00162**	**25**	**L-Glutamine**	−	***−***	**48**	**Cadaverine**	−	***–***	**71**	**γ-Glu-Cys-Gly-Oet**	−	**–**
3	**Gd**^**3+**^	+++	**0.000295**	**26**	**L-Isoglutamine**	+	** > *0.01***	**49**	**Spermidine**	++	**0.00249**	**72**	**γ-Glu-Val-Gly**	+ +	**0.00422**
4	**Mg**^**2+**^	+	** > *0.01***	**27**	**Glycine**	−	**−**	**50**	**Spermine**	+	** > *0.01***	**73**	**γ-Glu-ε-Lys**	−	**–**
5	**Pr**^**3+**^	** +++**	**0.000398**	**28**	**L-Isoleucine**	−	**−**	**51**	**β-Asp-Ala**	+	** > *0.01***	**74**	**Geneticin**	+	** > *0.01***
6	**Sr**^**2+**^	+	** > *0.01***	**29**	**L-Leucine**	−	**−**	**52**	**β-Asp-Gly**	+	** > *0.01***	**75**	**Gentamicin**	+ + +	**0.000989**
7	**Tb**^**3+**^	+++	**0.000175**	**30**	**L-Methionine**	−	**−**	**53**	**β-Asp-Leu**	+	**0.00315**	**76**	**Hygromycin B (Streptomyces hygroscopicus)**	−	**–**
8	**L-Alanine**	**−**	**–**	**31**	**L-Proline**	−	**−**	**54**	**β-Asp-Phe**	+	** > *0.01***	**77**	**Neomycin**	+ +	**0.00187**
9	**L-Asparagine**	**−**	**–**	**32**	**L-Serine**	−	**−**	**55**	**γ-Glu-Abu-Gly**	++	**0.00265**	**78**	**Paromomycin sulfate salt**	+ +	**0.00108**
10	**L-Aspartic acid**	++	**0.00411**	**33**	**L-Threonine**	−	**−**	**56**	**γ-Glu-Abu**	+	** > *0.01***	**79**	**Ribostamycin sulfate salt**	+	** > *0.01***
11	**D-Aspartic Acid**	**−**	**–**	**34**	**L-Tyrosine**	−	***−***	**57**	**γ-Glu-Ala**	+	** > *0.01***	**80**	**Sisomicin sulfate salt**	+ + +	**0.000299**
12	**L-Aspartic acid β-methyl ester hydrochloride**	+	** > *0.01***	**35**	**O-Phospho-L-tyrosine**	+	** > *0.01***	**58**	**γ-Glu-Cys**	+	** > *0.01***	**81**	**3-O-Methyl-D-glucopyranose**	−	**–**
13	**DL-Aspartic acid α-methyl ester**	**+**	** > *0.01***	**36**	**L-Valine**	−	**−**	**59**	**γ-Glu-Gln**	+	** > *0.01***	**82**	**D-( +)-Glucose**	−	**–**
14	**(S)-a-Methylaspartic acid**	**−**	**–**	**37**	**L-Histidine**	−	**−**	**60**	**γ-Glu-Glu**	++	**0.00207**	**83**	**Sucralose**	−	**–**
15	**L-Cysteine**	**−**	**–**	**38**	**Taurine**	−	**−**	**61**	**γ-Glu-Glu-Gln**	+	** > *0.01***	**84**	**Sucrose**	−	**–**
16	**Cystine (disufide)**	**−**	**–**	**39**	**L-Ornithine**	−	**−**	**62**	**γ-Glu-Glu-Glu**	++	**0.00235**	**85**	**1,4,8,11-tetraazacyclotetradecane**	+ + +	**0.000773**
17	**Se-(Methyl)selenocysteine**	++	**0.00447**	**40**	**Poly-L-arginine**	+++	**1.014 E-06**	**63**	**γ-Glu-Gly**	+	** > *0.01***	**86**	**1 s,3 s-1-aminocyclobutane-1,3-dicarboxylic acid**	+	** > *0.01***
18	**L-Cysteic acid monohydrate**	++	**0.00234**	**41**	**Poly-L-lysine**	+	** > *0.01***	**64**	**γ-Glu-Leu**	+	** > *0.01***	**87**	**2-Amino-4-phosphonobutyric acid**	+	** > *0.01***
19	**L-Homocysteic acid**	+	** > *0.01***	**42**	**Poly-L-ornithine**	+++	**0.00023**	**65**	**γ-Glu-Met**	+	** > *0.01***	**88**	**2-Aminopimelic acid**	+	** > *0.01***
20	**L-Glutamic acid**	**+**	** > *0.01***	**43**	**Glutathione**	++	**0.00639**	**66**	**γ-Glu-Phe**	++	**0.00232**	**89**	**L-( +)-2-Amino-3-phosphonopropionic acid**	+	** > *0.01***
21	**D-Glutamic acid**	**−**	**–**	**44**	**3-Glutathionyl-S-methylindole**	++	**0.00374**	**67**	**γ-Glu-Trp**	+	** > *0.01***	**90**	**L-2-Aminoadipic acid**	+	** > *0.01***
22	**4-Fluoro-DL-glutamic acid**	+	** > *0.01***	**45**	**S-(2-Hydroxyethyl)glutathione**	++	**0.00211**	**68**	**γ-Glu-Tyr**	++	**0.00388**	**91**	**Methylenediphosphonic acid**	+ +	**0.00129**
23	**2S,4S-g-Hydroxy-L-glutamic acid**	+	** > *0.01***	**46**	**S-Lactoylglutathione**	+	** > *0.01***	**69**	**γ-Glu-Val**	++	**0.00452**	**92**	**Methylphosphonic acid**	+	** > *0.01***

For each tested compound, a number has been assigned in Table 1, and they will be referenced by this number throughout this section. All tested di- and trivalent cations (1–7) were shown to be agonists of CaSR with the EC_50_ values ranging from 0.0003 to > 0.01 M.

From the L-amino acids, only L-Glu (20) and L-Asp (10) exhibited strong agonist activity for cCaSR. In addition, a number of L-amino acid derivatives showed agonist activity including derivatives of L-Asp (12, 13), L-Glu (22–24), L-Gln (26), L-Cys (17, 18) and L-Tyr (35), all with affinities lower than that of Ca^2+^. From the three tested polyamines, only spermidine (49) and spermine (50) were agonists of cCaSR.

GSH, considered to be an exemplar ligand of hCaSR, and four of its derivatives (43–47), were all active on cCaSR, with similar EC_50_ values around 2–6 mM, except for S-lactoglutathione (46), for which the EC_50_ could not be defined. We also screened four β-aspartyl peptides on cCaSR (51–54), all of which were active, and 19 γ-glutamyl peptides (55–73), of which 17 were agonists of cCaSR.

We screened seven aminoglycoside antibiotics (74–80) against cCaSR, and six were found to be agonists. This class of compounds had varying EC_50_ values to cCaSR, from 0.1–2 mM, slightly higher than values previously reported for antibiotics with hCaSR^30^.

There is some evidence that hCaSR is able to bind sweet-tasting compounds such as glucose, which has been shown to function as a PAM on the receptor^31^ (81–84), which is why we tested a number of sugars on cCaSR. However, none of the sugars we tested were predicted to function as agonists of the receptor according to our docking model and as predicted, none of the sugars or their derivatives were agonists of cCaSR in vitro.

Compounds 85–92 belong to various classes of synthetic/ pharmacologically-active compounds that were predicted to be active on cCaSR from the docking analysis. All of these were active on cCaSR, but we were only able to define an EC_50_ value for two, 1,4,8,11-tetraazacyclotetradecane (cyclam) and methylenediphosphonic acid (85, 92).

**Figure 5:**
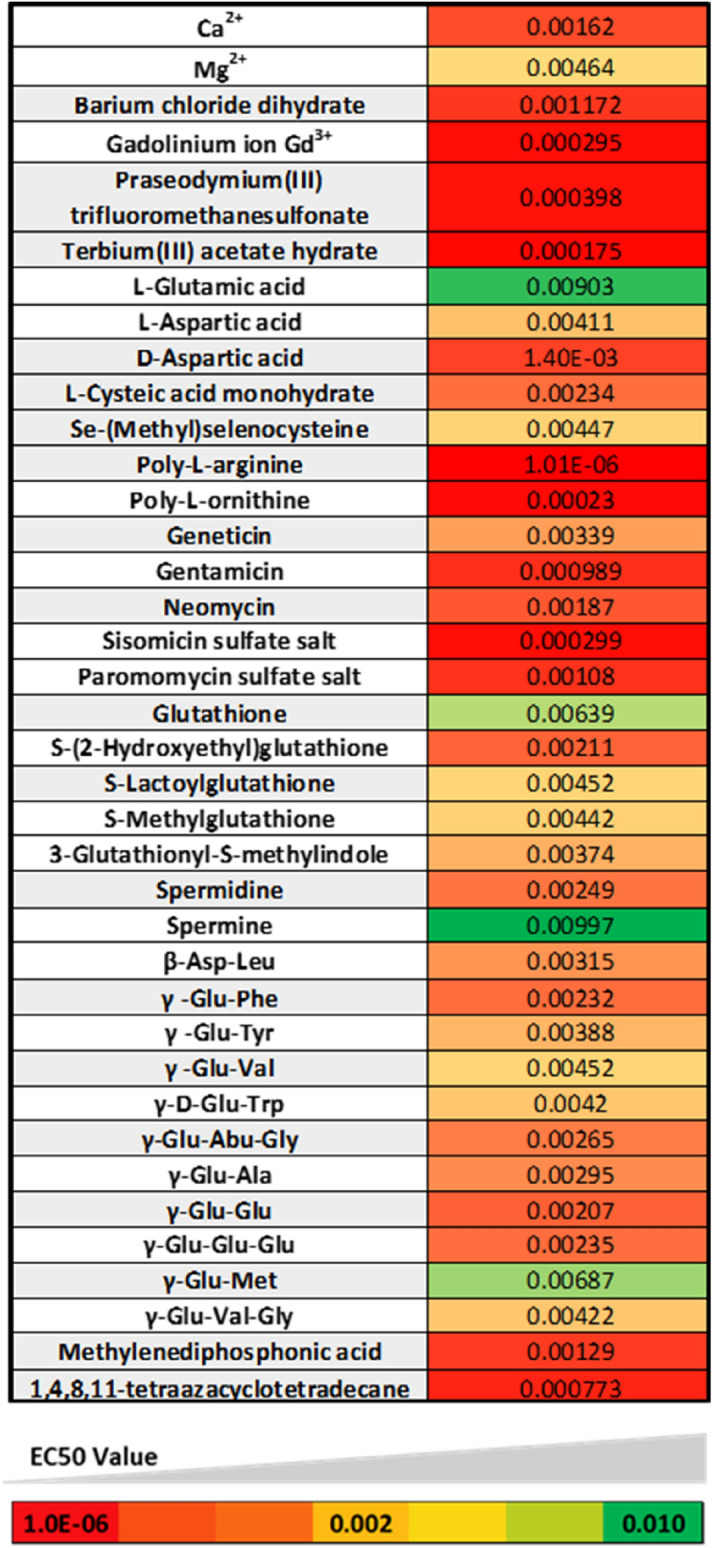
Heat map generated with CaSR agonists, classed according to their affinity for cCaSR. The Compounds with higher affinities (low µM range) appear in bright red, compounds with medium affinity in different hues of orange (mid to high µM range), and finally the compounds with lower affinities with the receptor are colored in green (mM range).

The strongest agonists were mostly cations, antibiotics, polybasic peptides, and cyclam (Fig. 5). The compounds with higher EC_50_ values for cCaSR are spermine, L-Glu, GSH and γ-Glu-Met (Fig. 5).

### A homology model of cCaSR and hCaSR reveals identical binding of cations

In order to further understand how the receptor structure differs between the two species, we used the aforementioned homology model generated for cCaSR and compared it to the crystal structure of hCaSR (Fig. 3) that has recently been elucidated^20,27^.

CaSR functions as a constitutive dimer, and the two CaSR subunits of one receptor form covalent homodimers^12,13^, tethered by a disulphide bond^15^. The crystal structures of hCaSR NTD^20,27^ show four Ca^2+^ binding sites, and we have included the residues that directly bind Ca^2+^ in Fig. 2b. One of the Ca^2+^ binding sites is situated in Lobe 1 of the NTD, one between Lobe 2 and the Cysteine-rich domain, and two between the lobes of the NTD within the hinge region. One of the Ca^2+^ ions within the hinge region is an integral part of the receptor structure, while the other one is presumed to enhance the closure of the two lobes upon ligand binding^27^. The cCaSR has a high (> 95%) sequence identity to hCaSR (Supplementary Data) with no discernible difference within the active sites in the NTD and the binding of Ca^2+^ ions and larger ligands such as GSH (Fig. 3a and b). In addition, we modelled hCaSR and cCaSR TMDs and found no difference between PAM active sites for these receptors and found that this binding site mainly accommodates large hydrophobic synthetic ligands that were of little interest to us in the remit of this study. In conclusion, we could find no structural explanation for the difference in ligand affinity between cCaSR and that reported for hCaSR.

### cCaSR and hCaSR have near identical ligand binding activity in the same cellular system

**Figure 6:**
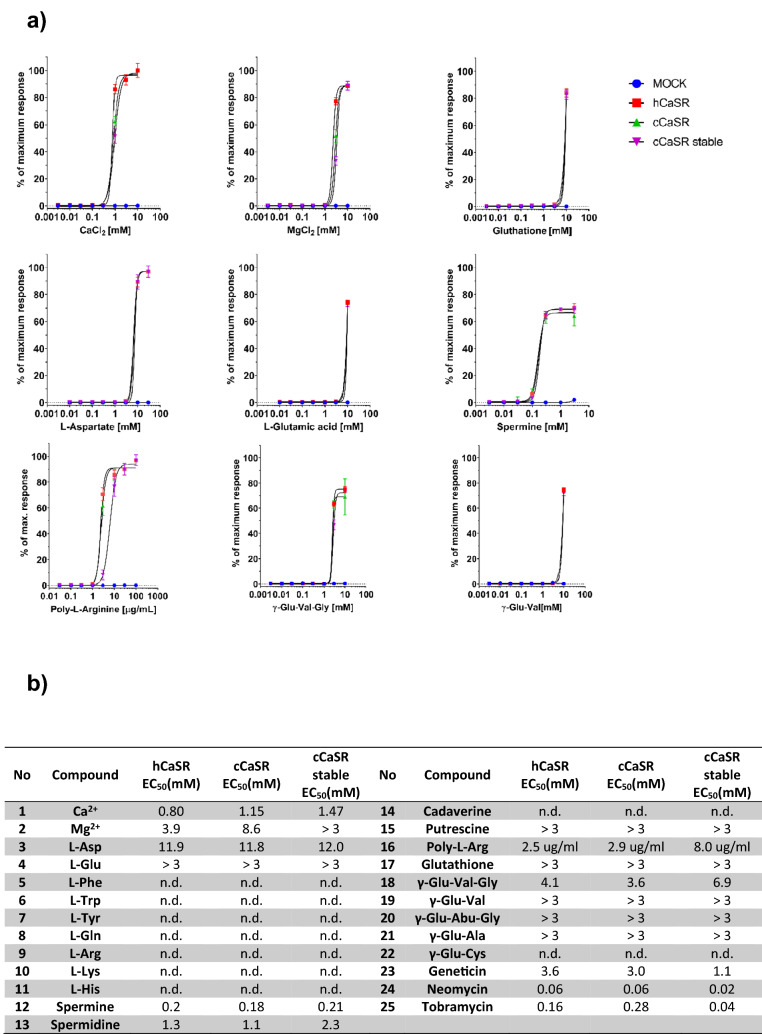
Both hCaSR and cCaSR bind agonists with similar affinities when expressed in the same cellular system (**a**). The EC_50_ values calculated for all the constructs are within similar ranges (**b**). (**a**) For each ligand the response of transiently expressed hCaSR (red square) and cCaSR (green triangle) and stably expressed cCaSR (purple triangle) were measured in response to increasing doses of the ligand. The maximal response used to calculate the % of maximal response was measured with CaCl_2_ at 30 mM. The measurements were made with luminescence, and each point was repeated in two independent measurements, and is represented with ± SEM. In each case, the measurements were made in parallel with cells containing the corresponding CaSR vectors, and cells containing a mock vector (blue circle) to confirm specificity of response. (**b**) All EC_50_ values are expressed in mM, except for poly-L-arginine which is expressed in µg/ml. All measurements were repeated over at least three wells on the same assay plate. If the maximal response to a compound was not reached, the EC_50_ value could not be determined and is expressed as an estimation at > 3 mM.

To better compare the ligand binding affinities of cCaSR to hCaSR, we next transiently-expressed both in the same cellular system and also compared their responses to the stable cCaSR cell line (Fig. 6a). The dose response curves are near identical between cCaSR expressed stably or transiently and very similar to hCaSR for most compounds tested (Fig. 6a), including the γ-glutamyl peptides, which had significantly lower EC_50_ values than reported previously^8^. The only case where we observed a slight difference in the dose response curves that translated to a difference in EC_50_ is poly-L-arginine, which had an approximately 2.5 fold lower EC_50_ value than hCaSR for cCaSR in both systems.

### CaCl_2,_ but not MgCl_2_ or GSH, show kokumi effect for cats in vivo

Having confirmed that hCaSR and cCaSR function similarly on a molecular level, we next compared in vivo responses in both cats and humans. Human kokumi responses have been relatively well-documented^6^, but the kokumi taste perception of strictly carnivorous mammals, such as cats, is unknown. We therefore designed a standard two-bottle choice test using a panel of cats trained to discriminate between tastants in liquid^23^. We tested three compounds, CaCl_2_, MgCl_2_ and GSH, first against water and then in a mixture of umami amino acids that are palatable to cats^34^, to test for an enhancement effect similar to kokumi sensations previously described for humans.

**Figure 7:**
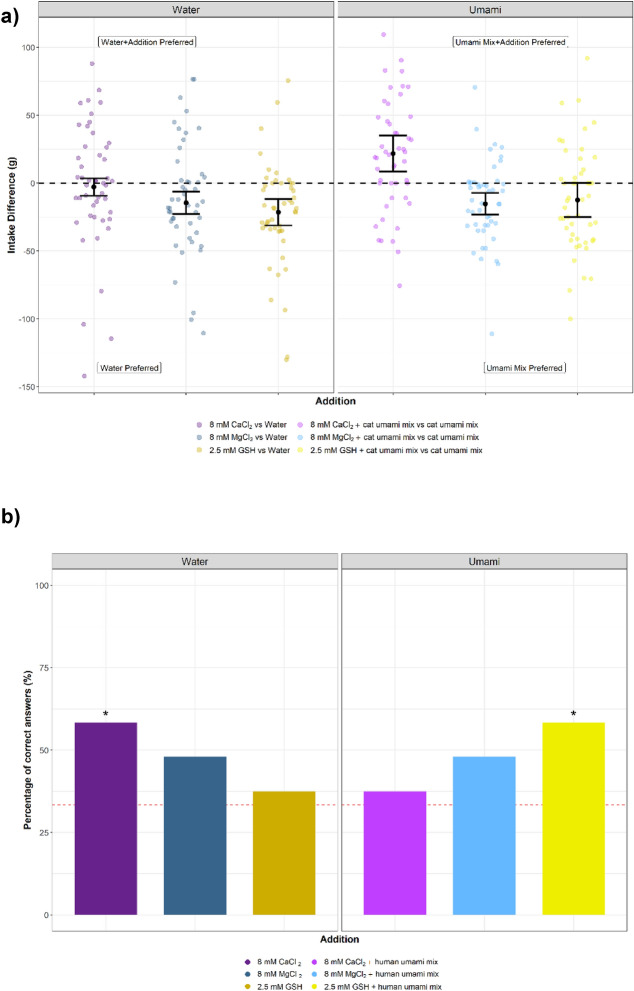
In vivo response of cats to three cCaSR agonists, CaCl_2_, MgCl_2_ and GSH, measured on a water panel in water and umami mix (15 mM L-Phe, 15 mM L-His and 15 mM L-Trp) (**a**). Human sensory panel evaluation of three compounds, CaCl_2_, MgCl_2_ and GSH, measured in Water and Umami Mix (1.18 mM MSG, 0.57 mM IMP and 119.7 mM NaCl) (**b**). (**a**) The cats did not significantly differentiate between water and CaCl_2_ (dark purple dots) at 8 mM, and there was no significant difference in intake (P = 0.3848). However, with the umami amino acid mix, the cats showed significant (P = 0.0039) preference to the mix that was supplemented with CaCl_2_ (light purple dots). The cats rejected the MgCl_2_ (dark blue dots) solution in comparison to pure water, and had a significantly lower intake of the MgCl_2_ solution (P = 0.0023). Similarly, the cats also significantly rejected the amino acid mixture containing MgCl_2_ (light blue dots) when compared to the amino acid mixture on its own (P = 0.0009). Finally, the cats significantly rejected the GSH solution (dark yellow dots) and had a lower intake for it when compared to pure water (P = 0.0002), and they had no significant preference to the amino acid mixture supplemented with GSH (light yellow dots) compared to the amino acid mixture on its own (P = 0.0630). n = 24 for all tests. For each compound, the test was repeated on two separate days, while switching the sides of the drinkers to account for side bias. (**b**) The assessors significantly differentiated CaCl_2_ in water (dark purple bar), but did not find any difference with the solutions proposed containing the synthetic umami mixture (light purple bar). The assessors did not differentiate the MgCl_2_ solutions significantly in either of the solutions proposed, water (dark blue bar) or the umami mix (light blue bar). The assessors did not significantly differentiate the GSH solubilised in water when compared with the blank (dark yellow bar), however they did differentiate it in the synthetic umami mix (light yellow bar). For each test, statistical significance was determined by a p-value ≤ 0.05 and n = 24 for all tests, except for MgCl_2_ in water and umami mix where n = 25.

There was no significant difference in the intake of a CaCl_2_ solution compared to water (P = 0.3848) (Fig. 7a), consistent with previous observations in humans where kokumi compounds are often tasteless on their own^6,7^. However, cats had significantly lower acceptance of both MgCl_2_ (P = 0.0023) and GSH (P = 0.0002) solutions, compared to water on its own (Fig. 7a), suggesting they are mildly aversive.

We then offered the same compounds to cats with a mixture of three palatable, umami-active amino acids dissolved in water (15 mM L-His, 15 mM L-Trp and 15 mM L-Phe)^34^, and compared the cats’ response to the amino acids alone. The cats had a significant preference (P = 0.0039) for the umami amino acid mix with CaCl_2_ added into the solution (Fig. 7a). However, the cats significantly preferred (P = 0.0009) the amino acid mix to the addition of MgCl_2_ (Fig. 7a). The cats displayed no statistically significant difference at the 5% level between the amino acid mix and the amino acid mix with GSH (Fig. 7a). We then used a combined approach to measure the difference between the intakes of each potential kokumi compound in water vs. in the umami amino acid mix. We measured a statistically significant difference for only one of the pairs, the two solutions containing CaCl_2_ (P = 0.0259).

### GSH, but not CaCl_2_ and MgCl_*2,*_ exhibit kokumi effect for a human sensory panel

In order to compare the cat in vivo response to humans, we assessed the same three compounds with a human sensory panel using assessors validated in line with ISO 8586:2012(E). Panellists were presented with each compound through a series of triangle tests. We prepared CaCl_2_, MgCl_2_ and GSH each in two different solutions; mineral water, and in an umami/ salt synthetic mix (1.18 mM MSG, 0.57 mM IMP and 119.7 mM NaCl) that was used to test kokumi taste with γ-glutamyl peptides previously^8^.

The panellists were able to detect CaCl_2_ in the water solution (% correct = 56%) (Fig. 7b). The most common descriptor used to describe CaCl_2_ in water was *bitter*, consistent with previous studies with CaCl_2_ on human taste panels^35^. The panellists were unable to detect CaCl_2_ in the synthetic umami and salty mixture (% correct = 36%), and indeed only 2/ 24 panellists used the word *bitter* to describe the solution they were testing.

The panellists were unable to significantly distinguish between the control and the samples containing MgCl_2_ in both solutions (% correct = 50% for both) (Fig. 7b), with 4/ 24 panellists describing the sample as *bitter* or *salty* when compared in blank water.

Finally, panellists were unable to taste GSH when mixed with water (% correct = 37.5%), but did significantly distinguish it in an umami mixture (% correct = 58.3%) (Fig. 7b).

## Discussion

There are some striking interspecies differences in taste perception, including in sweet, umami, and bitter taste^21,34,36,37^. One hypothesis to explain some of these differences, is that specialisation in the diets of species exerts differential selective pressure on their taste modalities^38^. Indeed, cats do not detect sweet taste due to the pseudogenisation of their T1R2 receptor^21^, while their umami receptor has a much wider specificity than, for example, the human receptor, and is capable of detecting a large number of amino acids^34,39^. As obligate carnivores, cats do not encounter many sweet compounds in their natural diet, however, detecting the presence of proteins and amino acids would be paramount^36^.

To our knowledge, no such comparisons have been conducted for kokumi taste between species from different dietary groups. Kokumi taste has been investigated in mice and humans^8,10,25^, both omnivores, and found to enhance the taste of peptides and amino acids. Therefore, we used phylogenetics, molecular modelling, in vitro activation, and in vivo sensory studies to compare kokumi taste perception in cat, as a strict carnivore, with humans. First, we confirmed that CaSR is expressed in cat taste tissue, specifically in the CV papillae on cat tongue (Fig. 1). Using sequence alignment and homology modelling, we found that cCaSR is very closely related to hCaSR from a structural perspective, and CaSR is highly-conserved between mammals overall (Fig. 2). Surprisingly, our in vitro data for cCaSR showed some differences in affinities and binding profiles when compared to human data^8,10,25,28^. However, further investigation with hCaSR in our cell system gave largely equivalent data to cCaSR, indicating that discrepancies with published data were due to differences in assay systems. We then proceeded to compare cat and human in vivo responses to CaSR agonists and kokumi compounds. Although the results from the in vitro assays showed identical agonist activity between hCaSR and cCaSR, we found that there were some differences in the in vivo responses between the two species, suggesting that there are more parameters to take into account with kokumi, than just the agonists used.

The phylogenetic tree (Fig. 2a) we generated for CaSR in mammals, as well as the multiple sequence alignment (Supplementary Data), indicated there is a high similarity between all the compared species, and we did not see any evidence of pseudogenisation of the receptor, on the contrary to the sweet taste receptor^21^. Since there did not seem to be any significant differences in the cat sequence when compared with the other mammalian species, we concluded that cats are an appropriate model species for carnivores.

In the residues that directly bind calcium^20^, aligned for human and cat CaSR, only one residue differs between the two species: residue 91 is a Leucine for human and Methionine for cat (Fig. 2b). When we looked at a more broad analysis of the sequence alignment we generated for the phylogenetic tree, we found that this residue is either a Methionine or a Leucine for all of the species. Leucine is most common for primates, and a few small mammals (Supplementary Fig. 1), whereas Methionine is not specific to any class of animals. Additionally, Methionine and Leucine are both hydrophobic residues of similar size^40^, and this difference does not affect the EC_50_ values measured for calcium (Fig. 6). Overall, our homology model of cCaSR confirms that the high sequence identity between cCaSR and hCaSR also translates into near identical 3D structure of the NTD of the two species (Fig. 3).

There is no aspect of the structural analysis that would suggest there is an important difference in kokumi perception between omnivores and carnivores. Calcium homeostasis is a vital function for all species, a pressure that may maintain a highly-conserved structure. Indeed, sensory receptors that have the most constraint, from an evolutionary point of view, are typically expressed in additional tissues and cell types, and may regulate non-redundant, critical physiological processes. This has previously been shown with specific olfactory receptors^41^.

From the alignment and structural analysis completed, we expected that the ligand binding properties of cCaSR would be very close to that of hCaSR and we screened a large library of compounds from different classes (Table 1). Most of the best binding compounds for cCaSR are not readily found in foods, such as antibiotics, cyclam or rare cations. They are more relevant for the understanding of CaSR function outside the taste system^14^, and their reported EC_50_ values with hCaSR are relatively close to the EC_50_ values we have found for cCaSR^15,27,30^. As these compounds would most likely be implicated in molecular functions related to calcium homeostasis and the other physiological functions of CaSR besides kokumi taste, we would expect their binding to be more conserved than the compounds that are only taste-active and don’t exert any other function on the receptor.

Of the potential kokumi-tasting compounds, some of the γ-glutamyl peptides, calcium, and the biogenic amine spermidine exhibited the lowest EC_50_ values for cCaSR. The formation of γ-glutamyl peptides is linked to GSH metabolism, and both γ-glutamyl peptides and GSH are found in different meats^32,33^, meaning that these compounds are present in a carnivorous diet. Similarly, spermidine, which is a biogenic amine, is found in food sources palatable to cats, including chicken liver^42^. Even the less-active compounds, for example GSH, had an affinity for cCaSR close to the concentration that it is found in specific meats^32^. Indeed, in combination with umami-active compounds such as L-amino acids, these compounds could elicit a strong positive taste response, consistent with cats’ strictly carnivorous diet.

When comparing the binding of the selected ligands measured for cCaSR with those previously published for hCaSR^8,10,25^, we found significant differences. However, when we expressed hCaSR and cCaSR in the same cellular system, the ligand binding properties of the receptors were near identical (Fig. 6). Contradictory reports on the ligand binding profile of CaSR have been published previously^31^^,^^43,44^. Thus, it is possible that CaSR is particularly sensitive to the heterologous expression system or methodology used to measure ligand binding. Comparisons between different studies should therefore be interpreted with caution.

Having concluded that the in vitro ligand binding between the two species is near identical, we designed two simple experiments to assess whether CaSR agonists also elicited the same kokumi taste response in cats and humans. CaCl_2_ elicited a clear kokumi-type response in cats, with no taste response to the compound in water alone, but a significant preference for it in an umami mix (Fig. 7a)^34^. For the human panel, we asked them to differentiate between a triad of solutions in a triangle test, in which they were presented with solution blanks and the same CaSR agonists as the cats (Fig. 7b). In contrast, CaCl_2_ is not a kokumi compound in humans, and although the human panel was able to significantly distinguish between plain water and the water solution of CaCl_2_, as expected they did not differentiate when CaCl_2_ was added into the synthetic umami mix (Fig. 7b). The umami mixes used for cats and humans were different, simply due to the fact that the human and cat umami receptors are activated by different ligands^34^. The cat umami mix contained 15 mM L-Phe, 15 mM L-His and 15 mM L-Trp, whereas the human synthetic umami mix was composed of 1.18 mM MSG, 0.57 mM IMP and 119.7 mM NaCl, as described previously with γ-glutamyl peptides^8^. Interestingly, the three cat umami-active amino acids are reported to activate hCaSR-expressing cells, in the presence of Ca^2+^ ^28^. We have previously shown that these amino acids elicit a typical umami response in vivo with the cats^34^. If these amino acids are also able to further activate the cCaSR in the presence of Ca^2+^, then the cats’ preference for the umami mix solution plus CaCl_2_ may also be partially due to this PAM effect.

However, the opposite of CaCl_2_ is true of GSH, which demonstrated a robust kokumi effect in our human panel, but was mildly aversive to cats both in water and in an umami-mixture (Fig. 7). Given the near identical ligand-activation profiles of CaSR in vitro, what underlies this apparent difference in kokumi taste between cats and humans? We consider there to be three potential explanations. It is possible that CaSR does not in fact mediate kokumi taste in cats despite being expressed in taste papillae. However, we consider it unlikely that the kokumi taste perception would arise twice in mammalian evolution when all other taste modalities appear to share a common molecular origin. It is also possible that methodological differences in the human and cat in vivo assays underlie the differences in kokumi taste reported here. Unlike in humans, training cats to signal taste discrimination in a forced choice test is challenging and risks a strong inherent preference bias. We instead exploited the fact that kokumi enhances umami taste, to measure preference in cats. While imperfect for inter-species comparison, we chose methodologies that are the most sensitive for each species. Instead, we propose that the most likely explanation for observed differences in kokumi taste between species is precisely because it functions as a synergistic taste modality: CaSR ligands frequently activate other taste receptors. MgCl_2_, for example, is an agonist of human bitter receptor T2R7^45^. This receptor is also expressed and functional in cats^36^. Our human panel was unable to detect MgCl_2_ at the concentration it was used, in accordance with previously published detection thresholds^35^. However, cats had a significant aversion both in plain water and in the umami amino acid mix (Fig. 7a), consistent with a bitter taste response^46^.

Similarly, the GSH solution tested was acidic (pH 3.48 for GSH at 2.5 mM). Humans sometimes describe its taste as *slightly sour* when concentrated enough, but overall it is tasteless in a water solution, confirmed by our sensory panel, which was unable to detect the GSH sample from the water blanks. Cats are particularly averse to sour-tasting compounds^47^ and this may explain their rejection of GSH in water, which is sufficiently strong such that any kokumi effect is mitigated. It is possible that in meat, where GSH is present at concentrations that can activate the cCaSR^32^, it could still be perceived as a kokumi compound since it would be present in a buffered pH environment with a mix of umami-active compounds. Finally, the cat umami receptor is much more broadly-tuned than the human umami taste receptor^34^, therefore the kokumi enhancement of umami taste may appear different in the presence of the same amino acid mix between the two species.

We conclude from these experiments that, despite the high conservation of the CaSR receptors, it is evident that there are some additional differences between how omnivorous and carnivorous mammals detect kokumi taste. This would suggest kokumi taste is among the more complex taste modalities. In this work we showed that a model carnivorous species and omnivorous species have very similar CaSR from a sequence and molecular structure perspective, as well as from the receptor binding point of view, but there are some important differences in the perception of kokumi taste between the species studied here. We hypothesise that this difference comes from the inherent difference in other taste modalities between the species, as well as the differences in their diets, and that the CaSR is very highly-conserved because of its physiological importance in other functions besides kokumi taste perception.

## Materials and methods

### Tissue preparation and RT-PCR

In order to confirm the expression of CaSR in the taste papillae of the cat, RT-PCR was conducted for CaSR with Glyceraldehyde 3-phosphate dehydrogenase (GAPDH), a ubiquitous enzyme found in most tissues, used as a control for non-taste tissue. The tissue was provided through a collaboration with the University of Veterinary Medicine in Hannover, Germany. Tissue samples from a six year old male cat were taken after euthanasia due to an inability of the cat to urinate. The cat was a client-owned cat, and permission to retrieve the samples was obtained prior to this work. An area of tongue epithelial tissue containing one circumvallate (CV) papilla was used for total RNA extraction along with a section containing no visible taste papillae. RNA was extracted using the RNeasy Plus Mini Kit (Qiagen, Germany) according to the manufacturer’s instructions. cDNA synthesis was performed with the Superscript III First Strand cDNA Synthesis Kit (Thermo Fisher Scientific, UK) with random primer based priming. The intron spanning primers used were as follows, CaSR, 5’-GCTGCTTTGAGTGTGTGGAA-3’ (forward) and 5’-ACCTCCTCGATGGTGTTACG-3’ (reverse); GAPDH 5’-GTGAAGGTCGGAGTCAACGG-3’ (forward) and 5’-ACCATAAGGTCCACCACCCG-3’ (reverse). For PCR, 25µL reactions were prepared with JumpStartTaq ReadyMix (Sigma-Aldrich, UK) according to the manufacturer’s instructions. PCR was performed with an initial denaturation step of 94 °C for 2 min, followed by 35 cycles of denaturation at 94 °C for 30 s, annealing at 57 °C for 30 s and extension at 74 °C for 2 min. A final elongation step at 72 °C for 5 min was performed before the reactions were held at 4 °C until they were stored at − 20 °C. Reaction products were run on a 1% agarose gel with post-staining in GelRed (Biotium, USA).

### Phylogenetic trees

The CaSR amino acid sequence (ENSFCAG00000008717) was taken from the domestic cat (*Felis catus*) Ensembl reference genome (FelCat9.0), and all orthologs were selected (Ensembl release 98, accessed in September 2019). The CaSR orthologs were filtered to remove sequences with < 60% identity compared with the cCaSR sequence, and to only keep sequences that were 1-to-1 matches from mammalian species. The associated taxonomic strings were taken from the NCBI Taxonomy Database^48^ using ETE 3^49^. Next, newick trees were created from the filtered ortholog sequences using FastTree 2.1.11 with the -wag option for the WAG + CAT model and -gamma option for rescaling of branch lengths and computing a Gamma20-based likelihood^50^. The newick trees were then visualised using iTOL (version 5.5)^51^.

### Functional expression of cCaSR and hCaSR

#### CaSR agonist preparation

All tested compounds were purchased as the purest form available from various chemical suppliers. All compounds were either directly dissolved in Calcium-free Tyrode’s buffer (130 mM NaCl, 5 mM KCl, 1 mM MgCl_2_, 5 mM NaHCO_3_, 20 mM HEPES; pH 7.4 sterile filtered and autoclaved), or in a mixture of Calcium-free Tyrode’s and DMSO, not exceeding a final DMSO concentration of 0.5% (v/ v) to avoid any toxic effects on transfected cells.

#### Cloning of cCaSR in expression vector

The coding sequence of the *Felis catus*^52^ and *Homo sapiens*^53^ *CaSR* gene was synthesised by GeneArt (Thermo Fisher Scientific). The cat coding sequence was excised from the GeneArt construct and inserted in pcDNA3.1 for constitutive and transient expression or pcDNA5/ TO for inducible expression. For the human sequence the pcDNA6 vector as delivered by GeneArt was used without further sub-cloning. After restriction analysis for the screening of positive constructs, a clone with correct DNA fragmentation was selected and confirmed by sequencing the whole coding region (data not shown).

#### Stable transfection of cCaSR in HEK cells

The cCaSR expression constructs were stably-transfected in the mammalian cell line, HEK293/ T-REx/ natClytin. Transfections were performed by electroporation, following standard methodologies. Stably-transfected target and mock pools were obtained after 2–3 weeks of antibiotic selection, and clones were selected for testing. In order to evaluate the activity of each clone, 10 mM Calcium was injected onto the cells and the resulting luminescence was measured. The HEK293/ T-REx/ natClytin-cCaSR clone pool analysis showed dozens of high-responding clones in cCaSR-expressing cells. Mock cells were analysed identically to the cCaSR cells, and no responses were obtained.

#### Transient transfection of HEK cells with hCaSR and cCaSR

The hCaSR and cCaSR expression constructs for transient expression were transfected in the mammalian cell line, HEK293/ natClytin. All transient transfections were performed with Lipofectamine 2000 (Invitrogen) according to the manufacturer’s protocol.

#### Luminescence assays on transient and stable constructs cCaSR and hCaSR

All compounds screened on the receptor were analysed in parallel with Calcium, on the HEK293/ T-REx/ natClytin-cCaSR or hCaSR stable or transient cell lines. Prior to the measurements, the cells were incubated with 10 µM of photoprotein substrate, Coelenterazine, prepared in Calcium-free Tyrode’s buffer at 37 °C for 3–4 h. All the data was captured using a FLIPR Tetra (Molecular Devices). A single or double injection protocol was used to capture the data for potential agonists and PAMs, respectively. The compounds tested were all prepared at desired concentration in Calcium-Free Tyrode’s buffer supplemented with 0.5% DMSO. For the activity determination of potential agonists, the single injection protocol was used, with CaCl_2_ as the positive control for each run. Each compound was injected into specific wells and the luminescence signal was measured for 1 min. for each compound. For potential PAMs, the double injection protocol was used. The first injection was identical to the one described above for potential agonists. A 20 min. resting period followed the first injection and a second injection of 0.5 mM CaCl_2_ in Calcium-free Tyrode’s buffer was injected to all wells, and the resulting luminescence signal was again measured for 1 min for each compound.

#### Data analysis

Dose–response curves were established by plotting signal amplitudes versus agonist concentration. For each compound, the % of maximum response was determined using a standard formula as follows:

The half maximal effective concentrations (EC_50_) were identified by nonlinear regression using a variable slope model with the equation Y = Bottom + (X^Hillslope)*(Top–Bottom)/ (X^HillSlope + EC50^HillSlope), where Y is the agonist concentration, Top and Bottom are the plateaus in the same units as Y and HillSlope is the Slope factor or Hill slope. All calculations and plots were done using GraphPad Prism 8. Several tested compounds elicited a cellular response only at 1 or 2 concentrations and not reaching a plateau, preventing us from using the above equation to calculate an EC_50_ value for these ligands. In these cases the EC_50_ was noted as being higher than the maximum concentration used in the assay.

### In silico modelling of the cat CaSR

The Protein Data Base (PDB) contains several structures of the human CaSR NTD, co-crystalised with Ca^2+^, Mg^2+^, and small molecular agonists, such as L-Trp derivative L-1,2,3,4-tetrahydronorharman-3-carboxylic acid (5FBK, 5FBH, 5K5T, and 5K5S)^20,27^.

These structures were used as the templates for our homology model of cCaSR constructed using the Modeller software, which includes both the sequence alignments and the building of the homology model (Discovery Studio- BIOVIA, Dassault Systèmes)^54^. Indeed, cCaSR has a high (> 95%) sequence identity to hCaSR with no discernible difference within the active sites of the lobes of NTD and in the vicinity of the binding sites of Ca^2+^ and Mg^2+^ ions. Ligands were docked into the model by using the program BioDock^55^. The resulting complexes were subjected to energy minimisation using the Gromacs software package^56^. Finally, the docking results were ranked manually, by energetic criteria that include hydrogen bonding, charged and hydrophobic interactions using CHARMM force fields^57^. The binding of the ligands exploited multiple electrostatic and polar interactions of the CaSR NTD binding site (Fig. 3). The placement of the amine groups of the amino acids within the hinge region of the cCaSR NTD followed the positioning of the amine groups to other structures of Group C GPCR’s and is similar to the binding of the tryptophan derivative to the hCaSR in the 5FBK structure (Fig. 3). The best-ranking compounds, conjectured to be active, were experimentally tested using the in vitro cellular assay.

The experimental results guided the refinement of the model towards energetically-favourable amino acid positions for ligand binding. The cCaSR model was iteratively improved, i.e., we first screened a number of compounds to test our hypothesis and then modelled the collected in vitro data to refine the binding model (Fig. 3). The initial models may have had the positioning of the loops and amino acids unresolved, and the experimental results from the in vitro work helped improve the models. The refinement of the models mostly involved the adjustment of sidechain rotamers. Discovery Studio and Gromacs software packages were used for model refinement.

### Taste choice tests with CaSR agonists using cat water panel testing

#### Animals

Healthy neutered adult domestic short hair cats (*Felis catus*) formed the panel for all the choice tests described here (n = 24). All 24 cats in the panel had previously been habituated and screened to prove suitability to perform this type of experiment. The cats had an average weight of 4.4 kg, average age of 4.8 years, and the panel was composed of 13–15 female and 11–9 male cats during the experiments. All the cats taking part in the panel were individually fed dry adult cat food twice per day to their individual Maintenance Energy Requirements (MER). On test days, the cats were housed during 18 h overnight in purpose-built, behaviourally-enriched lodges^58^. During the 18 h exposure, the cats only had access to the water solutions in the two-bottle testing apparatus, however they had free access to drinking water for the rest of the day when they were housed in a social group within an environmentally enriched room. All accommodation complied with the Code of Practice for Animal (Scientific Procedures) Act 1986.

#### Taste stimuli

All solutions were made with deionised water (Purite, UK). All taste-active substances were food-grade or of the highest available purity. In some cases, salts were purchased instead of the pure compound due to solubility, stability or availability reasons.

#### Experimental protocol

The in vivo testing was a two-bottle choice test and the methodology was adapted from^23^. All animal studies were in alignment with the Mars Animal Research Policy (Mars.com). These studies follow the 3Rs approach to experimentation with animals in scientific research^59^ and were approved by the Waltham Animal Welfare and Ethical Review Board, and all the methods were carried-out in accordance with local guidelines and regulations, including the ARRIVE guidelines^60^. On a given test day, the animals were placed in their individual lodges where they were offered two bottles containing 350 ml of solution each. The drinkers contained the test solution or the control solution, and the position of the drinkers presented to the cats was changed every day to control for any side bias, and each solution was offered twice to each cat. The cats were also offered at the same time as the water solutions, 50% of their daily food allowance. After 30 min. the food was removed and the cats were left in their individual lodges overnight, with access to the drinkers for 18 h. The next day the drinkers were weighed, and the remaining quantities were recorded as refused amounts. Total intakes were calculated using the difference of the offered and refused amounts. A correction for evaporation and any solution spillage was also performed on each data point.

#### Data analysis

Using a bespoke statistical analysis toolkit, based on R software (R Foundation, Vienna, Austria), a mixed model analysis was performed on the difference in intake (g) between water solutions containing the different taste compound, including cat (random) as a factor and weighting by grouped cat specific variability. This was used to test the mean intake difference versus 0, i.e. no difference, at a 5% level. The mean difference in intake was reported with a 95% confidence interval. All individual tests were then combined into a single model and the difference between the tastant pairs (i.e. both conditions containing the kokumi tastant in water vs. in the umami amino acid mix) were directly compared to measure the impact of the compound on the intake of either water or the umami mix. Any data errors (e.g. following manual error such as solution spillage, overflow) were removed from the final data set for analysis.

### Kokumi taste identification test with human sensory panel

#### Preparation of tastant solutions

All solutions were prepared using a commercial mineral water, Highland Spring (Highland Spring group, Blackford, UK) previously tested on our taste sensory panel and found to have the lowest level of inherent taste, thus being the least disruptive to taste tests. All taste-active substances were food-grade or of the highest available purity.

For tests with CaCl_2_, MgCl_2_, and GSH, each compound was dissolved directly in water, at 8 mM, 8 mM and 0.65 mM, respectively. Each compound was also tested in a mixture of umami and salty compounds, previously described in kokumi sensory evaluation^8^, composed of 0.02% MSG, 0.02% IMP and 0.07% NaCl.

#### Triangle test

The human sensory panel experimental protocols were approved by the Waltham Animal Welfare and Ethical Review Board and all methods were carried-out in accordance with all local regulations and guidelines. Informed consent was obtained from all subjects, and all participants were over the legal age of consent. All assessors used for the tests were validated following the process outlined in ISO 8586:2012(E). Briefly, all assessors were evaluated on their capacity to recognise tastants from blank solutions and successfully identify the basic taste modalities presented. The panellists were also assessed on their ability to correctly rank solutions of a tastant in ascending concentration order. The measures for successful validation of panellists were applied to all who completed the assessment.

For the kokumi tastant tests, six tests in all were completed, two with each CaSR agonist chosen CaCl_2_, MgCl_2_, or GSH. The number of participating panellists for each test varied between twenty-four and twenty-five. At an α of 0.05, a minimum of twenty-four assessors were used to ensure the tests had a 95% chance for 50% of the assessors to detect differences between samples. No replicate evaluations were done by the same assessor. For each compound, the assessors were presented with three separate forced choice triangle tests. In the first test, the compound was dissolved in spring water and compared to a blank of pure spring water, in the second the compound was dissolved into the previously described umami mix^8^ and compared to a blank of the umami mix. In each separate test, the panellists were presented with the samples in a randomised order to eliminate potential bias. Panellists were presented either two blanks and a sample or two samples and a blank and asked to identify the sample that was different in the triad. Panellists were asked to give a brief description of why the sample chosen was different. If they were unable to detect a difference, the assessors were instructed to choose a sample at random and specify in the description that they were unable to make a difference between the samples^61^.

#### Data analysis

The data was analysed for statistical significance using a standard methodology described in^61^. For this, the number of correct answers were compared to the number of assessors in each test, at the α-risk level chosen for the test. If the number of correct answers was equal or superior to the value given in the test table^61^, we concluded there was a perceptible difference between the solutions presented. As determined by a one tailed test, the minimum number of panellists required to successfully determine difference between samples was thirteen for the chosen α value^61^.

Initially, a contingency table of the number of correct answers and the number of wrong answers was created for each test. Then, Pearson’s chi-square test was then applied to the resulting table, with the probability of a correct answer at random being 1/ 3, while the probability of a wrong answer was 2/ 3. A p-value of ≤ 0.05 was deemed to indicate that the panellists were able to significantly detect the test solution correctly.

## Supplementary Information

Supplementary Information.
