# Supplementary material for: Kokumi taste perception is functional in a model carnivore, the domestic cat (Felis catus)
Source: Sci Rep. 2021 May 18;11:10527. doi: 10.1038/s41598-021-89558-w (PMC8131363; doi:10.1038/s41598-021-89558-w)
Supplement: Supplementary file 1 — Supplementary Information. [file 41598_2021_89558_MOESM1_ESM.pdf]

Kokumi taste perception is functional in a model carnivore, the domestic cat (*Felis catus*) – Supplementary Data

Laffitte A.[1†], Gibbs M.[1†], Hernangomez de Alvaro C.[1], Addison J.[1], Lonsdale ZN[1]., Giribaldi MG.[2,3], Rossignoli A.[2,3], Vennegeerts T.[2,3], Winnig M.[2,3], Klebansky B.[4], Skiles J.[4,5], Logan DW.[1], McGrane SJ.[1]

[1]WALTHAM Petcare Science Institute, Freeby Lane, Waltham on the Wolds, Melton Mowbray, Leicestershire, LE14 4RT, United Kingdom.

[2]IMAX Discovery GmbH, Otto-Hahn-Straße 15, 44227 Dortmund, Germany.

[3]AXXAM S.p,A,, OpenZone, Via Meucci 3, Bresso, MI 20091, Italy.

[4]BioPredict, Inc., 4 Adele Avenue, Demarest, NJ 07627, USA.

[5]Current Address: Valis Pharma, Ins., 545 Bonair Way, La Jolla, CA 92037, USA

[†]These authors contributed equally to this work.

Corresponding author: scott.mcgrane@effem.com

CaSR expression in cat circumvallate papillae RT-PCR gels

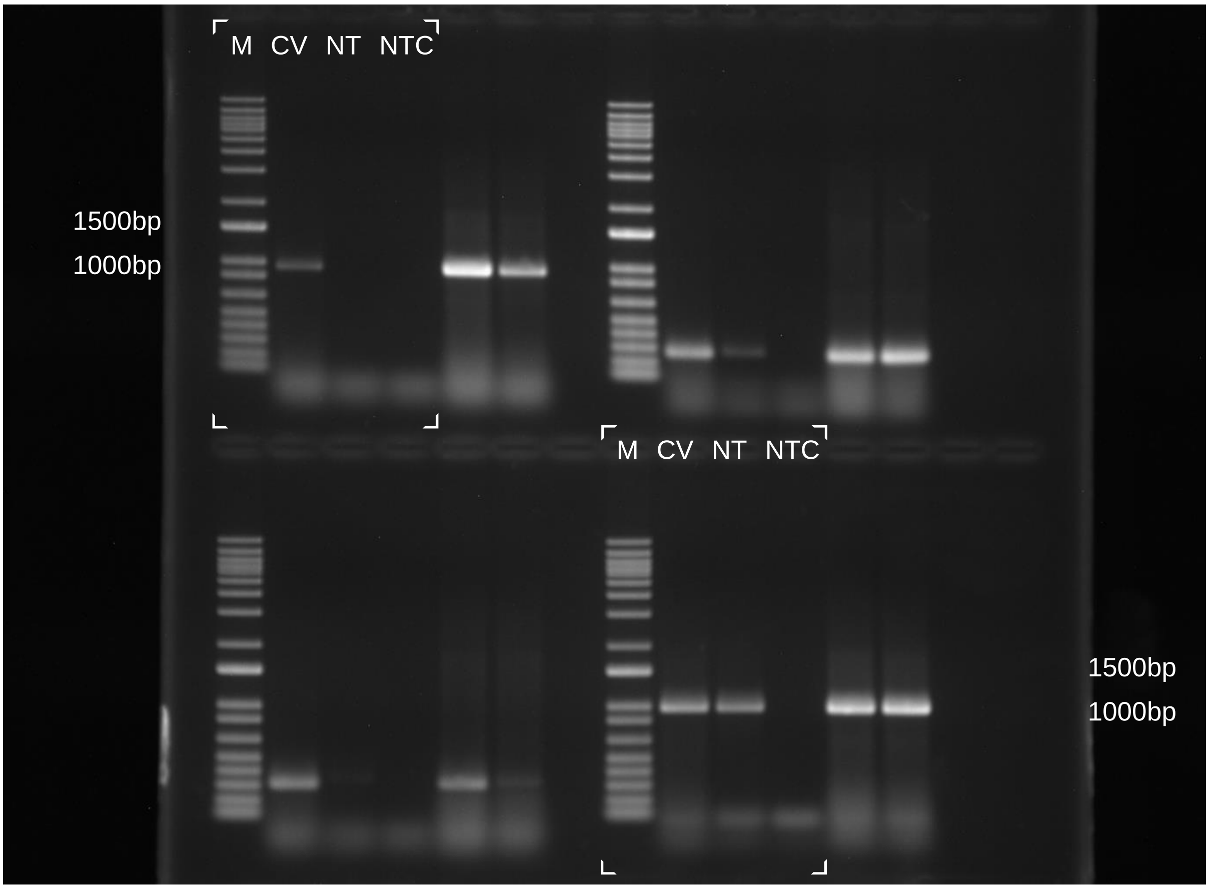

Supplementary Figure 1: *Full gel corresponding to Figure 1 in the manuscript, with CaSR expression (white brackets upper left-hand corner) and GAPDH expression (white brackets lower right-hand corner). Other samples shown are not relevant to the work presented in the paper. CV-circumvallate papilla, NT-non-taste epithelial tissue, NTC- no-template controls, M - molecular size marker.*

CaSR phylogenetic tree

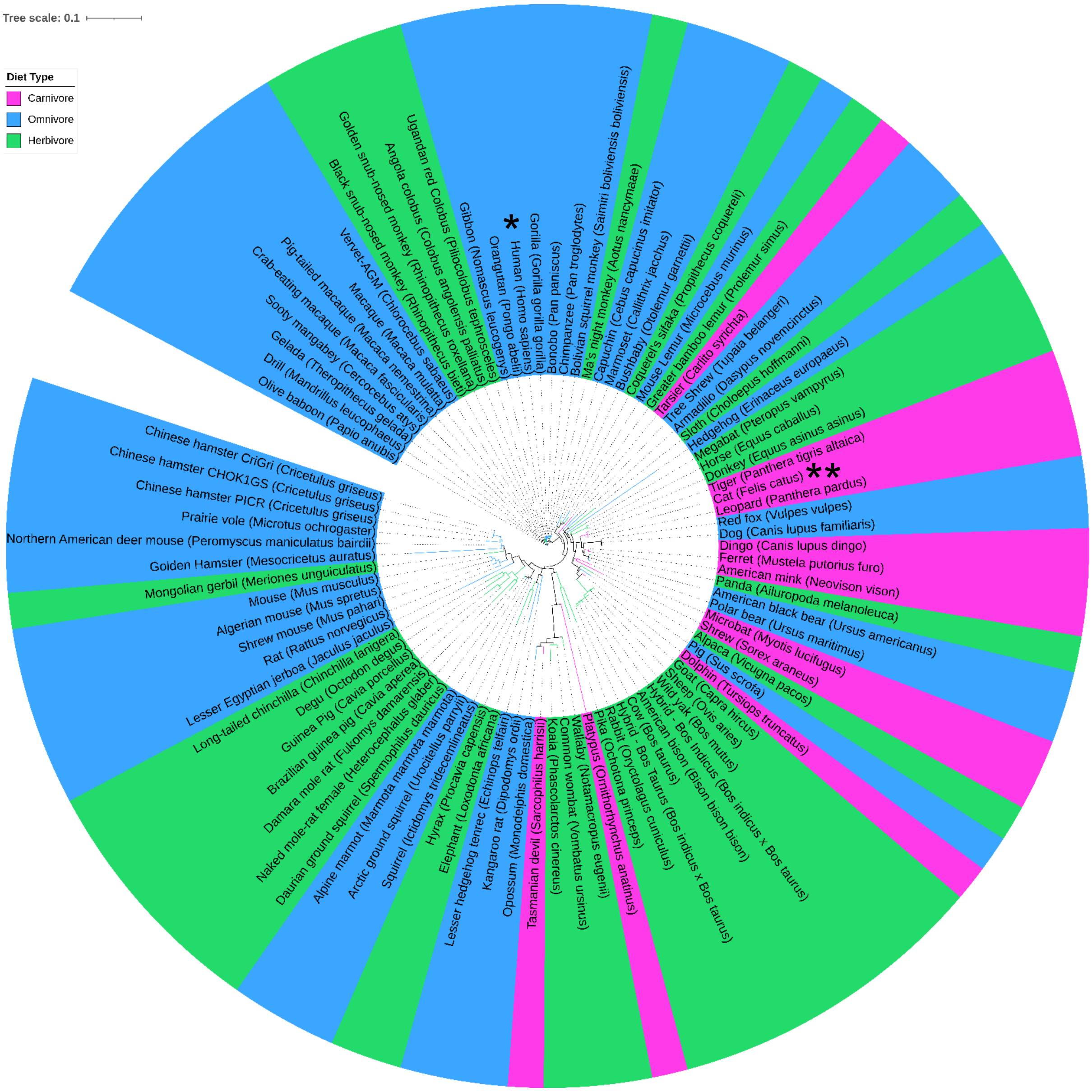

Supplementary Figure 2: *CaSR phylogenetic tree of mammalian species. Labels are coloured by diet type (green = herbivore; blue = omnivore; pink = carnivore). * - Human (Homo sapiens) ** - Cat (Felis catus).*

Mammalian CaSR Sequence alignment

```
CLUSTAL O(1.2.4) multiple sequence alignment


ENSMEUP00000013310_Neug/1-956           ------------------------------------------------------------   0
ENSTBEP00000002487_Tbel/1-914           ------------------------------------------------------------   0
ENSPCAP00000000755_Pcap/1-1085          ----------------------------MAFYSCCLMLLAF-AWRTSAYGPDQRAQKKGD   31
ENSCHOP00000011907_Chof/1-954           ----------------------------MAFYSCCVILLAS-VWCTSAYGPDQRAQKKGD   31
ENSVPAP00000001982_Vpac/1-1087          ----------------------------MASYSCCWILLAF-AWCASAYGPDQRAQKKGD   31
ENSEEUP00000009670_Eeur/1-946           ----------------------------MAFYCYCLILLAF-TWDISAYGPDQRAQKKGD   31
ENSOANP00000015961_Oana/1-1083          ----------------------------MTRFGCWLVLAAF-PWALSAYGPDQRAQKKGD   31
ENSOPRP00000000261_Opri/1-1082          ----------------------------MALHSHCLILLAL-AWHSSAYGPDQRAQKKGD   31
ENSJJAP00000004721_Jjac/1-1084          ----------------------------MVGYSSCLVLLALACLHASAYGPDQRAQKKGD   32
ENSPCIP00000034747_Pcin/1-1018          ----------------------------MTYYRSCLILLAF-TWNASAYGPDQRAQKKGD   31
ENSMODP00000022810_Mdom/1-1069          ----------------------------MTYYRCCLILLAL-TWNTSAYGPDQRAQKKGD   31
ENSVURP00010007344_Vurs/1-1070          ----------------------------MTYYRCCLILLAF-TWNISAYGPDQRAQKKGD   31
ENSSHAP00000014890_Shar/1-1072          ----------------------------MTFYRCCLVLLAF-TWNTSAYGPDQRAQKKGD   31
ENSMAUP00000005394_Maur/1-954           ----------------------------MASYSCCLALLAL-AWHSSAYGPDQRAQKKGD   31
ENSOCUP00000025672_Ocun/1-1063          ----------------------------MAFPSHCLIFLAL-AWYSSAYGPDQRAQKKGD   31
ENSPVAP00000011981_Pvam/1-1085          ----------------------------MALHSCCLILLAF-TWLTSAYGPDQRAQKKGD   31
ENSETEP00000011160_Etel/1-1024          ------------------------------------------------------------   0
ENSFDAP00000010532_Fdam/1-1108          MSGSKGESFRGSQTPLTTVSLPWREGRIMASYSCCLVLLAL-AWHASAYGPDQRAQKKGD   59
ENSODEP00000013257_Odeg/1-1070          ----------------------------MVSYGCCLVLLAL-AWYSSAYGPDQRAQKKGD   31
ENSHGLP00000002019_Hgfe/1-1108          MSCSKRESSQGPQITITIESLSWREGRIMASYSCCLVLLAL-AWHSSAYGPDQRAQKKGD   59
ENSCLAP00000021334_Clan/1-1114          MSCTSGESSRGPQTTIIIMSLAWREGRIMASCGCCLVLLAL-TWQVSAYGPDQRAQKKGD   59
ENSCAPP00000014352_Cape/1-1068          ----------------------------MAFYSCCLVLLVL-AWHSSAYGPDQRAQKKGD   31
ENSCPOP00000011705_Cpor/1-1071          ----------------------------MAFYSCCLVLLVL-AWHSSAYGPDQRAQKKGD   31
ENSSARP00000006151_Sara/1-1085          ----------------------------MAFYSCCLLLLAF-TWNTSAYGPDQRAQKKGD   31
ENSLAFP00000018459_Lafr/1-1090          ----------------------------MAFYSRCLILLAF-AWRPAAYGPDQRAQKKGD   31
ENSDNOP00000011819_Dnov/1-1075          ----------------------------MAFYSCSLILLAF-AWCTSAYGPDQRAQKKGD   31
ENSSSCP00000041077_Sscr/1-1089          ----------------------------MAFSSCCWILLAL-TWCTSAYGPDQRAQKKGD   31
ENSTTRP00000001326_Ttru/1-1091          ----------------------------MAFYSCCWIFLAF-TWCTSAYGPDQRAQKKGD   31
ENSBBBP00000002858_Bbbi/1-1068          ----------------------------MALYSCCWILLAFSTWCTSAYGPDQRAQKKGD   32
ENSOARP00000021602_Oari/1-1032          ----------------------------MALYSCCWILLAFSTWCTSAYGPDQRAQKKGD   32
ENSCHIP00000033385_Chir/1-1085          ----------------------------MALNSCCWILLAFSTWCTSAYGPDQRAQKKGD   32
ENSBTAP00000059078_Btau/1-1085          ----------------------------MALYSCCWILLAFSTWCTSAYGPDQRAQKKGD   32
ENSBIXP00000038534_Bthy/1-1085          ----------------------------MALYSCCWILLAFSTWCTSAYGPDQRAQKKGD   32
ENSBMUP00000019115_Bmut/1-1081          ----------------------------MALYSCCWILLAFSTWCTSAYGPDQRAQKKGD   32
ENSBIXP00005030570_Bihy/1-1085          ----------------------------MALYSCCWILLAFSTWCTSAYGPDQRAQKKGD   32
ENSMUGP00000006365_Mung/1-1080          ----------------------------MASYSCFLALLAL-AWHSSAYGPDQRAQKKGD   31
ENSRNOP00000069629_Rnor/1-1079          ----------------------------MASYSCCLALLAL-AWHSSAYGPDQRAQKKGD   31
MGP_PahariEiJ_P0028852_Mpah/1-1003      ----------------------------MAWYGCCLALLAL-AWHSSAYGPDQRAQKKGD   31
MGP_SPRETEiJ_P0043347_Mspr/1-1079       ----------------------------MAWFGYCLALLAL-TWHSSAYGPDQRAQKKGD   31
ENSMUSP00000069080_Mmus/1-1079          ----------------------------MAWFGYCLALLAL-TWHSSAYGPDQRAQKKGD   31
ENSMOCP00000010340_Moch/1-1076          ----------------------------MASYSCCLALLAL-ACHSSAYGPDQRAQKKGD   31
ENSPEMP00000029995_Pmba/1-1076          ----------------------------MAPYSCCLALLAL-AWHSSAYGPDQRAQKKGD   31
ENSCGRP00015024036_Cgpi/1-1081          ----------------------------MASYSCCLALLAL-AWHSSAYGPDQRAQKKGD   31
ENSCGRP00001016053_Cgch/1-1081          ----------------------------MASYSCCLALLAL-AWHSSAYGPDQRAQKKGD   31
ENSCGRP00000017369_Cgcr/1-1081          ----------------------------MASYSCCLALLAL-AWHSSAYGPDQRAQKKGD   31
ENSDORP00000002959_Dord/1-1078          ----------------------------MVLYSNCLILWAL-AWLSSAYGPDQRAQKKGD   31
ENSTSYP00000002643_Csyr/1-1015          ----------------------------MAFYSCCWMLVAL-AWHTSAYGPDQRAQKKGD   31
ENSMLUP00000008249_Mluc/1-1075          ----------------------------MALYSCCLILLAF-TWHTSAYGPDQRAQKKGD   31
ENSECAP00000022285_Ecab/1-1084          ----------------------------MAFYSCCLILVAF-TWCTSAYGPDQRAQKKGD   31
ENSEASP00005022676_Eaas/1-1094          ----------------------------MAFYSCCLILVAF-TWCTSAYGPDQRAQKKGD   31
ENSCAFP00000017381_Cfam/1-1075          ----------------------------MAFHSCSLILLAI-TWCTSAYGPDQRAQKKGD   31
ENSCAFP00020007154_Cldi/1-1075          ----------------------------MAFHSCSLILLAI-TWCTSAYGPDQRAQKKGD   31
ENSVVUP00000030826_Vvul/1-1075          ----------------------------MAFHSCSLILLAI-TWCTSAYGPDQRAQKKGD   31
ENSFCAP00000008080_Fcat/1-1081          ----------------------------MAFYSCCLILLAI-TWCTSAYGPDQRAQKKGD   31
ENSPPRP00000023800_Ppar/1-1081          ----------------------------MAFYSCCLILLAI-TWCTSAYGPDQRAQKKGD   31
ENSPTIP00000015042_Ptal/1-1081          ----------------------------MAFYSCCLILLAI-TWCISAYGPDQRAQKKGD   31
ENSMPUP00000004759_Mpfu/1-1082          ----------------------------MAFYSCCFILLAS-TWCTSAYGPDQRAQKKGD   31
ENSNVIP00000018564_Nvis/1-1092          ----------------------------MAFYSCCFILLAS-TWCTSAYGPDQRAQKKGD   31
ENSUMAP00000035085_Umar/1-1071          ----------------------------MAFYSCCLTLLAI-TWCTSAYGPDQRAQKKGD   31
ENSUAMP00000035930_Uame/1-1079          ----------------------------MAFYSCCLTLLAI-TWCTSAYGPDQRAQKKGD   31
ENSAMEP00000015060_Amel/1-1089          ----------------------------MAFYSCCLTLLAI-TWCTSAYGPDQRAQKKGD   31
ENSSDAP00000007437_Sdau/1-1079          ----------------------------MVPHSCCLVLLAF-AWYSSAYGPDQRAQKKGD   31
ENSSTOP00000003945_Itri/1-1070          ----------------------------MVPHSCCLVLLAL-AWYSSAYGPDQRAQKKGD   31
ENSMMMP00000015298_Mmma/1-1083          ----------------------------MVPHSCCLVLLAF-AWYSSAYGPDQRAQKKGD   31
ENSUPAP00010025947_Upar/1-1086          ----------------------------MVPHSCCLVLLAF-AWYSSAYGPDQRAQKKGD   31
ENSSBOP00000036416_Sbbo/1-1078          ----------------------------MAFYSCCWVLLAL-TWHSSAYGPDQRAQKKGD   31
ENSANAP00000028404_Anan/1-1078          ----------------------------MAFYSCCWVLLAL-AWHTSAYGPDQRAQKKGD   31
ENSCCAP00000024327_Ccap/1-1077          ----------------------------MAFYSCCWVLLAL-TWHTSAYGPDQRAQKKGD   31
ENSCJAP00000062854_Cjac/1-1089          ----------------------------MAFYSCCWVLLAL-TWHTSAYGPDQRAQKKGD   31
ENSNLEP00000000336_Nleu/1-1088          ----------------------------MAFYSCCWVLLAL-TWHTSAYGPDQRAQKKGD   31
ENSPPYP00000015087_Pabe/1-1078          ----------------------------MAFYSCCWVLLAL-TWHTSAYGPDQRAQKKGD   31
ENSP00000420194_Hsap/1-1088             ----------------------------MAFYSCCWVLLAL-TWHTSAYGPDQRAQKKGD   31
ENSGGOP00000020287_Ggor/1-1078          ----------------------------MAFYSCCWVLLAL-TWHTSAYGPDQRAQKKGD   31
ENSPPAP00000026371_Ppan/1-1078          ----------------------------MAFYSCCWVLLAL-TWHTSAYGPDQRAQKKGD   31
ENSPTRP00000043342_Ptro/1-1078          ----------------------------MAFYSCCWVLLAL-TWHTSAYGPDQRAQKKGD   31
ENSCSAP00000003635_Csab/1-882           ------------------------------------------------------------   0
ENSMMUP00000061002_Mmul/1-1078          ----------------------------MAFYCCFWVLLAL-TWHTSAYGPNQRAQKKGD   31
ENSPANP00000004316_Panu/1-1081          -----------------------------------WVLLAL-TWHTSAYGPDQRAQKKGD   24
ENSCATP00000028615_Caty/1-1078          ----------------------------MAFYCCFWVLLAL-TWHTSAYGPDQRAQKKGD   31
ENSMFAP00000042132_Mfas/1-1078          ----------------------------MAFYCCFWVLLAL-TWHTSAYGPDQRAQKKGD   31
```

ENSMNEP00000024602_Mnem/1-1078	----------------------------MAFYCCFWVLLAL-TWHTSAYGPDQRAQKKGD	31
ENSMLEP00000008744_Mleu/1-1088	----------------------------MAFYCCFWVLLAL-TWHTSAYGPDQRAQKKGD	31
ENSTGEP00000008766_Tgel/1-1088	----------------------------MAFYCCFWVLLAL-TWHTSAYGPDQRAQKKGD	31
ENSCANP00000040262_Capa/1-1088	----------------------------MAFYCCFWVLLAL-TWHTSAYGPDQRAQKKGD	31
ENSPTEP00000016080_Ptep/1-1088	----------------------------MAFYCCFWVLLAL-TWHTSAYGPDQRAQKKGD	31
ENSRBIP00000006831_Rbie/1-1078	----------------------------MAFYCCFWVLLAL-TWHTSAYGPDQRAQKKGD	31
ENSRROP00000007818_Rrox/1-1088	----------------------------MAFYCCFWVLLAL-TWHTSAYGPDQRAQKKGD	31
ENSOGAP00000002850_Ogar/1-1077	----------------------------MAFYSYCGILLAL-AWHSSAYGPDQRAQKKGD	31
ENSMICP00000050654_Mmur/1-1075	----------------------------MAFYSCCGILLAL-AWHTSAYGPDQRAQKKGD	31
ENSPCOP00000009624_Pcoq/1-1076	----------------------------MAFYSCCGILLAL-AWHTSAYGPDQRAQKKGD	31
ENSPSMP00000007909_Psim/1-1084	----------------------------MAFYSCCGILLAL-AWHTSAYGPDQRAQKKGD	31

ENSMEUP00000013310_Neug/1-956	-------------------------------YNFRGFRWLQAMIFAIEEINSSPALLPNM	29
ENSTBEP00000002487_Tbel/1-914	--	0
ENSPCAP00000000755_Pcap/1-1085	IILGGLFPIHFGVAAKDQDLKSRPESVECIRYNFRGFRWLQAMIFAIEEINSSPVLLPNM	91
ENSCHOP00000011907_Chof/1-954	IILGGLFPIHFGVAAKDQDLKSRPESVECIRYNFRGFRWLQAMIFAIEEINSSPALLPNM	91
ENSVPAP00000001982_Vpac/1-1087	IILGGLFPIHFGVAAKDQDLKSRPESVECIRYNFRGFRWLQAMIFAIEEINSSPALLPNM	91
ENSEEUP00000009670_Eeur/1-946	IILGGLFPIHFGVAAKDQDLKSRPESVECIRYNFRGFRWLQAMIFAIEEINSSPALLPNM	91
ENSOANP00000015961_Oana/1-1083	IILGGLFPIHFGVAAKDQDLKSRPESVECIRYNFRGFRWLQAMIFAIEEINSSPALLPNM	91
ENSOPRP00000000261_Opri/1-1082	IILGGLFPIHFGVAAKDQDLKSRPESVECIRYNFRGFRWLQAMIFAIEEINSSPALLPNM	91
ENSJJAP00000004721_Jjac/1-1084	IILGGLFPIHFGVAAKDQDLKSRPESVECIRYNFRGFRWLQAMIFAIEEINSSPALLPNM	92
ENSPCIP00000034747_Pcin/1-1018	IILGGLFPIHFGVAAKDQDLKSRPESVKCIRYNFRGFRWLQAMIFAIEEINSSPALLPNM	91
ENSMODP00000022810_Mdom/1-1069	IILGGLFPIHFGVAAKDQDLKSRPESVECIRYNFRGFRWLQAMIFAIEEINNSPALLPNM	91
ENSVURP00010007344_Vurs/1-1070	IILGGLFPIHFGVAAKDQDLKSRPESVECIRYNFRGFRWLQAMIFAIEEINSSPALLPNM	91
ENSSHAP00000014890_Shar/1-1072	IILGGLFPIHFGVAAKDQDLKSRPESVECIRYNFRGFRWLQAMIFAIEEINNSPSLLPNM	91
ENSMAUP00000005394_Maur/1-954	IILGGLFPIHFGVAAKDQDLKSRPESVECIRYNFRGFRWLQAMIFAIEEINSSPRLLPNM	91
ENSOCUP00000025672_Ocun/1-1063	IILGGLFPIHFGVAAKDQDLKSRPESVECIRYNFRGFRWLQAMIFAIEEINSSPALLPNM	91
ENSPVAP00000011981_Pvam/1-1085	IILGGLFPIHFGVAAKDQDLKSRPESVECIRYNFRGFRWLQAMIFAIEEINSSPALLPNM	91
ENSETEP00000011160_Etel/1-1024	-------------------------------YNFRGFRWLQAMIFAIEEINSSPALLPNM	29
ENSFDAP00000010532_Fdam/1-1108	IILGGLFPIHFGVAAKDQDLKSRPESVECIRYNFRGFRWLQAMIFAIEEINSSPALLPNL	119
ENSODEP00000013257_Odeg/1-1070	IILGGLFPIHFGVAAKDQDLKSRPESVECIRYNFRGFRWLQAMIFAIEEINSSPALLPNM	91
ENSHGLP00000002019_Hgfe/1-1108	IILGGLFPIHFGVAAKDQDLKSRPESVECIRYNFRGFRWLQAMIFAIEEINSSPALLPNM	119
ENSCLAP00000021334_Clan/1-1114	IILGGLFPIHFGVAAKDQGLKSRPESVECVRYNFRGFRWLQAMIFAIEEINSSPTLLPNM	119
ENSCAPP00000014352_Cape/1-1068	IILGGLFPIHFGVAAKDQDLESRPESVECIRYNFRGFRWLQAMIFAIEEINSSPALLPNM	91
ENSCPOP00000011705_Cpor/1-1071	IILGGLFPIHFGVAAKDQDLESRPESVECIRYNFRGFRWLQAMIFAIEEINSSPALLPNM	91
ENSSARP00000006151_Sara/1-1085	IILGGLFPIHFGVAAKDQDLKSRPESVECIRYNFRGFRWLQAMIFAIEEINSSPVCLSNM	91
ENSLAFP00000018459_Lafr/1-1090	IILGGLFPIHFGVAAKDQDLKSRPESVECIRYNFRGFRWLQAMIFAIEEINSSPVLLPNM	91
ENSDNOP00000011819_Dnov/1-1075	IILGGLFPIHFGVAAKDQDLKSRPESVECIRYNFRGFRWLQAMIFAIEEINSSPALLPNM	91
ENSSSCP00000041077_Sscr/1-1089	IILGGLFPIHFGVAAKDQNLESRPESVECIRYNFRGFRWLQAMIFAIEEINSSPALLPNM	91
ENSTTRP00000001326_Ttru/1-1091	IILGGLFPIHFGVAAKDQDLKSRPESVECIRYNFRGFRWLQAMIFAIEEINSSPALLPNM	91
ENSBBBP00000002858_Bbbi/1-1068	IILGGLFPIHFGVAAKDQDLKSRPESVECIRYNFRGFRWLQAMIFAIEEINSSPALLPNM	92
ENSOARP00000021602_Oari/1-1032	IILGGLFPIHFGVAAKDQDLKSRPESVECIRYNFRGFRWLQAMIFAIEEINSSPALLPNM	92
ENSCHIP00000033385_Chir/1-1085	IILGGLFPIHFGVAAKDQDLKSRPESVECIRYNFRGFRWLQAMIFAIEEINSSPALLPNM	92
ENSBTAP00000059078_Btau/1-1085	IILGGLFPIHFGVAVKDQDLKSRPESVECIRYNFRGFRWLQAMIFAIEEINSSPALLPNM	92
ENSBIXP00000038534_Bthy/1-1085	IILGGLFPIHFGVAVKDQDLKSRPESVECIRYNFRGFRWLQAMIFAIEEINSSPALLPNM	92
ENSBMUP00000019115_Bmut/1-1081	IILGGLFPIHFGVAAKDQDLKSRPESVECIRYNFRGFRWLQAMIFAIEEINSSPALLPNM	92
ENSBIXP00005030570_Bihy/1-1085	IILGGLFPIHFGVAAKDQDLKSRPESVECIRYNFRGFRWLQAMIFAIEEINSSPALLPNM	92
ENSMUGP00000006365_Mung/1-1080	IILGGLFPIHFGVAAKDQDLKSRPESVECIRYNFRGFRWLQAMIFAIEEINSSPALLPNL	91
ENSRNOP00000069629_Rnor/1-1079	IILGGLFPIHFGVAAKDQDLKSRPESVECIRYNFRGFRWLQAMIFAIEEINSSPSLLPNM	91
MGP_PahariEiJ_P0028852_Mpah/1-1003	IILGGLFPIHFGVAAKDQDLKSRPESVECIRYNFRGFRWLQAMIFAIEEINSSPALLPNM	91
MGP_SPRETEiJ_P0043347_Mspr/1-1079	IILGGLFPIHFGVAAKDQDLKSRPESVECIRYNFRGFRWLQAMIFAIEEINSSPTLLPNM	91
ENSMUSP00000069080_Mmus/1-1079	IILGGLFPIHFGVAAKDQDLKSRPESVECIRYNFRGFRWLQAMIFAIEEINSSPALLPNM	91
ENSMOCP00000010340_Moch/1-1076	IILGGLFPIHFGVAAKDQDLKSRPESVECIRYNFRGFRWLQAMIFAIEEINSSPTLLPNM	91
ENSPEMP00000029995_Pmba/1-1076	IILGGLFPIHFGVAAKDQDLKSRPESVECIRYNFRGFRWLQAMIFAIEEINSSPALLPNM	91
ENSCGRP00015024036_Cgpi/1-1081	IILGGLFPIHFGVAAKDQDLKSRPESVECIRYNFRGFRWLQAMIFAIEEINSSPALLPNM	91
ENSCGRP00001016053_Cgch/1-1081	IILGGLFPIHFGVAAKDQDLKSRPESVECIRYNFRGFRWLQAMIFAIEEINSSPALLPNM	91
ENSCGRP00000017369_Cgcr/1-1081	IILGGLFPIHFGVAAKDQDLKSRPESVECIRYNFRGFRWLQAMIFAIEEINSSPALLPNM	91
ENSDORP00000002959_Dord/1-1078	IILGGLFPIHFGVAAKDQDLKSRPESVECIRYNFRGFRWLQAMIFAIEEINSSPALLPNM	91
ENSTSYP00000002643_Csyr/1-1015	IILGGLFPIHFGVAAKDQDLKSRPESVECIRYNFRGFRWLQAMIFAIEEINSSPALLPNM	91
ENSMLUP00000008249_Mluc/1-1075	IILGGLFPIHFGVAAKDQDLKSRPESVECIRYNFRGFRWLQAMIFAIEEINSSPALLPNM	91
ENSECAP00000022285_Ecab/1-1084	IILGGLFPIHFGVAAKDQDLESRPESVECIRYNFRGFRWLQAMIFAIEEINSSPALLPNM	91
ENSEASP00005022676_Eaas/1-1094	IILGGLFPIHFGVAAKDQDLESRPESVECIRYNFRGFRWLQAMIFAIEEINSSPALLPNM	91
ENSCAFP00000017381_Cfam/1-1075	IILGGLFPIHFGVAAKDQDLKSRPESVECIRYNFRGFRWLQAMIFAIEEINSSPALLPNM	91
ENSCAFP00020007154_Cldi/1-1075	IILGGLFPIHFGVAAKDQDLKSRPESVECIRYNFRGFRWLQAMIFAIEEINSSPALLPNM	91
ENSVVUP00000030826_Vvul/1-1075	IILGGLFPIHFGVAAKDQDLKSRPESVECIRYNFRGFRWLQAMIFAIEEINSSPALLPNM	91
ENSFCAP00000008080_Fcat/1-1081	IILGGLFPIHFGVAAKDQDLKSRPESVECIRYNFRGFRWLQAMIFAIEEINSSPVLLPNM	91
ENSPPRP00000023800_Ppar/1-1081	IILGGLFPIHFGVAAKDQDLKSRPESVECIRYNFRGFRWLQAMIFAIEEINSSPVLLPNM	91
ENSPTIP00000015042_Ptal/1-1081	IILGGLFPIHFGVAAKDQDLKSRPESVECIRYNFRGFRWLQAMIFAIEEINSSPVLLPNM	91
ENSMPUP00000004759_Mpfu/1-1082	IILGGLFPIHFGVAAKDQDLKSRPESVECIRYNFRGFRWLQAMIFAIEEINSSPALLPNM	91
ENSNVIP00000018564_Nvis/1-1092	IILGGLFPIHFGVAAKDQDLKSRPESVECIRYNFRGFRWLQAMIFAIEEINSSPALLPNM	91
ENSUMAP00000035085_Umar/1-1071	IILGGLFPIHFGVAAKDQDLKSRPESVECIRYNFRGFRWLQAMIFAIEEINSSPALLPNM	91
ENSUAMP00000035930_Uame/1-1079	IILGGLFPIHFGVAAKDQDLKSRPESVECIRYNFRGFRWLQAMIFAIEEINSSPALLPNM	91
ENSAMEP00000015060_Amel/1-1089	IILGGLFPIHFGVAAKDQDLKSRPESVECIRYNFRGFRWLQAMIFAIEEINSSPALLPNM	91
ENSSDAP00000007437_Sdau/1-1079	IILGGLFPIHFGVAAKDQDLKSRPESVECIRYNFRGFRWLQAMIFAIEEINSSPALLPNM	91
ENSSTOP00000003945_Itri/1-1070	IILGGLFPIHFGVAAKDQDLKSRPESVECIRYNFRGFRWLQAMIFAIEEINSSPALLPNM	91
ENSMMMP00000015298_Mmma/1-1083	IILGGLFPIHFGVAAKDQDLKSRPESVECIRYNFRGFRWLQAMIFAIEEINSSPALLPNM	91
ENSUPAP00010025947_Upar/1-1086	IILGGLFPIHFGVAAKDQDLKSRPESVECIRYNFRGFRWLQAMIFAIEEINSSPALLPNM	91
ENSSBOP00000036416_Sbbo/1-1078	IILGGLFPIHFGVAAKDQDLKSRPESVECIRYNFRGFRWLQAMIFAIEEINSSPALLPNL	91
ENSANAP00000028404_Anan/1-1078	IILGGLFPIHFGVAAKDQDLKSRPESVECIRYNFRGFRWLQAMIFAIEEINSSPALLPNL	91
ENSCCAP00000024327_Ccap/1-1077	IILGGLFPIHFGVAAKDQDLKSRPESVECIRYNFRGFRWLQAMIFAIEEINSSPALLPNL	91
ENSCJAP00000062854_Cjac/1-1089	IILGGLFPIHFGVAAKDQDLKSRPESVECIRYNFRGFRWLQAMIFAIEEINSSPALLPNL	91
ENSNLEP00000000336_Nleu/1-1088	IILGGLFPIHFGVAAKDQDLKSRPESVECIRYNFRGFRWLQAMIFAIEEINSSPALLPNL	91
ENSPPYP00000015087_Pabe/1-1078	IILGGLFPIHFGVAAKDQDLKSRPESVECIRYNFRGFRWLQAMIFAIEEINSSPALLPNL	91
ENSP00000420194_Hsap/1-1088	IILGGLFPIHFGVAAKDQDLKSRPESVECIRYNFRGFRWLQAMIFAIEEINSSPALLPNL	91
ENSGGOP00000020287_Ggor/1-1078	IILGGLFPIHFGVAAKDQDLKSRPESVECIRYNFRGFRWLQAMIFAIEEINSSPALLPNL	91
ENSPPAP00000026371_Ppan/1-1078	IILGGLFPIHFGVAVKDQDLKSRPESVECIRYNFRGFRWLQAMIFAIEEINSSPALLPNL	91
ENSPTRP00000043342_Ptro/1-1078	IILGGLFPIHFGVAAKDQDLKSRPESVECIRYNFRGFRWLQAMIFAIEEINSSPALLPNL	91

```
ENSCSAP00000003635_Csab/1-882           ---------------------------------------------------------------   0
ENSMMUP00000061002_Mmul/1-1078          IILGGLFPIHFGVAAKDQDLKSRPESVECIRYNFRGFRWLQAMIFAIEEINSSPALLPNL   91
ENSPANP00000004316_Panu/1-1081          IILGGLFPIHFGVAAKDQDLKSRPESVECIRYNFRGFRWLQAMIFAIEEINSSPALLPNL   84
ENSCATP00000028615_Caty/1-1078          IILGGLFPIHFGVAAKDQDLKSRPESVECIRYNFRGFRWLQAMIFAIEEINSSPALLPNL   91
ENSMFAP00000042132_Mfas/1-1078          IILGGLFPIHFGVAAKDQDLKSRPESVECIRYNFRGFRWLQAMIFAIEEINSSPALLPNL   91
ENSMNEP00000024602_Mnem/1-1078          IILGGLFPIHFGVAAKDQDLKSRPESVECIRYNFRGFRWLQAMIFAIEEINSSPALLPNL   91
ENSMLEP00000008744_Mleu/1-1088          IILGGLFPIHFGVAAKDQDLKSRPESVECIRYNFRGFRWLQAMIFAIEEINSSPALLPNL   91
ENSTGEP00000008766_Tgel/1-1088          IILGGLFPIHFGVAAKDQDLKSRPESVECIRYNFRGFRWLQAMIFAIEEINSSPALLPNL   91
ENSCANP00000040262_Capa/1-1088          IILGGLFPIHFGVAAKDQDLKSRPESVECIRYNFRGFRWLQAMIFAIEEINSSPALLPNL   91
ENSPTEP00000016080_Ptep/1-1088          IILGGLFPIHFGVAAKDQDLKSRPESVECIRYNFRGFRWLQAMIFAIEEINSSPALLPNL   91
ENSRBIP00000006831_Rbie/1-1078          IILGGLFPIHFGVAAKDQDLKSRPESVECIRYNFRGFRWLQAMIFAIEEINSSPALLPNL   91
ENSRROP00000007818_Rrox/1-1088          IILGGLFPIHFGVAAKDQDLKSRPESVECIRYNFRGFRWLQAMIFAIEEINSSPALLPNL   91
ENSOGAP00000002850_Ogar/1-1077          IILGGLFPIHFGVAAKDQDLKSRPESVECIRYNFRGFRWLQAMIFAIEEINSSPALLPNM   91
ENSMICP00000050654_Mmur/1-1075          IILGGLFPIHFGVAAKDQDLKSRPESVECIRYNFRGFRWLQAMIFAIEEINSSPALLPNM   91
ENSPCOP00000009624_Pcoq/1-1076          IILGGLFPIHFGVAAKDQDLKSRPESVECIRYNFRGFRWLQAMIFAIEEINSSPALLPNM   91
ENSPSMP00000007909_Psim/1-1084          IILGGLFPIHFGVAAKDQDLKSRPESVECIRYNFRGFRWLQAMIFAIEEINSSPALLPNM   91


ENSMEUP00000013310_Neug/1-956           TLGYRIFDTCNTVSKALEATLSFVAQNKIDSLNLDEFCNCSEHIPSTIAVVGATGSGIST   89
ENSTBEP00000002487_Tbel/1-914           ------------------------------------------------------------   0
ENSPCAP00000000755_Pcap/1-1085          TLGYRIFDTCNTVSKALEATLSFVAQNKIDSLNLDEFCNCSEHIPSTIAVVGATGSGIST   151
ENSCHOP00000011907_Chof/1-954           TLGYRIFDTCNTVSKALEATLSFVAQNKIDSLNLDEFCNCSEHIPSTIAVVGATGSGVST   151
ENSVPAP00000001982_Vpac/1-1087          TLGYRIFDTCNTVSKALEATLSFVAQNKIDSLNLDEFCNCSEHIPSTIAVVGATGSGIST   151
ENSEEUP00000009670_Eeur/1-946           TLGYRIFDTCNTVSKALEATLSFVAQNKIDSLNLDEFCNCSEHIPSTIAVVGATGSGIST   151
ENSOANP00000015961_Oana/1-1083          TLGYRIFDTCNTVSKALEATLSFVAQNKIDSLNLDEFCNCSEHIPSTIAVVGATGSGIST   151
ENSOPRP00000000261_Opri/1-1082          TLGYRIFDTCNTVSKALEATLSFVAQNKIDSLNLDEFCNCSEHIPSTIAVVGATGSGVST   151
ENSJJAP00000004721_Jjac/1-1084          TLGYRIFDTCNTVSKALEATLSFVAQNKIDSLNLDEFCNCSEHIPSTIAVVGATGSGIST   152
ENSPCIP00000034747_Pcin/1-1018          TLGYRIFDTCNTVSKALEATLSFVVLG---------------------------------   118
ENSMODP00000022810_Mdom/1-1069          TLGYRIFDTCNTVSKALEATLSFVAQNKIDSLNLDEFCNCSEHIPSTIAVVGATGSGIST   151
ENSVURP00010007344_Vurs/1-1070          TLGYRIFDTCNTVSKALEATLSFVAQNKIDSLNLDEFCNCSEHIPSTIAVVGATGSGIST   151
ENSSHAP00000014890_Shar/1-1072          TLGYRIFDTCNTVSKALEATLSFVAQNKIDSLNLDEFCNCSEHIPSTIAVVGATGSGIST   151
ENSMAUP00000005394_Maur/1-954           TLGYRIFDTCNTVSKALEATLSFVAQNKIDSLNLDEFCNCSEHIPSTIAVVGATGSGVST   151
ENSOCUP00000025672_Ocun/1-1063          TLGYRIFDTCNTVSKALEATLSFVAQNKIDSLNLDEFCNCSEHIPSTIAVVGATGSGVST   151
ENSPVAP00000011981_Pvam/1-1085          TLGYRIFDTCNTVSKALEATLSFVAQNKIDSLNLDEFCNCSQHIPSTIAVVGATGSGIST   151
ENSETEP00000011160_Etel/1-1024          TLGYRIFDTCNTVSKALEATLSFVAQNKIDSLNLDEFCNCSEHIPSTIAVVGATGSGIST   89
ENSFDAP00000010532_Fdam/1-1108          TLGYRIFDTCNTVSKALEATLSFVAQNKIDSLNLDEFCNCSEHIPSTIAVVGATGSGVST   179
ENSODEP00000013257_Odeg/1-1070          TLGYRIFDTCNTVSKALEATLSFVAQNKIDSLNLDEFCNCSEHIPSTIAVVGATGSGVST   151
ENSHGLP00000002019_Hgfe/1-1108          TLGYRIFDTCNTVSKALEATLSFVAQNKIDSLNLDEFCNCSEHIPSTIAVVGATGSGVST   179
ENSCLAP00000021334_Clan/1-1114          TLGYRIFDTCNTVSKALEATLSFVAQNKIDSLNLDEFCNCSEHIPSTIAVVGATGSGVST   179
ENSCAPP00000014352_Cape/1-1068          TLGYRIFDTCNTVSKALEATLSFVAQNKIDSLNLDEFCNCSEHIPSTIAVVGATGSGVST   151
ENSCPOP00000011705_Cpor/1-1071          TLGYRIFDTCNTVSKALEATLSFVAQNKIDSLNLDEFCNCSEHIPSTIAVVGATGSGVST   151
ENSSARP00000006151_Sara/1-1085          TLGYRIFDTCNTVSKALEATLSFVAQNKNDALNLDEFCNCSEHIPSTIAVVGATGSGIST   151
ENSLAFP00000018459_Lafr/1-1090          TLGYRIFDTCNTVSKALEATLSFVAQNKIDSLNLDEFCNCSEHIPSTIAVVGATGSGIST   151
ENSDNOP00000011819_Dnov/1-1075          TLGYRIFDTCNTVSKALEATLSFVAQNKIDSLNLDEFCNCSEHIPSTIAVVGATGSGIST   151
ENSSSCP00000041077_Sscr/1-1089          TLGYRIFDTCNTVSKALEATLSFVAQNKIDSLNLDEFCNCSEHIPSTIAVVGATGSGIST   151
ENSTTRP00000001326_Ttru/1-1091          TLGYRIFDTCNTVSKALEATLSFVAQNKIDSLNLDEFCNCSEHIPSTIAVVGATGSGIST   151
ENSBBBP00000002858_Bbbi/1-1068          TLGYRIFDTCNTVSKALEATLSFVAQNKIDSLNLDEFCNCSEHIPSTIAVVGATGSGIST   152
ENSOARP00000021602_Oari/1-1032          TLGYRIFDTCNTVSKALEATLSFVAQNKIDSLNLDEFCNCSEHIPSTIAVVGATGSGIST   152
ENSCHIP00000033385_Chir/1-1085          TLGYRIFDTCNTVSKALEATLSFVAQNKIDSLNLDEFCNCSEHIPSTIAVVGATGSGIST   152
ENSBTAP00000059078_Btau/1-1085          TLGYRIFDTCNTVSKALEATLSFVAQNKIDSLNLDEFCNCSEHIPSTIAVVGATGSGIST   152
ENSBIXP00000038534_Bthy/1-1085          TLGYRIFDTCNTVSKALEATLSFVAQNKIDSLNLDEFCNCSEHIPSTIAVVGATGSGIST   152
ENSBMUP00000019115_Bmut/1-1081          TLGYRIFDTCNTVSKALEATLSFVAQNKIDSLNLDEFCNCSEHIPSTIAVVGATGSGIST   152
ENSBIXP00005030570_Bihy/1-1085          TLGYRIFDTCNTVSKALEATLSFVAQNKIDSLNLDEFCNCSEHIPSTIAVVGATGSGIST   152
ENSMUGP00000006365_Mung/1-1080          TLGYRIFDTCNTVSKALEATLSFVAQNKIDSLNLDEFCNCSEHIPSTIAVVGATGSGVST   151
ENSRNOP00000069629_Rnor/1-1079          TLGYRIFDTCNTVSKALEATLSFVAQNKIDSLNLDEFCNCSEHIPSTIAVVGATGSGVST   151
MGP_PahariEiJ_P0028852_Mpah/1-1003      TLGYRIFDTCNTVSKALEATLSFVAQNKIDSLNLDEFCNCSEHIPSTIAVVGATGSGVST   151
MGP_SPRETEiJ_P0043347_Mspr/1-1079       TLGYRIFDTCNTVSKALEATLSFVAQNKIDSLNLDEFCNCSEHIPSTIAVVGATGSGVST   151
ENSMUSP00000069080_Mmus/1-1079          TLGYRIFDTCNTVSKALEATLSFVAQNKIDSLNLDEFCNCSEHIPSTIAVVGATGSGVST   151
ENSMOCP00000010340_Moch/1-1076          TLGYRIFDTCNTVSKALEATLSFVAQNKIDSLNLDEFCNCSEHIPSTIAVVGATGSGVST   151
ENSPEMP00000029995_Pmba/1-1076          TLGYRIFDTCNTVSKALEATLSFVAQNKIDSLNLDEFCNCSEHIPSTIAVVGATGSGVST   151
ENSCGRP00015024036_Cgpi/1-1081          TLGYRIFDTCNTVSKALEATLSFVAQNKIDSLNLDEFCNCSEHIPSTIAVVGATGSGVST   151
ENSCGRP00001016053_Cgch/1-1081          TLGYRIFDTCNTVSKALEATLSFVAQNKIDSLNLDEFCNCSEHIPSTIAVVGATGSGVST   151
ENSCGRP00000017369_Cgcr/1-1081          TLGYRIFDTCNTVSKALEATLSFVAQNKIDSLNLDEFCNCSEHIPSTIAVVGATGSGVST   151
ENSDORP00000002959_Dord/1-1078          TLGYRIFDTCNTVSKALEATLSFVAQNKIDSLNLDEFCNCSEHIPSTIAVVGATGSGVST   151
ENSTSYP00000002643_Csyr/1-1015          TLGYRIFDTCNTVSKALEATLSFVAQNKIDSLNLDEFCNCSEHIPSTIAVVGATGSGVST   151
ENSMLUP00000008249_Mluc/1-1075          TLGYRIFDTCNTVSKALEATLSFVAQNKIDSLNLDEFCNCSQHIPSTIAVVGATGSGIST   151
ENSECAP00000022285_Ecab/1-1084          TLGYRIFDTCNTVSKALEATLSFVAQNKIDSLNLDEFCNCSEHIPSTIAVVGATGSGVST   151
ENSEASP00005022676_Eaas/1-1094          TLGYRIFDTCNTVSKALEATLSFVAQNKIDSLNLDEFCNCSEHIPSTIAVVGATGSGVST   151
ENSCAFP00000017381_Cfam/1-1075          TLGYRIFDTCNTVSKALEATLSFVAQNKIDSLNLDEFCNCSEHIPSTIAVVGATGSGIST   151
ENSCAFP00020007154_Cldi/1-1075          TLGYRIFDTCNTVSKALEATLSFVAQNKIDSLNLDEFCNCSEHIPSTIAVVGATGSGIST   151
ENSVVUP00000030826_Vvul/1-1075          TLGYRIFDTCNTVSKALEATLSFVAQNKIDSLNLDEFCNCSEHIPSTIAVVGATGSGIST   151
ENSFCAP00000008080_Fcat/1-1081          TLGYRIFDTCNTVSKALEATLSFVAQNKIDSLNLDEFCNCSEHIPSTIAVVGATGSGIST   151
ENSPPRP00000023800_Ppar/1-1081          TLGYRIFDTCNTVSKALEATLSFVAQNKIDSLNLDEFCNCSEHIPSTIAVVGATGSGIST   151
ENSPTIP00000015042_Ptal/1-1081          TLGYRIFDTCNTVSKALEATLSFVAQNKIDSLNLDEFCNCSEHIPSTIAVVGATGSGIST   151
ENSMPUP00000004759_Mpfu/1-1082          TLGYRIFDTCNTVSKALEATLSFVAQNKIDSLNLDEFCNCSEHIPSTIAVVGATGSGIST   151
ENSNVIP00000018564_Nvis/1-1092          TLGYRIFDTCNTVSKALEATLSFVAQNKIDSLNLDEFCNCSEHIPSTIAVVGATGSGIST   151
ENSUMAP00000035085_Umar/1-1071          TLGYRIFDTCNTVSKALEATLSFVAQNKIDSLNLDEFCNCSEHIPSTIAVVGATGSGVST   151
ENSUAMP00000035930_Uame/1-1079          TLGYRIFDTCNTVSKALEATLSFVAQNKIDSLNLDEFCNCSEHIPSTIAVVGATGSGVST   151
ENSAMEP00000015060_Amel/1-1089          TLGYRIFDTCNTVSKALEATLSFVAQNKIDSLNLDEFCNCSEHIPSTIAVVGATGSGVST   151
ENSSDAP00000007437_Sdau/1-1079          TLGYRIFDTCNTVSKALEATLSFVAQNKIDSLNLDEFCNCSEHIPSTIAVVGATGSGVST   151
ENSSTOP00000003945_Itri/1-1070          TLGYRIFDTCNTVSKALEATLSFVAQNKIDSLNLDEFCNCSEHIPSTIAVVGATGSGVST   151
ENSMMMP00000015298_Mmma/1-1083          TLGYRIFDTCNTVSKALEATLSFVAQNKIDSLNLDEFCNCSEHIPSTIAVVGATGSGVST   151
ENSUPAP00010025947_Upar/1-1086          TLGYRIFDTCNTVSKALEATLSFVAQNKIDSLNLDEFCNCSEHIPSTIAVVGATGSGVST   151
ENSSBOP00000036416_Sbbo/1-1078          TLGYRIFDTCNTVSKALEATLSFVAQNKIDSLNLDEFCNCSEHIPSTIAVVGATGSGVST   151
ENSANAP00000028404_Anan/1-1078          TLGYRIFDTCNTVSKALEATLSFVAQNKIDSLNLDEFCNCSEHIPSTIAVVGATGSGVST   151
ENSCCAP00000024327_Ccap/1-1077          TLGYRIFDTCNTVSKALEATLSFVAQNKIDSLNLDEFCNCSEHIPSTIAVVGATGSGVST   151
ENSCJAP00000062854_Cjac/1-1089          TLGYRIFDTCNTVSKALEATLSFVAQNKIDSLNLDEFCNCSEHIPSTIAVVGATGSGVST   151
ENSNLEP00000000336_Nleu/1-1088          TLGYRIFDTCNTVSKALEATLSFVAQNKIDSLNLDEFCNCSEHIPSTIAVVGATGSGVST   151
```

ENSPPYP00000015087_Pabe/1-1078	TLGYRIFDTCNTVSKALEATLSFVAQNKIDSLNLDEFCNCSEHIPSTIAVVGATGSGVST	151
ENSP00000420194_Hsap/1-1088	TLGYRIFDTCNTVSKALEATLSFVAQNKIDSLNLDEFCNCSEHIPSTIAVVGATGSGVST	151
ENSGGOP00000020287_Ggor/1-1078	TLGYRIFDTCNTVSKALEATLSFVAQNKIDSLNLDEFCNCSEHIPSTIAVVGATGSGVST	151
ENSPPAP00000026371_Ppan/1-1078	TLGYRIFDTCNTVSKALEATLSFVAQNKIDSLNLDEFCNCSEHIPSTIAVVGATGSGVST	151
ENSPTRP00000043342_Ptro/1-1078	TLGYRIFDTCNTVSKALEATLSFVAQNKIDSLNLDEFCNCSEHIPSTIAVVGATGSGVST	151
ENSCSAP00000003635_Csab/1-882	--	0
ENSMMUP00000061002_Mmul/1-1078	TLGYRIFDTCNTVSKALEATLSFVAQNKIDSLNLDEFCNCSEHIPSTIAVVGATGSGVST	151
ENSPANP00000004316_Panu/1-1081	TLGYRIFDTCNTVSKALEATLSFVAQNKIDSLNLDEFCNCSEHIPSTIAVVGATGSGVST	144
ENSCATP00000028615_Caty/1-1078	TLGYRIFDTCNTVSKALEATLSFVAQNKIDSLNLDEFCNCSEHIPSTIAVVGATGSGVST	151
ENSMFAP00000042132_Mfas/1-1078	TLGYRIFDTCNTVSKALEATLSFVAQNKIDSLNLDEFCNCSEHIPSTIAVVGATGSGVST	151
ENSMNEP00000024602_Mnem/1-1078	TLGYRIFDTCNTVSKALEATLSFVAQNKIDSLNLDEFCNCSEHIPSTIAVVGATGSGVST	151
ENSMLEP00000008744_Mleu/1-1088	TLGYRIFDTCNTVSKALEATLSFVAQNKIDSLNLDEFCNCSEHIPSTIAVVGATGSGVST	151
ENSTGEP00000008766_Tgel/1-1088	TLGYRIFDTCNTVSKALEATLSFVAQNKIDSLNLDEFCNCSEHIPSTIAVVGATGSGVST	151
ENSCANP00000040262_Capa/1-1088	TLGYRIFDTCNTVSKALEATLSFVAQNKIDSLNLDEFCNCSEHIPSTIAVVGATGSGVST	151
ENSPTEP00000016080_Ptep/1-1088	TLGYRIFDTCNTVSKALEATLSFVAQNKIDSLNLDEFCNCSEHIPSTIAVVGATGSGVST	151
ENSRBIP00000006831_Rbie/1-1078	TLGYRIFDTCNTVSKALEATLSFVAQNKIDSLNLDEFCNCSEHIPSTIAVVGATGSGIST	151
ENSRROP00000007818_Rrox/1-1088	TLGYRIFDTCNTVSKALEATLSFVAQNKIDSLNLDEFCNCSEHIPSTIAVVGATGSGIST	151
ENSOGAP00000002850_Ogar/1-1077	TLGYRIFDTCNTVSKALEATLSFVAQNKIDSLNLDEFCNCSEHIPSTIAVVGATGSGVST	151
ENSMICP00000050654_Mmur/1-1075	TLGYRIFDTCNTVSKALEATLSFVAQNKIDSLNLDEFCNCSEHIPSTIAVVGATGSGIST	151
ENSPCOP00000009624_Pcoq/1-1076	TLGYRIFDTCNTVSKALEATLSFVAQNKIDSLNLDEFCNCSEHIPSTIAVVGATGSGIST	151
ENSPSMP00000007909_Psim/1-1084	TLGYRIFDTCNTVSKALEATLSFVAQNKIDSLNLDEFCNCSEHIPSTIAVVGATGSGIST	151

ENSMEUP00000013310_Neug/1-956	AVANLLGLFYIPXXXXXXXXXXXXXXXXXXXXXXXXXXXXXXXXXXXXXXXXXXXXX--X	147
ENSTBEP00000002487_Tbel/1-914	--------------VSYASSSRLLSNKNQFKSFLRTIPNDEHQATAMADIIEYFRWNWVG	46
ENSPCAP00000000755_Pcap/1-1085	AVANLLGLFYIPQ-VSYASSSRLLSNKNQFKSFLRTIPNDEHQATAMADIVEYFRWNWVG	210
ENSCHOP00000011907_Chof/1-954	AVANLLGLFYIPQXXXXXXXXXXXXXXXXXXXXXXXXXXXXXXXXXXXXXXXXXX-XX	210
ENSVPAP00000001982_Vpac/1-1087	AVANLLGLFYIPQ-VSYASSSRLLSNKNQFKSFLRTIPNDEHQATAMADIIEYFRWNWVG	210
ENSEEUP00000009670_Eeur/1-946	AVANLLGLFYIPQ-V---------------------------------NLIFHFGWPWIG	177
ENSOANP00000015961_Oana/1-1083	AVANLLGLFYIPQ-VSYASSSRLLSNKNQFKSFLRTIPNDEHQATAMADIIEHFRWNWVG	210
ENSOPRP00000000261_Opri/1-1082	AVANLLGLFYIPQ-VSYASSSRLLSNKNQFKSFLRTIPNDEHQATAMADIIEYFRWNWVG	210
ENSJJAP00000004721_Jjac/1-1084	AVANLLGLFYIPQ-VSYASSSRLLSNKNQFKSFLRTIPNDEHQATAMADIIEYFRWNWVG	211
ENSPCIP00000034747_Pcin/1-1018	------------L-LGYASSSRLLSNKNQYKSFLRTIPNDEHQATAMADIIEYFRWNWVG	165
ENSMODP00000022810_Mdom/1-1069	AVANLLGLFYIPQ-VSYASSSRLLSNKNQYKSFLRTIPNDEHQATAMADIIEYFRWNWVG	210
ENSVURP00010007344_Vurs/1-1070	AVANLLGLFYIPQ-VSYASSSRLLSNKNQYKSFLRTIPNDEHQATAMADIIEYFRWNWVG	210
ENSSHAP00000014890_Shar/1-1072	AVANLLGLFYIPQ-VSYASSSRLLSNKNQYKSFLRTIPNDEHQATAMADIIEYFRWNWVG	210
ENSMAUP00000005394_Maur/1-954	AVANLLGLFYIPQ-VSYASSSRLLSNKNQYKSFLRTIPNDEHQATAMADIIEYFRWNWVG	210
ENSOCUP00000025672_Ocun/1-1063	AVANLLGLFYIPQ-VSYASSSRLLSNKNQFKSFLRTIPNDEHQATAMADIIEYFRWNWVG	210
ENSPVAP00000011981_Pvam/1-1085	AVANLLGLFYIPQ-VSYASSSRLLSNKNQFKSFLRTIPNDEHQATAMADIIEYFRWNWVG	210
ENSETEP00000011160_Etel/1-1024	AVANLLGLFYIPQ-VSYALPSRLLSNKNQFKSFLRTIPNDENQATAMADIIEYFRWNWVG	148
ENSFDAP00000010532_Fdam/1-1108	AVANLLGLFYIPQ-VSYASSSRLLSNKNQFKSFLRTIPNDEHQATAIADIIEYFRWNWVG	238
ENSODEP00000013257_Odeg/1-1070	AVANLLGLFYIPQ-VSYASSSRLLSNKNQFKSFLRTIPNDEHQATAMADIIEYFRWNWVG	210
ENSHGLP00000002019_Hgfe/1-1108	AVANLLGLFYIPQ-VSYASSSRLLSNKNQFKSFLRTIPNDEHQATAMADIIEYFRWNWVG	238
ENSCLAP00000021334_Clan/1-1114	AVANLLGLFYIPQ-VSYASSSRLLSNKNQFKSFLRTIPNDEHQATAMADIIEYFRWNWVG	238
ENSCAPP00000014352_Cape/1-1068	AVANLLGLFYIPQ-VSYASSSRLLSNKNQFKSFLRTIPNDEHQATAMADIIEYFRWNWVG	210
ENSCPOP00000011705_Cpor/1-1071	AVANLLGLFYIPQ-VSYASSSRLLSNKNQFKSFLRTIPNDEHQATAMADIIEYFRWNWVG	210
ENSSARP00000006151_Sara/1-1085	AVANLLGLFYIPQ-VSYASSSRLLSNKNQFKSFLRTIPNDEHQATAMADIVEYFRWNWVG	210
ENSLAFP00000018459_Lafr/1-1090	AVANLLGLFYIPQ-VSYASSSRLLSNKNQFKSFLRTIPNDEHQATAMADIIEYFRWNWVG	210
ENSDNOP00000011819_Dnov/1-1075	AVANLLGLFYIPQ-VSYASSSRLLSNKNQFKSFLRTIPNDEHQATAMADIIEYFRWDWVG	210
ENSSSCP00000041077_Sscr/1-1089	AVANLLGLFYIPQ-VSYASSSRLLSNKNQFKSFLRTIPNDEHQATAMADIIEYFRWNWVG	210
ENSTTRP00000001326_Ttru/1-1091	AVANLLGLFYIPQ-VSYASSSRLLSNKNQFKSFLRTIPNDEHQATAMADIIEYFRWNWVG	210
ENSBBBP00000002858_Bbbi/1-1068	AVANLLGLFYIPQ-VSYASSSRLLSNKNQFKSFLRTIPNDEHQATAMADIIEYFRWNWVG	211
ENSOARP00000021602_Oari/1-1032	AVANLLGLFYIPQ-VSYASSSRLLSNKNQFKSFLRTIPNDEHQATAMADIIEYFRWNWVG	211
ENSCHIP00000033385_Chir/1-1085	AVANLLGLFYIPQ-VSYASSSRLLSNKNQFKSFLRTIPNDEHQATAMADIIEYFRWNWVG	211
ENSBTAP00000059078_Btau/1-1085	AVANLLGLFYIPQ-VSYASSSRLLSNKNQFKSFLRTIPNDEHQATAMADIIEYFRWNWVG	211
ENSBIXP00000038534_Bthy/1-1085	AVANLLGLFYIPQ-VSYASSSRLLSNKNQFKSFLRTIPNDEHQATAMADIIEYFRWNWVG	211
ENSBMUP00000019115_Bmut/1-1081	AVANLLGLFYIPQ-VSYASSSRLLSNKNQFKSFLRTIPNDEHQATAMADIIEYFRWNWVG	211
ENSBIXP00005030570_Bihy/1-1085	AVANLLGLFYIPQ-VSYASSSRLLSNKNQFKSFLRTIPNDEHQATAMADIIEYFRWNWVG	211
ENSMUGP00000006365_Mung/1-1080	AVANLLGLFYIPQ-VSYASSSRLLSNKNQFKSFLRTIPNDEHQATAMADIIEYFRWNWVG	210
ENSRNOP00000069629_Rnor/1-1079	AVANLLGLFYIPQ-VSYASSSRLLSNKNQYKSFLRTIPNDEHQATAMADIIEYFRWNWVG	210
MGP_PahariEiJ_P0028852_Mpah/1-1003	AVANLLGLFYIPQ-VSYASSSRLLSNKNQFKSFLRTIPNDEHQATAMADIIEYFRWNWVG	210
MGP_SPRETEiJ_P0043347_Mspr/1-1079	AVANLLGLFYIPQ-VSYASSSRLLSNKNQFKSFLRTIPNDEHQATAMADIIEYFRWNWVG	210
ENSMUSP00000069080_Mmus/1-1079	AVANLLGLFYIPQ-VSYASSSRLLSNKNQFKSFLRTIPNDEHQATAMADIIEYFRWNWVG	210
ENSMOCP00000010340_Moch/1-1076	AVANLLGLFYIPQ-VSYASSSRLLSNKNQYKSFLRTIPNDEHQATAMADIIEYFRWNWVG	210
ENSPEMP00000029995_Pmba/1-1076	AVANLLGLFYIPQ-VSYASSSRLLSNKNQYKSFLRTIPNDEHQATAMADIIEYFRWNWVG	210
ENSCGRP00015024036_Cgpi/1-1081	AVANLLGLFYIPQ-VSYASSSRLLSNKNQYKSFLRTIPNDELQATAMADIIEYFRWNWVG	210
ENSCGRP00001016053_Cgch/1-1081	AVANLLGLFYIPQ-VSYASSSRLLSNKNQYKSFLRTIPNDELQATAMADIIEYFRWNWVG	210
ENSCGRP00000017369_Cgcr/1-1081	AVANLLGLFYIPQ-VSYASSSRLLSNKNQYKSFLRTIPNDELQATAMADIIEYFRWNWVG	210
ENSDORP00000002959_Dord/1-1078	AVANLLGLFYIPQ-VSYASSSRLLSNKNQFKSFLRTIPNDEHQATAMADIVEYFRWNWVG	210
ENSTSYP00000002643_Csyr/1-1015	AVANLLGLFYIPQ-VSYASSSRLLSNKNQFKSFLRTIPNDEHQATAMADIIEYFRWNWVG	210
ENSMLUP00000008249_Mluc/1-1075	AVANLLGLFYIPQ-VSYASSSRLLSNKNQFKSFLRTIPNDEHQATAMADIIEYFRWNWVG	210
ENSECAP00000022285_Ecab/1-1084	AVANLLGLFYIPQ-VSYASSSRLLSNKNQFKSFLRTIPNDEHQATAMADIIEYFRWNWVG	210
ENSEASP00005022676_Eaas/1-1094	AVANLLGLFYIPQ-VSYASSSRLLSNKNQFKSFLRTIPNDEHQATAMADIIEYFRWNWVG	210
ENSCAFP00000017381_Cfam/1-1075	AVANLLGLFYIPQ-VSYASSSRLLSNKNQFKSFLRTIPNDEHQATAMADIIEYFRWNWVG	210
ENSCAFP00020007154_Cldi/1-1075	AVANLLGLFYIPQ-VSYASSSRLLSNKNQFKSFLRTIPNDEHQATAMADIIEYFRWNWVG	210
ENSVVUP00000030826_Vvul/1-1075	AVANLLGLFYIPQ-VSYASSSRLLSNKNQFKSFLRTIPNDEHQATAMADIIEYFRWNWVG	210
ENSFCAP00000008080_Fcat/1-1081	AVANLLGLFYIPQ-VSYASSSRLLSNKNQFKSFLRTIPNDEHQATAMADIIEYFRWNWVG	210
ENSPPRP00000023800_Ppar/1-1081	AVANLLGLFYIPQ-VSYASSSRLLSNKNQFKSFLRTIPNDEHQATAMADIIEYFRWNWVG	210
ENSPTIP00000015042_Ptal/1-1081	AVANLLGLFYIPQ-VSYASSSRLLSNKNQFKSFLRTIPNDEHQATAMADIIEYFRWNWVG	210
ENSMPUP00000004759_Mpfu/1-1082	AVANLLGLFYIPQ-VSYASSSRLLSNKNQFKSFLRTIPNDEHQATAMADIIEYFRWNWVG	210
ENSNVIP00000018564_Nvis/1-1092	AVANLLGLFYIPQ-VSYASSSRLLSNKNQFKSFLRTIPNDEHQATAMADIIEYFRWNWVG	210
ENSUMAP00000035085_Umar/1-1071	AVANLLGLFYIPQ-VSYASSSRLLSNKNQFKSFLRTIPNDEHQATAMADIIEYFRWNWVG	210
ENSUAMP00000035930_Uame/1-1079	AVANLLGLFYIPQ-VSYASSSRLLSNKNQFKSFLRTIPNDEHQATAMADIIEYFRWNWVG	210
ENSAMEP00000015060_Amel/1-1089	AVANLLGLFYIPQ-VSYASSSRLLSNKNQFKSFLRTIPNDEHQATAMADIIEYFRWNWVG	210
ENSSDAP00000007437_Sdau/1-1079	AVANLLGLFYIPQ-VSYASSSRLLSNKNQFKSFLRTIPNDEHQATAMADIIEYFRWNWVG	210
ENSSTOP00000003945_Itri/1-1070	AVANLLGLFYIPQ-VSYASSSRLLSNKNQFKSFLRTIPNDEHQATAMADIIEYFRWNWVG	210
ENSMMMP00000015298_Mmma/1-1083	AVANLLGLFYIPQ-VSYASSSRLLSNKNQFKSFLRTIPNDEHQATAMADIIEYFRWNWVG	210
ENSUPAP00010025947_Upar/1-1086	AVANLLGLFYIPQ-VSYASSSRLLSNKNQFKSFLRTIPNDEHQATAMADIIEYFRWNWVG	210

ENSSBOP00000036416_Sbbo/1-1078	AVANLLGLFYIPQ-VSYASSSRLLSNKNQFKSFLRTIPNDEHQATAMADIIEYFRWNWVG	210
ENSANAP00000028404_Anan/1-1078	AVANLLGLFYIPQ-VSYASSSRLLSNKNQFKSFLRTIPNDEHQATAMADIIEYFRWNWVG	210
ENSCCAP00000024327_Ccap/1-1077	AVANLLGLFYIPQ-VSYASSSRLLSNKNQFKSFLRTIPNDEHQATAMADIIEYFRWNWVG	210
ENSCJAP00000062854_Cjac/1-1089	AVANLLGLFYIPQ-VSYASSSRLLSNKNQFKSFLRTIPNDEHQATAMADIIEYFRWNWVG	210
ENSNLEP00000000336_Nleu/1-1088	AVANLLGLFYIPQ-VSYASSSRLLSNKNQFKSFLRTIPNDEHQATAMADIIEYFRWNWVG	210
ENSPPYP00000015087_Pabe/1-1078	AVANLLGLFYIPQ-VSYASSSRLLSNKNQFKSFLRTIPNDEHQATAMADIIEYFRWNWVG	210
ENSP00000420194_Hsap/1-1088	AVANLLGLFYIPQ-VSYASSSRLLSNKNQFKSFLRTIPNDEHQATAMADIIEYFRWNWVG	210
ENSGGOP00000020287_Ggor/1-1078	AVANLLGLFYIPQ-VSYASSSRLLSNKNQFKSFLRTIPNDEHQATAMADIIEYFRWNWVG	210
ENSPPAP00000026371_Ppan/1-1078	AVANLLGLFYIPQ-VSYASSSRLLSNKNQFKSFLRTIPNDEHQATAMADIIEYFRWNWVG	210
ENSPTRP00000043342_Ptro/1-1078	AVANLLGLFYIPQ-VSYASSSRLLSNKNQFKSFLRTIPNDEHQATAMADIIEYFRWNWVG	210
ENSCSAP00000003635_Csab/1-882	--MADIIEYFRWNWVG	14
ENSMMUP00000061002_Mmul/1-1078	AVANLLGLFYIPQ-VSYASSSRLLSNKNQFKSFLRTIPNDEHQATAMADIIEYFRWNWVG	210
ENSPANP00000004316_Panu/1-1081	AVANLLGLFYIPQ-VSYASSSRLLSNKNQFKSFLRTIPNDEHQATAMADIIEYFRWNWVG	203
ENSCATP00000028615_Caty/1-1078	AVANLLGLFYIPQ-VSYASSSRLLSNKNQFKSFLRTIPNDEHQATAMADIIEYFRWNWVG	210
ENSMFAP00000042132_Mfas/1-1078	AVANLLGLFYIPQ-VSYASSSRLLSNKNQFKSFLRTIPNDEHQATAMADIIEYFRWNWVG	210
ENSMNEP00000024602_Mnem/1-1078	AVANLLGLFYIPQ-VSYASSSRLLSNKNQFKSFLRTIPNDEHQATAMADIIEYFRWNWVG	210
ENSMLEP00000008744_Mleu/1-1088	AVANLLGLFYIPQ-VSYASSSRLLSNKNQFKSFLRTIPNDEHQATAMADIIEYFRWNWVG	210
ENSTGEP00000008766_Tgel/1-1088	AVANLLGLFYIPQ-VSYASSSRLLSNKNQFKSFLRTIPNDEHQATAMADIIEYFRWNWVG	210
ENSCANP00000040262_Capa/1-1088	AVANLLGLFYIPQ-VSYASSSRLLSNKNQFKSFLRTIPNDEHQATAMADIIEYFRWNWVG	210
ENSPTEP00000016080_Ptep/1-1088	AVANLLGLFYIPQ-VSYASSSRLLSNKNQFKSFLRTIPNDEHQATAMADIIEYFRWNWVG	210
ENSRBIP00000006831_Rbie/1-1078	AVANLLGLFYIPQ-VSYASSSRLLSNKNQFKSFLRTIPNDEHQATAMADIIEYFRWNWVG	210
ENSRROP00000007818_Rrox/1-1088	AVANLLGLFYIPQ-VSYASSSRLLSNKNQFKSFLRTIPNDEHQATAMADIIEYFRWNWVG	210
ENSOGAP00000002850_Ogar/1-1077	AVANLLGLFYIPQ-VSYASSSRLLSNKNQFKSFLRTIPNDEHQATAMADIIEYFRWNWVG	210
ENSMICP00000050654_Mmur/1-1075	AVANLLGLFYIPQ-VSYASSSRLLSNKNQFKSFLRTIPNDEHQATAMADIIEYFRWNWVG	210
ENSPCOP00000009624_Pcoq/1-1076	AVANLLGLFYIPQ-VSYASSSRLLSNKNQFKSFLRTIPNDEHQATAMADIIEYFRWNWVG	210
ENSPSMP00000007909_Psim/1-1084	AVANLLGLFYIPQ-VSYASSSRLLSNKNQFKSFLRTIPNDEHQATAMADIIEYFRWNWVG	210

ENSMEUP00000013310_Neug/1-956	XXXXXXXXXXXXXXXXXXXXXXXXXXXXXXXXXXXXXXXXXXXXXXXXXXXXXXXXXXXX	207
ENSTBEP00000002487_Tbel/1-914	TIAADDDYGRPGIEKFREEAEERDICIDFSELISQYSDEEEIQQVVEVIQNSTAKVIVVF	106
ENSPCAP00000000755_Pcap/1-1085	TIAADDDYGRPGIEKFREEAEERDICIDFSELISQYSDEEEIQHVVEVIQNSTAKVIVVF	270
ENSCHOP00000011907_Chof/1-954	XXXXXXXXXXXXXXXXXXXXXXXXXXXXXXXXXXXXXXXXXXXXXXXXXXXXXXXXXXXX	270
ENSVPAP00000001982_Vpac/1-1087	TIAADDDYGRPGIEKFREEAEERDICIDFSELISQYSDEEEIQQVVEVIQNSTAKVIVVF	270
ENSEEUP00000009670_Eeur/1-946	IISSGDDYGK--MHQFIEKIYKKKTCLAFSEVIPKIYSKEKIQKVIDTIEKSTARVIVLY	235
ENSOANP00000015961_Oana/1-1083	TIAADDDYGRPGIEKFREEAEERDICIDFSELISQYSDEVEIQQAVEVIQNSTAKVIVVF	270
ENSOPRP00000000261_Opri/1-1082	TIAADDDYGRPGIEKFREEAEERDICIDFSELISQYSDDKEIQQVVEVIQNSTAKVIVVF	270
ENSJJAP00000004721_Jjac/1-1084	TIAADDDYGRPGIERFREEAEERDICIDFSELISQYSDEEDIRQVVEVIQNSTARVIVVF	271
ENSPCIP00000034747_Pcin/1-1018	TIAADDDYGRPGIEKFREEAEERDICINFSELISQYSDEDEIQHVVEVIQNSTAKVIVVF	225
ENSMODP00000022810_Mdom/1-1069	TIAADDDYGRPGIEKFREEAEERDICINFSELISQYSDEDEIQHVVEVIQNSTAKVIVVF	270
ENSVURP00010007344_Vurs/1-1070	TIAADDDYGRPGIEKFREEAEERDICINFSELISQYSDEDEIQHVVEVIQNSTAKVIVVF	270
ENSSHAP00000014890_Shar/1-1072	TIAADDDYGRPGIEKFREEAEERDICINFSELISQYSDEDEIQHVVEVIQNSTAKVIVVF	270
ENSMAUP00000005394_Maur/1-954	TIAADDDYGRPGIEKFREEAEERDICIDFSELISQYSDEEEIQHVVEVIQNSTAKVIVVF	270
ENSOCUP00000025672_Ocun/1-1063	TIAADDDYGRPGIEKFREEAEERDICIDFSELISQYSDEDEIKQVVEVIQNSTAKVIVVF	270
ENSPVAP00000011981_Pvam/1-1085	TIAADDDYGRPGIEKFREEAEERDICIDFSELISQYSDEEEIQHVVEVIQNSTAKVIVVF	270
ENSETEP00000011160_Etel/1-1024	TIAADDDYGRPGIEKFREEAEERDICIDFSELISQYSQEKDIHQVVEVIQNSTAKVIVVF	208
ENSFDAP00000010532_Fdam/1-1108	TIAADDDYGRPGIEKFREEAEERDICIDFSELISQYSDKKEIQQVVEVIQNSTAKVIVVF	298
ENSODEP00000013257_Odeg/1-1070	TIAADDDYGRPGIEKFREEAEERDICIDFSELISQYSDEEEIQHVVEVIQNSTAKVIVVF	270
ENSHGLP00000002019_Hgfe/1-1108	TIAADDDYGRPGIEKFREEAEERDICIDFSELISQYSEEDEIQQVVEVIQNSKAKVIVVF	298
ENSCLAP00000021334_Clan/1-1114	TIAADDDYGRPGIEKFREEAEERDICIDFSELISQYSDEEEIQHVVEVIQNSTAKVIVVF	298
ENSCAPP00000014352_Cape/1-1068	TIAADDDYGRPGIEKFREEAEERDICIDFSELISQYSDEEEIQHVVEVIQNSTAKVIVVF	270
ENSCPOP00000011705_Cpor/1-1071	TIAADDDYGRPGIEKFREEAEERDICIDFSELISQYSDEEEIQHVVEVIQNSTAKVIVVF	270
ENSSARP00000006151_Sara/1-1085	TIAADDDYGRPGIEKFREEAEERDICIDFSELISQYSDEEEIQQVVEVIQNSTAKVIVVF	270
ENSLAFP00000018459_Lafr/1-1090	TIAADDDYGRPGIEKFREEAEERDICIDFSELISQYSDEEEIQHVVEVIQNSTAKVIVVF	270
ENSDNOP00000011819_Dnov/1-1075	TIAADDDYGRPGIEKFREEAEERDICIDFSELISQYSDEEEIQQVVEVIQNSTAKVIVVF	270
ENSSSCP00000041077_Sscr/1-1089	TIAADDDYGRPGIEKFREEAEERDICIDFSELISQYSDEEEIQQVVEVIQNSTAKVIVVF	270
ENSTTRP00000001326_Ttru/1-1091	TIAADDDYGRPGIEKFREEAEERDICIDFSELISQYSDEEEIQQVVEVIQNSTAKVIVVF	270
ENSBBBP00000002858_Bbbi/1-1068	TIAADDDYGRPGIEKFREEAEERDICIDFSELISQYSDEEKIQQVVEVIQNSTAKVIVVF	271
ENSOARP00000021602_Oari/1-1032	TIAADDDYGRPGIEKFREEAEERDICIDFSELISQYSDEEEIQQVVGVIQNSTAKVIVVF	271
ENSCHIP00000033385_Chir/1-1085	TIAADDDYGRPGIEKFREEAEERDICIDFSELISQYSDEEEIQQVVGVIQNSTAKVIVVF	271
ENSBTAP00000059078_Btau/1-1085	TIAADDDYGRPGIEKFREEAEERDICIDFSELISQYSDEEKIQQVVEVIQNSTAKVIVVF	271
ENSBIXP00000038534_Bthy/1-1085	TIAADDDYGRPGIEKFREEAEERDICIDFSELISQYSDEEKIQQVVEVIQNSTAKVIVVF	271
ENSBMUP00000019115_Bmut/1-1081	TIAADDDYGRPGIEKFREEAEERDICIDFSELISQYSDEEKIQQVVEVIQNSTAKVIVVF	271
ENSBIXP00005030570_Bihy/1-1085	TIAADDDYGRPGIEKFREEAEERDICIDFSELISQYSDEEKIQQVVEVIQNSTAKVIVVF	271
ENSMUGP00000006365_Mung/1-1080	TIAADDDYGRPGIEKFREEAEERDICIDFSELISQYSGEKEIQQVVEVIQNSTVKVIVVF	270
ENSRNOP00000069629_Rnor/1-1079	TIAADDDYGRPGIEKFREEAEERDICIDFSELISQYSDEEEIQQVVEVIQNSTAKVIVVF	270
MGP_PahariEiJ_P0028852_Mpah/1-1003	TIAADDDYGRPGIEKFREEAEERDICIDFSELISQYSDEEEIQQVVEVIQNSTAKVIVVF	270
MGP_SPRETEiJ_P0043347_Mspr/1-1079	TIAADDDYGRPGIEKFREEAEERDICIDFSELISQYSDEEEIQQVVEVIQNSTAKVIVVF	270
ENSMUSP00000069080_Mmus/1-1079	TIAADDDYGRPGIEKFREEAEERDICIDFSELISQYSDEEEIQQVVEVIQNSTAKVIVVF	270
ENSMOCP00000010340_Moch/1-1076	TIAADDDYGRPGIEKFREEAEERDICIDFSELISQYSDEKEIQQVVEVIQNSTAKVIVVF	270
ENSPEMP00000029995_Pmba/1-1076	TIAADDDYGRPGIEKFREEAEERDICIDFSELISQYSDEEEIQQVVEVIQNSTAKVIVVF	270
ENSCGRP00015024036_Cgpi/1-1081	TIAADDDYGRPGIEKFREEAEERDICIDFSELISQYSDKKEIQQVVEVIQNSTAKVIVVF	270
ENSCGRP00001016053_Cgch/1-1081	TIAADDDYGRPGIEKFREEAEERDICIDFSELISQYSDKKEIQQVVEVIQNSTAKVIVVF	270
ENSCGRP00000017369_Cgcr/1-1081	TIAADDDYGRPGIEKFREEAEERDICIDFSELISQYSDKKEIQQVVEVIQNSTAKVIVVF	270
ENSDORP00000002959_Dord/1-1078	TIAADDDYGRPGIEKFREEAEERDICIDFSELISQYSDEEEIRHVVEVIQNSTAKVIVVF	270
ENSTSYP00000002643_Csyr/1-1015	TIAADDDYGRPGIEKFREEAEERDICIDFSELISQYSDEEEIQQVVEVIQNSKAKVIVVF	270
ENSMLUP00000008249_Mluc/1-1075	TIAADDDYGRPGIEKFREEAEERDICIDFSELISQYSDEEEIQQVVEVIQNSTAKVIVVF	270
ENSECAP00000022285_Ecab/1-1084	TIAADDDYGRPGIEKFREEAEERDICIDFSELISQYSDEEEIQHVVEVIQNSTAKVIVVF	270
ENSEASP00005022676_Eaas/1-1094	TIAADDDYGRPGIEKFREEAEERDICIDFSELISQYSDEEEIQHVVEVIQNSTAKVIVVF	270
ENSCAFP00000017381_Cfam/1-1075	TIAADDDYGRPGIEKFREEAEERDICIDFSELISQYSDEEEIQQVVEVIQNSTAKVIVVF	270
ENSCAFP00020007154_Cldi/1-1075	TIAADDDYGRPGIEKFREEAEERDICIDFSELISQYSDEEEIQQVVEVIQNSTAKVIVVF	270
ENSVVUP00000030826_Vvul/1-1075	TIAADDDYGRPGIEKFREEAEERDICIDFSELISQYSDEEEIQQVVEVIQNSTAKVIVVF	270
ENSFCAP00000008080_Fcat/1-1081	TIAADDDYGRPGIEKFREEAEERDICIDFSELISQYSDEEEIQQVVEVIQNSTAKVIVVF	270
ENSPPRP00000023800_Ppar/1-1081	TIAADDDYGRPGIEKFREEAEERDICIDFSELISQYSDEEEIQQVVEVIQNSTAKVIVVF	270
ENSPTIP00000015042_Ptal/1-1081	TIAADDDYGRPGIEKFREEAEERDICIDFSELISQYSDEEEIQQVVEVIQNSTAKVIVVF	270
ENSMPUP00000004759_Mpfu/1-1082	TIAADDDYGRPGIEKFREEAEERDICIDFSELISQYSDEEEIQQVVEVIQNSTAKVIVVF	270
ENSNVIP00000018564_Nvis/1-1092	TIAADDDYGRPGIEKFREEAEERDICIDFSELISQYSDEEEIQQVVEVIQNSTAKVIVVF	270
ENSUMAP00000035085_Umar/1-1071	TIAADDDYGRPGIEKFREEAEERDICIDFSELISQYSDEEEIQQVVEVIQNSTAKVIVVF	270
ENSUAMP00000035930_Uame/1-1079	TIAADDDYGRPGIEKFREEAEERDICIDFSELISQYSDEEEIQQVVEVIQNSTAKVIVVF	270

```
ENSAMEP00000015060_Amel/1-1089         TIAADDDYGRPGIEKFREEAEERDICIDFSELISQYSDEEEIQQVVEVIQNSTAKVIVVF   270
ENSSDAP00000007437_Sdau/1-1079         TIAADDDYGRPGIEKFREEAEERDICIDFSELISQYSDEEEIQQVVEVIQNSTAKVIVVF   270
ENSSTOP00000003945_Itri/1-1070         TIAADDDYGRPGIEKFREEAEERDICIDFSELISQYSDEEEIQQVVEVIQNSTAKVIVVF   270
ENSMMMP00000015298_Mmma/1-1083         TIAADDDYGRPGIEKFREEAEERDICIDFSELISQYSDEEEIQQVVEVIQNSTAKVIVVF   270
ENSUPAP00010025947_Upar/1-1086         TIAADDDYGRPGIEKFREEAEERDICIDFSELISQYSDEEEIQQVVEVIQNSTAKVIVVF   270
ENSSBOP00000036416_Sbbo/1-1078         TIAADDDYGRPGIEKFREEAEERDICIDFSELISQYSDEEEIQQVVEVIQNSTAKVIVVF   270
ENSANAP00000028404_Anan/1-1078         TIAADDDYGRPGIEKFREEAEERDICIDFSELISQYSDEEEIQQVVEVIQNSTAKVIVVF   270
ENSCCAP00000024327_Ccap/1-1077         TIAADDDYGRPGIEKFREEAEERDICIDFSELISQYSDEEEIQQVVEVIQNSTAKVIVVF   270
ENSCJAP00000062854_Cjac/1-1089         TIAADDDYGRPGIEKFREEAEERDICIDFSELISQYSDEEEIQQVVEVIQNSTAKVIVVF   270
ENSNLEP00000000336_Nleu/1-1088         TIAADDDYGRPGIEKFREEAEERDICIDFSELISQYSDEEEIQHVVEVIQNSTAKVIVVF   270
ENSPPYP00000015087_Pabe/1-1078         TIAADDDYGRPGIEKFREEAEERDICIDFSELISQYSDAEEIQHVVEVIQNSTAKVIVVF   270
ENSP00000420194_Hsap/1-1088            TIAADDDYGRPGIEKFREEAEERDICIDFSELISQYSDEEEIQHVVEVIQNSTAKVIVVF   270
ENSGGOP00000020287_Ggor/1-1078         TIAADDDYGRPGIEKFREEAEERDICIDFSELISQYSDEEEIQHVVEVIQNSTAKVIVVF   270
ENSPPAP00000026371_Ppan/1-1078         TIAADDDYGRPGIEKFREEAEERDICIDFSELISQYSDEEEIQHVVEVIQNSTAKVIVVF   270
ENSPTRP00000043342_Ptro/1-1078         TIAADDDYGRPGIEKFREEAEERDICIDFSELISQYSDEEEIQHVVEVIQNSTAKVIVVF   270
ENSCSAP00000003635_Csab/1-882          TIAADDDYGRPGIEKFREEAEERDICIDFSELISQYSDEEEIQHVVEVIQNSTAKVIVVF   74
ENSMMUP00000061002_Mmul/1-1078         TIAADDDYGRPGIEKFREEAEERDICIDFSELISQYSDEEEIQHVVEVIQNSTAKVIVVF   270
ENSPANP00000004316_Panu/1-1081         TIAADDDYGRPGIEKFREEAEERDICIDFSELISQYSDEEEIQHVVEVIQNSTAKVIVVF   263
ENSCATP00000028615_Caty/1-1078         TIAADDDYGRPGIEKFREEAEERDICIDFSELISQYSDEEEIQHVVEVIQNSTAKVIVVF   270
ENSMFAP00000042132_Mfas/1-1078         TIAADDDYGRPGIEKFREEAEERDICIDFSELISQYSDEEEIQHVVEVIQNSTAKVIVVF   270
ENSMNEP00000024602_Mnem/1-1078         TIAADDDYGRPGIEKFREEAEERDICIDFSELISQYSDEEEIQHVVEVIQNSTAKVIVVF   270
ENSMLEP00000008744_Mleu/1-1088         TIAADDDYGRPGIEKFREEAEERDICIDFSELISQYSDEEEIQHVVEVIQNSTAKVIVVF   270
ENSTGEP00000008766_Tgel/1-1088         TIAADDDYGRPGIEKFREEAEERDICIDFSELISQYSDEEEIQHVVEVIQNSTAKVIVVF   270
ENSCANP00000040262_Capa/1-1088         TIAADDDYGRPGIEKFREEAEERDICIDFSELISQYSDEEEIQHVVEVIQNSTAKVIVVF   270
ENSPTEP00000016080_Ptep/1-1088         TIAADDDYGRPGIEKFREEAEERDICIDFSELISQYSDEEEIQHVVEVIQNSTAKVIVVF   270
ENSRBIP00000006831_Rbie/1-1078         TIAADDDYGRPGIEKFREEAEERDICIDFSELISQYSDEEEIQHVVEVIQNSTAKVIVVF   270
ENSRROP00000007818_Rrox/1-1088         TIAADDDYGRPGIEKFREEAEERDICIDFSELISQYSDEEEIQHVVEVIQNSTAKVIVVF   270
ENSOGAP00000002850_Ogar/1-1077         TIAADDDYGRPGIEKFREEAEERDICIDFSELISQYSDEEEIQQVVEVIQNSTAKVIVVF   270
ENSMICP00000050654_Mmur/1-1075         TIAADDDYGRPGIEKFREEAEERDICIDFSELISQYSDEEEIQQVVEVIQNSTAKVIVVF   270
ENSPCOP00000009624_Pcoq/1-1076         TIAADDDYGRPGIEKFREEAEERDICIDFSELISQYSDEEEIQQVVEVIQNSTAKVIVVF   270
ENSPSMP00000007909_Psim/1-1084         TIAADDDYGRPGIEKFREEAEERDICIDFSELISQYSDEEEIQQVVEVIQNSTAKVIVVF   270


ENSMEUP00000013310_Neug/1-956          XXXXXXXXXXXXXXXXXXXXXXXXXSEAWASSSLIAM-EFFHVIGGTIGFALKGGQIPGF   266
ENSTBEP00000002487_Tbel/1-914          SSGPDLEPLIKEIVRRNITGRIWLASEAWASSSLIAMPEYFHVVGGTIGFALKAGQIPGF   166
ENSPCAP00000000755_Pcap/1-1085         SSGPDLEPLIKEIVRRNITGRIWLASEAWASSSLIAMPEYFHVVGGTIGFALKAGQIPGF   330
ENSCHOP00000011907_Chof/1-954          XXXXXXXXXXXXXXXXXXXXXXXXXXXXXPSSSLIAMREYFHVVGG-IGFALKAGQIPGF   329
ENSVPAP00000001982_Vpac/1-1087         SSGPDLEPLIKEIVRRNITGRIWLASEAWASSSLIAMPEYFHVVGGTIGFAL-AGQIPGF   329
ENSEEUP00000009670_Eeur/1-946          ASDLDLGPLVMAIVHHNITGRTWIASEAWITSAALAKPEYFPYFGGTIGFAVPRADIPGL   295
ENSOANP00000015961_Oana/1-1083         SSGPDLEPLIKEIVRRNITGRIWLASEAWASSSLIAMPEFFHVIGGTIGFALKAGQIPGF   330
ENSOPRP00000000261_Opri/1-1082         SSGPDLEPLIKEIVRRNITGRIWLASEAWASSSLIAMPEYFHVVGGTIGFALKAGQIPGF   330
ENSJJAP00000004721_Jjac/1-1084         SSGPDLEPLIKEIVRRNITGKIWLASEAWASSSLIAMPDYFHVVGGTIGFGLKAGQIPGF   331
ENSPCIP00000034747_Pcin/1-1018         SSGPDLEPLIKEIVRRNITGRIWLASEAWAS---------------TIGFALKGGQIPGF   270
ENSMODP00000022810_Mdom/1-1069         SSGPDLEPLIKEIVRRNITGRIWLASEAWASSSLIAMPEFFHVIGGTIGFGLKAGQIPGF   330
ENSVURP00010007344_Vurs/1-1070         SSGPDLEPLIKEIVRRNITGRIWLASEAWASSSLIAMPEFFHVIGGTIGFALKGGQIPGF   330
ENSSHAP00000014890_Shar/1-1072         SSGPDLEPLIKEIVRRNITGRIWLASEAWASSSLIAMPEFFHVIGGTIGFALKGGQIPGF   330
ENSMAUP00000005394_Maur/1-954          SSGPDLEPLIKEIVRRNITGRIWLASEAWASSSLIAMPEYFHVVGGTIGFGLKAGQIPGF   330
ENSOCUP00000025672_Ocun/1-1063         SSGPDLEPLIKEIVRRNITGRIWLASEAWASSSLIAMPEYFHVVGGTIGFALKAGQIPGF   330
ENSPVAP00000011981_Pvam/1-1085         SSGPDLEPLIKEIVRRNITGRIWLASEAWASSSLIAMPEYFHVVGGTIGFALKAGQIPGF   330
ENSETEP00000011160_Etel/1-1024         SSGPDLEPLIKEIVRRNITGRIWLASEAWASSSLIAMPEYFHVVGGTIGFGLKAGQIPGF   268
ENSFDAP00000010532_Fdam/1-1108         SSGPDLEPLIKEIVRHNITGKIWLASEAWASSSLIAMPQYFHVVGGTIGFALKAGQIPGF   358
ENSODEP00000013257_Odeg/1-1070         SSGPDLEPLIKEIVRRNITGKIWLASEAWASSSLIAMPQYFHVVGGTIGFALKAGKIPGF   330
ENSHGLP00000002019_Hgfe/1-1108         SSGPDLEPLIKEIVRRNITGKIWLASEAWASSSLIAMPEYFHVVGGTIGFALKAGKIPGF   358
ENSCLAP00000021334_Clan/1-1114         SSGPDLEPLIKEIVQRNITGRIWLASEAWASSSLIAMPEYFHVVGGTIGFALKAGQIPGF   358
ENSCAPP00000014352_Cape/1-1068         SSGPDLEPLIKEIVRRNITGKIWLASEAWASSSLIAMPEYFHVVGGTIGFALRAGQIPGF   330
ENSCPOP00000011705_Cpor/1-1071         SSGPDLEPLIKEIVRRNITGKIWLASEAWASSSLIAMPEYFHVVGGTIGFALRAGQIPGF   330
ENSSARP00000006151_Sara/1-1085         SSGPDLEPLIKEIVRRNITGRIWLASEAWASSSLIAMPEYFHVVGGTIGFGLKAGQIPGF   330
ENSLAFP00000018459_Lafr/1-1090         SSGPDLEPLIKEIVRRNITGRIWLASEAWASSSLIAMPEYFHVVGGTIGFGLKAGQIPGF   330
ENSDNOP00000011819_Dnov/1-1075         SSGPDLEPLIKEIVRRNITGRIWLASEAWASSSLIAMPEYFHVVGGTIGFALKAGQIPGF   330
ENSSSCP00000041077_Sscr/1-1089         SSGPDLEPLIKEIVRRNITGKIWLASEAWASSSLIAMPEYFHVVGGTIGFALKAGQIPGF   330
ENSTTRP00000001326_Ttru/1-1091         SSGPDLEPLIKEIVRRNITGRIWLASEAWASSSLIAMPEYFHVVGGTIGFALKAGQIPGF   330
ENSBBBP00000002858_Bbbi/1-1068         SSGPDLEPLIKEIVRRNITGRIWLASEAWASSSLIAMPEYFHVVGGTIGFGLKAGQIPGF   331
ENSOARP00000021602_Oari/1-1032         SSGPDLEPLIKEIVRRNITGRIWLASEAWASSSLIAMPEYFHVVGGTIGFGLKAGQIPGF   331
ENSCHIP00000033385_Chir/1-1085         SSGPDLEPLIKEIVRRNITGRIWLASEAWASSSLIAMPEYFHVVGGTIGFGLKAGQIPGF   331
ENSBTAP00000059078_Btau/1-1085         SSGPDLEPLIKEIVRRNITGRIWLASEAWASSSLIAMPEYFHVVGGTIGFGLKAGQIPGF   331
ENSBIXP00000038534_Bthy/1-1085         SSGPDLEPLIKEIVRRNITGRIWLASEAWASSSLIAMPEYFHVVGGTIGFGLKAGQIPGF   331
ENSBMUP00000019115_Bmut/1-1081         SSGPDLEPLIKEIVRRNITGRIWLASEAWASSSLIAMPEYFHVVGGTIGFGLKAGQIPGF   331
ENSBIXP00005030570_Bihy/1-1085         SSGPDLEPLIKEIVRRNITGRIWLASEAWASSSLIAMPEYFHVVGGTIGFGLKAGQIPGF   331
ENSMUGP00000006365_Mung/1-1080         SSGPDLEPLIKEIVRRNITGRIWLASEAWASSSLIAMPEYFHVVGGTIGFGLKAGQIPGF   330
ENSRNOP00000069629_Rnor/1-1079         SSGPDLEPLIKEIVRRNITGRIWLASEAWASSSLIAMPEYFHVVGGTIGFGLKAGQIPGF   330
MGP_PahariEiJ_P0028852_Mpah/1-1003     SSGPDLEPLIKEIVRRNITGRIWLASEAWASSSLIAMPEYFHVVGGTIGFGLKAGQIPGF   330
MGP_SPRETEiJ_P0043347_Mspr/1-1079      SSGPDLEPLIKEIVRRNITGRIWLASEAWASSSLIAMPEYFHVVGGTIGFGLKAGQIPGF   330
ENSMUSP00000069080_Mmus/1-1079         SSGPDLEPLIKEIVRRNITGRIWLASEAWASSSLIAMPEYFHVVGGTIGFGLKAGQIPGF   330
ENSMOCP00000010340_Moch/1-1076         SSGPDLEPLIKEIVRRNITGRIWLASEAWASSSLIAMPEYFHVVGGTIGFGLKAGQIPGF   330
ENSPEMP00000029995_Pmba/1-1076         SSGPDLEPLIKEIVRRNITGRIWLASEAWASSSLIAMPEYFHVVGGTIGFGLKAGQIPGF   330
ENSCGRP00015024036_Cgpi/1-1081         SSGPDLEPLIKEIVRRNITGRIWLASEAWASSSLIAMPEYFHVVGGTIGFGLKAGQIPGF   330
ENSCGRP00001016053_Cgch/1-1081         SSGPDLEPLIKEIVRRNITGRIWLASEAWASSSLIAMPEYFHVVGGTIGFGLKAGQIPGF   330
ENSCGRP00000017369_Cgcr/1-1081         SSGPDLEPLIKEIVRRNITGRIWLASEAWASSSLIAMPEYFHVVGGTIGFGLKAGQIPGF   330
ENSDORP00000002959_Dord/1-1078         SSGPDLEPLIREIVRRNITGRIWLASEAWASSSLIAMPQYFHVVGGTIGFGLKAGQIPGF   330
ENSTSYP00000002643_Csyr/1-1015         SSGPDLEPLIKEIVQRNITGRIWLASEAWASSSLIAMPEYFHVVGGTIGFALKAGQIPGF   330
ENSMLUP00000008249_Mluc/1-1075         SSGPDLEPLIKEIVRRNITGRIWLASEAWASSSLIAMPEYFHVVGGTIGFGLKAGQIPGF   330
ENSECAP00000022285_Ecab/1-1084         SSGPDLEPLIKEIVRRNITGKIWLASEAWASSSLIAMPEYFHVVGGTIGFALRAGQIPGF   330
ENSEASP00005022676_Eaas/1-1094         SSGPDLEPLIKEIVRRNITGKIWLASEAWASSSLIAMPEYFHVVGGTIGFALRAGQIPGF   330
ENSCAFP00000017381_Cfam/1-1075         SSGPDLEPLIKEIVRRNITGRIWLASEAWASSSLIAMPEYFHVVGGTIGFALKAGQIPGF   330
ENSCAFP00020007154_Cldi/1-1075         SSGPDLEPLIKEIVRRNITGRIWLASEAWASSSLIAMPEYFHVVGGTIGFALKAGQIPGF   330
ENSVVUP00000030826_Vvul/1-1075         SSGPDLEPLIKEIVRRNITGRIWLASEAWASSSLIAMPEYFHVVGGTIGFALKAGQIPGF   330
ENSFCAP00000008080_Fcat/1-1081         SSGPDLEPLIKEIVRRNITGRIWLASEAWASSSLIAMPEYFHVVGGTIGFALKAGQIPGF   330
ENSPPRP00000023800_Ppar/1-1081         SSGPDLEPLIKEIVRRNITGRIWLASEAWASSSLIAMPEYFHVVGGTIGFALKAGQIPGF   330
```

```
ENSPTIP00000015042_Ptal/1-1081         SSGPDLEPLIKEIVRRNITGRIWLASEAWASSSLIAMPEYFHVVGGTIGFALKAGQIPGF   330
ENSMPUP00000004759_Mpfu/1-1082         SSGPDLEPLIKEIVRRNITGRIWLASEAWASSSLIAMPEYFHVVGGTIGFALKAGKIPGF   330
ENSNVIP00000018564_Nvis/1-1092         SSGPDLEPLIKEIVRRNITGRIWLASEAWASSSLIAMPEYFHVVGGTIGFALKAGKIPGF   330
ENSUMAP00000035085_Umar/1-1071         SSGPDLEPLIKEIVRRNITGRIWLASEAWASSSLIAMPEYFHVVGGTIGFALKAGQIPGF   330
ENSUAMP00000035930_Uame/1-1079         SSGPDLEPLIKEIVRRNITGRIWLASEAWASSSLIAMPEYFHVVGGTIGFALKAGQIPGF   330
ENSAMEP00000015060_Amel/1-1089         SSGPDLEPLIKEIVRRNITGRIWLASEAWASSSLIAMPEYFHVVGGTIGFALKAGQIPGF   330
ENSSDAP00000007437_Sdau/1-1079         SSGPDLEPLIKEIVRRNITGRIWLASEAWASSSLIAMPEYFHVVGGTIGFALKAGQIPGF   330
ENSSTOP00000003945_Itri/1-1070         SSGPDLEPLIKEIVRRNITGRIWLASEAWASSSLIAMPEYFHVVGGTIGFALKAGQIPGF   330
ENSMMMP00000015298_Mmma/1-1083         SSGPDLEPLIKEIVRRNITGRIWLASEAWASSSLIAMPEYFHVVGGTIGFALKAGQIPGF   330
ENSUPAP00010025947_Upar/1-1086         SSGPDLEPLIKEIVRRNITGRIWLASEAWASSSLIAMPEYFHVVGGTIGFALKAGQIPGF   330
ENSSBOP00000036416_Sbbo/1-1078         SSGPDLEPLIKEIVRRNITGKIWLASEAWASSSLIAMPEYFHVVGGTIGFALKAGQIPGF   330
ENSANAP00000028404_Anan/1-1078         SSGPDLEPLIKEIVRRNITGKIWLASEAWASSSLIAMPEYFHVVGGTIGFALKAGQIPGF   330
ENSCCAP00000024327_Ccap/1-1077         SSGPDLEPLIKEIVRRNITGKIWLASEAWASSSLIAMPEYFHVVGGTIGFALKAGQIPGF   330
ENSCJAP00000062854_Cjac/1-1089         SSGPDLEPLIKEIVRRNITGKIWLASEAWASSSLIAMPEYFHVVGGTIGFALKAGQIPGF   330
ENSNLEP00000000336_Nleu/1-1088         SSGPDLEPLIKEIVRRNITGKIWLASEAWASSSLIAMPQYFHVVGGTIGFALKAGQIPGF   330
ENSPPYP00000015087_Pabe/1-1078         SSGPDLEPLIKEIVRRNITGKIWLASEAWASSSLIAMPQYFHVVGGTIGFALKAGQIPGF   330
ENSP00000420194_Hsap/1-1088            SSGPDLEPLIKEIVRRNITGKIWLASEAWASSSLIAMPQYFHVVGGTIGFALKAGQIPGF   330
ENSGGOP00000020287_Ggor/1-1078         SSGPDLEPLIKEIVRRNITGKIWLASEAWASSSLIAMPQYFHVVGGTIGFALKAGQIPGF   330
ENSPPAP00000026371_Ppan/1-1078         SSGPDLEPLIKEIVRRNITGKIWLASEAWASSSLIAMPQYFHVVGGTIGFALKAGQIPGF   330
ENSPTRP00000043342_Ptro/1-1078         SSGPDLEPLIKEIVRRNITGKIWLASEAWASSSLIAMPQYFHVVGGTIGFALKAGQIPGF   330
ENSCSAP00000003635_Csab/1-882          SSGPDLEPLIKEIVRRNITGKIWLASEAWASSSLIAMPQYFHVVGGTIGFALKAGQIPGF   134
ENSMMUP00000061002_Mmul/1-1078         SSGPDLEPLIKEIVRRNITGKIWLASEAWASSSLIAMPQYFHVVGGTIGFALKAGQIPGF   330
ENSPANP00000004316_Panu/1-1081         SSGPDLEPLIKEIVRRNITGKIWLASEAWASSSLIAMPEYFHVVGGTIGFALKAGQIPGF   323
ENSCATP00000028615_Caty/1-1078         SSGPDLEPLIKEIVRRNITGKIWLASEAWASSSLIAMPEYFHVVGGTIGFALKAGQIPGF   330
ENSMFAP00000042132_Mfas/1-1078         SSGPDLEPLIKEIVRRNITGKIWLASEAWASSSLIAMPEYFHVVGGTIGFALKAGQIPGF   330
ENSMNEP00000024602_Mnem/1-1078         SSGPDLEPLIKEIVRRNITGKIWLASEAWASSSLIAMPEYFHVVGGTIGFALKAGQIPGF   330
ENSMLEP00000008744_Mleu/1-1088         SSGPDLEPLIKEIVRRNITGKIWLASEAWASSSLIAMPEYFHVVGGTIGFALKAGQIPGF   330
ENSTGEP00000008766_Tgel/1-1088         SSGPDLEPLIKEIVRRNITGKIWLASEAWASSSLIAMPEYFHVVGGTIGFALKAGQIPGF   330
ENSCANP00000040262_Capa/1-1088         SSGPDLEPLIKEIVRRNITGKIWLASEAWASSSLIAMPEYFHVVGGTIGFALKAGQIPGF   330
ENSPTEP00000016080_Ptep/1-1088         SSGPDLEPLIKEIVRRNITGKIWLASEAWASSSLIAMPEYFHVVGGTIGFALKAGQIPGF   330
ENSRBIP00000006831_Rbie/1-1078         SSGPDLEPLIKEIVRRNITGKIWLASEAWASSSLIAMPEYFHVVGGTIGFALKAGQIPGF   330
ENSRROP00000007818_Rrox/1-1088         SSGPDLEPLIKEIVRRNITGKIWLASEAWASSSLIAMPEYFHVVGGTIGFALKAGQIPGF   330
ENSOGAP00000002850_Ogar/1-1077         SSGPDLEPLIKQIVRRNITGRIWLASEAWASSSLIAMPEYFHVVGGTIGFALKAGQIPGF   330
ENSMICP00000050654_Mmur/1-1075         SSGPDLEPLIKEIVRRNITGRIWLASEAWASSSLIAMPEYFHVVGGTIGFALKAGQIPGF   330
ENSPCOP00000009624_Pcoq/1-1076         SSGPDLEPLIKEIVRRNITGRIWLASEAWASSSLIAMPEYFHVVGGTIGFALKAGQIPGF   330
ENSPSMP00000007909_Psim/1-1084         SSGPDLEPLIKEIVRRNITGRIWLASEAWASSSLIAMPEYFHVVGGTIGFALKAGQIPGF   330
                                                                     :                ***.:  ..***:

ENSMEUP00000013310_Neug/1-956          REFLQKVHPK-KSAHNGFTKEFWEETFNCHLQEGSK---GSLTLDPFLKNREEGGRSRPN   322
ENSTBEP00000002487_Tbel/1-914          REFLQKVHPR-KSVHNGFAKEFWEETFNCHLQEGAK---GPLPVDTFLRGHEEGGG-RVG   221
ENSPCAP00000000755_Pcap/1-1085         REFLQKVHPR-KSAHNGFAKEFWEETFNCHLQESAK---GPLPVGPVLRDHKEDSL---S   383
ENSCHOP00000011907_Chof/1-954          REFLQKVHPR-KSVHNGFAKEFWEETFNCHLQEGAK---GPLPVDSFLRGHEEGGD-RFS   384
ENSVPAP00000001982_Vpac/1-1087         XXXXXXXXXXXXXXXXXXXXXXXXXXXXXXXXXXXXXXXXXXXXXXXXXXXXXXXX-XXX   388
ENSEEUP00000009670_Eeur/1-946          KEFLYDIHPS-KDPTDVLTIEFWQTAFNCV------------------------------   324
ENSOANP00000015961_Oana/1-1083         RQFLQKVHPR-RSPHNGFTREFWEETFNCHLPEGPK---GPAAPDPFLKVREAGGA-GPG   385
ENSOPRP00000000261_Opri/1-1082         REFLQKVHPR-KSIHNGFAKEFWEETFNCHLQESAK---GPLPMDTFLRGHDEGGS-MVS   385
ENSJJAP00000004721_Jjac/1-1084         REFLQKVHPR-KSVHNGFAKEFWEETFNCHLQEGTK---GPSPVDAFLRGHEKGGN-RLS   386
ENSPCIP00000034747_Pcin/1-1018         REFLQKVHPK-KSVHNGFAKEFWEETFNCHLQEGSK---GSLSLDSFLKNREEGGRSRPN   326
ENSMODP00000022810_Mdom/1-1069         REFLQKVHPK-KSVHNGFAKEFWEETFNCHLQEGSK---GSLSLDPFQKSREEGGRNRPN   386
ENSVURP00010007344_Vurs/1-1070         REFLQKVHPK-KSVHNGFAKEFWEETFNCHLQEGSK---GSLSLDPFLKNREEGGRSRPN   386
ENSSHAP00000014890_Shar/1-1072         REFLQKVHPK-KSVHNGFAKEFWEETFNCHLQEGSK---GSLSFDPFLKSREEGGRNRPN   386
ENSMAUP00000005394_Maur/1-954          REFLQKVHPR-KSVHNGFAKEFWEETFNCHLQEGAK---GPLPVDTFIRSHEEGGN-RLS   385
ENSOCUP00000025672_Ocun/1-1063         REFLQKVHPR-KSVHNGFAKEFWEETFNCHLQESAK---GPLPVDTFLRSHEEGSS-RLS   385
ENSPVAP00000011981_Pvam/1-1085         REFLQKVHPR-KSVHNGFAKEFWEETFNCHLQEGAK---GPLPMDTFLRGQEEGGS-RIS   385
ENSETEP00000011160_Etel/1-1024         REFLQKVHPR-KSVHNGFAKEFWEETFNCHLQEGAK---GPSPVDSLLRDQKGEVS-KSS   323
ENSFDAP00000010532_Fdam/1-1108         REFLQKVHPR-KSVHNGFAKEFWEETFNCHLQEGTK---GPLPTDTFLRGHEKGGN-KLS   413
ENSODEP00000013257_Odeg/1-1070         REFLQKVHPR-KSVHNGFAKEFWEETFNCHLQEGAK---GPLPMDTFLRGHEEVGI-KFS   385
ENSHGLP00000002019_Hgfe/1-1108         REFLQKVHPR-KSVHNGFVKEFWEETFNCHLQEGAK---GPLPMDTFLRGHEEGNS-KLS   413
ENSCLAP00000021334_Clan/1-1114         REFLQKVHPR-KSAHNGFAKEFWEETFNCHLQEGAK---GPLPMDTFLRGHEEGGI-KLS   413
ENSCAPP00000014352_Cape/1-1068         REFLQKVHPR-KSAHNGFAKEFWEETFNCHLQEGAK---GPLPMDTFLKGHEEGGT-KLS   385
ENSCPOP00000011705_Cpor/1-1071         REFLQKVHPR-KSAHNGFAKEFWEETFNCHLQEGAK---GPLPMDTFLKGHEEGGT-KLS   385
ENSSARP00000006151_Sara/1-1085         REFLQKVHPR-KSH-N-GLKEFREETFNCHLQEGAK---GPFPMETFLKGNEEGVS-RIS   383
ENSLAFP00000018459_Lafr/1-1090         REFLQKVHPR-KSVHNGFAKEFWEETFNCHLQENAK---GPLPVDPLLRDHKEGGS-RFS   385
ENSDNOP00000011819_Dnov/1-1075         REFLQKVHPR-KSVHNGFAKEFWEETFNCHLQEGTK---GPLPVNTFLRGHEEGGG-RLG   385
ENSSSCP00000041077_Sscr/1-1089         REFLQKVHPS-KSVHNGFAKEFWEETFNCHLQEGAK---GPLTTDTFLRGHEEGGG-RIS   385
ENSTTRP00000001326_Ttru/1-1091         REFLQKVHPR-KSVHNGFAKEFWEETFNCHLQEGAK---GPLPMDTFLRGHEEGGG-RIS   385
ENSBBBP00000002858_Bbbi/1-1068         REFLQKVHPR-KSVHNGFAKEFWEETFNCHLQEGAK---GPLPVDTFLRGHEEGGA-RLS   386
ENSOARP00000021602_Oari/1-1032         REFLQKVHPR-KSVHNGFAKEFWEETFNCHLQEGAK---GPLPVDTFLRGHEEGGA-RIS   386
ENSCHIP00000033385_Chir/1-1085         REFLQKVHPR-KSVHNGFAKEFWEETFNCHLQEGAK---GPLPVDTFLRGHEEGGA-RIS   386
ENSBTAP00000059078_Btau/1-1085         REFLQKVHPR-KSVHNGFAKEFWEETFNCHLQEGAK---GPLPVDTFLRGHEEGGA-RLS   386
ENSBIXP00000038534_Bthy/1-1085         REFLQKVHPR-KSVHNGFAKEFWEETFNCHLQEGAK---GPLPVDTFLRGHEEGGA-RLS   386
ENSBMUP00000019115_Bmut/1-1081         REFLQKVHPR-KSVHNGFAKEFWEETFNCHLQEGAK---GPLPVDTFLRGHEEGGV-RLS   386
ENSBIXP00005030570_Bihy/1-1085         REFLQKVHPR-KSVHNGFAKEFWEETFNCHLQEGAK---GPLPVDTFLRGHEEGGA-RLS   386
ENSMUGP00000006365_Mung/1-1080         REFLQKVHPR-KSAHNGFAKEFWEETFNCHLQEGTK---GPLPVDSFVRDREEAGN-RLS   385
ENSRNOP00000069629_Rnor/1-1079         REFLQKVHPR-KSVHNGFAKEFWEETFNCHLQEGAK---GPLPVDTFVRSHEEGGN-RLL   385
MGP_PahariEiJ_P0028852_Mpah/1-1003     REFLQKVHPR-KSVHNGFAKEFWEETFNCHLQDGAK---GPLPVDTFVRSHEEGGN-RLL   385
MGP_SPRETEiJ_P0043347_Mspr/1-1079      REFLQKVHPR-KSVHNGFAKEFWEETFNCHLQDGAK---GPLPVDTFVRSHEEGGN-RLL   385
ENSMUSP00000069080_Mmus/1-1079         REFLQKVHPR-KSVHNGFAKEFWEETFNCHLQDGAK---GPLPVDTFVRSHEEGGN-RLL   385
ENSMOCP00000010340_Moch/1-1076         REFLQKVHPR-KSVHNGFAKEFWEETFNCHLQEGAK---GPLPVDTFLRSHEEGGS-RLS   385
ENSPEMP00000029995_Pmba/1-1076         REFLQKVHPR-KSVHNGFAKEFWEETFNCHLQEGAK---GPLPVDTFIRSHEEGGN-RLS   385
ENSCGRP00015024036_Cgpi/1-1081         REFLQKVHPR-KSVHNGFAKEFWEETFNCHLQEGAK---GPLPADTFIRSHEEGGN-RLS   385
ENSCGRP00001016053_Cgch/1-1081         REFLQKVHPR-KSVHNGFAKEFWEETFNCHLQEGAK---GPLPADTFIRSHEEGGN-RLS   385
ENSCGRP00000017369_Cgcr/1-1081         REFLQKVHPR-KSVHNGFAKEFWEETFNCHLQEGAK---GPLPADTFIRSHEEGGN-RLS   385
ENSDORP00000002959_Dord/1-1078         REFLQKVHPR-KSVHNGFAKEFWEETFNCHLQEGVK---GPVPVDSLLRGHEESGG-RVS   385
ENSTSYP00000002643_Csyr/1-1015         REFLQKVHPR-KSVHNGFAKEFWEETFNCHLQEGGK---GPLPADTFLRGHEESGG-RFS   385
ENSMLUP00000008249_Mluc/1-1075         REFLQKVHPR-K-SHNGFAKEFWEETFNCHLQEGAK---GPLPMDTFLRGQEEGGG-RIS   384
ENSECAP00000022285_Ecab/1-1084         REFLQKVHPK-KSAHNGFAKEFWEETFNCHLQEGAK---GPLPMDTFLRGHEEAGG-RLS   385
ENSEASP00005022676_Eaas/1-1094         REFLQKVHPK-KSAHNGFAKEFWEETFNCHLQEGAK---GPLPMDTFLRGHEEAGG-RLS   385
```

ENSCAFP00000017381_Cfam/1-1075	REFLQKVHPR-KSVHNGFAKEFWEETFNCHLQEGAK---GPLSMDTFLRGHEEGGG-RIS	385
ENSCAFP00020007154_Cldi/1-1075	REFLQKVHPR-KSVHNGFAKEFWEETFNCHLQEGAK---GPLSMDTFLRGHEEGGG-RIS	385
ENSVVUP00000030826_Vvul/1-1075	REFLQKVHPR-KSVHNGFAKEFWEETFNCHLQEGAK---GPLSMDTFLRGHEEGGG-RIS	385
ENSFCAP00000008080_Fcat/1-1081	REFLQKVHPR-KSVHNGFAKEFWEETFNCHLQEGAK---GPLALDTFLRGHEEGGG-RIS	385
ENSPPRP00000023800_Ppar/1-1081	REFLQKVHPR-KSVHNGFAKEFWEETFNCHLQEGAK---GPLALDTFLRGHEEGGG-RIS	385
ENSPTIP00000015042_Ptal/1-1081	REFLQKVHPR-KSVHNGFAKEFWEETFNCHLQEGAK---GPLALDTFLRGHEEGGG-RIS	385
ENSMPUP00000004759_Mpfu/1-1082	REFLQKVHPR-KSVHNGFAKEFWEETFNCHLQEGAK---GALSMDTFMRGHEEGGG-RIS	385
ENSNVIP00000018564_Nvis/1-1092	REFLQKVHPR-KSVHNGFAKEFWEETFNCHLQEGAK---GTLSMDTFMRGHEEGGG-RIS	385
ENSUMAP00000035085_Umar/1-1071	REFLQKVHPR-KSVHNGFAKEFWEETFNCHLQEGAK---GPLSMDTFLRGHEEGGG-RIS	385
ENSUAMP00000035930_Uame/1-1079	REFLQKVHPR-KSVHNGFAKEFWEETFNCHLQEGAK---GPLSMDTFLRGHEEGGG-RIS	385
ENSAMEP00000015060_Amel/1-1089	REFLQKVHPR-KSVHNGFAKEFWEETFNCHLQEGPK---GPLSMDTFLRGHEEGGG-RIS	385
ENSSDAP00000007437_Sdau/1-1079	REFLQKVHPR-KSVHNGFAKEFWEETFNCHLQEGAK---GPLPMDTFLRGHEEGGS-RLS	385
ENSSTOP00000003945_Itri/1-1070	REFLQKVHPR-KSVHNGFAKEFWEETFNCHLQEGAK---GPLPMDTFLRSHEEGGS-RLS	385
ENSMMMP00000015298_Mmma/1-1083	REFLQKVHPR-KSVHNGFAKEFWEETFNCHLQEGAK---GPLPVDTFLRGHEEGGS-RLS	385
ENSUPAP00010025947_Upar/1-1086	REFLQKVHPR-KSVHNGFAKEFWEETFNCHLQEGAK---GPLPMDTFLRGHEEGGS-RLS	385
ENSSBOP00000036416_Sbbo/1-1078	REFLKKVHPR-KSVHNGFAKEFWEETFNCHLQEGVK---GPLPVDTFLRGHEESGG-RFS	385
ENSANAP00000028404_Anan/1-1078	REFLKKVHPR-KSVHNGFAKEFWEETFNCHLQEGAK---GPLPVDTFLRGHEESGS-RFS	385
ENSCCAP00000024327_Ccap/1-1077	REFLKKVHPR-KSVHNGFAKEFWEETFNCHLQEGAK---GPLPMDTFLRGHEESGG-RFS	385
ENSCJAP00000062854_Cjac/1-1089	REFLKKVHPR-KSVHNGFAKEFWEETFNCHLQEGAK---GPLPVDNFLRGHEESGG-RFS	385
ENSNLEP00000000336_Nleu/1-1088	REFLKKVHPR-KSVHNGFAKEFWEETFNCHLQEGAK---GPLPVDTFLRGHEESGG-RFS	385
ENSPPYP00000015087_Pabe/1-1078	REFLKKVHPR-KSVHNGFAKEFWEETFNCHLQEGAK---GLLPVDTFLRGHEESGG-KFS	385
ENSP00000420194_Hsap/1-1088	REFLKKVHPR-KSVHNGFAKEFWEETFNCHLQEGAK---GPLPVDTFLRGHEESGD-RFS	385
ENSGGOP00000020287_Ggor/1-1078	REFLKKVHPR-KSVHNGFAKEFWEETFNCHLQEGAK---GPLPVDTFLRGHEESGD-RFS	385
ENSPPAP00000026371_Ppan/1-1078	REFLKKVHPR-KSVHNGFAKEFWEETFNCHLQEGAK---GPLPVDTFLRGHEESGD-RFS	385
ENSPTRP00000043342_Ptro/1-1078	REFLKKVHPR-KSVHNGFAKEFWEETFNCHLQEGAK---GPLPVDTFLRGHEESAD-RFS	385
ENSCSAP00000003635_Csab/1-882	REFLKKVHPR-KSVHNGFAKEFWEETFNCHLQEGAK---GPLPVDTFLRGHEESGG-RFS	189
ENSMMUP00000061002_Mmul/1-1078	REFLKKVHPR-KSVHNGFAKEFWEETFNCHLQEGAK---GPLPVDTFLRGHEESGG-RFS	385
ENSPANP00000004316_Panu/1-1081	REFLKKVHPR-KSVHNGFAKEFWEETFNCHLQEGAK---GPLPVDTFLRGHEESGG-RFS	378
ENSCATP00000028615_Caty/1-1078	REFLKKVHPR-KSVHNGFAKEFWEETFNCHLQEGAK---GPLPVDTFLRGHEESGG-RFS	385
ENSMFAP00000042132_Mfas/1-1078	REFLKKVHPR-KSVHNGFAKEFWEETFNCHLQEGAK---GPLPVDTFLRGHEESGG-RFS	385
ENSMNEP00000024602_Mnem/1-1078	REFLKKVHPR-KSVHNGFAKEFWEETFNCHLQEGAK---GPLPVDTFLRGHEESGG-RFS	385
ENSMLEP00000008744_Mleu/1-1088	REFLKKVHPR-KSVHNGFAKEFWEETFNCHLQEGAK---GPLPVDTFLRGHEESGG-RFS	385
ENSTGEP00000008766_Tgel/1-1088	REFLKKVHPR-KSVHNGFAKEFWEETFNCHLQEGAK---GPLPVDTFLRGHEESGG-RFS	385
ENSCANP00000040262_Capa/1-1088	REFLKKVHPR-KSVHNGFAKEFWEETFNCHLQEGVK---GPLPVDTFLRGHEESGG-RFS	385
ENSPTEP00000016080_Ptep/1-1088	REFLKKVHPR-KSVHNGFAKEFWEETFNCHLQEGVK---GPLPVDTFLRGHEESGG-RFS	385
ENSRBIP00000006831_Rbie/1-1078	REFLKKVHPR-KSVHNGFAKEFWEETFNCHLQEGVK---GPLPVDTFLRGHEESGG-RFS	385
ENSRROP00000007818_Rrox/1-1088	REFLKKVHPR-KSVHNGFAKEFWEETFNCHLQEGVK---GPLPVDTFLRGHEESGG-RFS	385
ENSOGAP00000002850_Ogar/1-1077	REFLKKVHPR-KSVHNGFAKEFWEETFNCHLQEGTK---GPLSTDTFLRGQEDSGN-RFS	385
ENSMICP00000050654_Mmur/1-1075	REFLEKVHPR-KSVHNGFAKEFWEETFNCHLQEGAK---GPLPTDTFLRGQEDSGG-RFS	385
ENSPCOP00000009624_Pcoq/1-1076	REFLKKVHPK-KSVHNGFAKEFWEETFNCHLQEGAK---GPLPTDTFLRGHEDSGG-RFS	385
ENSPSMP00000007909_Psim/1-1084	REFLKKVHPR-KSVHNGFAKEFWEETFNCHLQEGAK---GLLPTDTFLRGQEDSGG-RFS	385

ENSMEUP00000013310_Neug/1-956	NTSTTFRPLCTGDENITSVETPYMDYTHLRISYNVYLAVYSIAHALQD-IYTCSPGKGLF	381
ENSTBEP00000002487_Tbel/1-914	NSSTAFRPLCTGDENISSVETPYMDYTHLRISYNVYLAVYSIAHALQD-IYTCLPGRGLF	280
ENSPCAP00000000755_Pcap/1-1085	NSSTAFRPLCTGNENISSVETPYMDYTHLRISYNVYLAVYSIAHALXXXXXXXXXXXXXX	443
ENSCHOP00000011907_Chof/1-954	NSSTALRPLCTGDENISSVETPYMDYTHLRISYNVYLAVYSIAHALQD-IYTCLPGRGLF	443
ENSVPAP00000001982_Vpac/1-1087	XXXXXXXXXXXXXXXXXXXXXXXXXXX-----XXXXXXXXXXXXXXXXXXXXXXXXXXXX	443
ENSEEUP00000009670_Eeur/1-946	--	324
ENSOANP00000015961_Oana/1-1083	NGSASFRPPCTGRENLTGVETPYLDYTHLRISYNVYLAVYSIAHALQN-IFTCTPGHGLF	444
ENSOPRP00000000261_Opri/1-1082	NSSTAFRPLCTGAENISSVETPYMDYTHLRISYNVYLAVYSIAHALQD-IYTCLPGRGLF	444
ENSJJAP00000004721_Jjac/1-1084	NSSTTFRPLCTGDENISSVETPYMDYTHLRISYNVYLAVYSIAHALQD-IYTCLPGRGLF	445
ENSPCIP00000034747_Pcin/1-1018	NTTTTFRPLCTGDENITSVETPYMDYTHLRISYNVYLAVYSIAHALQD-IYTCSPGKGLF	385
ENSMODP00000022810_Mdom/1-1069	NTTTTFRPLCTGDENITSVETPYMDYTHLRISYNVYLAVYSIAHALQD-IYTCSPGKGLF	445
ENSVURP00010007344_Vurs/1-1070	NMTTTFRPLCTGDENITSVETPYMDYTHLRISYNVYLAVYSIAHALQD-IYTCSPGKGLF	445
ENSSHAP00000014890_Shar/1-1072	NTTTTFRPLCTGDENITSVETPYMDYTHLRISYNVYLAVYSIAHALQD-IYTCSPGKGLF	445
ENSMAUP00000005394_Maur/1-954	NSSVAFRPLCTGDENISSVETPYMDYEHLRISYNVYLAVYSIAHALQD-IYTCLPGRGLF	444
ENSOCUP00000025672_Ocun/1-1063	NSSTAFRPLCTGAENISSVETPYMDYTHLRISYNVYLAVYSIAHALQD-IYTCLPGRGLF	444
ENSPVAP00000011981_Pvam/1-1085	NSSAAFRPLCTGDENISSVETPYMDYTHLRISYNVYLAVYSIAHALQD-IYTCLPGRGLF	444
ENSETEP00000011160_Etel/1-1024	NSSTTFRPLCTGDENISSVETPYMDYTHLRISYNVYLAVYSIAHALQD-IYTCLPGRGLF	382
ENSFDAP00000010532_Fdam/1-1108	NSSTAFRPLCTGEENISSVETPYMDYTHLRISYNVYLAVYSIAQALQD-IYTCVPGRGLF	472
ENSODEP00000013257_Odeg/1-1070	NSSTAFRPLCTGEENISSVETPYMDYTHLRISYNVYLAVYSIAHALQD-IYTCLPGRGLF	444
ENSHGLP00000002019_Hgfe/1-1108	NSSTAFRPLCTGEENISSVETPYMDYTHLRISYNVYLAVYSIAHALQD-IYTCLPGRGLF	472
ENSCLAP00000021334_Clan/1-1114	NSSTAFRPLCTGEENISSVETPYMDYTHLRISYNVYLAVYSIAHALQD-IYTCLPGRGLF	472
ENSCAPP00000014352_Cape/1-1068	NSSTAFRPLCTGKENISSVETPYMDYTHLRISYNVYLAVYSIAHALQD-IYTCLPGRGLF	444
ENSCPOP00000011705_Cpor/1-1071	NSSTAFRPLCTGKENISSVETPYMDYTHLRISYNVYLAVYSIAHALQD-IYTCLPGRGLF	444
ENSSARP00000006151_Sara/1-1085	NSSTAFRPLCTGDENISSVETPYMDYTHLRISYNVYLAVYSIAHALQD-IYTCLPGRGLF	442
ENSLAFP00000018459_Lafr/1-1090	NSSTAFRPLCTGDENISSVETPYIDYTHLRISYNVYLAVYSIAHALQD-IYTCLPGKGLF	444
ENSDNOP00000011819_Dnov/1-1075	NSSTAFRPLCTGDENISSVETPYMDYTHLRISYNVYLAVYSIAHALQD-IYTCLPGRGLF	444
ENSSSCP00000041077_Sscr/1-1089	NSSTAFRPLCTGDENISSVETPYMDYTHLRISYNVYLAVYSIAHALQD-IYTCIPGRGLF	444
ENSTTRP00000001326_Ttru/1-1091	NSSTAFRPLCTGDENISSVETPYMDYTHLRISYNVYLAVYSIAHALQD-IYTCIPGRGLF	444
ENSBBBP00000002858_Bbbi/1-1068	NSSTAFRPLCTGEENISSVETPYMDYTHLRISYNVYLAVYSIAHALQD-IYTCIPGRGLF	445
ENSOARP00000021602_Oari/1-1032	NSSTAFRPLCTGEENISSVETPYMDYTHLRISYNVYLAVYSIAHALQD-IYTCIPGRGLF	445
ENSCHIP00000033385_Chir/1-1085	NSSTAFRPLCTGEENISSVETPYMDYTHLRISYNVYLAVYSIAHALQD-IYTCIPGRGLF	445
ENSBTAP00000059078_Btau/1-1085	NSPTAFRPLCTGEENISSVETPYMDYTHLRISYNVYLAVYSIAHALQD-IYTCIPGRGLF	445
ENSBIXP00000038534_Bthy/1-1085	NSPTAFRPLCTGEENISSVETPYMDYTHLRISYNVYLAVYSIAHALQD-IYTCIPGRGLF	445
ENSBMUP00000019115_Bmut/1-1081	NSSTAFRPLCTGEENISSVETPYMDYTHLRISYNVYLAVYSIAHALQD-IYTCIPGRGLF	445
ENSBIXP00005030570_Bihy/1-1085	NSSSAFRPLCTGEENISSVETPYMDYTHLRISYNVYLAVYSIAHALQD-IYTCIPGRGLF	445
ENSMUGP00000006365_Mung/1-1080	NSSTAFRPLCTGEENLSSVETPYMDYEHLRISYNVYLAVYSIAHALQE-IYTCLPGRGLF	444
ENSRNOP00000069629_Rnor/1-1079	NSSTAFRPLCTGDENINSVETPYMDYEHLRISYNVYLAVYSIAHALQD-IYTCLPGRGLF	444
MGP_PahariEiJ_P0028852_Mpah/1-1003	NSSTAFRPLCTGDENINSVETPYMDYEHLRISYNVYLAVYSIAHALQD-IYTCLPGRGLF	444
MGP_SPRETEiJ_P0043347_Mspr/1-1079	NSSTAFRPLCTGDENINSVETPYMDYEHLRISYNVYLAVYSIAHALQD-IYTCLPGRGLF	444
ENSMUSP00000069080_Mmus/1-1079	NSSTAFRPLCTGDENINSVETPYMDYEHLRISYNVYLAVYSIAHALQD-IYTCLPGRGLF	444
ENSMOCP00000010340_Moch/1-1076	NSSTAFRPLCTGDENISSVETPYMDYEHLRISYNVYLAVYSIAHALQD-IYTCLPGRGLF	444
ENSPEMP00000029995_Pmba/1-1076	NSSTAFRPPCTGDENISSVETPYMDYEHLRISYNVYLAVYSIAHALQD-IYTCLPGRGLF	444
ENSCGRP00015024036_Cgpi/1-1081	NSSTAFRPLCTGDENISSVETPYMDYEHLRISYNVYLAVYSIAHALQD-IYTCLPGRGLF	444
ENSCGRP00001016053_Cgch/1-1081	NSSTAFRPLCTGDENISSVETPYMDYEHLRISYNVYLAVYSIAHALQD-IYTCLPGRGLF	444
ENSCGRP00000017369_Cgcr/1-1081	NSSTAFRPLCTGDENISSVETPYMDYEHLRISYNVYLAVYSIAHALQD-IYTCLPGRGLF	444

```
ENSDORP00000002959_Dord/1-1078          NSSTAFRPLCTGDENISSVETPYIDYTHLRISYNVYLAVYSIAHALQD-IYTCLPGRGLF   444
ENSTSYP00000002643_Csyr/1-1015          NSSTTFRPLCTGDENISSVETPYMDYTHLRISYNVYLAVYSIAHALQD-IFTCLPGKGLF   444
ENSMLUP00000008249_Mluc/1-1075          NSSTVFRPLCTGDENISSVETPYMDYTHLRISYNVYLAVYSIAHALQD-IYTCLPGRGLF   443
ENSECAP00000022285_Ecab/1-1084          NSSTAFRPLCTGDENISSVETPYMDYTHLRISYNVYLAVYSIAHALQD-IYTCSPGRGLF   444
ENSEASP00005022676_Eaas/1-1094          NSSTAFRPLCTGDENISSVETPYMDYTHLRISYNVYLAVYSIAHALQD-IYTCSPGRGLF   444
ENSCAFP00000017381_Cfam/1-1075          NSSTAFRPLCTGDENISSVETPYMDYTHLRISYNVYLAVYSIAHALQD-IYTCLPGRGLF   444
ENSCAFP00020007154_Cldi/1-1075          NSSTAFRPLCTGDENISSVETPYMDYTHLRISYNVYLAVYSIAHALQD-IYTCLPGRGLF   444
ENSVVUP00000030826_Vvul/1-1075          NSSTAFRPLCTGDENISSVETPYMDYTHLRISYNVYLAVYSIAHALQD-IYTCLPGRGLF   444
ENSFCAP00000008080_Fcat/1-1081          NSSTALRPLCTGDENISSVETPYMDYTHLRISYNVYLAVYSIAHALQD-IYTCLPGRGLF   444
ENSPPRP00000023800_Ppar/1-1081          NSSTALRPLCTGDENISSVETPYMDYTHLRISYNVYLAVYSIAHALQD-IYTCLPGRGLF   444
ENSPTIP00000015042_Ptal/1-1081          NSSTALRPLCTGDENISSVETPYMDYTHLRISYNVYLAVYSIAHALQD-IYTCLPGRGLF   444
ENSMPUP00000004759_Mpfu/1-1082          NSSTAFRPLCTGDENISSVETPYMDYTHLRISYNVYLAVYSIAHALQD-IYTCLPGRGLF   444
ENSNVIP00000018564_Nvis/1-1092          NSSTAFRPLCTGDENISSVETPYMDYTHLRISYNVYLAVYSIAHALQD-IYTCLPGRGLF   444
ENSUMAP00000035085_Umar/1-1071          NSSTAFRPLCTGDENISSVETPYMDYTHLRISYNVYLAVYSIAHALQD-IYTCLPGRGLF   444
ENSUAMP00000035930_Uame/1-1079          NSSTAFRPLCTGDENISSVETPYMDYTHLRISYNVYLAVYSIAHALQD-IYTCLPGRGLF   444
ENSAMEP00000015060_Amel/1-1089          NSSTAFRPLCTGDENISSVETPYMDFTHLRISYNVYLAVYSIAHALQD-IYTCLPGRGLF   444
ENSSDAP00000007437_Sdau/1-1079          NSSTAFRPLCTGDENISSVETPYMDYTHLRISYNVYLAVYSIAHALQD-IYTCLPGRGLF   444
ENSSTOP00000003945_Itri/1-1070          NSSTAFRPLCTGDENISSVETPYMDYTHLRISYNVYLAVYSIAHALQD-IYTCLPGRGLF   444
ENSMMMP00000015298_Mmma/1-1083          NSSTAFRPLCTGDENISSVETPYMDYTHLRISYNVYLAVYSIAHALQD-IYTCLPGRGLF   444
ENSUPAP00010025947_Upar/1-1086          NSSTAFRPLCTGDENISSVETPYMDYTHLRISYNVYLAVYSIAHALQD-IYTCLPGRGLF   444
ENSSBOP00000036416_Sbbo/1-1078          NSSTAFRPLCTGDENISSVETPYIDYTHLRISYNVYLAVYSIAHALQD-IYTCSPGRGLF   444
ENSANAP00000028404_Anan/1-1078          NSSSVFRPLCTGDENISSVETPYIDYTHLRISYNVYLAVYSIAHALQD-IYTCSPGRGLF   444
ENSCCAP00000024327_Ccap/1-1077          NSSTAFRPLCTGDENISSVETPYIDYTHLRISYNVYLAVYSIAHALQD-IYTCSPGRGLF   444
ENSCJAP00000062854_Cjac/1-1089          NSSTAFRPLCTGDENISSVETPYIDYTHLRISYNVYLAVYSIAHALQD-IYTCSPGRGLF   444
ENSNLEP00000000336_Nleu/1-1088          NSSTAFRPLCTGDENISSVETPYIDYTHLRISYNVYLAVYSIAHALQD-IYTCLPGRGLF   444
ENSPPYP00000015087_Pabe/1-1078          NSSTAFRPLCTGDENISSVETPYIDYTHLRISYNVYLAVYSIAHALQD-IYTCLPGRGLF   444
ENSP00000420194_Hsap/1-1088             NSSTAFRPLCTGDENISSVETPYIDYTHLRISYNVYLAVYSIAHALQD-IYTCLPGRGLF   444
ENSGGOP00000020287_Ggor/1-1078          NSSTAFRPLCTGDENISSVETPYIDYTHLRISYNVYLAVYSIAHALQD-IYTCLPGRGLF   444
ENSPPAP00000026371_Ppan/1-1078          NSSTAFRPLCTGDENISSVETPYIDYTHLRISYNVYLAVYSIAHALQD-IYTCLPGKGLF   444
ENSPTRP00000043342_Ptro/1-1078          NSSTAFRPLCTGDENISSVETPYIDYTHLRISYNVYLAVYSIAHALQD-IYTCLPGKGLF   444
ENSCSAP00000003635_Csab/1-882           NSSTAFRPLCTGDENISSVETPYIDYTHLRISYNVYLAVYSIAHALQD-IYTCLPGRGLF   248
ENSMMUP00000061002_Mmul/1-1078          NSSTAFRPLCTGDENISSVETPYIDYTHLRISYNVYLAVYSIAHALQD-IYTCLPGRGLF   444
ENSPANP00000004316_Panu/1-1081          NSSTAFRPLCTGDENISSVETPYIDYTHLRISYNVYLAVYSIAHALQD-IYTCLPGRGLF   437
ENSCATP00000028615_Caty/1-1078          NSSTAFRPLCTGDENISSVETPYIDYTHLRISYNVYLAVYSIAHALQD-IYTCLPGRGLF   444
ENSMFAP00000042132_Mfas/1-1078          NSSTAFRPLCTGDENISSVETPYIDYTHLRISYNVYLAVYSIAHALQD-IYTCLPGRGLF   444
ENSMNEP00000024602_Mnem/1-1078          NSSTAFRPLCTGDENISSVETPYIDYTHLRISYNVYLAVYSIAHALQD-IYTCLPGRGLF   444
ENSMLEP00000008744_Mleu/1-1088          NSSTAFRPLCTGDENISSVETPYIDYTHLRISYNVYLAVYSIAHALQD-IYTCLPGRGLF   444
ENSTGEP00000008766_Tgel/1-1088          NSSTAFRPLCTGDENISSVETPYIDYTHLRISYNVYLAVYSIAHALQD-IYTCLPGRGLF   444
ENSCANP00000040262_Capa/1-1088          NSSTAFRPLCTGDENISSVETPYIDYTHLRISYNVYLAVYSIAHALQD-IYTCLPGRGLF   444
ENSPTEP00000016080_Ptep/1-1088          NSSTAFRPLCTGDENISSVETPYIDYTHLRISYNVYLAVYSIAHALQD-IYTCLPGRGLF   444
ENSRBIP00000006831_Rbie/1-1078          NSSTAFRPLCTGDENISSVETPYIDYTHLRISYNVYLAVYSIAHALQD-IYTCLPGRGLF   444
ENSRROP00000007818_Rrox/1-1088          NSSTAFRPLCTGDENISSVETPYIDYTHLRISYNVYLAVYSIAHALQD-IYTCLPGRGLF   444
ENSOGAP00000002850_Ogar/1-1077          NSSTAFRPLCTGDENISSVETPYMDYTHLRISYNVYLAVYSIAHALQD-IYTCLPGRGLF   444
ENSMICP00000050654_Mmur/1-1075          NSSTAFRPLCTGDENISSVETPYMDYTHLRISYNVYLAVYSIAHALQD-IYTCLPGRGLF   444
ENSPCOP00000009624_Pcoq/1-1076          NSSTAFRPLCTGDENISSVETPYMDYTHLRISYNVYLAVYSIAHALQD-IYTCLPGRGLF   444
ENSPSMP00000007909_Psim/1-1084          NSSTAFRPLCTGDENISTVETPYMDYTHLRISYNVYLAVYSIAHALQD-IYTCLPGRGLF   444


ENSMEUP00000013310_Neug/1-956           TNGSCADIKKVEAWQXXXXXXXXXXXXXXXXXXXXXXXXXXXXXXXXXXXXXXXXXXXXX   441
ENSTBEP00000002487_Tbel/1-914           TNGSCADIKKVEAWQVLKXXXXXXXXXXXXXXXXXXXXXXXXXXXXXXXXXXXX-XXX--   337
ENSPCAP00000000755_Pcap/1-1085          XXXXXXXXXXXXXXXXXXXXXXXXXXXXXXXXX--XXXXXXXXXXXXXXXXXXXXXXX--   499
ENSCHOP00000011907_Chof/1-954           TNGSCADIKKVEAWQVLKHLRHLNFTSNMGEQ-VTFDECGDLVGNYSIINWHLSPEDGSI   502
ENSVPAP00000001982_Vpac/1-1087          XXXXXXXXXXXXXXXVLKHLRHLNFTNNMGEQ-VTFDECGDLAGNYSIINWHLSPEDGSI   502
ENSEEUP00000009670_Eeur/1-946           ----------------LKHLRHINFTNNMGEQ-VTFDECGDLVGNYSIINWHLSPEDSSI   367
ENSOANP00000015961_Oana/1-1083          TNGSCADIKKVEAWQVLKHLRHLNFTSNMGEQ-VDFDEFGDLVGNYSIINWHLSPLDGSI   503
ENSOPRP00000000261_Opri/1-1082          TNGSCADIKKVEAWQVLKHLRHLNFTNNMGEQ-VTFDECGDLMGNYSIINWHLSPEDGSI   503
ENSJJAP00000004721_Jjac/1-1084          TNGSCADIKKVEAWQVLKHLRHLNFTNNMGEQ-VTFDECGDLMGNYSIINWHLSPEDGSI   504
ENSPCIP00000034747_Pcin/1-1018          TNGSCADIKKVEAWQVLKHLRHLNFTNNMGEQ-VDFDEFGDLVGNYSIINWHLSPEDGSI   444
ENSMODP00000022810_Mdom/1-1069          TNGSCADIKKVEAWQVLKHLRHLNFTSNMGEQ-VDFDEFGDLVGNYSIINWHLSPEDGSI   504
ENSVURP00010007344_Vurs/1-1070          TNGSCADIKKVEAWQVLKHLRHLNFTNNMGEQ-VDFDEFGDLVGNYSIINWHLSPEDGSI   504
ENSSHAP00000014890_Shar/1-1072          TNGSCADIKKVEAWQVLKHLRHLNFTNNMGEQ-VDFDEFGDLVGNYSIINWHLSPEDGSI   504
ENSMAUP00000005394_Maur/1-954           TNGSCADIKKVEAWQVLKHLRHLNFTNNMGEQ-AVIDDLL----------------VPSV   487
ENSOCUP00000025672_Ocun/1-1063          ANGSCADIKKVEAWQVLKHLRHLNFTNNMGEH-VNFDECGDLMGNYSIINWHLSPEDGSI   503
ENSPVAP00000011981_Pvam/1-1085          TNGSCADIKKVEAWQVLKHLRHLNFTNNMGEQ-VTFDECGDLMGNYSIINWHLSPEDGSI   503
ENSETEP00000011160_Etel/1-1024          TNGSCADIKKVEAWQVLKHLRHLNFTNNMGEQ-VAFDECGDLMGNYSIINWHLSPEDGSI   441
ENSFDAP00000010532_Fdam/1-1108          TNGSCADIKKVEAWQVLKHLRHLNFTNSMGEQ-VTFDENGNLMGNYSIINWHLSPEDGSI   531
ENSODEP00000013257_Odeg/1-1070          TNSSCADIKKVEAWQVLKHLRHLNFTNNMGEQ-VTFDECGDLVGNYSIINWHLSPEDGSI   503
ENSHGLP00000002019_Hgfe/1-1108          TNGSCADIKKVEAWQVLKHLRHLNFTNNIGEQ-VTFDEYGDLVGNYSIINWHLSPEDGSI   531
ENSCLAP00000021334_Clan/1-1114          TNGSCADIKKVEAWQVLKHLRHLNFTSNIGEQ-VTFDECGDLVGNYSIINWHLSPEDGSI   531
ENSCAPP00000014352_Cape/1-1068          TNGSCADIKKVEAWQVLKHLRHLNFTNNMGEQ-VTFDECGDLVGNYSIINWHLSPEDGSI   503
ENSCPOP00000011705_Cpor/1-1071          TNGSCADIKKVEAWQVLKHLRHLNFTNNMGEQ-VTFDECGDLVGNYSIINWHLSPEDGSI   503
ENSSARP00000006151_Sara/1-1085          TNGSCADIKKVEAWQVLKHLRHINFTNNMGEQ-VTFDECGDLVGNYSIINWHLSPEDGSI   501
ENSLAFP00000018459_Lafr/1-1090          TNGSCADIKKVEAWQVLKHLRHLNFTNNMGEQ-VTFDECGDLVGNYSIINWHLSPEDGSI   503
ENSDNOP00000011819_Dnov/1-1075          TNGSCADIKKVEAWQVLKHLRHLNFTNNMGEQ-VTFDECGDLVGNYSIINWHLSPEDGSI   503
ENSSSCP00000041077_Sscr/1-1089          TNGSCADIKKVEAWQVLKHLRHLNFTSNMGEQ-VTFDEYGDLAGNYSIINWHLSPEDGSI   503
ENSTTRP00000001326_Ttru/1-1091          TNGSCADIKKVEAWQVLKHLRHLNFTNNMGEQ-VTFDECGDLAGNYSIINWHLSPEDGSI   503
ENSBBBP00000002858_Bbbi/1-1068          TNGSCADIKKVEAWQVLKHLRHLNFTSNMGEQ-VTFDECGDLAGNYSIINWHLSPEDGSI   504
ENSOARP00000021602_Oari/1-1032          TNGSCADIKKVEAWQVLKHLRHLNFTSNMGEQ-VTFDECGDLAGNYSIINWHLSPEDGSI   504
ENSCHIP00000033385_Chir/1-1085          TNGSCADIKKVEAWQVLKHLRHLNFTSNMGEQ-VTFDECGDLAGNYSIINWHLSPEDGSI   504
ENSBTAP00000059078_Btau/1-1085          TNGSCADIKKVEAWQVLKHLRHLNFTSNMGEQ-VTFDECGDLAGNYSIINWHLSPEDGSI   504
ENSBIXP00000038534_Bthy/1-1085          TNGSCADIKKVEAWQVLKHLRHLNFTSNMGEQ-VTFDECGDLAGNYSIINWHLSPEDGSI   504
ENSBMUP00000019115_Bmut/1-1081          TNGSCADIKKVEAWQVLKHLRHLNFTSNMGEQ-VTFDECGDLAGNYSIINWHLSPEDGSI   504
ENSBIXP00005030570_Bihy/1-1085          TNGSCADIKKVEAWQVLKHLRHLNFTSNMGEQ-VTFDECGDLAGNYSIINWHLSPEDGSI   504
ENSMUGP00000006365_Mung/1-1080          TNGSCADIKKVEAWQVLKHLRHLNFTNNMGEQ-VAFDECGDLLGNYSIINWHLSPEDGSI   503
ENSRNOP00000069629_Rnor/1-1079          TNGSCADIKKVEAWQVLKHLRHLNFTNNMGEQ-VTFDECGDLVGNYSIINWHLSPEDGSI   503
MGP_PahariEiJ_P0028852_Mpah/1-1003      TNGSCADIKKVEAWQ---------------------------------------------   459
MGP_SPRETEiJ_P0043347_Mspr/1-1079       TNGSCADIKKVEAWQVLKHLRHLNFTNNMGEQ-VTFDECGDLVGNYSIINWHLSPEDGSI   503
ENSMUSP00000069080_Mmus/1-1079          TNGSCADIKKVEAWQVLKHLRHLNFTNNMGEQ-VTFDECGDLVGNYSIINWHLSPEDGSI   503
```

```
ENSMOCP00000010340_Moch/1-1076         TNGSCADIKKVEAWQVLKHLRHLNFTNNMGEQ-VTFDECGDLVGNYSIINWHLSPEDGSI   503
ENSPEMP00000029995_Pmba/1-1076         TNGSCADIKKVEAWQVLKHLRHLNFTNSMGEQ-VTFDECGDLVGNYSIINWHLSPEDGSI   503
ENSCGRP00015024036_Cgpi/1-1081         TNGSCADIKKVEAWQVLKHLRHLNFTNNMGEQ-VAFDECGDLVGNYSIINWHLSPEDGSI   503
ENSCGRP00001016053_Cgch/1-1081         TNGSCADIKKVEAWQVLKHLRHLNFTNNMGEQ-VAFDECGDLVGNYSIINWHLSPEDGSI   503
ENSCGRP00000017369_Cgcr/1-1081         TNGSCADIKKVEAWQVLKHLRHLNFTNNMGEQ-VAFDECGDLVGNYSIINWHLSPEDGSI   503
ENSDORP00000002959_Dord/1-1078         TNGSCADIKKVEAWQVLKHLRHLNFTNNMGEQ-VTFDECGDLMGNYSIINWHLSPEDGSI   503
ENSTSYP00000002643_Csyr/1-1015         TNGSCADIKKVEAWQVLKHLRHLNFTNNMGEW-VTFDECGDLVGNYSIINWHLSPEDGSI   503
ENSMLUP00000008249_Mluc/1-1075         TNGSCADIKKVEAWQVLKHLRHLNFTNNMGEQ-VTFDECGDLVGNYSIINWHLSPEDGSI   502
ENSECAP00000022285_Ecab/1-1084         TNGSCADIKKVEAWQVLKHLRHLNFTNNMGEQ-VTFDECGDLVGNYSIINWHLSPEDGSI   503
ENSEASP00005022676_Eaas/1-1094         TNGSCADIKKVEAWQVLKHLRHLNFTNNMGEQ-VTFDECGDLVGNYSIINWHLSPEDGSI   503
ENSCAFP00000017381_Cfam/1-1075         TNGSCADIKKVEAWQVLKHLRHLNFTNNMGEQ-VTFDECGDLMGNYSIINWHLSPEDGSI   503
ENSCAFP00020007154_Cldi/1-1075         TNGSCADIKKVEAWQVLKHLRHLNFTNNMGEQ-VTFDECGDLMGNYSIINWHLSPEDGSI   503
ENSVVUP00000030826_Vvul/1-1075         TNGSCADIKKVEAWQVLKHLRHLNFTNNMGEQ-VTFDECGDLMGNYSIINWHLSPEDGSI   503
ENSFCAP00000008080_Fcat/1-1081         TNGSCADIKKVEAWQVLKHLRHLNFTNNMGEQ-VTFDECGDLVGNYSIINWHLSPEDGSI   503
ENSPPRP00000023800_Ppar/1-1081         TNGSCADIKKVEAWQVLKHLRHLNFTNNMGEQ-VTFDECGDLVGNYSIINWHLSPEDGSI   503
ENSPTIP00000015042_Ptal/1-1081         TNGSCADIKKVEAWQVLKHLRHLNFTNNMGEQ-VTFDECGDLVGNYSIINWHLSPEDGSI   503
ENSMPUP00000004759_Mpfu/1-1082         TNGSCADIKKVEAWQVLKHLRHLNFTNNMGEQ-VTFDECGDLMGNYSIINWHLSPEDGSI   503
ENSNVIP00000018564_Nvis/1-1092         TNGSCADIKKVEAWQVLKHLRHLNFTNNMGEQ-VTFDECGDLMGNYSIINWHLSPEDGSI   503
ENSUMAP00000035085_Umar/1-1071         TNGSCADIKKVEAWQVLKHLRHLNFTNNMGEQ-VTFDECGDLMGNYSIINWHLSPEDGSI   503
ENSUAMP00000035930_Uame/1-1079         TNGSCADIKKVEAWQVLKHLRHLNFTNNMGEQ-VTFDECGDLMGNYSIINWHLSPEDGSI   503
ENSAMEP00000015060_Amel/1-1089         TNGSCADIKKVEAWQVLKHLRHLNFTNNMGEQ-VTFDECGDLMGNYSIINWHLSPEDGSI   503
ENSSDAP00000007437_Sdau/1-1079         TNGSCADIKKVEAWQVLKHLRHLNFTNNMGEQ-VTFDECGDLVGNYSIINWHLSPEDGSI   503
ENSSTOP00000003945_Itri/1-1070         TNGSCADIKKVEAWQVLKHLRHLNFTNNMGEQ-VTFDECGDLVGNYSIINWHLSPEDGSI   503
ENSMMMP00000015298_Mmma/1-1083         TNGSCADIKKVEAWQVLKHLRHLNFTNNMGEQ-VTFDECGDLVGNYSIINWHLSPEDGSI   503
ENSUPAP00010025947_Upar/1-1086         TNGSCADIKKVEAWQVLKHLRHLNFTNNMGEQ-VTFDECGDLVGNYSIINWHLSPEDGSI   503
ENSSBOP00000036416_Sbbo/1-1078         TNGSCADIKKVEAWQVLKHLRHLNFTNNMGEQ-VTFDECGDLVGNYSIINWHLSPEDGSI   503
ENSANAP00000028404_Anan/1-1078         TNGSCADIKKVEAWQVLKHLRHLNFTNNMGEQ-VTFDECGDLVGNYSIINWHLSPEDGSI   503
ENSCCAP00000024327_Ccap/1-1077         TNGSCADIKKVEAWQVLKHLRHLNFTNNMGEQ-VTFDECGDLVGNYSIINWHLSPEDGSI   503
ENSCJAP00000062854_Cjac/1-1089         TNGSCADIKKVEAWQVLKHLRHLNFTNNMGEQ-VTFDECGDLVGNYSIINWHLSPEDGSI   503
ENSNLEP00000000336_Nleu/1-1088         TNGSCADIKKVEAWQVLKHLRHLNFTNNMGEQ-VTFDECGDLVGNYSIINWHLSAEDGSI   503
ENSPPYP00000015087_Pabe/1-1078         TNGSCADIKKVEAWQVLKHLRHLNFTNNMGEQ-VTFDECGDLVGNYSIINWHLSPEDGSI   503
ENSP00000420194_Hsap/1-1088            TNGSCADIKKVEAWQVLKHLRHLNFTNNMGEQ-VTFDECGDLVGNYSIINWHLSPEDGSI   503
ENSGGOP00000020287_Ggor/1-1078         TNGSCADIKKVEAWQVLKHLRHLNFTNNMGEQ-VTFDECGDLVGNYSIINWHLSPEDGSI   503
ENSPPAP00000026371_Ppan/1-1078         TNGSCADIKKVEAWQVLKHLRHLNFTNNMGEQ-VTFDECGDLVGNYSIINWHLSPEDGSI   503
ENSPTRP00000043342_Ptro/1-1078         TNGSCADIKKVEAWQVLKHLRHLNFTNNMGEQ-VTFDECGDLVGNYSIINWHLSPEDGSI   503
ENSCSAP00000003635_Csab/1-882          TNGSCADIKKVEAWQVLKHLRHLNFTNNMGEQ-VTFDECGDLVGNYSIINWHLSPEDGSI   307
ENSMMUP00000061002_Mmul/1-1078         TNGSCADIKKVEAWQVLKHLRHLNFTNNMGEQ-VTFDECGDLVGNYSIINWHLSPEDGSI   503
ENSPANP00000004316_Panu/1-1081         TNGSCADIKKVEAWQVLKHLRHLNFTNNMGEQ-VTFDECGDLVGNYSIINWHLSPEDGSI   496
ENSCATP00000028615_Caty/1-1078         TNGSCADIKKVEAWQVLKHLRHLNFTNNMGEQ-VTFDECGDLVGNYSIINWHLSPEDGSI   503
ENSMFAP00000042132_Mfas/1-1078         TNGSCADIKKVEAWQVLKHLRHLNFTNNMGEQ-VTFDECGDLVGNYSIINWHLSPEDGSI   503
ENSMNEP00000024602_Mnem/1-1078         TNGSCADIKKVEAWQVLKHLRHLNFTNNMGEQ-VTFDECGDLVGNYSIINWHLSPEDGSI   503
ENSMLEP00000008744_Mleu/1-1088         TNGSCADIKKVEAWQVLKHLRHLNFTNNMGEQ-VTFDECGDLVGNYSIINWHLSPEDGSI   503
ENSTGEP00000008766_Tgel/1-1088         TNGSCADIKKVEAWQVLKHLRHLNFTNNMGEQ-VTFDECGDLVGNYSIINWHLSPEDGSI   503
ENSCANP00000040262_Capa/1-1088         TNGSCADIKKVEAWQVLKHLRHLNFTNNMGEQ-VTFDECGDLVGNYSIINWHLSPEDGSI   503
ENSPTEP00000016080_Ptep/1-1088         TNGSCADIKKVEAWQVLKHLRHLNFTNNMGEQ-VTFDECGDLVGNYSIINWHLSPEDGSI   503
ENSRBIP00000006831_Rbie/1-1078         TNGSCADIKKVEAWQVLKHLRHLNFTNNMGEQ-VTFDECGDLVGNYSIINWHLSPEDGSI   503
ENSRROP00000007818_Rrox/1-1088         TNGSCADIKKVEAWQVLKHLRHLNFTNNMGEQ-VTFDECGDLVGNYSIINWHLSPEDGSI   503
ENSOGAP00000002850_Ogar/1-1077         TNGSCADIKKVEAWQVLKHLRHLNFTNNMGEQ-VTFDECGDLMGNYSIINWHLSPEDSSI   503
ENSMICP00000050654_Mmur/1-1075         TNGSCADIKKVEAWQVLKHLRHLNFTNNMGEQ-VTFDECGDLVGNYSIINWHLSPEDGSI   503
ENSPCOP00000009624_Pcoq/1-1076         TNGSCADIKKVEAWQVLKHLRHLNFTNNMGEQ-VTFDECGDLVGNYSIINWHLSPEDGSI   503
ENSPSMP00000007909_Psim/1-1084         TNGSCADIKKVEAWQVLKHLRHLNFTNNMGEQ-VTFDECGDLVGNYSIINWHLSPEDGSI   503


ENSMEUP00000013310_Neug/1-956          -XX---XXXXXXXXXXXXXXXXXXXXXXXXXXXXXXFIFFLFMLKVPFSNCSRDCLAGTR   497
ENSTBEP00000002487_Tbel/1-914          -XXXXXXXXXXXXXXXXXXXXXXXXXXXXXXXXXXXPFIFMLSVQVPFSNCSRDCLAGTR   396
ENSPCAP00000000755_Pcap/1-1085         -XXXXXXXXXXXXXXXXXXXXXXXXXXXXXXXXXXXSLFLLSVPQVPFSNCSRDCLAT-R   557
ENSCHOP00000011907_Chof/1-954          VFKEVGNYNVYARKGE-RLFINEEKILWSGFSREPLILVLC-VLQVPFSNCSRVCLAGTR   560
ENSVPAP00000001982_Vpac/1-1087         VFKEVGYYNVYAKKGE-RLFINEEKILWSGFSREPLTVVLS-IFQVPFSNCSRDCLAGTR   560
ENSEEUP00000009670_Eeur/1-946          MFKEVGYYNVYAKKGE-RLFINEEKILWSGFSRETLTLVLS-VLQVPFSNCSRDCLAGTR   425
ENSOANP00000015961_Oana/1-1083         EFEEVGYYNVYARKGE-RLFVNENKILWSGFSRE-----------VPFSNCSRDCLAGTR   551
ENSOPRP00000000261_Opri/1-1082         MXXXXXXXXXXXXXXXXXXXXXXXXXXXXXXXXXXXXX-----XXXXXXXXXXXXXXXXX   558
ENSJJAP00000004721_Jjac/1-1084         MFKEVGYYNVYAKKGE-RLFINEEKILWSGFSREVRANCQG-------------------   544
ENSPCIP00000034747_Pcin/1-1018         VFKEVGHYNVYAKKGE-RLFINEEKILWSGFSREVCTE----YKKVPFSNCSRDCLAGTR   499
ENSMODP00000022810_Mdom/1-1069         VFKEVGHYNVYAKKGE-RLFINEEKILWSGFSRE-----------VPFSNCSRDCLAGTR   552
ENSVURP00010007344_Vurs/1-1070         VFKEVGHYNVYAKKGE-RLFINEEKILWSGFSRE-----------VPFSNCSRDCLAGTR   552
ENSSHAP00000014890_Shar/1-1072         VFKEVGHYNVYAKKGE-RLFINEEKILWSGFSRE-----------VPFSNCSRDCLAGTR   552
ENSMAUP00000005394_Maur/1-954          LFAVSLIFLLGLEFSI-HLFIYSLI----------------------IYV----------   514
ENSOCUP00000025672_Ocun/1-1063         MFKEVGYYNVYAKKGE-RLFINEEKILWSGFSRE-----------VPFSNCSRDCLAGTR   551
ENSPVAP00000011981_Pvam/1-1085         VFKEVGYYNVYAKKGE-RLFINEEKILWSGFSREPVPFV-LSVLQVPFSNCSRDCLAGTR   561
ENSETEP00000011160_Etel/1-1024         MFKEVGYYNAYAKKGE-RLFIXXXXXXXXXXXXXPLT---LPVLQVPFSNCSRDCLAGTR   497
ENSFDAP00000010532_Fdam/1-1108         VFKEIGNYNVSAKKGE-RLFINEEKILWSGFSRE-----------VPFSNCSRDCLAGTR   579
ENSODEP00000013257_Odeg/1-1070         MFKEIGNYNVYAKKGE-RLFINENKILWNGFSRE-----------VPFSNCSRDCLAGTR   551
ENSHGLP00000002019_Hgfe/1-1108         MFKEIGNYNVSANKGV-RLFINEEKILWSGFSRE-----------VPFSNCSRDCLAGTR   579
ENSCLAP00000021334_Clan/1-1114         MFKEIGNYNVYAKKGE-RLFINEKKILWNGFSRE-----------VPFSNCSRDCLAGTR   579
ENSCAPP00000014352_Cape/1-1068         VFKEIGNYNAYAKKGE-RLFIDEKKILWSGFSRE-----------VPFSNCSRDCLAGTR   551
ENSCPOP00000011705_Cpor/1-1071         MFKEIGNYNAYAKKGE-RLFINEKKILWSGFSRE-----------VPFSNCSRDCLAGTR   551
ENSSARP00000006151_Sara/1-1085         VFKEVGYYNVYAKKGE-RLFINEEKILWSGFSREPLTFVIS--ILVPFSNCSRDCLAGTR   558
ENSLAFP00000018459_Lafr/1-1090         VFKEVGYYNVYAKKGE-RLFINEEKILWSGFSREPHHSLRALCPQVPFSNCSRDCLAGTR   562
ENSDNOP00000011819_Dnov/1-1075         VFKEVGYYNVYARKGE-RLFINEEKILWSGFSRE-----------VPFSNCSRDCLAGTR   551
ENSSSCP00000041077_Sscr/1-1089         VFKEVGYYNVYAKKGE-RLFINEEKILWSGFSREPLTVVLPI-LQVPFSNCSRDCLAGTR   561
ENSTTRP00000001326_Ttru/1-1091         VFKEVGYYNVYAKKGE-RLFINEEKILWSGFSREPLTIVISV-LQVPFSNCSRDCLAGTR   561
ENSBBBP00000002858_Bbbi/1-1068         VFKEVGYYNVYAKKGE-RLFINDEKILWSGFSREVSPLCCNLRPQVPFSNCSRDCLAGTR   563
ENSOARP00000021602_Oari/1-1032         VFKEVGYYNVYAKKGE-RLFINDEKILWSGFSRE-----------VPFSNCSRDCLAGTR   552
ENSCHIP00000033385_Chir/1-1085         VFKEVGYYNVYAKKGE-RLFINDEKILWSGFSRE-----------VPFSNCSRDCLAGTR   552
ENSBTAP00000059078_Btau/1-1085         VFKEVGYYNVYAKKGE-RLFINDEKILWSGFSRE-----------VPFSNCSRDCLAGTR   552
ENSBIXP00000038534_Bthy/1-1085         VFKEVGYYNVYAKKGE-RLFINDEKILWSGFSRE-----------VPFSNCSRDCLAGTR   552
ENSBMUP00000019115_Bmut/1-1081         VFKEVGYYNVYAKKGE-RLFINDEKILWSGFSRE-----------VPFSNCSRDCLAGTR   552
ENSBIXP00005030570_Bihy/1-1085         VFKEVGYYNVYAKKGE-RLFINDEKILWSGFSRE-----------VPFSNCSRDCLAGTR   552
```

ENSMUGP00000006365_Mung/1-1080	VFKEVGYYNGNARKGD-RLFINEEKILWNGFSRE-----------VPFSNCSRDCLAGTR	551
ENSRNOP00000069629_Rnor/1-1079	VFKEVGYYNVYAKKGE-RLFINEEKILWSGFSRE-----------VPFSNCSRDCQAGTR	551
MGP_PahariEiJ_P0028852_Mpah/1-1003	---VPFSNCSRDCQAGTR	474
MGP_SPRETEiJ_P0043347_Mspr/1-1079	VFKEVGYYNVYAKKGE-RLFINEGKILWSGFSRE-----------VPFSNCSRDCQAGTR	551
ENSMUSP00000069080_Mmus/1-1079	VFKEVGYYNVYAKKGE-RLFINEGKILWSGFSRE-----------VPFSNCSRDCQAGTR	551
ENSMOCP00000010340_Moch/1-1076	VFKEVGHYNAYAKKGE-RLFINEEKILWSGFSRE-----------VPFSNCSRDCLAGTR	551
ENSPEMP00000029995_Pmba/1-1076	VFKEVGHYNVYAKKGE-RLFINEEKILWSGFSRE-----------VPFSNCSRDCLAGTR	551
ENSCGRP00015024036_Cgpi/1-1081	VFREVGHYNVNAKKGE-RLFINEEKILWSGFSRE-----------VPFSNCSRDCLAGTR	551
ENSCGRP00001016053_Cgch/1-1081	VFREVGHYNVNAKKGE-RLFINEEKILWSGFSRE-----------VPFSNCSRDCLAGTR	551
ENSCGRP00000017369_Cgcr/1-1081	VFREVGHYNVNAKKGE-RLFINEEKILWSGFSRE-----------VPFSNCSRDCLAGTR	551
ENSDORP00000002959_Dord/1-1078	VFKEVGYYNVYAKKGE-RLFINEEKILWSGFSRE-----------VPFSNCSRDCLAGTR	551
ENSTSYP00000002643_Csyr/1-1015	MFKEVGYYNVYAKKGE-RLFINEEKILWSGFSREPLTFVLSI-FQVPFSNCSRDCLAGTR	561
ENSMLUP00000008249_Mluc/1-1075	VFKEVGYYNVYAKKGE-RLFINEKKILWSGFSRE-----------VPFSNCSRVCLEGTR	550
ENSECAP00000022285_Ecab/1-1084	VFKEVGYYNVYAKKGE-RLFINEKKILWSGFSRE-----------VPFSNCSRDCLAGTR	551
ENSEASP00005022676_Eaas/1-1094	VFKEVGYYNVYAKKGE-RLFINEKKILWSGFSREPLTSVLSI-FQVPFSNCSRDCLAGTR	561
ENSCAFP00000017381_Cfam/1-1075	VFKEVGYYNVYAKKGE-RLFINEEKILWSGFSRE-----------MPFSNCSRDCLAGTR	551
ENSCAFP00020007154_Cldi/1-1075	VFKEVGYYNVYAKKGE-RLFINEEKILWSGFSRE-----------MPFSNCSRDCLAGTR	551
ENSVVUP00000030826_Vvul/1-1075	VFKEVGYYNVYAKKGE-RLFINEEKILWSGFSRE-----------MPFSNCSRDCLAGTR	551
ENSFCAP00000008080_Fcat/1-1081	VFKEVGYYNVYAKKGE-RLFINEEKILWSGFSRE-----------VPFSNCSRDCLAGTR	551
ENSPPRP00000023800_Ppar/1-1081	VFKEVGYYNVYAKKGE-RLFINEEKILWSGFSRE-----------VPFSNCSRDCLAGTR	551
ENSPTIP00000015042_Ptal/1-1081	VFKEVGYYNVYAKKGE-RLFINEEKILWSGFSRE-----------VPFSNCSRDCLAGTR	551
ENSMPUP00000004759_Mpfu/1-1082	VFKEVGYYNVYAKKGE-RLFINEEKILWSGFSRE-----------VPFSNCSRDCLAGTR	551
ENSNVIP00000018564_Nvis/1-1092	VFKEVGYYNVYAKKGE-RLFIKEEKILWSGFSREPFTFVLSV-LQVPFSNCSRDCLAGTR	561
ENSUMAP00000035085_Umar/1-1071	VFKEVGYYNVYAKKGE-RLFINEEKILWSGFSREPFTFVLSV-LQVPFSNCSRDCLAGTR	561
ENSUAMP00000035930_Uame/1-1079	VFKEVGYYNVYAKKGE-RLFINEEKILWSGFSRE-----------VPFSNCSRDCLAGTR	551
ENSAMEP00000015060_Amel/1-1089	VFKEVGYYNVYAKKGE-RLFINEEKILWSGFSREPFTFVLSV-LQVPFSNCSRDCLAGTR	561
ENSSDAP00000007437_Sdau/1-1079	VFKEVGYYNVYAKKGE-RLFINEEKILWSGFSRE-----------VPFSNCSRDCLAGTR	551
ENSSTOP00000003945_Itri/1-1070	VFKEVGYYNVYAKKGE-RLFINEEKILWSGFSRE-----------VPFSNCSRDCLAGTR	551
ENSMMMP00000015298_Mmma/1-1083	VFKEVGHYNVYAKKGE-RLFINEEKILWSGFSRE-----------VPFSNCSRDCLAGTR	551
ENSUPAP00010025947_Upar/1-1086	MFKEVGYYNVYAKKGE-RLFINEEKILWSGFSRE-----------VPFSNCSRDCLAGTR	551
ENSSBOP00000036416_Sbbo/1-1078	MFKEVGYYNVYAKKGE-RLFINEEKILWSGFSRE-----------VPFSNCSRDCLAGTR	551
ENSANAP00000028404_Anan/1-1078	MFKEVGYYNVYAKKGE-RLFINEEKILWSGFSRE-----------VPFSNCSRDCLAGTR	551
ENSCCAP00000024327_Ccap/1-1077	MFKEVGYYNVYAKKGE-RLFINEEKILWSGFSRE-----------VPFSNCSRDCLAGTR	551
ENSCJAP00000062854_Cjac/1-1089	MFKEVGYYNVYAKKGE-RLFINEEKILWSGFSREPVTFVLLV-LQVPFSNCSRDCLAGTR	561
ENSNLEP00000000336_Nleu/1-1088	VFKEVGYYNVYAKKGE-RLFINEEKILWSGFSREPLTFVLSV-PQVPFSNCSRDCLAGTR	561
ENSPPYP00000015087_Pabe/1-1078	VFKEVGYYNVYAKKGE-RLFINEEKILWSGFSRE-----------VPFSNCSRDCLAGTR	551
ENSP00000420194_Hsap/1-1088	VFKEVGYYNVYAKKGE-RLFINEEKILWSGFSREPLTFVLSV-LQVPFSNCSRDCLAGTR	561
ENSGGOP00000020287_Ggor/1-1078	VFKEVGYYNVYAKKGE-RLFINEEKILWSGFSRE-----------VPFSNCSRDCLAGTR	551
ENSPPAP00000026371_Ppan/1-1078	VFKEVGYYNVYAKKGE-RLFINEEKILWSGFSRE-----------VPFSNCSRDCLAGTR	551
ENSPTRP00000043342_Ptro/1-1078	VFKEVGYYNVYAKKGE-RLFINEEKILWSGFSRE-----------VPFSNCSRDCLAGTR	551
ENSCSAP00000003635_Csab/1-882	VFKEVGYYNVYAKKGE-RLFINEEKILWSGFSRE-----------VPFSNCSRDCLAGTR	355
ENSMMUP00000061002_Mmul/1-1078	VFKEVGYYNVYAKKGE-RLFINEEKILWSGFSRE-----------VPFSNCSRDCLAGTR	551
ENSPANP00000004316_Panu/1-1081	VFKEVGYYNVYAKKGE-RLFINEEKILWSGFSRETLTFVLSV-LQVPFSNCSRDCLAGTR	554
ENSCATP00000028615_Caty/1-1078	VFKEVGYYNVYAKKGE-RLFINEEKILWSGFSRE-----------VPFSNCSRDCLAGTR	551
ENSMFAP00000042132_Mfas/1-1078	VFKEVGYYNVYAKKGE-RLFINEEKILWSGFSRE-----------VPFSNCSRDCLAGTR	551
ENSMNEP00000024602_Mnem/1-1078	VFKEVGYYNVYAKKGE-RLFINEEKILWSGFSRE-----------VPFSNCSRDCLAGTR	551
ENSMLEP00000008744_Mleu/1-1088	VFKEVGYYNVYAKKGE-RLFINEEKILWSGFSRETLTFVLSV-LQVPFSNCSRDCLAGTR	561
ENSTGEP00000008766_Tgel/1-1088	VFKEVGYYNVYAKKGE-RLFINEEKILWSGFSRETLTFVLSV-LQVPFSNCSRDCLAGTR	561
ENSCANP00000040262_Capa/1-1088	MFKEVGYYNVYAKKGE-RLFINEEKILWSGFSREPLTFVLLV-LQVPFSNCSRDCLAGTR	561
ENSPTEP00000016080_Ptep/1-1088	MFKEVGYYNVYAKKGE-RLFINEEKILWSGFSREPLTFVLSI-LQVPFSNCSRDCLAGTR	561
ENSRBIP00000006831_Rbie/1-1078	MFKEVGYYNVYAKKGE-RLFINEEKILWSGFSRE-----------VPFSNCSRDCLAGTR	551
ENSRROP00000007818_Rrox/1-1088	VFKEVGYYNVYAKKGE-RLFINEEKILWSGFSREPLTFVLSI-LQVPFSNCSRDCLAGTR	561
ENSOGAP00000002850_Ogar/1-1077	VFKEVGYYNVYAKKGE-RLFINEEKILWSGFSRE-----------VPFSNCSRDCLAGTR	551
ENSMICP00000050654_Mmur/1-1075	VFKEVGYYNVYAKKGE-RLFINEEKILWSGFSRE-----------VPFSNCSRDCLAGTR	551
ENSPCOP00000009624_Pcoq/1-1076	VFKEVGYYNVYAKKGE-RLFINEEKILWSGFSRE-----------VPFSNCSRDCLAGTR	551
ENSPSMP00000007909_Psim/1-1084	MFKEVGYYNVYAKKGE-RLFINEEKILWSGFSREPLTFVFPV-LQVPFSNCSRDCLAGTR	561

ENSMEUP00000013310_Neug/1-956	KGI-IEGEPTCCFECVECPDGEYSDE--TDASACDKCPDDSWSNENHTSCIAKQIEFLSW	554
ENSTBEP00000002487_Tbel/1-914	KGI-IEGEPTCCFECVECPDGEYSDE--TDASACDKCPDDFWSNENHTSCIAKEIEFLSW	453
ENSPCAP00000000755_Pcap/1-1085	KGI-I--EGPCCFECVECPDGEYSDE--TDASACDKCPDDFWSNENHTSCIAKEIEFLSW	612
ENSCHOP00000011907_Chof/1-954	KGI-IEGEPTCCFECVECPDGEYSDE--TDASACDKCPDDFWSNENHTSCIAKEIEFLSW	617
ENSVPAP00000001982_Vpac/1-1087	KGI-IEGEPTCCFECVECPDGEYSDE--TDASACDKCPDDFWSNENHTSCIAKEIEFLSW	617
ENSEEUP00000009670_Eeur/1-946	KGI-IEGEPTCCFECVECPDGEYSDE--TDASACDKCPDDFWSNENHTSCIAKEIEFLSW	482
ENSOANP00000015961_Oana/1-1083	KGI-IEGEPTCCFECVECPDGEYSGE--TDASACDKCPDDSWSNANHTSCVAKQIEFLSW	608
ENSOPRP00000000261_Opri/1-1082	XXXXXXXXXXXXXXXXXXXXXXXXXXXXXXASACDKCPDDFWSNENHTSCIAKEIEFLAW	618
ENSJJAP00000004721_Jjac/1-1084	------------------TGGYPTST--TPIYACDKCPDDFWSNENHTSCIAKEIEFLSW	584
ENSPCIP00000034747_Pcin/1-1018	KGI-IEGEPTCCFECVECPDGEYSDE--TDASACDKCPDDSWSNENHTSCIAKQIEFLSW	556
ENSMODP00000022810_Mdom/1-1069	KGI-IEGEPTCCFECVECPDGEYSDE--TDASACDKCPDDSWSNENHTSCIAKQIEFLSW	609
ENSVURP00010007344_Vurs/1-1070	KGI-IEGEPTCCFECVECPDGEYSDE--TDASACDKCPDDSWSNENHTSCIAKQIEFLSW	609
ENSSHAP00000014890_Shar/1-1072	KGI-IEGEPTCCFECVECPDGEYSDE--TDASACDKCPDDSWSNENHTSCIAKQIEFLSW	609
ENSMAUP00000005394_Maur/1-954	---------CACFTCI------YVCA--LNASACDKCPDDFWSNENHTSCIAKEIEFLAW	557
ENSOCUP00000025672_Ocun/1-1063	KGI-IEGEPTCCFECVECPDGEYSDE--TDASACDKCPDDFWSNENHTSCIAKEIEFLSW	608
ENSPVAP00000011981_Pvam/1-1085	KGI-IEGEPTCCFECVECPDGEYSDE--TDASACDKCPDDFWSNENHTSCIAKEIEFLSW	618
ENSETEP00000011160_Etel/1-1024	KGI-IEGEPTCCFECVECPDGEYSDE--TDASACDKCPDDFWSNENHTSCIAKEIEFLSW	554
ENSFDAP00000010532_Fdam/1-1108	KGI-IEGEPTCCFECVECPDGEYSDE--TDASACDKCPDDFWSNENHTSCIAKEIEFLAW	636
ENSODEP00000013257_Odeg/1-1070	KGI-IEGEPTCCFECVECPDGEYSDE--TDASACDKCPDDFWSNENHTSCIAKEIEFLEW	608
ENSHGLP00000002019_Hgfe/1-1108	KGI-IEGEPTCCFECVECPDGEYSDE--TDASACDKCPDDFWSNENHTSCIAKEIEFLAW	636
ENSCLAP00000021334_Clan/1-1114	KGI-IEGEPTCCFECVECPDGEYSDE--TDASACDKCPDDFWSNENHTSCIAKEIEFLAW	636
ENSCAPP00000014352_Cape/1-1068	KGI-IEGEPTCCFECVECPDGEYSDE--TDASACDKCPDDFWSNENHTSCIAKEIEFLAW	608
ENSCPOP00000011705_Cpor/1-1071	KGI-IEGEPTCCFECVECPDGEYSDE--TDASACDKCPDDFWSNENHTSCIAKEIEFLAW	608
ENSSARP00000006151_Sara/1-1085	KGI-IEGEPTCCFECVECPDGEYSDE--TDASACDKCPDDFWSNENHTSCIAKEIEFLSW	615
ENSLAFP00000018459_Lafr/1-1090	KGI-IEGEPTCCFECVECPDGEYSDE--TDASACDKCPDDFWSNENHTSCIAKEIEFLSW	619
ENSDNOP00000011819_Dnov/1-1075	KGI-IEGEPTCCFECVECPDGEYSDE--TDASACDKCPDDFWSNENHTSCIAKEIEFLSW	608
ENSSSCP00000041077_Sscr/1-1089	KGI-IEGEPTCCFECVECPDGEYSDE--TDASACDKCPDDFWSNENHTSCIAKEIEFLSW	618
ENSTTRP00000001326_Ttru/1-1091	KGI-IEGEPTCCFECVECPDGEYSDE--TDASACDKCPDDFWSNENHTSCIAKEIEFLSW	618
ENSBBBP00000002858_Bbbi/1-1068	KGI-IEGEPTCCFECVECPDGEYSDE--TDASACDKCPDDFWSNENHTSCIAKEIEFLSW	620
ENSOARP00000021602_Oari/1-1032	KGI-IEGEPTCCFECVECPDGEYSDE--TDASACDKCPDDFWSNENHTSCIAKEIEFLSW	609

```
ENSCHIP00000033385_Chir/1-1085          KGI-IEGEPTCCFECVECPDGEYSDE--TDASACDKCPDDFWSNENHTSCIAKEIEFLSW   609
ENSBTAP00000059078_Btau/1-1085          KGI-IEGEPTCCFECVECPDGEYSDE--TDASACDKCPDDFWSNENHTSCIAKEIEFLSW   609
ENSBIXP00000038534_Bthy/1-1085          KGI-IEGEPTCCFECVECPDGEYSDE--TDASACDKCPDDFWSNENHTSCIAKEIEFLSW   609
ENSBMUP00000019115_Bmut/1-1081          KGI-IEGEPTCCFECVECPDGEYSDE--TDASACDKCPDDFWSNENHTSCIAKEIEFLSW   609
ENSBIXP00005030570_Bihy/1-1085          KGI-IEGEPTCCFECVECPDGEYSDE--TDASACDKCPDDFWSNENHTSCIAKEIEFLSW   609
ENSMUGP00000006365_Mung/1-1080          KGI-IEGEPTCCFECVECPDGEYSDE--TDASACDKCPDDFWSNENHTSCIAKEIEFLAW   608
ENSRNOP00000069629_Rnor/1-1079          KGI-IEGEPTCCFECVECPDGEYSGE--TDASACDKCPDDFWSNENHTSCIAKEIEFLAW   608
MGP_PahariEiJ_P0028852_Mpah/1-1003      KGI-IEGEPTCCFECVECPDGEYSGE--TDASACDKCPDDFWSNENHTSCIAKEIEFLAW   531
MGP_SPRETEiJ_P0043347_Mspr/1-1079       KGI-IEGEPTCCFECVECPDGEYSGE--TDASACDKCPDDFWSNENHTSCIAKEIEFLAW   608
ENSMUSP00000069080_Mmus/1-1079          KGI-IEGEPTCCFECVECPDGEYSGE--TDASACDKCPDDFWSNENHTSCIAKEIEFLAW   608
ENSMOCP00000010340_Moch/1-1076          KGI-IEGEPTCCFECVECPDGEYSDE--TDASACDKCPDDFWSNENHTSCIAKEIEFLAW   608
ENSPEMP00000029995_Pmba/1-1076          KGI-IEGEPTCCFECVECPDGEYSDE--TDASACDKCPDDFWSNENHTSCIAKEIEFLAW   608
ENSCGRP00015024036_Cgpi/1-1081          KGI-IEGEPTCCFECVECPDGEYSDE--TDASACDKCPDDFWSNENHTSCIAKEIEFLAW   608
ENSCGRP00001016053_Cgch/1-1081          KGI-IEGEPTCCFECVECPDGEYSDE--TDASACDKCPDDFWSNENHTSCIAKEIEFLAW   608
ENSCGRP00000017369_Cgcr/1-1081          KGI-IEGEPTCCFECVECPDGEYSDE--TDASACDKCPDDFWSNENHTSCIAKEIEFLAW   608
ENSDORP00000002959_Dord/1-1078          KGI-IEGEPTCCFECVECPDGEYSDE--TDASACDKCPDDFWSNENHTSCIAKEIEFLSW   608
ENSTSYP00000002643_Csyr/1-1015          KGI-IEGEPTCCFECVECPDGEYSDE--TDASACDKCPDDFWSNENHTSCIAKEIEFLAW   618
ENSMLUP00000008249_Mluc/1-1075          KGI-IEGKPTCCFECVKCPDGEYSDE--IDASACDKCPDDFWSSENHTSCIAKEIEFLSW   607
ENSECAP00000022285_Ecab/1-1084          KGI-IEGEPTCCFECVECPDGEYSDE--TDASACDKCPDDFWSNENHTSCIAKEIEFLSW   608
ENSEASP00005022676_Eaas/1-1094          KGI-IEGEPTCCFECVECPDGEYSDE--TDASACDKCPDDFWSNENHTSCIAKEIEFLSW   618
ENSCAFP00000017381_Cfam/1-1075          KGI-IEGEPTCCFECVECPDGEYSDE--TDASACDKCPDDFWSNENHTSCIAKEIEFLSW   608
ENSCAFP00020007154_Cldi/1-1075          KGI-IEGEPTCCFECVECPDGEYSDE--TDASACDKCPDDFWSNENHTSCIAKEIEFLSW   608
ENSVVUP00000030826_Vvul/1-1075          KGI-IEGEPTCCFECVECPDGEYSDE--TDASACDKCPDDFWSNENHTSCIAKEIEFLSW   608
ENSFCAP00000008080_Fcat/1-1081          KGI-IEGEPTCCFECVECPDGEYSDE--TDASACDKCPDDFWSNENHTSCIAKEIEFLSW   608
ENSPPRP00000023800_Ppar/1-1081          KGI-IEGEPTCCFECVECPDGEYSDE--TDASACDKCPDDFWSNENHTSCIAKEIEFLSW   608
ENSPTIP00000015042_Ptal/1-1081          KGI-IEGEPTCCFECVECPDGEYSDE--TDASACDKCPDDFWSNENHTSCIAKEIEFLSW   608
ENSMPUP00000004759_Mpfu/1-1082          KGI-IEGEPTCCFECVECPDGEYSDE--TDASACDKCPDDFWSNENHTSCIAKEIEFLSW   608
ENSNVIP00000018564_Nvis/1-1092          KGI-IEGEPTCCFECVECPDGEYSDE--TDASACDKCPDDFWSNENHTSCIAKEIEFLSW   618
ENSUMAP00000035085_Umar/1-1071          KGI-IEGEPTCCFECVECPDGEYSDE--TDASACDKCPDDFWSNENHTSCIAKEIEFLSW   618
ENSUAMP00000035930_Uame/1-1079          KGI-IEGEPTCCFECVECPDGEYSDE--TDASACDKCPDDFWSNENHTSCIAKEIEFLSW   608
ENSAMEP00000015060_Amel/1-1089          KGI-IEGEPTCCFECVECPDGEYSDE--TDASACDKCPDDFWSNENHTSCIAKEIEFLSW   618
ENSSDAP00000007437_Sdau/1-1079          KGI-IEGEPTCCFECVECADGEYSDE--TDASACIKCPDDFWSNENHTSCIAKEIEFLSW   608
ENSSTOP00000003945_Itri/1-1070          KGI-IEGEPTCCFECVECADGEYSDE--TDASACIKCPDDFWSNENHTSCIAKEIEFLSW   608
ENSMMMP00000015298_Mmma/1-1083          KGI-IEGEPTCCFECVECADGEYSDE--TDASACIKCPDDFWSNENHTSCIAKEIEFLSW   608
ENSUPAP00010025947_Upar/1-1086          KGI-IEGEPTCCFECVECADGEYSDE--TDASACIKCPDDFWSNENHTSCIAKEIEFLSW   608
ENSSBOP00000036416_Sbbo/1-1078          KGI-IEGEPTCCFECVECPDGEYSDE--TDASACNKCPDDFWSNENHTSCIAKEIEFLSW   608
ENSANAP00000028404_Anan/1-1078          KGI-IEGEPTCCFECVECPDGEYSDE--TDASACNKCPDDFWSNENHTSCIAKEIEFLSW   608
ENSCCAP00000024327_Ccap/1-1077          KGI-IEGEPTCCFECVECPDGEYSDE--TDASACNKCPDDFWSNENHTSCIAKEIEFLSW   608
ENSCJAP00000062854_Cjac/1-1089          KGI-IEGEPTCCFECVECPDGEYSDE--TDASACNKCPDDFWSNENHTSCIAKEIEFLSW   618
ENSNLEP00000000336_Nleu/1-1088          KGI-IEGEPTCCFECVECPDGEYSDE--TDASACNKCPDDFWSNENHTSCIAKEIEFLSW   618
ENSPPYP00000015087_Pabe/1-1078          KGI-IEGEPTCCFECVECPDGEYSDE--TDASACNKCPDDFWSNENHTSCIAKEIEFLSW   608
ENSP00000420194_Hsap/1-1088             KGI-IEGEPTCCFECVECPDGEYSDE--TDASACNKCPDDFWSNENHTSCIAKEIEFLSW   618
ENSGGOP00000020287_Ggor/1-1078          KGI-IEGEPTCCFECVECPDGEYSDE--TDASACNKCPDDFWSNENHTSCIAKEIEFLSW   608
ENSPPAP00000026371_Ppan/1-1078          KGI-IEGEPTCCFECVECPDGEYSDE--TDASACNKCPDDFWSNENHTSCIAKEIEFLSW   608
ENSPTRP00000043342_Ptro/1-1078          KGI-IEGEPTCCFECVECPDGEYSDE--TDASACNKCPDDFWSNENHTSCIAKEIEFLSW   608
ENSCSAP00000003635_Csab/1-882           KGI-IEGEPTCCFECVECPDGEYSDE--TDASACNKCPDDFWSNENHTSCIAKEIEFLSW   412
ENSMMUP00000061002_Mmul/1-1078          KGI-IEGEPTCCFECVECPDGEYSDE--TDASACNKCPDDFWSNENHTSCIAKEIEFLSW   608
ENSPANP00000004316_Panu/1-1081          KGI-IEGEPTCCFECVECPDGEYSDE--TDASACNKCPDDFWSNENHTSCIAKEIEFLSW   611
ENSCATP00000028615_Caty/1-1078          KGI-IEGEPTCCFECVECPDGEYSDE--TDASACNKCPDDFWSNENHTSCIAKEIEFLSW   608
ENSMFAP00000042132_Mfas/1-1078          KGI-IEGEPTCCFECVECPDGEYSDE--TDASACNKCPDDFWSNENHTSCIAKEIEFLSW   608
ENSMNEP00000024602_Mnem/1-1078          KGI-IEGEPTCCFECVECPDGEYSDE--TDASACNKCPDDFWSNENHTSCIAKEIEFLSW   608
ENSMLEP00000008744_Mleu/1-1088          KGI-IEGEPTCCFECVECPDGEYSDE--TDASACNKCPDDFWSNENHTSCIAKEIEFLSW   618
ENSTGEP00000008766_Tgel/1-1088          KGI-IEGEPTCCFECVECPDGEYSDE--TDASACNKCPDDFWSNENHTSCIAKEIEFLSW   618
ENSCANP00000040262_Capa/1-1088          KGI-IEGEPTCCFECVECPDGEYSDE--TDASACDKCPDDFWSNENHTSCIAKEIEFLSW   618
ENSPTEP00000016080_Ptep/1-1088          KGI-IEGEPTCCFECVECPDGEYSDE--TDASACDKCPDDFWSNENHTSCIAKEIEFLSW   618
ENSRBIP00000006831_Rbie/1-1078          KGI-IEGEPTCCFECVECPDGEYSDE--TDASACNKCPDDFWSNENHTSCIAKEIEFLSW   608
ENSRROP00000007818_Rrox/1-1088          KGI-IEGEPTCCFECVECPDGEYSDE--TDASACNKCPDDFWSNENHTSCIAKEIEFLSW   618
ENSOGAP00000002850_Ogar/1-1077          KGI-IEGEPTCCFECVECPDGEYSDE--TDASACDKCPDDFWSNENHTSCIAKEIEFLSW   608
ENSMICP00000050654_Mmur/1-1075          KGI-IEGEPTCCFECVECPDGEYSDE--TDASACDKCPDDFWSNENHTSCIAKEIEFLSW   608
ENSPCOP00000009624_Pcoq/1-1076          KGI-IEGEPTCCFECVECPDGEYSDE--TDASACDKCPDDFWSNENHTSCIAKEIEFLSW   608
ENSPSMP00000007909_Psim/1-1084          KGI-IEGEPTCCFECVECPDGEYSDE--TDASACDKCPDDFWSNENHTSCIAKEIEFLSW   618
                                                                         ** ***** **. *****:**:**** *

ENSMEUP00000013310_Neug/1-956           TEPFGIALTLFAVLGIFLTSFVLGVFIKFRNTPIVKATNRELSYLLLFSLICCFSSSLFF   614
ENSTBEP00000002487_Tbel/1-914           TEPFGIALTLFAVLGIFLTAFVLGVFIKFRNTPIVKATNRELSYLLLFSLLCCFSSSLFF   513
ENSPCAP00000000755_Pcap/1-1085          TEPFGIALTLFAVLGILLTAFVLGVFIKFRNTPIVKATNRELSYLLLFSLLCCFSSSLFF   672
ENSCHOP00000011907_Chof/1-954           TEPFGIALTLFAVLGIFLTAFVLGVFIRFRNTPIVKATNRELSYLLLFSLLCCFSSSLFF   677
ENSVPAP00000001982_Vpac/1-1087          TEPFGIALTLFAVLGIFLTAFLLAVFIKFRNTPIVKATNRELSYLLLFSLLCCFSSSLFF   677
ENSEEUP00000009670_Eeur/1-946           TEPFGIALTLFAVLGIFLTTFVLGVFIKFRNTPIVKATNRELSYLLLFSLLCCFSSSLFF   542
ENSOANP00000015961_Oana/1-1083          TEPFGIALTLFAVLGIFLTSFVLGVFLKFRNTPIVKATNRELSYLLLFSLLCCFSSSLFF   668
ENSOPRP00000000261_Opri/1-1082          TEPFGIALTLFAVLGIFLTAFVLGVFIKFRNTPIVKATNRELSYLLLFSLLCCFSSSLFF   678
ENSJJAP00000004721_Jjac/1-1084          TEPFGIALTLFAVLGICLTAFVLGVFIKFRNTPMVKATNRELSYLLLFSLLCCFSSSLLF   644
ENSPCIP00000034747_Pcin/1-1018          TEPFGIALTLFAVLGIFLTSFVLGVFIKFRNTPIVKATNRELSYLLLFSLICCFSSSLFF   616
ENSMODP00000022810_Mdom/1-1069          TEPFGIALTLFAVLGIFLTSFVLGVFIKFRNTPIVKATNRELSYLLLFSLLCCFSSSLFF   669
ENSVURP00010007344_Vurs/1-1070          TEPFGIALTLFAVLGIFLTSFVLGVFIKFRNTPIVKATNRELSYLLLFSLICCFSSSLFF   669
ENSSHAP00000014890_Shar/1-1072          TEPFGIALTLFAVLGIFLTSFVLGVFIKFRNTPIVKATNRELSYLLLFSLICCFSSSLFF   669
ENSMAUP00000005394_Maur/1-954           TEPFGIALTLFAVLGIFLTAFVLGVFIKFRNTPIVKATNRELSYLLLFSLLCCFSSSLFF   617
ENSOCUP00000025672_Ocun/1-1063          TEPFGIALTLLAVLGIFLTAFVLGVFIKFRNTPIVKATNRELSYLLLFSLLCCFSSSLFF   668
ENSPVAP00000011981_Pvam/1-1085          TEPFGIALTLFAVLGIFLTAFVLGVFIKFRNTPIVKATNRELSYLLLFSLLCCFSSSLFF   678
ENSETEP00000011160_Etel/1-1024          TEPFGIALTLFAVLGIFLTAFVLGVFIKFRNTPIVKATNRELSYLLLFSLLCCFSSSLFF   614
ENSFDAP00000010532_Fdam/1-1108          TEPFGIALTLLAVLGIFLTAFVLGVFIKFRNTPIVKATNRELSYLLLFSLLCCFSSSLFF   696
ENSODEP00000013257_Odeg/1-1070          TEPFGIALTLFAVLGIFLTAFVLGVFIKFRNTPIVKATNRELSYLLLFSLLCCFSSSLFF   668
ENSHGLP00000002019_Hgfe/1-1108          TEPFGIALTLFAVLGIFLTAFVLGVFIKFRNTPIVKATNRELSYLLLFSLLCCFSSSLFF   696
ENSCLAP00000021334_Clan/1-1114          TEPFGIALTLFAVLGIFLTTFVLGVFIKFRNTPIVKATNRELSYLLLFSLLCCFSSSLFF   696
ENSCAPP00000014352_Cape/1-1068          TEPFGIALTLFAVLGIFLTAFVLGVFIKFRNTPIVKATNRELSYLLLFSLLCCFSSSLFF   668
ENSCPOP00000011705_Cpor/1-1071          TEPFGIALTLFAVLGIFLTAFVLGVFIKFRNTPIVKATNRELSYLLLFSLLCCFSSSLFF   668
ENSSARP00000006151_Sara/1-1085          TEPFGIALTLFAVLGIFLTAFVLGVFIKFRNTPIVKATNRELSYLLLFSLLCCFSSSLFF   675
ENSLAFP00000018459_Lafr/1-1090          TEPFGIALTLFAVLGIFLTAFVLGVFIKFRNTPIVKATNRELSYVLLFSLLCCFSSSLFF   679
```

```
ENSDNOP00000011819_Dnov/1-1075          TEPFGIALTLFAVLGILLTAFVLGVFIKFRNTPIVKATNRELSYLLLFSLLCCFSSSLFF   668
ENSSSCP00000041077_Sscr/1-1089          TEPFGIALTLFAVLGIFLTAFVLGVFIKFRNTPIVKATNRELSYLLLFSLLCCFSSSLFF   678
ENSTTRP00000001326_Ttru/1-1091          TEPFGIALTLFAVLGIFLTAFVLGVFIKFRNTPIVKATNRELSYLLLFSLLCCFSSSLFF   678
ENSBBBP00000002858_Bbbi/1-1068          TEPFGIALTLFAVLGIFLTAFVLGVFIKFRNTPIVKATNRELSYLLLFSLLCCFSSSLFF   680
ENSOARP00000021602_Oari/1-1032          TEPFGIALTLFAVLGIFLTAFVLGVFIKFRNTPIVKATNRELSYLLLFSLLCCFSSSLFF   669
ENSCHIP00000033385_Chir/1-1085          TEPFGIALTLFAVLGIFLTAFVLGVFIKFRNTPIVKATNRELSYLLLFSLLCCFSSSLFF   669
ENSBTAP00000059078_Btau/1-1085          TEPFGIALTLFAVLGIFLTAFVLGVFIKFRNTPIVKATNRELSYLLLFSLLCCFSSSLFF   669
ENSBIXP00000038534_Bthy/1-1085          TEPFGIALTLFAVLGIFLTAFVLGVFIKFRNTPIVKATNRELSYLLLFSLLCCFSSSLFF   669
ENSBMUP00000019115_Bmut/1-1081          TEPFGIALTLFAVLGIFLTAFVLGVFIKFRNTPIVKATNRELSYLLLFSLLCCFSSSLFF   669
ENSBIXP00005030570_Bihy/1-1085          TEPFGIALTLFAVLGIFLTAFVLGVFIKFRNTPIVKATNRELSYLLLFSLLCCFSSSLFF   669
ENSMUGP00000006365_Mung/1-1080          TEPFGIALTLFAVLGIFLTAFVLGVFIKFRNTPIVKATNRELSYLLLFSLLCCFSSSLFF   668
ENSRNOP00000069629_Rnor/1-1079          TEPFGIALTLFAVLGIFLTAFVLGVFIKFRNTPIVKATNRELSYLLLFSLLCCFSSSLFF   668
MGP_PahariEiJ_P0028852_Mpah/1-1003      TEPFGIALTLFAVLGIFLTAFVLGVFIKFRNTPIVKATNRELSYLLLFSLLCCFSSSLFF   591
MGP_SPRETEiJ_P0043347_Mspr/1-1079       TEPFGIALTLFAVLGIFLTAFVLGVFIKFRNTPIVKATNRELSYLLLFSLLCCFSSSLFF   668
ENSMUSP00000069080_Mmus/1-1079          TEPFGIALTLFAVLGIFLTAFVLGVFIKFRNTPIVKATNRELSYLLLFSLLCCFSSSLFF   668
ENSMOCP00000010340_Moch/1-1076          TEPFGIALTLFAVLGIFLTAFVLGVFIKFRNTPIVKATNRELSYLLLFSLLCCFSSSLFF   668
ENSPEMP00000029995_Pmba/1-1076          TEPFGIALTLFAVLGIFLTAFVLGVFIKFRNTPIVKATNRELSYLLLFSLLCCFSSSLFF   668
ENSCGRP00015024036_Cgpi/1-1081          TEPFGIALTLFAVLGIFLTAFVLGVFIKFRNTPIVKATNRELSYLLLFSLLCCFSSSLFF   668
ENSCGRP00001016053_Cgch/1-1081          TEPFGIALTLFAVLGIFLTAFVLGVFIKFRNTPIVKATNRELSYLLLFSLLCCFSSSLFF   668
ENSCGRP00000017369_Cgcr/1-1081          TEPFGIALTLFAVLGIFLTAFVLGVFIKFRNTPIVKATNRELSYLLLFSLLCCFSSSLFF   668
ENSDORP00000002959_Dord/1-1078          TEPFGIALTLFAVLGIFLTAFVLGVFIKFRNTPIVKATNRELSYLLLFSLLCCFSSSLFF   668
ENSTSYP00000002643_Csyr/1-1015          TEPFGIALTLFAVLGIFLTASVLGVFIKFRNTPIVKATNRELSYLLLFSLLCCFSSSLFF   678
ENSMLUP00000008249_Mluc/1-1075          TEPFGIALTLFAVLGIFLTAFVLGVFIKFRNTPIVKATNRELSYLLLFSLLCCFSSSLFF   667
ENSECAP00000022285_Ecab/1-1084          TEPFGIALTLFAVLGIFLTGFVLGVFIKFRNTPIVKATNRELSYLLLFSLLCCFSSSLFF   668
ENSEASP00005022676_Eaas/1-1094          TEPFGIALTLFAVLGIFLTGFVLGVFIKFRNTPIVKATNRELSYLLLFSLLCCFSSSLFF   678
ENSCAFP00000017381_Cfam/1-1075          TEPFGIALTLFAVLGIFLTAFVLGVFIKFRNTPIVKATNRELSYLLLFSLLCCFSSSLFF   668
ENSCAFP00020007154_Cldi/1-1075          TEPFGIALTLFAVLGIFLTAFVLGVFIKFRNTPIVKATNRELSYLLLFSLLCCFSSSLFF   668
ENSVVUP00000030826_Vvul/1-1075          TEPFGIALTLFAVLGIFLTAFVLGVFIKFRNTPIVKATNRELSYLLLFSLLCCFSSSLFF   668
ENSFCAP00000008080_Fcat/1-1081          TEPFGIALTLFAVLGIFLTAFVLGVFLKFRNTPIVKATNRELSYLLLFSLLCCFSSSLFF   668
ENSPPRP00000023800_Ppar/1-1081          TEPFGIALTLFAVLGIFLTAFVLGVFLKFRNTPIVKATNRELSYLLLFSLLCCFSSSLFF   668
ENSPTIP00000015042_Ptal/1-1081          TEPFGIALTLFAVLGIFLTAFVLGVFLKFRNTPIVKATNRELSYLLLFSLLCCFSSSLFF   668
ENSMPUP00000004759_Mpfu/1-1082          TEPFGIALTLFAVLGIFLTAFVLGVFIKFRNTPIVKATNRELSYLLLFSLLCCFSSSLFF   668
ENSNVIP00000018564_Nvis/1-1092          TEPFGIALTLFAVLGIFLTAFVLGVFIKFRNTPIVKATNRELSYLLLFSLLCCFSSSLFF   678
ENSUMAP00000035085_Umar/1-1071          TEPFGIALTLFAVLGIFLTAFVLGVFIKFCNTPIVKATNRELSYLLLFSLLCCFSSSLFF   678
ENSUAMP00000035930_Uame/1-1079          TEPFGIALTLFAVLGIFLTAFVLGVFIKFCNTPIVKATNRELSYLLLFSLLCCFSSSLFF   668
ENSAMEP00000015060_Amel/1-1089          TEPFGIALTLFAVLGIFLTAFVLGVFIKFCNTPIVKATNRELSYLLLFSLLCCFSSSLFF   678
ENSSDAP00000007437_Sdau/1-1079          TEPFGIALTLFAVLGIFLTAFVLGVFIKFRNTPIVKATNRELSYLLLFSLLCCFSSSLFF   668
ENSSTOP00000003945_Itri/1-1070          TEPFGIALTLFAVLGIFLTAFVLGVFIKFRNTPIVKATNRELSYLLLFSLLCCFSSSLFF   668
ENSMMMP00000015298_Mmma/1-1083          TEPFGIALTLFAVLGIFLTAFVLGVFIKFRNTPIVKATNRELSYLLLFSLLCCFSSSLFF   668
ENSUPAP00010025947_Upar/1-1086          TEPFGIALTLFAVLGIFLTAFVLGVFIKFRNTPIVKATNRELSYLLLFSLLCCFSSSLFF   668
ENSSBOP00000036416_Sbbo/1-1078          TEPFGIALTLFAVLGIFLTAFVLGVFIKFRNTPIVKATNRELSYLLLFSLLCCFSSSLFF   668
ENSANAP00000028404_Anan/1-1078          TEPFGIALTLFAVLGIFLTAFVLGVFIKFRNTPIVKATNRELSYLLLFSLLCCFSSSLFF   668
ENSCCAP00000024327_Ccap/1-1077          TEPFGIALTLFAVLGIFLTAFVLGVFIKFRNTPIVKATNRELSYLLLFSLLCCFSSSLFF   668
ENSCJAP00000062854_Cjac/1-1089          TEPFGIALTLFAVLGIFLTAFVLGVFIKFRNTPIVKATNRELSYLLLFSLLCCFSSSLFF   678
ENSNLEP00000000336_Nleu/1-1088          TEPFGIALTLFAVLGIFLTAFVLGVFIKFRNTPIVKATNRELSYLLLFSLLCCFSSSLFF   678
ENSPPYP00000015087_Pabe/1-1078          TEPFGIALTLFAVLGIFLTAFVLGVFIKFRNTPIVKATNRELSYLLLFSLLCCFSSSLFF   668
ENSP00000420194_Hsap/1-1088             TEPFGIALTLFAVLGIFLTAFVLGVFIKFRNTPIVKATNRELSYLLLFSLLCCFSSSLFF   678
ENSGGOP00000020287_Ggor/1-1078          TEPFGIALTLFAVLGIFLTAFVLGVFIKFRNTPIVKATNRELSYLLLFSLLCCFSSSLFF   668
ENSPPAP00000026371_Ppan/1-1078          TEPFGIALTLFAVLGIFLTAFVLGVFIKFRNTPIVKATNRELSYLLLFSLLCCFSSSLFF   668
ENSPTRP00000043342_Ptro/1-1078          TEPFGIALTLFAVLGIFLTAFVLGVFIKFRNTPIVKATNRELSYLLLFSLLCCFSSSLFF   668
ENSCSAP00000003635_Csab/1-882           TEPFGIALTLFAVLGIFLTAFVLGVFIKFRNTPIVKATNRELSYLLLFSLLCCFSSSLFF   472
ENSMMUP00000061002_Mmul/1-1078          TEPFGIALTLFAVLGIFLTAFVLGVFIKFRNTPIVKATNRELSYLLLFSLLCCFSSSLFF   668
ENSPANP00000004316_Panu/1-1081          TEPFGIALTLFAVLGIFLTAFVLGVFIKFRNTPIVKATNRELSYLLLFSLLCCFSSSLFF   671
ENSCATP00000028615_Caty/1-1078          TEPFGIALTLFAVLGIFLTAFVLGVFIKFRNTPIVKATNRELSYLLLFSLLCCFSSSLFF   668
ENSMFAP00000042132_Mfas/1-1078          TEPFGIALTLFAVLGIFLTAFVLGVFIKFRNTPIVKATNRELSYLLLFSLLCCFSSSLFF   668
ENSMNEP00000024602_Mnem/1-1078          TEPFGIALTLFAVLGIFLTAFVLGVFIKFRNTPIVKATNRELSYLLLFSLLCCFSSSLFF   668
ENSMLEP00000008744_Mleu/1-1088          TEPFGIALTLFAVLGIFLTAFVLGVFIKFRNTPIVKATNRELSYLLLFSLLCCFSSSLFF   678
ENSTGEP00000008766_Tgel/1-1088          TEPFGIALTLFAVLGIFLTAFVLGVFIKFRNTPIVKATNRELSYLLLFSLLCCFSSSLFF   678
ENSCANP00000040262_Capa/1-1088          TEPFGIALTLFAVLGIFLTAFVLGVFIKFRNTPIVKATNRELSYLLLFSLLCCFSSSLFF   678
ENSPTEP00000016080_Ptep/1-1088          TEPFGIALTLFAVLGIFLTAFVLGVFIKFRNTPIVKATNRELSYLLLFSLLCCFSSSLFF   678
ENSRBIP00000006831_Rbie/1-1078          TEPFGIALTLFAVLGIFLTAFVLGVFIKFRNTPIVKATNRELSYLLLFSLLCCFSSSLFF   668
ENSRROP00000007818_Rrox/1-1088          TEPFGIALTLFAVLGIFLTAFVLGVFIKFRNTPIVKATNRELSYLLLFSLLCCFSSSLFF   678
ENSOGAP00000002850_Ogar/1-1077          TEPFGIALTLFAVLGIFLTAFVLGVFIKFRNTPIVKATNRELSYLLLFSLLCCFSSSLFF   668
ENSMICP00000050654_Mmur/1-1075          TEPFGIALTLFAVLGILLTAFVLGVFIKFRNTPIVKATNRELSYLLLFSLLCCFSSSLFF   668
ENSPCOP00000009624_Pcoq/1-1076          TEPFGIALTLFAVLGILLTAFVLGVFIKFRNTPIVKATNRELSYLLLFSLLCCFSSSLFF   668
ENSPSMP00000007909_Psim/1-1084          TEPFGIALTLFAVLGILLTAFVLGVFIKFRNTPIVKATNRELSYLLLFSLLCCFSSSLFF   678
                                        **********:***** **  :*.**::* ***:**********:*****:*******:*

ENSMEUP00000013310_Neug/1-956           IGEPQDWTCCLRQPAFGISFVLCISCILVKTNRILLVFEAKIPTSLHRKWWGLNLQFLLV   674
ENSTBEP00000002487_Tbel/1-914           IGEPQDWTCRLRQPAFGISFVLCISCILVKTNRVLLVFEAKIPTSFHRKWWGLNLQFLLV   573
ENSPCAP00000000755_Pcap/1-1085          IGEPQDWTCRLRQPAFGISFVLCISCILVKTNRVLLVFEAKIPTSFHRKWWGLNLQFLLV   732
ENSCHOP00000011907_Chof/1-954           IGEPQNWTCRLRQPAFGISFVLCISCILVKTNRVLLVFEAKIPTSFHRKWWGLNLQFLLV   737
ENSVPAP00000001982_Vpac/1-1087          IGEPQDWTCRLRQPAFGISFVLCISCILVKTNRVLLVFEAKIPTSFHRKWWGLNLQFLLV   737
ENSEEUP00000009670_Eeur/1-946           IGEPQDWTCRLRQPAFGISFVLCISCILVKTNNILLVFEAKIPTSFHRKWWGLNLQFLLV   602
ENSOANP00000015961_Oana/1-1083          IGEPRDWTCRLRQPAFGTSFVLCVSCILVKTNRVLLAFEAKIPTGLHRKWWGLNLQFLLV   728
ENSOPRP00000000261_Opri/1-1082          IGEPQDWNCRLRQPAFGISFVLCISCILVKTNRVLLVFEAKIPTSFHRKWWGLNLQFLLV   738
ENSJJAP00000004721_Jjac/1-1084          IGEPQDWTCCLRQPAFGISFVLCISCILVKTNRVLLVFEAKIPTSFHRKWWGLNLQFLLV   704
ENSPCIP00000034747_Pcin/1-1018          IGEPQDWTCCLRQPAFGISFVLCISCILVKTNRILLVFEAKIPTSLHRKWWGLNLQFLLV   676
ENSMODP00000022810_Mdom/1-1069          IGEPQDWTCCLRQPAFGISFVLCISCILVKTNRILLVFEAKIPTSLHRKWWGLNLQFLLV   729
ENSVURP00010007344_Vurs/1-1070          IGEPQDWTCCLRQPAFGISFVLCISCILVKTNRILLVFEAKIPTSLHRKWWGLNLQFLLV   729
ENSSHAP00000014890_Shar/1-1072          IGEPQDWTCCLRQPAFGISFVLCISCILVKTNRILLVFEAKIPTSLHRKWWGLNLQFLLV   729
ENSMAUP00000005394_Maur/1-954           IGEPQDWTCRLRQPAFGISFVLCISCILVKTNRVLLVFEAKIPTSFHRKWWGLNLQFLLV   677
ENSOCUP00000025672_Ocun/1-1063          IGEPQDWTCRLRQPAFGISFVLCISCILVKTNRVLLVFEAKIPTSFHRKWWGLNLQFLLV   728
ENSPVAP00000011981_Pvam/1-1085          IGEP-DWTCRLRQPAFGISFVLCISCILVKTNRVLLVFEAKIPTSFHRKWWGLNLQFLLV   737
ENSETEP00000011160_Etel/1-1024          IGEPHDWTCRLRQPAFGISFVLCISCILVKTNRVLLVFEAKIPTSFHRKWWGLNLQFLVV   674
ENSFDAP00000010532_Fdam/1-1108          IGEPQDWTCRLRQPAFGISFVLCISCILVKTNRVLLVFEAKIPTSFHRKWWGLNLQFLLV   756
ENSODEP00000013257_Odeg/1-1070          IGEPQDWTCRLRQPAFGISFVLCISCILVKTNRVLLVFEAKIPTSFHRKWWGLNLQFLLV   728
ENSHGLP00000002019_Hgfe/1-1108          IGEPQDWTCRLRQPAFGISFVLCISCILVKTNRVLLVFEAKIPTSFHRKWWGLNLQFLLV   756
```

```
ENSCLAP00000021334_Clan/1-1114        IGEPQDWTCRLRQPAFGISFVLCISCILVKTNRVLLVFEAKIPTSFHRKWWGLNLQFLLV   756
ENSCAPP00000014352_Cape/1-1068        IGEPQDWTCRLRQPAFGISFVLCISCILVKTNRVLLVFEAKIPTSFHRKWWGLNLQFLLV   728
ENSCPOP00000011705_Cpor/1-1071        IGEPQDWTCRLRQPAFGISFVLCISCILVKTNRVLLVFEAKIPTSFHRKWWGLNLQFLLV   728
ENSSARP00000006151_Sara/1-1085        IGEPQDWTCRLRQPAFGISFVLCISCILVKTNRVLLVFEAKIPTSFHRKWWGLNLQFLLV   735
ENSLAFP00000018459_Lafr/1-1090        IGEPQDWTCRLRQPAFGISFVLCISCILVKTNRVLLVFEAKIPTSFHRKWWGLNLQFLLV   739
ENSDNOP00000011819_Dnov/1-1075        IGEPQNWTCRLRQPAFGISFVLCISCILVKTNRVLLVFEAKIPTSFHRKWWGLNLQFLLV   728
ENSSSCP00000041077_Sscr/1-1089        IGEPQDWTCRLRQPAFGISFVLCISCILVKTNRVLLVFEAKIPTSFHRKWWGLNLQFLLV   738
ENSTTRP00000001326_Ttru/1-1091        IGEPQDWTCRLRQPAFGISFVLCISCILVKTNRVLLVFEAKIPTSFHRKWWGLNLQFLLV   738
ENSBBBP00000002858_Bbbi/1-1068        IGEPQDWTCRLRQPAFGISFVLCISCILVKTNRVLLVFEAKIPTSFHRKWWGLNLQFLLV   740
ENSOARP00000021602_Oari/1-1032        IGEPQDWTCRLRQPAFGISFVLCISCILVKTNRVLLVFEAKIPTSFHRKWWGLNLQFLLV   729
ENSCHIP00000033385_Chir/1-1085        IGEPQDWTCRLRQPAFGISFVLCISCILVKTNRVLLVFEAKIPTSFHRKWWGLNLQFLLV   729
ENSBTAP00000059078_Btau/1-1085        IGEPQDWTCRLRQPAFGISFVLCISCILVKTNRVLLVFEAKIPTSFHRKWWGLNLQFLLV   729
ENSBIXP00000038534_Bthy/1-1085        IGEPQDWTCRLRQPAFGISFVLCISCILVKTNRVLLVFEAKIPTSFHRKWWGLNLQFLLV   729
ENSBMUP00000019115_Bmut/1-1081        IGEPQDWTCRLRQPAFGISFVLCISCILVKTNRVLLVFEAKIPTSFHRKWWGLNLQFLLV   729
ENSBIXP00005030570_Bihy/1-1085        IGEPQDWTCRLRQPAFGISFVLCISCILVKTNRVLLVFEAKIPTSFHRKWWGLNLQFLLV   729
ENSMUGP00000006365_Mung/1-1080        IGEPQDWTCRLRQPAFGISFVLCISCILVKTNRVLLVFEAKIPTSFHRKWWGLNLQFLLV   728
ENSRNOP00000069629_Rnor/1-1079        IGEPQDWTCRLRQPAFGISFVLCISCILVKTNRVLLVFEAKIPTSFHRKWWGLNLQFLLV   728
MGP_PahariEiJ_P0028852_Mpah/1-1003    IGEPQDWTCRLRQPAFGISFVLCISCILVKTNRVLLVFEAKIPTSFHRKWWGLNLQFLLV   651
MGP_SPRETEiJ_P0043347_Mspr/1-1079     IGEPQDWTCRLRQPAFGISFVLCISCILVKTNRVLLVFEAKIPTSFHRKWWGLNLQFLLV   728
ENSMUSP00000069080_Mmus/1-1079        IGEPQDWTCRLRQPAFGISFVLCISCILVKTNRVLLVFEAKIPTSFHRKWWGLNLQFLLV   728
ENSMOCP00000010340_Moch/1-1076        IGEPQDWTCRLRQPAFGISFVLCISCILVKTNRVLLVFEAKIPTSFHRKWWGLNLQFLLV   728
ENSPEMP00000029995_Pmba/1-1076        IGEPQDWTCRLRQPAFGISFVLCISCILVKTNRVLLVFEAKIPTSFHRKWWGLNLQFLLV   728
ENSCGRP00015024036_Cgpi/1-1081        IGEPQDWTCRLRQPAFGISFVLCISCILVKTNRVLLVFEAKIPTSFHRKWWGLNLQFLLV   728
ENSCGRP00001016053_Cgch/1-1081        IGEPQDWTCRLRQPAFGISFVLCISCILVKTNRVLLVFEAKIPTSFHRKWWGLNLQFLLV   728
ENSCGRP00000017369_Cgcr/1-1081        IGEPQDWTCRLRQPAFGISFVLCISCILVKTNRVLLVFEAKIPTSFHRKWWGLNLQFLLV   728
ENSDORP00000002959_Dord/1-1078        IGEPQDWTCRLRQPAFGISFVLCISCILVKTNRVLLVFEAKIPTSFHRKWWGLNLQFLLV   728
ENSTSYP00000002643_Csyr/1-1015        IGEPQDWTCRLRQPAFGISFVLCISCILVKTNRVLLVFEAKIPTSLHRKWWGLNLQFLLV   738
ENSMLUP00000008249_Mluc/1-1075        IGEPQDWTCRLRQPAFGISFVLCISCILVKTNRVLLVFEAKIPTSFHRKWWGLNLQFLLV   727
ENSECAP00000022285_Ecab/1-1084        IGEPQDWTCRLRQPAFGISFVLCISCILVKTNRVLLVFEAKIPTSFHRKWWGLNLQFLLV   728
ENSEASP00005022676_Eaas/1-1094        IGEPQDWTCRLRQPAFGISFVLCISCILVKTNRVLLVFEAKIPTSFHRKWWGLNLQFLLV   738
ENSCAFP00000017381_Cfam/1-1075        IGEPQDWTCRLRQPAFGISFVLCISCILVKTNRVLLVFEAKIPTSFHRKWWGLNLQFLLV   728
ENSCAFP00020007154_Cldi/1-1075        IGEPQDWTCRLRQPAFGISFVLCISCILVKTNRVLLVFEAKIPTSFHRKWWGLNLQFLLV   728
ENSVVUP00000030826_Vvul/1-1075        IGEPQDWTCRLRQPAFGISFVLCISCILVKTNRVLLVFEAKIPTSFHRKWWGLNLQFLLV   728
ENSFCAP00000008080_Fcat/1-1081        IGEPQDWTCRLRQPAFGISFVLCISCILVKTNRVLLVFEAKIPTSFHRKWWGLNLQFLLV   728
ENSPPRP00000023800_Ppar/1-1081        IGEPQDWTCRLRQPAFGISFVLCISCILVKTNRVLLVFEAKIPTSFHRKWWGLNLQFLLV   728
ENSPTIP00000015042_Ptal/1-1081        IGEPQDWTCRLRQPAFGISFVLCISCILVKTNRVLLVFEAKIPTSFHRKWWGLNLQFLLV   728
ENSMPUP00000004759_Mpfu/1-1082        IGEPQDWTCRLRQPAFGISFVLCISCILVKTNRVLLVFEAKIPTSFHRKWWGLNLQFLLV   728
ENSNVIP00000018564_Nvis/1-1092        IGEPQDWTCRLRQPAFGISFVLCISCILVKTNRVLLVFEAKIPTSFHRKWWGLNLQFLLV   738
ENSUMAP00000035085_Umar/1-1071        IGEPQDWTCRLRQPAFGISFVLCISCILVKTNRVLLVFEAKIPTSFHRKWWGLNLQFLLV   738
ENSUAMP00000035930_Uame/1-1079        IGEPQDWTCRLRQPAFGISFVLCISCILVKTNRVLLVFEAKIPTSFHRKWWGLNLQFLLV   728
ENSAMEP00000015060_Amel/1-1089        IGEPQDWTCRLRQPAFGISFVLCISCILVKTNRVLLVFEAKIPTSFHRKWWGLNLQFLLV   738
ENSSDAP00000007437_Sdau/1-1079        IGEPQDWTCRLRQPAFGISFVLCISCILVKTNRVLLVFEAKIPTSFHRKWWGLNLQFLLV   728
ENSSTOP00000003945_Itri/1-1070        IGEPQDWTCRLRQPAFGISFVLCISCILVKTNRVLLVFEAKIPTSFHRKWWGLNLQFLLV   728
ENSMMMP00000015298_Mmma/1-1083        IGEPQDWTCRLRQPAFGISFVLCISCILVKTNRVLLVFEAKIPTSFHRKWWGLNLQFLLV   728
ENSUPAP00010025947_Upar/1-1086        IGEPQDWTCRLRQPAFGISFVLCISCILVKTNRVLLVFEAKIPTSFHRKWWGLNLQFLLV   728
ENSSBOP00000036416_Sbbo/1-1078        IGEPQDWTCRLRQPAFGISFVLCISCILVKTNRVLLVFEAKIPTSFHRKWWGLNLQFLLV   728
ENSANAP00000028404_Anan/1-1078        IGEPQDWTCRLRQPAFGISFVLCISCILVKTNRVLLVFEAKIPTSFHRKWWGLNLQFLLV   728
ENSCCAP00000024327_Ccap/1-1077        IGEPQDWTCRLRQPAFGISFVLCISCILVKTNRVLLVFEAKIPTSFHRKWWGLNLQFLLV   728
ENSCJAP00000062854_Cjac/1-1089        IGEPQDWTCRLRQPAFGISFVLCISCILVKTNRVLLVFEAKIPTSFHRKWWGLNLQFLLV   738
ENSNLEP00000000336_Nleu/1-1088        IGEPQDWTCRLRQPAFGISFVLCISCILVKTNRVLLVFEAKIPTSFHRKWWGLNLQFLLV   738
ENSPPYP00000015087_Pabe/1-1078        IGEPQDWTCRLRQPAFGISFVLCISCILVKTNRVVLVFEAKIPTSFHRKWWGLNLQFLLV   728
ENSP00000420194_Hsap/1-1088           IGEPQDWTCRLRQPAFGISFVLCISCILVKTNRVLLVFEAKIPTSFHRKWWGLNLQFLLV   738
ENSGGOP00000020287_Ggor/1-1078        IGEPQDWTCRLRQPAFGISFVLCISCILVKTNRVLLVFEAKIPTSFHRKWWGLNLQFLLV   728
ENSPPAP00000026371_Ppan/1-1078        IGEPQDWTCRLRQPAFGISFVLCISCILVKTNRVLLVFEAKIPTSFHRKWWGLNLQFLLV   728
ENSPTRP00000043342_Ptro/1-1078        IGEPQDWTCRLRQPAFGISFVLCISCILVKTNRVLLVFEAKIPTSFHRKWWGLNLQFLLV   728
ENSCSAP00000003635_Csab/1-882         IGEPQDWTCRLRQPAFGISFVLCISCILVKTNRVLLVFEAKIPTSFHRKWWGLNLQFLLV   532
ENSMMUP00000061002_Mmul/1-1078        IGEPQDWTCRLRQPAFGISFVLCISCILVKTNRVLLVFEAKIPTSFHRKWWGLNLQFLLV   728
ENSPANP00000004316_Panu/1-1081        IGEPQDWTCRLRQPAFGISFVLCISCILVKTNRVLLVFEAKIPTSFHRKWWGLNLQFLLV   731
ENSCATP00000028615_Caty/1-1078        IGEPQDWTCRLRQPAFGISFVLCISCILVKTNRVLLVFEAKIPTSFHRKWWGLNLQFLLV   728
ENSMFAP00000042132_Mfas/1-1078        IGEPQDWTCRLRQPAFGISFVLCISCILVKTNRVLLVFEAKIPTSFHRKWWGLNLQFLLV   728
ENSMNEP00000024602_Mnem/1-1078        IGEPQDWTCRLRQPAFGISFVLCISCILVKTNRVLLVFEAKIPTSFHRKWWGLNLQFLLV   728
ENSMLEP00000008744_Mleu/1-1088        IGEPQDWTCRLRQPAFGISFVLCISCILVKTNRVLLVFEAKIPTSFHRKWWGLNLQFLLV   738
ENSTGEP00000008766_Tgel/1-1088        IGEPQDWTCRLRQPAFGISFVLCISCILVKTNRVLLVFEAKIPTSFHRKWWGLNLQFLLV   738
ENSCANP00000040262_Capa/1-1088        IGEPQDWTCRLRQPAFGISFVLCISCILVKTNRVLLVFEAKIPTSFHHKWWGLNLQFLLV   738
ENSPTEP00000016080_Ptep/1-1088        IGEPQDWTCRLRQPAFGISFVLCISCILVKTNRVLLVFEAKIPTSFHRKWWGLNLQFLLV   738
ENSRBIP00000006831_Rbie/1-1078        IGEPQDWTCRLRQPAFGISFVLCISCILVKTNRVLLVFEAKIPTSFHRKWWGLNLQFLLV   728
ENSRROP00000007818_Rrox/1-1088        IGEPQDWTCRLRQPAFGISFVLCISCILVKTNRVLLVFEAKIPTSFHRKWWGLNLQFLLV   738
ENSOGAP00000002850_Ogar/1-1077        IGEPQDWTCRLRQPAFGISFVLCISCILVKTNRVLLVFEAKIPTSFHRKWWGLNLQFLLV   728
ENSMICP00000050654_Mmur/1-1075        IGEPQDWTCRLRQPAFGISFVLCISCILVKTNRVLLVFEAKIPTSFHRKWWGLNLQFLLV   728
ENSPCOP00000009624_Pcoq/1-1076        IGEPQDWTCRLRQPAFGISFVLCISCILVKTNRVLLVFEAKIPTSFHRKWWGLNLQFLLV   728
ENSPSMP00000007909_Psim/1-1084        IGEPQDWTCRLRQPAFGISFVLCISCILVKTNRVLLVFEAKIPTSFHRKWWGLNLQFLLV   738
                                      **** :*.* ******* *****:********.::*.*******.:*:**********:*

ENSMEUP00000013310_Neug/1-956         FLCTFMQIVICVIWLYNAPPSSYRNYELEDEIIFVTCHEGSLMALGFLIGYTCLLAAICF   734
ENSTBEP00000002487_Tbel/1-914         FLCTFMQIVICVIWLYTAPPSSYRNHELEDEIIFITCHEGSLMALGFLIGYTCLLAAICF   633
ENSPCAP00000000755_Pcap/1-1085        FLCTFMQIVICVIWLYTAPPSSYRNHELEDEIIFITCHEGSLMALGFLIGYTCLLAAVCF   792
ENSCHOP00000011907_Chof/1-954         FLCTFMQIVICVIWLHTAPPSSYRNHELEDEIIFITCHEGSLMALGFLIGYTCLLAAICF   797
ENSVPAP00000001982_Vpac/1-1087        FLCTFMQIVICAIWLYTAPPSSYRNQELEDEIIFITCHEGSLMALGFLIGYTCLLAAICF   797
ENSEEUP00000009670_Eeur/1-946         FLCTFMQIVICVIWLYTSPPSSYRNHELEDEIIFITCHEGSLMALGFLIGYTCLLAAICF   662
ENSOANP00000015961_Oana/1-1083        FLCTLVQVVICAIWLYTAPPSSYRNHELEDEIIFITCHEGSLMALGFLIGYTCLLAAVCF   788
ENSOPRP00000000261_Opri/1-1082        FLCTFMQIVICGIWLYTAPPSSYRNHELEDEIIFITCHEGSLMALGFLIGYTCLLAAVCF   798
ENSJJAP00000004721_Jjac/1-1084        FLCTFMQIVICAIWLYTAPPSSYRNHELEDEIIFITCHEGSLMALGSLIGYTCLLAAICF   764
ENSPCIP00000034747_Pcin/1-1018        FLCTFMQIVICVIWLYNAPPSSYRNYELEDEIIFVTCHEGSLMALGFLIGYTCLLAAICF   736
ENSMODP00000022810_Mdom/1-1069        FLCTFMQIVICVIWLYNAPPSSYRNYELEDEIIFITCHEGSLMALGFLIGYTCLLAAICF   789
ENSVURP00010007344_Vurs/1-1070        FLCTFMQIVICVIWLYNAPPSSYRNYELEDEIIFVTCHEGSLMALGFLIGYTCLLAAICF   789
ENSSHAP00000014890_Shar/1-1072        FLCTFMQIVICVIWLYNAPPSSYRNYELEDEIIFVTCHEGSLMALGFLIGYTCLLAAICF   789
ENSMAUP00000005394_Maur/1-954         FLCTFMQIVICVIWLYTAPPSSYRNHELEDEIIFITCHEGSLMALGSLIGYTCLLAAICF   737
ENSOCUP00000025672_Ocun/1-1063        FLCTFMQIVICGIWLYTAPPSSYRNHELEDEIIFITCHEGSLMALGFLIGYTCLLAAICF   788
```

ENSPVAP00000011981_Pvam/1-1085 FLCF-MQIVIXXXXXXXXXXXXXXXXXXXXXXXXXXXXXXXXXXXXXXXXXXXXXXXXXX 796
ENSETEP00000011160_Etel/1-1024 FLCTFMQIVICVIWLYTAPPSSYRNHELEDEIIFITCHEGSLMALGFLIGYTCLLAAICF 734
ENSFDAP00000010532_Fdam/1-1108 FLCTFMQIVICVIWLYTAPPSSYRNHELEDEIIFITCHEGSLMALGFLIGYTCLLAAICF 816
ENSODEP00000013257_Odeg/1-1070 FLCTFMQIVICAIWLYTAPPSSYRNHELEDEIIFITCHEGSLMALGFLIGYTCLLAAICF 788
ENSHGLP00000002019_Hgfe/1-1108 FLCTFMQIVICVIWLYTAPPSSYRNHELEDEIIFITCHEGSLMALGFLIGYTCLLAAICF 816
ENSCLAP00000021334_Clan/1-1114 FLCTFMQIVICVIWLYTAPPSSYRNHELEDEIIFITCHEGSLMALGFLIGYTCLLAAICF 816
ENSCAPP00000014352_Cape/1-1068 FLCTFMQIVICAIWLYTAPPSSYRNHELEDEIIFITCHEGSLMALGFLIGYTCLLAAICF 788
ENSCPOP00000011705_Cpor/1-1071 FLCTFMQIVICAIWLYTAPPSSYRNHELEDEIIFITCHEGSLMALGFLIGYTCLLAAICF 788
ENSSARP00000006151_Sara/1-1085 FLCTFMQIVICVIWLYTAPPSSYRNHELEDEIIFITCHEGSLMALGFLIGYTCLLAAICF 795
ENSLAFP00000018459_Lafr/1-1090 FLCTFMQIAICVIWLYTAPPSSYRNHELEDEIIFITCHEGSLMALGFLIGYTCLLAAICF 799
ENSDNOP00000011819_Dnov/1-1075 FLCTFMQIVICVIWLYTAPPSSYRNHELEDEIIFITCHEGSLMALGFLIGYTCLLAAVCF 788
ENSSSCP00000041077_Sscr/1-1089 FLCTFMQIVICAIWLYTAPPSSYRNHELEDEIIFITCHEGSLMALGFLIGYTCLLAAICF 798
ENSTTRP00000001326_Ttru/1-1091 FLCTFMQIVICAIWLNTAPPSSYRNHELEDEIIFITCHEGSLMALGFLIGYTCLLAAICF 798
ENSBBBP00000002858_Bbbi/1-1068 FLCTFMQIVICAIWLNTAPPSSYRNHELEDEIIFITCHEGSLMALGFLIGYTCLLAAICF 800
ENSOARP00000021602_Oari/1-1032 FLCTFMQIVICAIWLNTAPPSSYRNHELEDEIIFITCHEGSLMALGFLIGYTCLLAAICF 789
ENSCHIP00000033385_Chir/1-1085 FLCTFMQIVICAIWLNTAPPSSYRNHELEDEIIFITCHEGSLMALGFLIGYTCLLAAICF 789
ENSBTAP00000059078_Btau/1-1085 FLCTFMQIVICAIWLNTAPPSSYRNHELEDEIIFITCHEGSLMALGFLIGYTCLLAAICF 789
ENSBIXP00000038534_Bthy/1-1085 FLCTFMQIVICAIWLNTAPPSSYRNHELEDEIIFITCHEGSLMALGFLIGYTCLLAAICF 789
ENSBMUP00000019115_Bmut/1-1081 FLCTFMQIVICAIWLNTAPPSSYRNHELEDEIIFITCHEGSLMALGFLIGYTCLLAAICF 789
ENSBIXP00005030570_Bihy/1-1085 FLCTFMQIVICAIWLNTAPPSSYRNHELEDEIIFITCHEGSLMALGFLIGYTCLLAAICF 789
ENSMUGP00000006365_Mung/1-1080 FLCTFMQIVICVIWLYTAPPSSYRNHELEDEIIFITCHEGSLMALGSLIGYTCLLAAICF 788
ENSRNOP00000069629_Rnor/1-1079 FLCTFMQILICIIWLYTAPPSSYRNHELEDEIIFITCHEGSLMALGSLIGYTCLLAAICF 788
MGP_PahariEiJ_P0028852_Mpah/1-1003 FLCTFMQIVICIIWLYTAPPSSYRNHELEDEIIFITCHEGSLMALGSLIGYTCLLAAICF 711
MGP_SPRETEiJ_P0043347_Mspr/1-1079 FLCTFMQIVICIIWLYTAPPSSYRNHELEDEIIFITCHEGSLMALGSLIGYTCLLAAICF 788
ENSMUSP00000069080_Mmus/1-1079 FLCTFMQIVICIIWLYTAPPSSYRNHELEDEIIFITCHEGSLMALGSLIGYTCLLAAICF 788
ENSMOCP00000010340_Moch/1-1076 FLCTFMQIVICVIWLYTAPPSSYRNHELEDEIIFITCHEGSLMALGSLIGYTCLLAAICF 788
ENSPEMP00000029995_Pmba/1-1076 FLCTFMQIVICVIWLYTAPPSSYRNHELEDEIIFITCHEGSLMALGSLIGYTCLLAAICF 788
ENSCGRP00015024036_Cgpi/1-1081 FLCTFMQIVICVIWLYTAPPSSYRNHELEDEIIFITCHEGSLMALGSLIGYTCLLAAICF 788
ENSCGRP00001016053_Cgch/1-1081 FLCTFMQIVICVIWLYTAPPSSYRNHELEDEIIFITCHEGSLMALGSLIGYTCLLAAICF 788
ENSCGRP00000017369_Cgcr/1-1081 FLCTFMQIVICVIWLYTAPPSSYRNHELEDEIIFITCHEGSLMALGSLIGYTCLLAAICF 788
ENSDORP00000002959_Dord/1-1078 FLCTFMQIVICVIWLYTAPPSSYRNHELEDEIIFITCHEGSLMALGSLIGYTCLLAAICF 788
ENSTSYP00000002643_Csyr/1-1015 FLCTFMQIVICVIWLYTAPPSSYRNHELEDEVIFITCHEGSLMALGFLIGYTCLLAAVCF 798
ENSMLUP00000008249_Mluc/1-1075 FLCTFVQIVICVIWLYTAPPSSYRNHELEDEIIFITCHEGSLMALGFLIGYTCLLAAICF 787
ENSECAP00000022285_Ecab/1-1084 FLCTFMQIVICVIWLYTAPPSSYRNHELEDEIIFITCHEGSLMALGFLIGYTCLLAAICF 788
ENSEASP00005022676_Eaas/1-1094 FLCTFMQIVICVIWLYTAPPSSYRNHELEDEIIFITCHEGSLMALGFLIGYTCLLAAICF 798
ENSCAFP00000017381_Cfam/1-1075 FLCTFMQIVICVIWLYTAPPSSYRNHELEDEIIFITCHEGSLMALGFLIGYTCLLAAICF 788
ENSCAFP00020007154_Cldi/1-1075 FLCTFMQIVICVIWLYTAPPSSYRNHELEDEIIFITCHEGSLMALGFLIGYTCLLAAICF 788
ENSVVUP00000030826_Vvul/1-1075 FLCTFMQIVICVIWLYTAPPSSYRNHELEDEIIFITCHEGSLMALGFLIGYTCLLAAICF 788
ENSFCAP00000008080_Fcat/1-1081 FLCTFMQIVICVIWLYTAPPSSYRNHELEDEIIFITCHEGSLMALGFLIGYTCLLAAICF 788
ENSPPRP00000023800_Ppar/1-1081 FLCTFMQIVICVIWLYTAPPSSYRNHELEDEIIFITCHEGSLMALGFLIGYTCLLAAICF 788
ENSPTIP00000015042_Ptal/1-1081 FLCTFMQIVICVIWLYTAPPSSYRNHELEDEIIFITCHEGSLMALGFLIGYTCLLAAICF 788
ENSMPUP00000004759_Mpfu/1-1082 FLCTFMQIVICVIWLYTAPPSSYRNHELEDEIIFITCHEGSLMALGFLIGYTCLLAAICF 788
ENSNVIP00000018564_Nvis/1-1092 FLCTFMQIVICVIWLYTAPPSSYRNHELEDEIIFITCHEGSLMALGFLIGYTCLLAAICF 798
ENSUMAP00000035085_Umar/1-1071 FLCTFMQIVICVIWLYTAPPSSYRNHELEDEIIFITCHEGSLMALGFLIGYTCLLAAICF 798
ENSUAMP00000035930_Uame/1-1079 FLCTFMQIVICVIWLYTAPPSSYRNHELEDEIIFITCHEGSLMALGFLIGYTCLLAAICF 788
ENSAMEP00000015060_Amel/1-1089 FLCTFMQIVICVIWLYTAPPSSYRNHELEDEIIFITCHEGSLMALGFLIGYTCLLAAICF 798
ENSSDAP00000007437_Sdau/1-1079 FLCTFMQIVICVIWLYTAPPSSYRNHELEDEIIFITCHEGSLMALGFLIGYTCLLAAICF 788
ENSSTOP00000003945_Itri/1-1070 FLCTFMQIVICAIWLYTAPPSSYRNHELEDEIIFITCHEGSLMALGFLIGYTCLLAAICF 788
ENSMMMP00000015298_Mmma/1-1083 FLCTFMQIVICVIWLYTAPPSSYRNHELEDEIIFITCHEGSLMALGFLIGYTCLLAAICF 788
ENSUPAP00010025947_Upar/1-1086 FLCTFMQIVICVIWLYTAPPSSYRNHELEDEIIFITCHEGSLMALGFLIGYTCLLAAICF 788
ENSSBOP00000036416_Sbbo/1-1078 FLCTFMQIVICVIWLYTAPPSSYRNHELEDEIIFITCHEGSLMALGFLIGYTCLLAAICF 788
ENSANAP00000028404_Anan/1-1078 FLCTFMQIVICVIWLYTAPPSSYRNHELEDEIIFITCHEGSLMALGFLIGYTCLLAAICF 788
ENSCCAP00000024327_Ccap/1-1077 FLCTFMQIVICVIWLYTAPPSSYRNHELEDEIIFITCHEGSLMALGFLIGYTCLLAAICF 788
ENSCJAP00000062854_Cjac/1-1089 FLCTFMQIVICVIWLYTAPPSSYRNHELEDEIIFITCHEGSLMALGFLIGYTCLLAAICF 798
ENSNLEP00000000336_Nleu/1-1088 FLCTFMQIVICVIWLYTAPPSSYRNHELEDEIIFITCHEGSLMALGFLIGYTCLLAAICF 798
ENSPPYP00000015087_Pabe/1-1078 FLCTFMQIVICVIWLYTAPPSSYRNHELEDEIIFITCHEGSLMALGFLIGYTCLLAAICF 788
ENSP00000420194_Hsap/1-1088 FLCTFMQIVICVIWLYTAPPSSYRNQELEDEIIFITCHEGSLMALGFLIGYTCLLAAICF 798
ENSGGOP00000020287_Ggor/1-1078 FLCTFMQIVICVIWLYTAPPSSYRNHELEDEIIFITCHEGSLMALGFLIGYTCLLAAICF 788
ENSPPAP00000026371_Ppan/1-1078 FLCTFMQIVICVIWLYTAPPSSYRNHELEDEIIFITCHEGSLMALGFLIGYTCLLAAVCF 788
ENSPTRP00000043342_Ptro/1-1078 FLCTFMQIVICVIWLYTAPPSSYRNHELEDEIIFITCHEGSLMALGFLIGYTCLLAAVCF 788
ENSCSAP00000003635_Csab/1-882 FLCTFMQIVICVIWLYTAPPSSYRNHELEDEIIFITCHEGSLMALGFLIGYTCLLAAICF 592
ENSMMUP00000061002_Mmul/1-1078 FLCTFMQIVICVIWLYTAPPSSYRNHELEDEIIFITCHEGSLMALGFLIGYTCLLAAICF 788
ENSPANP00000004316_Panu/1-1081 FLCTFMQIVICVIWLYTAPPSSYRNHELEDEIIFITCHEGSLMALGFLIGYTCLLAAICF 791
ENSCATP00000028615_Caty/1-1078 FLCTFMQIVICVIWLYTAPPSSYRNHELEDEIIFITCHEGSLMALGFLIGYTCLLAAICF 788
ENSMFAP00000042132_Mfas/1-1078 FLCTFMQIVICVIWLYTAPPSSYRNHELEDEIIFITCHEGSLMALGFLIGYTCLLAAICF 788
ENSMNEP00000024602_Mnem/1-1078 FLCTFMQIVICVIWLYTAPPSSYRNHELEDEIIFITCHEGSLMALGFLIGYTCLLAAICF 788
ENSMLEP00000008744_Mleu/1-1088 FLCTFMQIVICVIWLYTAPPSSYRNHELEDEIIFITCHEGSLMALGFLIGYTCLLAAICF 798
ENSTGEP00000008766_Tgel/1-1088 FLCTFMQIVICVIWLYTAPPSSYRNHELEDEIIFITCHEGSLMALGFLIGYTCLLAAICF 798
ENSCANP00000040262_Capa/1-1088 FLCTFMQIVICVIWLYTAPPSSYRNHELEDEIIFITCHEGSLMALGFLIGYTCLLAAICF 798
ENSPTEP00000016080_Ptep/1-1088 FLCTFMQIVICVIWLYTAPPSSYRNHELEDEIIFITCHEGSLMALGFLIGYTCLLAAICF 798
ENSRBIP00000006831_Rbie/1-1078 FLCTFMQIVICVIWLYTAPPSSYRNHELEDEIIFITCHEGSLMALGFLIGYTCLLAAICF 788
ENSRROP00000007818_Rrox/1-1088 FLCTFMQIVICVIWLYTAPPSSYRNHELEDEIIFITCHEGSLMALGFLIGYTCLLAAICF 798
ENSOGAP00000002850_Ogar/1-1077 FLCTFMQIVICVIWLYTAPPSSYRNHELEDEIIFITCHEGSLMALGFLIGYTCLLAAICF 788
ENSMICP00000050654_Mmur/1-1075 FLCTFMQIVICVIWLYTAPPSSYRNHELEDEIIFITCHEGSLMALGFLIGYTCLLAAICF 788
ENSPCOP00000009624_Pcoq/1-1076 FLCTFMQIVICVIWLYTAPPSSYRNHELEDEIIFITCHEGSLMALGFLIGYTCLLAAICF 788
ENSPSMP00000007909_Psim/1-1084 FLCTFMQIVICVIWLYTAPPSSYRNHELEDEIIFITCHEGSLMALGFLIGYTCLLAAICF 798
*** :*: *

ENSMEUP00000013310_Neug/1-956 FFAFKSRKLPENFNEAKFITFSMLIFFIVWISFIPAYASTYGKFVSAVEVIAILAASFGL 794
ENSTBEP00000002487_Tbel/1-914 FFAFKSRKLPENFNEAKFITFSMLIFFIVWISFIPAYASTYGKFVSAVEVIAILAASFGL 693
ENSPCAP00000000755_Pcap/1-1085 FFAFKSRKLPENFNEAKFITFSMLIFFIVWISFIPAYASTYGKFVSAVEVIAILAASFGL 852
ENSCHOP00000011907_Chof/1-954 FFAFKSRKLPENFNEAKFITFSMLIFFIVWISFIPAYASTYGKFVSAVEVIAILAASFGL 857
ENSVPAP00000001982_Vpac/1-1087 FFAFKSRKLPENFNEAKFITFSMLIFFIVWISFIPAYASTYGKFVSAVEVIAILAASFGL 857
ENSEEUP00000009670_Eeur/1-946 FFAFKSRKLPENFNEAKFITFSMLIFFIVWISFIPAYASTYGKFVSAVEVIAILAASFGL 722
ENSOANP00000015961_Oana/1-1083 FFAFKSRKLPENFNEAKFITFSMLIFFIVWISFIPAYASTYGKFVSAVEVIAILAASFGL 848
ENSOPRP00000000261_Opri/1-1082 FFAFKSRKLPENFNEAKFITFSMLIFFIVWISFIPAYASTYGKFVSAVEVIAILAASFGL 858
ENSJJAP00000004721_Jjac/1-1084 FFAFKSRKLPENFNEAKFITFSMLIFFIVWISFIPAYASTYGKFVSAVEVIAILAASFGL 824
ENSPCIP00000034747_Pcin/1-1018 FFAFKSRKLPENFNEAKFITFSMLIFFIVWISFIPAYASTYGKFVSAVEVIAILAASFGL 796

```
ENSMODP00000022810_Mdom/1-1069        FFAFKSRKLPENFNEAKFITFSMLIFFIVWISFIPAYASTYGKFVSAVEVIAILAASFGL   849
ENSVURP00010007344_Vurs/1-1070        FFAFKSRKLPENFNEAKFITFSMLIFFIVWISFIPAYASTYGKFVSAVEVIAILAASFGL   849
ENSSHAP00000014890_Shar/1-1072        FFAFKSRKLPENFNEAKFITFSMLIFFIVWISFIPAYASTYGKFVSAVEVIAILAASFGL   849
ENSMAUP00000005394_Maur/1-954         FFAFKSRKLPENFNEAKFITFSMLIFFIVWISFIPAYASTYGKFVSAVEVIAILAASFGL   797
ENSOCUP00000025672_Ocun/1-1063        FFAFKSRKLPENFNEAKFITFSMLIFFIVWISFIPAYASTYGKFVSAVEVIAILAASFGL   848
ENSPVAP00000011981_Pvam/1-1085        FFAFKFRKL-ENFHEAKFI-FSMLIFFIVWISFIPPYAS-YGK-VSAVEVIAILAASFGL   852
ENSETEP00000011160_Etel/1-1024        FFAFKSRKLPENFNEAKFITFSMLIFFIVWISFIPAYASTYGKFVSAVEVIAILAASFGL   794
ENSFDAP00000010532_Fdam/1-1108        FFAFKSRKLPENFNEAKFITFSMLIFFIVWISFIPAYASTYGKFVSAVEVIAILAASFGL   876
ENSODEP00000013257_Odeg/1-1070        FFAFKSRKLPENFNEAKFITFSMLIFFIVWISFIPAYASTYGKFVSAVEVIAILAASFGL   848
ENSHGLP00000002019_Hgfe/1-1108        FFAFKSRKLPENFNEAKFITFSMLIFFIVWISFIPAYASTYGKFVSAVEVIAILAASFGL   876
ENSCLAP00000021334_Clan/1-1114        FFAFKSRKLPENFNEAKFITFSMLIFFIVWISFIPAYASTYGKFVSAVEVIAILAASFGL   876
ENSCAPP00000014352_Cape/1-1068        FFAFKSRKLPENFNEAKFITFSMLIFFIVWISFIPAYASTYGKFVSAVEVIAILAASFGL   848
ENSCPOP00000011705_Cpor/1-1071        FFAFKSRKLPENFNEAKFITFSMLIFFIVWISFIPAYASTYGKFVSAVEVIAILAASFGL   848
ENSSARP00000006151_Sara/1-1085        FFAFKSRKLPENFNEAKFITFSMLIFFIVWISFIPAYASTYGKFVSAVEVIAILAASFGL   855
ENSLAFP00000018459_Lafr/1-1090        FFAFKSRKLPENFNEAKFITFSMLIFFIVWISFIPAYASTYGKFVSAVEVIAILAASFGL   859
ENSDNOP00000011819_Dnov/1-1075        FFAFKSRKLPENFNEAKFITFSMLIFFIVWISFIPAYASTYGKFVSAVEVIAILAASFGL   848
ENSSSCP00000041077_Sscr/1-1089        FFAFKSRKLPENFNEAKFITFSMLIFFIVWISFIPAYASTYGKFVSAVEVIAILAASFGL   858
ENSTTRP00000001326_Ttru/1-1091        FFAFKSRKLPENFNEAKFITFSMLIFFIVWISFIPAYASTYGKFVSAVEVIAILAASFGL   858
ENSBBBP00000002858_Bbbi/1-1068        FFAFKSRKLPENFNEAKFITFSMLIFFIVWISFIPAYASTYGKFVSAVEVIAILA-----   855
ENSOARP00000021602_Oari/1-1032        FFAFKSRKLPENFNEAKFITFSMLIFFIVWISFIPAYASTYGKFVSAVEVIAILAASFGL   849
ENSCHIP00000033385_Chir/1-1085        FFAFKSRKLPENFNEAKFITFSMLIFFIVWISFIPAYASTYGKFVSAVEVIAILAASFGL   849
ENSBTAP00000059078_Btau/1-1085        FFAFKSRKLPENFNEAKFITFSMLIFFIVWISFIPAYASTYGKFVSAVEVIAILAASFGL   849
ENSBIXP00000038534_Bthy/1-1085        FFAFKSRKLPENFNEAKFITFSMLIFFIVWISFIPAYASTYGKFVSAVEVIAILAASFGL   849
ENSBMUP00000019115_Bmut/1-1081        FFAFKSRKLPENFNEAKFITFSMLIFFIVWISFIPAYASTYGKFVSAVEVIAILAASFGL   849
ENSBIXP00005030570_Bihy/1-1085        FFAFKSRKLPENFNEAKFITFSMLIFFIVWISFIPAYASTYGKFVSAVEVIAILAASFGL   849
ENSMUGP00000006365_Mung/1-1080        FFAFKSRKLPENFNEAKFITFSMLIFFIVWISFIPAYASTYGKFVSAVEVIAILAASFGL   848
ENSRNOP00000069629_Rnor/1-1079        FFAFKSRKLPENFNEAKFITFSMLIFFIVWISFIPAYASTYGKFVSAVEVIAILAASFGL   848
MGP_PahariEiJ_P0028852_Mpah/1-1003    FFAFKSRKLPENFNEAKFITFSMLIFFIVWISFIPAYASTYGKFVSAVEVIAILAASFGL   771
MGP_SPRETEiJ_P0043347_Mspr/1-1079     FFAFKSRKLPENFNEAKFITFSMLIFFIVWISFIPAYASTYGKFVSAVEVIAILAASFGL   848
ENSMUSP00000069080_Mmus/1-1079        FFAFKSRKLPENFNEAKFITFSMLIFFIVWISFIPAYASTYGKFVSAVEVIAILAASFGL   848
ENSMOCP00000010340_Moch/1-1076        FFAFKSRKLPENFNEAKFITFSMLIFFIVWISFIPAYASTYGKFVSAVEVIAILAASFGL   848
ENSPEMP00000029995_Pmba/1-1076        FFAFKSRKLPENFNEAKFITFSMLIFFIVWISFIPAYASTYGKFVSAVEVIAILAASFGL   848
ENSCGRP00015024036_Cgpi/1-1081        FFAFKSRKLPENFNEAKFITFSMLIFFIVWISFIPAYASTYGKFVSAVEVIAILAASFGL   848
ENSCGRP00001016053_Cgch/1-1081        FFAFKSRKLPENFNEAKFITFSMLIFFIVWISFIPAYASTYGKFVSAVEVIAILAASFGL   848
ENSCGRP00000017369_Cgcr/1-1081        FFAFKSRKLPENFNEAKFITFSMLIFFIVWISFIPAYASTYGKFVSAVEVIAILAASFGL   848
ENSDORP00000002959_Dord/1-1078        FFAFKSRKLPENFNEAKFITFSMLIFFIVWISFIPAYASTYGKFVSAVEVIAILAASFGL   848
ENSTSYP00000002643_Csyr/1-1015        FFAFKSRKLPENFNEAKFITFSMLIFFIVWISFIPAYASTYGKFVSAVEVIAILAASFGL   858
ENSMLUP00000008249_Mluc/1-1075        FFAFKSRKLPENFNEAKFITFSMLIFFIVWISFIPAYASTYGKFVSAVEVIAILAASFGL   847
ENSECAP00000022285_Ecab/1-1084        FFAFKSRKLPENFNEAKFITFSMLIFFIVWISFIPAYASTYGKFVSAVEVIAILAASFGL   848
ENSEASP00005022676_Eaas/1-1094        FFAFKSRKLPENFNEAKFITFSMLIFFIVWISFIPAYASTYGKFVSAVEVIAILAASFGL   858
ENSCAFP00000017381_Cfam/1-1075        FFAFKSRKLPENFNEAKFITFSMLIFFIVWISFIPAYASTYGKFVSAVEVIAILAASFGL   848
ENSCAFP00020007154_Cldi/1-1075        FFAFKSRKLPENFNEAKFITFSMLIFFIVWISFIPAYASTYGKFVSAVEVIAILAASFGL   848
ENSVVUP00000030826_Vvul/1-1075        FFAFKSRKLPENFNEAKFITFSMLIFFIVWISFIPAYASTYGKFVSAVEVIAILAASFGL   848
ENSFCAP00000008080_Fcat/1-1081        FFAFKSRKLPENFNEAKFITFSMLIFFIVWISFIPAYASTYGKFVSAVEVIAILAASFGL   848
ENSPPRP00000023800_Ppar/1-1081        FFAFKSRKLPENFNEAKFITFSMLIFFIVWISFIPAYASTYGKFVSAVEVIAILAASFGL   848
ENSPTIP00000015042_Ptal/1-1081        FFAFKSRKLPENFNEAKFITFSMLIFFIVWISFIPAYASTYGKFVSAVEVIAILAASFGL   848
ENSMPUP00000004759_Mpfu/1-1082        FFAFKSRKLPENFNEAKFITFSMLIFFIVWISFIPAYASTYGKFVSAVEVIAILAASFGL   848
ENSNVIP00000018564_Nvis/1-1092        FFAFKSRKLPENFNEAKFITFSMLIFFIVWISFIPAYASTYGKFVSAVEVIAILAASFGL   858
ENSUMAP00000035085_Umar/1-1071        FFAFKSRKLPENFNEAKFITFSMLIFFIVWISFIPAYASTYGKFVSAVEVIAILAASFGL   858
ENSUAMP00000035930_Uame/1-1079        FFAFKSRKLPENFNEAKFITFSMLIFFIVWISFIPAYASTYGKFVSAVEVIAILAASFGL   848
ENSAMEP00000015060_Amel/1-1089        FFAFKSRKLPENFNEAKFITFSMLIFFIVWISFIPAYASTYGKFVSAVEVIAILAASFGL   858
ENSSDAP00000007437_Sdau/1-1079        FFAFKSRKLPENFNEAKFITFSMLIFFIVWISFIPAYASTYGKFVSAVEVIAILAASFGL   848
ENSSTOP00000003945_Itri/1-1070        FFAFKSRKLPENFNEAKFITFSMLIFFIVWISFIPAYASTYGKFVSAVEVIAILAASFGL   848
ENSMMMP00000015298_Mmma/1-1083        FFAFKSRKLPENFNEAKFITFSMLIFFIVWISFIPAYASTYGKFVSAVEVIAILAASFGL   848
ENSUPAP00010025947_Upar/1-1086        FFAFKSRKLPENFNEAKFITFSMLIFFIVWISFIPAYASTYGKFVSAVEVIAILAASFGL   848
ENSSBOP00000036416_Sbbo/1-1078        FFAFKSRKLPENFNEAKFITFSMLIFFIVWISFIPAYASTYGKFVSAVEVIAILAASFGL   848
ENSANAP00000028404_Anan/1-1078        FFAFKSRKLPENFNEAKFITFSMLIFFIVWISFIPAYASTYGKFVSAVEVIAILAASFGL   848
ENSCCAP00000024327_Ccap/1-1077        FFAFKSRKLPENFNEAKFITFSMLIFFIVWISFIPAYASTYGKFVSAVEVIAILAASFGL   848
ENSCJAP00000062854_Cjac/1-1089        FFAFKSRKLPENFNEAKFITFSMLIFFIVWISFIPAYASTYGKFVSAVEVIAILAASFGL   858
ENSNLEP00000000336_Nleu/1-1088        FFAFKSRKLPENFNEAKFITFSMLIFFIVWISFIPAYASTYGKFVSAVEVIAILAASFGL   858
ENSPPYP00000015087_Pabe/1-1078        FFAFKSRKLPENFNEAKFITFSMLIFFIVWISFIPAYASTYGKFVSAVEVIAILAASFGL   848
ENSP00000420194_Hsap/1-1088           FFAFKSRKLPENFNEAKFITFSMLIFFIVWISFIPAYASTYGKFVSAVEVIAILAASFGL   858
ENSGGOP00000020287_Ggor/1-1078        FFAFKSRKLPENFNEAKFITFSMLIFFIVWISFIPAYASTYGKFVSAVEVIAILAASFGL   848
ENSPPAP00000026371_Ppan/1-1078        FFAFKSRKLPENFNEAKFITFSMLIFFIVWISFIPAYASTYGKFVSAVEVIAILAASFGL   848
ENSPTRP00000043342_Ptro/1-1078        FFAFKSRKLPENFNEAKFITFSMLIFFIVWISFIPAYASTYGKFVSAVEVIAILAASFGL   848
ENSCSAP00000003635_Csab/1-882         FFAFKSRKLPENFNEAKFITFSMLIFFIVWISFIPAYASTYGKFVSAVEVIAILAASFGL   652
ENSMMUP00000061002_Mmul/1-1078        FFAFKSRKLPENFNEAKFITFSMLIFFIVWISFIPAYASTYGKFVSAVEVIAILAASFGL   848
ENSPANP00000004316_Panu/1-1081        FFAFKSRKLPENFNEAKFITFSMLIFFIVWISFIPAYASTYGKFVSAVEVIAILAASFGL   851
ENSCATP00000028615_Caty/1-1078        FFAFKSRKLPENFNEAKFITFSMLIFFIVWISFIPAYASTYGKFVSAVEVIAILAASFGL   848
ENSMFAP00000042132_Mfas/1-1078        FFAFKSRKLPENFNEAKFITFSMLIFFIVWISFIPAYASTYGKFVSAVEVIAILAASFGL   848
ENSMNEP00000024602_Mnem/1-1078        FFAFKSRKLPENFNEAKFITFSMLIFFIVWISFIPAYASTYGKFVSAVEVIAILAASFGL   848
ENSMLEP00000008744_Mleu/1-1088        FFAFKSRKLPENFNEAKFITFSMLIFFIVWISFIPAYASTYGKFVSAVEVIAILAASFGL   858
ENSTGEP00000008766_Tgel/1-1088        FFAFKSRKLPENFNEAKFITFSMLIFFIVWISFIPAYASTYGKFVSAVEVIAILAASFGL   858
ENSCANP00000040262_Capa/1-1088        FFAFKSRKLPENFNEAKFITFSMLIFFIVWISFIPAYASTYGKFVSAVEVIAILAASFGL   858
ENSPTEP00000016080_Ptep/1-1088        FFAFKSRKLPENFNEAKFITFSMLIFFIVWISFIPAYASTYGKFVSAVEVIAILAASFGL   858
ENSRBIP00000006831_Rbie/1-1078        FFAFKSRKLPENFNEAKFITFSMLIFFIVWISFIPAYASTYGKFVSAVEVIAILAASFGL   848
ENSRROP00000007818_Rrox/1-1088        FFAFKSRKLPENFNEAKFITFSMLIFFIVWISFIPAYASTYGKFVSAVEVIAILAASFGL   858
ENSOGAP00000002850_Ogar/1-1077        FFAFKSRKLPENFNEAKFITFSMLIFFIVWISFIPAYASTYGKFVSAVEVIAILAASFGL   848
ENSMICP00000050654_Mmur/1-1075        FFAFKSRKLPENFNEAKFITFSMLIFFIVWISFIPAYASTYGKFVSAVEVIAILAASFGL   848
ENSPCOP00000009624_Pcoq/1-1076        FFAFKSRKLPENFNEAKFITFSMLIFFIVWISFIPAYASTYGKFVSAVEVIAILAASFGL   848
ENSPSMP00000007909_Psim/1-1084        FFAFKSRKLPENFNEAKFITFSMLIFFIVWISFIPAYASTYGKFVSAVEVIAILAASFGL   858
                                      ***** *** ***:***** ************** *** *** ***********

ENSMEUP00000013310_Neug/1-956         LACIFFNKVYIILFKPSRNTIEEVRCSTAAHAFKVAARATLRRSNVSRKRSNSLGGSTGS   854
ENSTBEP00000002487_Tbel/1-914         LACIFFNKVYIILFKPARNTIEEVRCSTAAHAFKVAARATLRRSNVSRKRSSSLGGSTGS   753
ENSPCAP00000000755_Pcap/1-1085        LACIFFNKVYIILFKPSRNTIEEVRCSTAAHAFKVAARATLRRSNASRKRSSSLGGSAGS   912
ENSCHOP00000011907_Chof/1-954         LACIFFNKVYIILFKPSRNTIEEVRCSTAAHAFKVAARATLRRSNVSRKRSGSLGGSMGS   917
ENSVPAP00000001982_Vpac/1-1087        LACIFFNKVYIILFKPSRNTIEEVRCSTAAHAFKVAARATLRRSNVSRQRSSSLGGSTGS   917
```

```
ENSEEUP00000009670_Eeur/1-946           LACIFFNKVYIILFKPSRNTIEEVRCSTAAHAFKVAARATLRRSNVSRKRSNSLGGSTGS   782
ENSOANP00000015961_Oana/1-1083          LACIFFNKVYIILFKPARNTIEEVRCSTAAHAFKVAARATLRRSNLSRRRAGSLGGSSGS   908
ENSOPRP00000000261_Opri/1-1082          LACIFFNKVYIILVKPSRNTIEEVRCSTAAHAFKVAARATLRRSNVSRKRSSSLGGSTGS   918
ENSJJAP00000004721_Jjac/1-1084          LACIFFNKVYIILFKPARNTIEEVRCSTAAHAFKVAARATLRRSNVSRKRSSSLGGSTGS   884
ENSPCIP00000034747_Pcin/1-1018          LACIFFNKVYIILFKPSRNTIEEVRCSTAAHAFKVAARATLRRSNVSRKRSNSLGGSTGS   856
ENSMODP00000022810_Mdom/1-1069          LACIFFNKVYIILFKPSRNTIEEVRCSTAAHAFKVAARATLRRSNVSRKRSNSLGGSTGS   909
ENSVURP00010007344_Vurs/1-1070          LACIFFNKVYIILFKPSRNTIEEVRCSTAAHAFKVAARATLRRSNVSRKRSNSLGGSTGS   909
ENSSHAP00000014890_Shar/1-1072          LACIFFNKVYIILFKPSRNTIEEVRCSTAAHAFKVAARATLRRSNVSRKRSNSLGGSTGS   909
ENSMAUP00000005394_Maur/1-954           LACIFFNKVYIILFKPSRNTIEEVRCSTAAHAFKVAARATLRRSNVSRKRSSSLGGSTGS   857
ENSOCUP00000025672_Ocun/1-1063          LACIFFNKVYIILFKPSRNTIEEVRCSTAAHAFKVAARATLRRSNVSRKRSSSLGGSTGS   908
ENSPVAP00000011981_Pvam/1-1085          LACIFFNKVYIILFKPSRNTIEEVRCSTAAHAFKVAARATLRRSNISRKRSSSLGGSTGS   912
ENSETEP00000011160_Etel/1-1024          LACIFFNKVYIILFKPSRNTIEEVRCSTAAHAFKVAARATLRRSNVSRKRSNSLGGSTGS   854
ENSFDAP00000010532_Fdam/1-1108          LACIFFNKVYIILFKPSRNTIEEVRCSTAAHAFKVAARATLRRSNVSRKRSSSLGGSTGS   936
ENSODEP00000013257_Odeg/1-1070          LACIFFNKVYIILFKPSRNTIEEVRCSTAAHAFKVAARATLRRSNVSRKRSSSLGGSTGS   908
ENSHGLP00000002019_Hgfe/1-1108          LACIFFNKVYIILFKPSRNTIEEVRCSTAAHAFKVAARATLRRSNVSRKRSSSLGGSTGS   936
ENSCLAP00000021334_Clan/1-1114          LACIFFNKVYIILFKPSRNTIEEVRCSTAAHAFKVAARATLRRSNVSRKRSSSLGGSTGS   936
ENSCAPP00000014352_Cape/1-1068          LACIFFNKVYIILFKPSRNTIEEVRCSTAAHAFKVAARATLRRSNVSRKRSSSLGGSTGS   908
ENSCPOP00000011705_Cpor/1-1071          LACIFFNKVYIILFKPSRNTIEEVRCSTAAHAFKVAARATLGRSNVS----PAYGGSTGS   904
ENSSARP00000006151_Sara/1-1085          LACIFFNKVYIILFKPSRNTIEEVRCSTAAHAFKVAARATLRRSNVSRKRSSSLGGSTGS   915
ENSLAFP00000018459_Lafr/1-1090          LACIFFNKVYIILFKPSRNTIEEVRCSTAAHAFKVAARATLRRSNVSRKRSSSLGGSTGS   919
ENSDNOP00000011819_Dnov/1-1075          LACIFFNKVYIILFKPSRNTIEEVRCSTAAHAFKVAARATLRRSNVSRQRSSSLGGSVGS   908
ENSSSCP00000041077_Sscr/1-1089          LACIFFNKVYIILFKPSRNTIEEVRCSTAAHAFKVAARATLRRSNVSRQRSSSLGGSTGS   918
ENSTTRP00000001326_Ttru/1-1091          LACIFFNKVYIILFKPSRNTIEEVRCSTAAHAFKVAARATLRRSNVSRQRSSSLGGSTGS   918
ENSBBBP00000002858_Bbbi/1-1068          --------VYTILFKPSRNTIEEVRCSTAAHAFKVAARATLRRSNVSRQRSSSLGGSTGS   907
ENSOARP00000021602_Oari/1-1032          LACIFFNKVYIILFKPSRNTIEEVRCSTAAHAFKVAARATLRRSNVSRQRSSSLGGSTGS   909
ENSCHIP00000033385_Chir/1-1085          LACIFFNKVYIILFKPSRNTIEEVRCSTAAHAFKVAARATLRRSNVSRQRSSSLGGSTGS   909
ENSBTAP00000059078_Btau/1-1085          LACIFFNKVYIILFKPSRNTIEEVRCSTAAHAFKVAARATLRRSNVSRQRSSSLGGSTGS   909
ENSBIXP00000038534_Bthy/1-1085          LACIFFNKVYIILFKPSRNTIEEVRCSTAAHAFKVAARATLRRSNVSRQRSSSLGGSTGS   909
ENSBMUP00000019115_Bmut/1-1081          LACIFFNKVYIILFKPSRNTIEEVRCSTAAHAFKVAARATLRRSNVSRQRSSSLGGSTGS   909
ENSBIXP00005030570_Bihy/1-1085          LACIFFNKVYIILFKPSRNTIEEVRCSTAAHAFKVAARATLRRSNVSRQRSSSLGGSTGS   909
ENSMUGP00000006365_Mung/1-1080          LACIFFNKVYIILFKPSRNTIEEVRCSTAAHAFKVAARATLRRSNVSRKRSSSLGGSTGS   908
ENSRNOP00000069629_Rnor/1-1079          LACIFFNKVYIILFKPSRNTIEEVRSSTAAHAFKVAARATLRRPNISRKRSSSLGGSTGS   908
MGP_PahariEiJ_P0028852_Mpah/1-1003      LACIFFNKVYIILFKPSRNTIEEVRSSTAAHAFKVAARATLRRPNISRKRSSSLGGSTGS   831
MGP_SPRETEiJ_P0043347_Mspr/1-1079       LACIFFNKVYIILFKPSRNTIEEVRSSTAAHAFKVAARATLRRPNISRKRSSSLGGSTGS   908
ENSMUSP00000069080_Mmus/1-1079          LACIFFNKVYIILFKPSRNTIEEVRSSTAAHAFKVAARATLRRPNISRKRSSSLGGSTGS   908
ENSMOCP00000010340_Moch/1-1076          LACIFFNKVYIILFKPSRNTIEEVRCSTAAHAFKVAARATLRRSNASRKRSSSLGGSTGS   908
ENSPEMP00000029995_Pmba/1-1076          LACIFFNKVYIILFKPSRNTIEEVRCSTAAHAFKVAARATLRRSNVSRKRSSSLGGSTGS   908
ENSCGRP00015024036_Cgpi/1-1081          LACIFFNKVYIILFKPSRNTIEEVRCSTAAHAFKVAARATLRRSNVSRKRSSSLGGSTGS   908
ENSCGRP00001016053_Cgch/1-1081          LACIFFNKVYIILFKPSRNTIEEVRCSTAAHAFKVAARATLRRSNVSRKRSSSLGGSTGS   908
ENSCGRP00000017369_Cgcr/1-1081          LACIFFNKVYIILFKPSRNTIEEVRCSTAAHAFKVAARATLRRSNVSRKRSSSLGGSTGS   908
ENSDORP00000002959_Dord/1-1078          LACIFFNKVYIILFKPSRNTIEEVRCSTAAHAFKVAARATLRRSNVSRKRSSSLGGSTGS   908
ENSTSYP00000002643_Csyr/1-1015          LACIFFNKVYIILFKPARNTXXXXXXXXR-------------------------------   887
ENSMLUP00000008249_Mluc/1-1075          LACIFFNKVYIILFKPSRNTIEEVRCSTAAHAFKVAARATLRRSNVSRKRSGSLGGSTGS   907
ENSECAP00000022285_Ecab/1-1084          LACIFFNKVYIILFKPSRNTIEEVRCSTAAHAFKVAARATLRRSNVSRKRSSSLGGSTGS   908
ENSEASP00005022676_Eaas/1-1094          LACIFFNKVYIILFKPSRNTIEEVRCSTAAHAFKVAARATLRRSNVSRKRSSSLGGSTGS   918
ENSCAFP00000017381_Cfam/1-1075          LACIFFNKVYIILFKPSRNTIEEVRCSTAAHAFKVAARATLRRSNVSRKRSGSLGGSTGS   908
ENSCAFP00020007154_Cldi/1-1075          LACIFFNKVYIILFKPSRNTIEEVRCSTAAHAFKVAARATLRRSNVSRKRSGSLGGSTGS   908
ENSVVUP00000030826_Vvul/1-1075          LACIFFNKVYIILFKPSRNTIEEVRCSTAAHAFKVAARATLRRSNVSRKRSGSLGGSTGS   908
ENSFCAP00000008080_Fcat/1-1081          LACIFFNKVYIILFKPSRNTIEEVRCSTAAHAFKVAARATLRRSNVSRKRSSSLGGSTGS   908
ENSPPRP00000023800_Ppar/1-1081          LACIFFNKVYIILFKPSRNTIEEVRCSTAAHAFKVAARATLRRSNVSRKRSSSLGGSTGS   908
ENSPTIP00000015042_Ptal/1-1081          LACIFFNKVYIILFKPSRNTIEEVRCSTAAHAFKVAARATLRRSNVSRKRSSSLGGSTGS   908
ENSMPUP00000004759_Mpfu/1-1082          LACIFFNKVYIILFKPSRNTIEEVRCSTAAHAFKVAARATLRRSNVSRKRSSSLGGSTGS   908
ENSNVIP00000018564_Nvis/1-1092          LACIFFNKVYIILFKPSRNTIEEVRCSTAAHAFKVAARATLRRSNVSRKRSSSLGGSTGS   918
ENSUMAP00000035085_Umar/1-1071          LACIFFNKVYIILFKPSRNTIEEVRCSTAAHAFKVAARATLRRSNVSRKRSSSLGGSTGS   918
ENSUAMP00000035930_Uame/1-1079          LACIFFNKVYIILFKPSRNTIEEVRCSTAAHAFKVAARATLRRSNVSRKRSSSLGGSTGS   908
ENSAMEP00000015060_Amel/1-1089          LACIFFNKVYIILFKPSRNTIEEVRCSTAAHAFKVAARATLRRSNVSRKRSSSLGGSTGS   918
ENSSDAP00000007437_Sdau/1-1079          LACIFFNKVYIILFKPSRNTIEEVRCSTAAHAFKVAARATLRRSNVSRKRSXXXXXXXXX   908
ENSSTOP00000003945_Itri/1-1070          LACIFFNKVYIILFKPSRNTIEEVRCSTAAHAFKVAARATLRRSNVSRKRSNSLGGSTGS   908
ENSMMMP00000015298_Mmma/1-1083          LACIFFNKVYIILFKPSRNTIEEVRCSTAAHAFKVAARATLRRSNVSRKRSNSLGGSTGS   908
ENSUPAP00010025947_Upar/1-1086          LACIFFNKVYIILFKPSRNTIEEVRCSTAAHAFKVAARATLRRSNVSRKRSNSLGGSTGS   908
ENSSBOP00000036416_Sbbo/1-1078          LACIFFNKIYIILFKPSRNTIEEVRCSTAAHAFKVAARATLRRSNVSRKRSSSLGGSTGS   908
ENSANAP00000028404_Anan/1-1078          LACIFFNKIYIILFKPSRNTIEEVRCSTAAHAFKVAARATLRRSNVSRKRSSSLGGSTGS   908
ENSCCAP00000024327_Ccap/1-1077          LACIFFNKIYIILFKPSRNTIEEVRCSTAAHAFKVAARATLRRSNVSRKRSSSLGGSTGS   908
ENSCJAP00000062854_Cjac/1-1089          LACIFFNKIYIILFKPSRNTIEEVRCSTAAHAFKVAARATLRRSNVSRKRSSSLGGSTGS   918
ENSNLEP00000000336_Nleu/1-1088          LACIFFNKIYIILFKPSRNTIEEVRCSTAAHAFKVAARATLRRSNVSRKRSSSLGGSTGS   918
ENSPPYP00000015087_Pabe/1-1078          LACIFFNKIYIILFKPSRNTIEEVRCSTAAHAFKVAARATLRRSNVSRKRSSSLGGSTGS   908
ENSP00000420194_Hsap/1-1088             LACIFFNKIYIILFKPSRNTIEEVRCSTAAHAFKVAARATLRRSNVSRKRSSSLGGSTGS   918
ENSGGOP00000020287_Ggor/1-1078          LACIFFNKIYIILFKPSRNTIEEVRCSTAAHAFKVAARATLRRSNVSRKRSSSLGGSTGS   908
ENSPPAP00000026371_Ppan/1-1078          LACIFFNKIYIILFKPSRNTIEEVRCSTAAHAFKVAARATLRRSNVSRKRSSSLGGSTGS   908
ENSPTRP00000043342_Ptro/1-1078          LACIFFNKIYIILFKPSRNTIEEVRCSTAAHAFKVAARATLRRSNVSRKRSSSLGGSTGS   908
ENSCSAP00000003635_Csab/1-882           LACIFFNKIYIILFKPSRNTIEEVRCSTAAHAFKVAARATLRRSNVSRKRSSSLGGSTGS   712
ENSMMUP00000061002_Mmul/1-1078          LACIFFNKIYIILFKPSRNTIEEVRCSTAAHAFKVAARATLRRSNVSRKRSSSLGGSTGS   908
ENSPANP00000004316_Panu/1-1081          LACIFFNKIYIILFKPSRNTIEEVRCSTAAHAFKVAARATLRRSNVSRKRSSSLGGSTGS   911
ENSCATP00000028615_Caty/1-1078          LACIFFNKIYIILFKPSRNTIEEVRCSTAAHAFKVAARATLRRSNVSRKRSSSLGGSTGS   908
ENSMFAP00000042132_Mfas/1-1078          LACIFFNKIYIILFKPSRNTIEEVRCSTAAHAFKVAARATLRRSNVSRKRSSSLGGSTGS   908
ENSMNEP00000024602_Mnem/1-1078          LACIFFNKIYIILFKPSRNTIEEVRCSTAAHAFKVAARATLRRSNVSRKRSSSLGGSTGS   908
ENSMLEP00000008744_Mleu/1-1088          LACIFFNKIYIILFKPSRNTIEEVRCSTAAHAFKVAARATLRRSNVSRKRSSSLGGSTGS   918
ENSTGEP00000008766_Tgel/1-1088          LACIFFNKIYIILFKPSRNTIEEVRCSTAAHAFKVAARATLRRSNVSRKRSSSLGGSTGS   918
ENSCANP00000040262_Capa/1-1088          LACIFFNKIYIILFKPSRNTIEEVRCSTAAHAFKVAARATLRRSNVSRKRSSSLGGSTGS   918
ENSPTEP00000016080_Ptep/1-1088          LACIFFNKIYIILFKPSRNTIEEVRCSTAAHAFKVAARATLRRSNVSHKRSSSLGGSTGS   918
ENSRBIP00000006831_Rbie/1-1078          LACIFFNKIYIILFKPSRNTIEEVRCSTAAHAFKVAARATLRRSNVSRKRSSSLGGSTGS   908
ENSRROP00000007818_Rrox/1-1088          LACIFFNKIYIILFKPSRNTIEEVRCSTAAHAFKVAARATLRRSNVSRKRSSSLGGSTGS   918
ENSOGAP00000002850_Ogar/1-1077          LACIFFNKIYIILFKPSRNTIEEVRCSTAAHAFKVAARATLRRSNVSRKRSSSLGGSTGS   908
ENSMICP00000050654_Mmur/1-1075          LACIFFNKIYIILFKPSRNTIEEVRCSTAAHAFKVAARATLRRSNVSRKRSSSVGGSTGS   908
ENSPCOP00000009624_Pcoq/1-1076          LACIFFNKIYIILFKPSRNTIEEVRCSTAAHAFKVAARATLRRSNVSRKRSSSLGGSTGS   908
ENSPSMP00000007909_Psim/1-1084          LACIFFNKIYIILFKPSRNTIEEVRCSTAAHAFKVAARATLRRSNVSRKRSSSLGGSTGS   918
                                                :* **.**:***
```

```
ENSMEUP00000013310_Neug/1-956          TPSSSMS-SKSNSEDPFPQPEREKGQQQQQLQQ-----------------QQQQPLTPQQ   896
ENSTBEP00000002487_Tbel/1-914          TPSSSIS-SKSNSEDPFPQPERQKQQHPLALT-----------QQE-------------P   788
ENSPCAP00000000755_Pcap/1-1085         TPSSSIS-SKSNSEDPFPQPEGLKQQQPLALM------------PQPQPQQLLTLQQQQQ   960
ENSCHOP00000011907_Chof/1-954          TPSSSIS--SINSEDSFPQPEKQKQQQQQQPL-ALTQPEQ--------------------   954
ENSVPAP00000001982_Vpac/1-1087         TPSSSIS-SKSNSEDPFPQPERQKQQQPLALTQHVP---------------PPQPSNPQP   961
ENSEEUP00000009670_Eeur/1-946          TPSSSIS-SKSNSEDPFPPPERQKQQQPLALTQ---------------------QEQQPQ   820
ENSOANP00000015961_Oana/1-1083         TPSSSIS-TKSNAEEAAGRPEPWQRQGVPPPP-GSQ--------------GSQGPPAPQR   952
ENSOPRP00000000261_Opri/1-1082         TPSSSIS-SKSNSEDPFPQSERLKQPPPPLAS----------------------PQPGPK   955
ENSJJAP00000004721_Jjac/1-1084         LPSSSSIGSGSGGDDAFPQPDGPKQPQPQPLPLALTQPEQHLHQQQQQQQ----QQQQPR   940
ENSPCIP00000034747_Pcin/1-1018         TPSSSMS-SKSNSEDPFPQPEREKEQQQQT----------------------LT-P-QQQ   892
ENSMODP00000022810_Mdom/1-1069         TPSSSMS-SKSNSEDPFPQPEREKEQQQQP-------------------------L-TPQ   943
ENSVURP00010007344_Vurs/1-1070         TPSSSMS-SKSNSEDPFPQPEREKGQQQQ-----------------------QTLT-PQQ   944
ENSSHAP00000014890_Shar/1-1072         TPSSSMS-SKSNSEDPFPQPDREKEQQQQQQ---------------------QPLT-PQQ   946
ENSMAUP00000005394_Maur/1-954          IPSSSIS-SKSNSEDPFPQPERQKQQQPLRASMCQHILAWCLTPNKHL------------   904
ENSOCUP00000025672_Ocun/1-1063         TPSSSISSXXXXXXXXXXXXXXXXXXXXXXXXXXXXXXXXXXXPERQKQPPPLLCPAAAA   968
ENSPVAP00000011981_Pvam/1-1085         TPSSSIS-SKSNSEDPFPQPEGQKQ----QQPLALTQQEQRR--------QPQLPLTIQQ   959
ENSETEP00000011160_Etel/1-1024         TPSSSIS-SKSNSEDPFPQPKQAEERPKQQQPLTLTQDE---------------RQDQQQ   898
ENSFDAP00000010532_Fdam/1-1108         IPSSSIS-SKSNSEDPFPQPERQKQQQP----LALTGQEQ-------Q--HPMTLQLQQP   982
ENSODEP00000013257_Odeg/1-1070         IPSSSIS-SKSNSEDPFPQPERQKQQHP----LQQQ--------QQQQ--QPMPLQPQQP   953
ENSHGLP00000002019_Hgfe/1-1108         IPSSSIS-SKSNSEDPFPQAERQKQQRP----LALTGQEQQ------Q--QPVTLQPQQP   983
ENSCLAP00000021334_Clan/1-1114         IPSSSIS-SKSNSEDPFPQPERQKQQRP----PALTGQEQQQQQQQQQ--QAVTLQPQQP   989
ENSCAPP00000014352_Cape/1-1068         IPSSSIS-SKSNSEDPFPQPERLKQQWP----VALTGQEQQQQQPQ---------QQPQP   954
ENSCPOP00000011705_Cpor/1-1071         IPSSVYQVARATAKIPSHSPRGRCNSGP----VALTGQEQQQQQPQ-Q--HPVPLQPQQP   957
ENSSARP00000006151_Sara/1-1085         TPSSSIS-SKSNSEDPFPPPERQKQ--Q--QPLALAPQEQPPQL-----------QQQPP   959
ENSLAFP00000018459_Lafr/1-1090         TPSSSIS-SKSNSEDPFPQPERQK--QE--QPLALTQQEQQQPQ-------PL---TLRR   964
ENSDNOP00000011819_Dnov/1-1075         TPSSSIS-SKSNSEDPFPQPEGQKQRQP----LALTQQEQQQ--------------QPLA   949
ENSSSCP00000041077_Sscr/1-1089         TPSSSIS-SKSNSEDPFPQPERQKKQQ----PLALTQ---HVPQ-------PQAPSTPQP   963
ENSTTRP00000001326_Ttru/1-1091         TPSSSIS-SKSNSEDPFPQPERQK--QQ--QPLALTQ-CAQQPQ-------PRPPSTLQL   965
ENSBBBP00000002858_Bbbi/1-1068         TPSSSIS-SKSNSEDPFPQQQPKRQKQP--QPLALSPHN---------------------   943
ENSOARP00000021602_Oari/1-1032         TPSSSIS-SKSNSE----------------------------------------------   922
ENSCHIP00000033385_Chir/1-1085         TPSSSIS-SKSNSEDPFPQQQPERQKQP--QPLALSPHNAQQPQ-------PRPPSTPQP   959
ENSBTAP00000059078_Btau/1-1085         TPSSSIS-SKSNSEDPFPQQQPKRQKQP--QPLALSPHNAQQPQ-------PRPPSTPQP   959
ENSBIXP00000038534_Bthy/1-1085         TPSSSIS-SKSNSEDPFPQQQPKRQKQP--QPLALSPHNAQQPQ-------PRPPSTPQP   959
ENSBMUP00000019115_Bmut/1-1081         TPSSSIS-SKSNSEDPFPQRQ----KQP--QPLALSPHNAQQPQ-------PRPPSTPQP   955
ENSBIXP00005030570_Bihy/1-1085         TPSSSIS-SKSNSEDPFPQQQPKRQKQP--QPLALSPHNAQQPQ-------PRPPSTPQP   959
ENSMUGP00000006365_Mung/1-1080         IPSSSMS-SKSNSEDPFPQPERQKQQQP--L--ALTQQEQQ-QQ--------QPLALQPQ   954
ENSRNOP00000069629_Rnor/1-1079         IPSSSIS-SKSNSEDRFPQPERQKQQQP--L--SLTQQEQ--QQ--------QPLTLHPQ   953
MGP_PahariEiJ_P0028852_Mpah/1-1003     IPSSSIS-SKSNSEDRFPQPERQKQQQP--L--ALTQQEQ-QQQ--------QPLTLQPQ   877
MGP_SPRETEiJ_P0043347_Mspr/1-1079      IPSSSIS-SKSNSEDRFPQPERQKQQQP--L--ALTQQEQ--QQ--------QPLTLQPQ   953
ENSMUSP00000069080_Mmus/1-1079         IPSSSIS-SKSNSEDRFPQPERQKQQQP--L--ALTQQEQ--QQ--------QPLTLQPQ   953
ENSMOCP00000010340_Moch/1-1076         IPSSI-S-SKSNSEDPFPQLERQKQQQR--L--KQQQQKQQQQ------------PLTLQ   950
ENSPEMP00000029995_Pmba/1-1076         IPSSSIS-SKSNSEDPFPQPERRKQQQP--L--ALTQQEQQQQQ--------P--LALQQ   953
ENSCGRP00015024036_Cgpi/1-1081         IPSSSIS-SKSNSEDPFPQPERQKQQQP--L--ALSQQEQQPQQ--------QPLTLQQQ   955
ENSCGRP00001016053_Cgch/1-1081         IPSSSIS-SKSNSEDPFPQPERQKQQQP--L--ALSQQEQQPQQ--------QQPLTLQQ   955
ENSCGRP00000017369_Cgcr/1-1081         IPSSSIS-SKSNSEDPFPQPERQKQQQP--L--ALSQQEQQPQQ--------QQPLTLQQ   955
ENSDORP00000002959_Dord/1-1078         IPSSSIS-SKSNSEDPFPQPERQKQQPP--P-LQ----------QQQQ--QQQPLAL-QP   951
ENSTSYP00000002643_Csyr/1-1015         ----------------------------------------------------------SP   889
ENSMLUP00000008249_Mluc/1-1075         TPSSSIS-SKSNSEDPFPQPERQKPQ----QPLALPQQEPQPQPQ-----------P-PS   950
ENSECAP00000022285_Ecab/1-1084         TPSSSIS-SKSNSEDPFPQPERQKQQ----QPLALTQQEQQQQQQQPQ--QPLTL---QQ   958
ENSEASP00005022676_Eaas/1-1094         TPSSSIS-SKSNSEDPFPQPERQKQQ----QPLALTQQEQQQQQQQPQ--QPLTL---QQ   968
ENSCAFP00000017381_Cfam/1-1075         TPSSSIS-SKSNSEDPFPQPERQKQ-Q---QPLALTQREQQ-----PP--QPL-------   949
ENSCAFP00020007154_Cldi/1-1075         TPSSSIS-SKSNSEDPFPQPERQKQ-Q---QPLALTQREQQ-----PP--QPL-------   949
ENSVVUP00000030826_Vvul/1-1075         TPSSSIS-SKSNSEDPFPQPERQKQ-Q---QPLALTQREQQ-----PP--QPL-------   949
ENSFCAP00000008080_Fcat/1-1081         TPSSSIS-SKSNSEDPFPQPERQKQ-Q---QPLALTQQEQQ-----PQ--PQQPSSL-QQ   955
ENSPPRP00000023800_Ppar/1-1081         TPSSSIS-SKSNSEDPFPQPERQKQ-Q---QPLALTQQEQQ-----PQ--PQQPSSL-QQ   955
ENSPTIP00000015042_Ptal/1-1081         TPSSSIS-SKSNSEDPFPQPERQKQ-Q---QPLALTQQEQQ-----PQ--PQQPSSL-QQ   955
ENSMPUP00000004759_Mpfu/1-1082         TPSSSIS-SKSNSEDPFPQPERQKQQQ---QPLALTQQEQQ-----PQ--QPLTLQQ-QP   956
ENSNVIP00000018564_Nvis/1-1092         TPSSSIS-SKSNSEDPFPQPERQKQQQ---QPLALTQQEQQ-----LQ--QPLTLQQ-QP   966
ENSUMAP00000035085_Umar/1-1071         TPSSSIS-SKSNSEDPFPQPERQKQ-Q---QPLALTQQEQQ-----PQ--QPLTLQQ-Q-   964
ENSUAMP00000035930_Uame/1-1079         TPSSSIS-SKSNSEDPFPQPERQKQ-Q---QPLALTQQEQQ-----PQ--QPLTLQQ-Q-   954
ENSAMEP00000015060_Amel/1-1089         TPSSSIS-SKSNSEDPFPQPERQKQ-Q---QPLALTQQEQQ-----PQ--QPLTLQQ-Q-   964
ENSSDAP00000007437_Sdau/1-1079         XXXXXXXXXXXXXXXXXXXXXXXXXXX----------XXXXQQQQQQ---QPLTL-Q-PQ   953
ENSSTOP00000003945_Itri/1-1070         LPSSSIS-SKSNSEDPFPQPERQKQQQ---QPLALTQQE--------------------Q   944
ENSMMMP00000015298_Mmma/1-1083         LPSSSIS-SKSNSEDPFPQPERPKQQ----QPLALTQQEQQ-QQQQQ---QPLTL-Q-PQ   957
ENSUPAP00010025947_Upar/1-1086         LPSSSIS-SKSNSEDPFPQPERQKQQQQ--QPLALTQQEQQQQQQQQ---QPLTL-Q-PQ   960
ENSSBOP00000036416_Sbbo/1-1078         TPSSSIS-SKSNSEDPFPQPERQKQQ----QPLALTQQEQQ-----Q---QSLTL-Q--Q   952
ENSANAP00000028404_Anan/1-1078         TPSSSIS-SKSNSEDPFPQPERQKQQ----QPLALTQQEQQ-----Q---QPLTL-Q--Q   952
ENSCCAP00000024327_Ccap/1-1077         TPSSSIS-SKSNSEDPFPQPERQKQQ----QPLALTQQEQQ---------QPLTL-Q--Q   951
ENSCJAP00000062854_Cjac/1-1089         TPSSSIS-SKSNSEDPFPQPERQKQQ----QPLALTQQEEQQ----Q---QPLTL-Q--Q   963
ENSNLEP00000000336_Nleu/1-1088         TPSSSIS-SKSNSEDPFPPPERQKQQ----QPLALTQQEQ-Q----Q---QPLTL-P--Q   962
ENSPPYP00000015087_Pabe/1-1078         TPSSSIS-SKSNSEDPFPQPERQKQQ----QPLALTQQEQ-Q----Q---QPLTL-P--Q   952
ENSP00000420194_Hsap/1-1088            TPSSSIS-SKSNSEDPFPQPERQKQQ----QPLALTQQEQ-Q----Q---QPLTL-P--Q   962
ENSGGOP00000020287_Ggor/1-1078         TPSSSIS-SKSNSEDPFPQPERQKQQ----QPLALAQQEQ-Q----Q---QPLTL-P--Q   952
ENSPPAP00000026371_Ppan/1-1078         TPSSSIS-SKSNSEDPFPQPERQKQQ----QPLALTQQEQ-Q----Q---QPLTL-P--Q   952
ENSPTRP00000043342_Ptro/1-1078         TPSSSIS-SKSNSEDPFPQPERQKQQ----QPLALTQQEQ-Q----Q---QPLTL-P--Q   952
ENSCSAP00000003635_Csab/1-882          TPSSSIS-SKSNSEDPFPQPERQKQQ----QPLALTQQGQ-Q----Q---QPLTL-P--Q   756
ENSMMUP00000061002_Mmul/1-1078         TPSSSIS-SKSNSEDPFPQPERQKQQ----QPLALTQQEQ-Q----Q---QPLTL-P--Q   952
ENSPANP00000004316_Panu/1-1081         TPSSSIS-SKSNSEDPFPQPERQKQQ----QPLALTQQEQ-Q----Q---QPLTL-P--Q   955
ENSCATP00000028615_Caty/1-1078         TPSSSIS-SKSNSEDPFPQPERQKQQ----QPLALTQQEQ-Q----Q---QPLTL-P--Q   952
ENSMFAP00000042132_Mfas/1-1078         TPSSSIS-SKSNSEDPFPQPERQKQQ----QPLALTQQEQ-Q----Q---QPLTL-P--Q   952
ENSMNEP00000024602_Mnem/1-1078         TPSSSIS-SKSNSEDPFPQPERQKQQ----QPLALTQQEQ-Q----Q---QPLTL-P--Q   952
ENSMLEP00000008744_Mleu/1-1088         TPSSSIS-SKSNSEDPFPQPERQKQQ----QPLALTQQEQ-Q----Q---QPLTL-P--Q   962
ENSTGEP00000008766_Tgel/1-1088         TPSSSIS-SKSNSEDPFPQPERQKQQ----QPLALTQQEQ-Q----Q---QPLTL-P--Q   962
ENSCANP00000040262_Capa/1-1088         TPSSSIS-SKSNSEDPFPQPERQKQQ----QPLALTQQEQ-Q----Q---QPLTL-L--Q   962
ENSPTEP00000016080_Ptep/1-1088         TPSSSIS-SKSNSEDPFPQPERQKQQ----QPLALTQQEQ-Q----Q---QPLTL-P--Q   962
ENSRBIP00000006831_Rbie/1-1078         TPSSSIS-SKSNSEDPFPQPERQKQQ----QPLALTQQEQ-P----Q---QPLTL-P--Q   952
ENSRROP00000007818_Rrox/1-1088         TPSSSIS-SKSNSEDPFPQPERQKQQ----QPLALTQQEQ-P----Q---QPLTL-P--Q   962
ENSOGAP00000002850_Ogar/1-1077         TPSSSIS-SKSNSEDPFPQPERQKQQ----PPPALTSQEQQ---------QPLTL-Q--Q   951
```

ENSMICP00000050654_Mmur/1-1075	TPSSSIS-SKSNSEDPFPQPDRQKPQ----PPPALTQQEQQ---------QQP-L-A---	949
ENSPCOP00000009624_Pcoq/1-1076	TPSSSIS-SKSNSEDPFPQPERQKQQ----QPPALTQQEQQ---------QQP-L-T--L	950
ENSPSMP00000007909_Psim/1-1084	TPSSSIS-SKSNSEDPFPQPERQKRQ----QPPALTQPEQQ---------QQP-L-----	958

ENSMEUP00000013310_Neug/1-956	QRPQQQQHRCKQKVIFGSGTVTFSLSFDEPQKNAMAH---------KTKQQNSLEAQNS-	946
ENSTBEP00000002487_Tbel/1-914	QPQQSQQPRCKQKVIFGSGTVTFSLSFDEPQKNTTA--------HRNSTHQNSLETQKG-	839
ENSPCAP00000000755_Pcap/1-1085	QQAQQQQPRCKQKVIFGSGTVTFSLSFDEPQKNAMA--------HRNSTHHNSLEAQKS-	1011
ENSCHOP00000011907_Chof/1-954	--	954
ENSVPAP00000001982_Vpac/1-1087	QPQSQQQLSCKQKVIFGSGTVTFSLSFDEPQKSAVA--------HRNSTHQNSLEAQKN-	1012
ENSEEUP00000009670_Eeur/1-946	PQPQQQPPRCKQKVIFGSGTVTFSLSFDEPQKGATG--------HRNSTQQNSLEAQKG-	871
ENSOANP00000015961_Oana/1-1083	GTPPPPPPRSKQKVIFGSGTVTFSLSFDEPRKDAGA--------HRHGRRRHSLEPQDSG	1004
ENSOPRP00000000261_Opri/1-1082	QQQPPHQPRCKQKVIFGSGTVTFSLSFDEPQKNAMT--------PRNSQRQNPTEAQKS-	1006
ENSJJAP00000004721_Jjac/1-1084	LQQQPPQPRCKQKVIFGSGTVTFSLSFDEPHKNATAAAARNNSSSSSSGRHNSLEAQKS-	999
ENSPCIP00000034747_Pcin/1-1018	RPQQQQQHRCKQKVIFGSGTVTFSLSFDEPQKNAMS--------HKVTKQQNSLEAQSS-	943
ENSMODP00000022810_Mdom/1-1069	PQRPQQQHRCKQKVIFGSGTVTFSLSFDEPQKNATA--------HKVAKQQNSLEAQNS-	994
ENSVURP00010007344_Vurs/1-1070	QRPQQQQHRCKQKVIFGSGTVTFSLSFDEPQKNAMA--------HKVTKQQNSLEAQNS-	995
ENSSHAP00000014890_Shar/1-1072	QRPQQQQHRCKQKVIFGSGTVTFSLSFDEPQKNAMA--------HKVAKQQNSLEAQNS-	997
ENSMAUP00000005394_Maur/1-954	--VLVNMI-	910
ENSOCUP00000025672_Ocun/1-1063	AAASAAASKCKQKVIFGSGTVTFSLSFDEPQKNAMA--------PRNSMHRNSLEAQKS-	1019
ENSPVAP00000011981_Pvam/1-1085	QQQSQQQPRCKQKVIFGSGTVTFSLSFDEPQKSATA--------HRNSIHQNSLEAQKS-	1010
ENSETEP00000011160_Etel/1-1024	QQQAQQQPRCKQKVIFGSGTVTFSLSFDEPQKNAMA--------QRNPTHHNSLEAQKS-	949
ENSFDAP00000010532_Fdam/1-1108	-QQSQPQPRCKQKVIFGSGTVTFSLSFDEPQKNITA--------HRNATHRNSLENQKS-	1032
ENSODEP00000013257_Odeg/1-1070	-QPSQPQPRCKQKVIFGSGTVTFSLSFDEPQKNAMV--------HRNATHQNSLETQKS-	1003
ENSHGLP00000002019_Hgfe/1-1108	-QQSQPQPRCKQKVIFGSGTVTFSLSFDEPQKNAMA--------HRNTTHQNSLETQKS-	1033
ENSCLAP00000021334_Clan/1-1114	-QQSQPQPRCKQKVIFGSGTVTFSLSFDEPQKNTMT--------HRNATHHNSLETQKS-	1039
ENSCAPP00000014352_Cape/1-1068	-QQSQPQPRCKQKVIFGSGTVTFSLSFDEPQKNATA--------HRHATHQNSLETQKN-	1004
ENSCPOP00000011705_Cpor/1-1071	-QQSQPQPRCKQKVIFGSGTVTFSLSFDEPQKNATA--------HRHATHQNSLETQKN-	1007
ENSSARP00000006151_Sara/1-1085	TPQSQQPPRCKQKVIFGSGTVTFSLSFDEPQKSALT--------PRNSTHQNSLETQKS-	1010
ENSLAFP00000018459_Lafr/1-1090	QQQTQQQPRCKQKVIFGSGTVTFSLSFDEPQKNAMA--------HRNSTHQNSLEAQKS-	1015
ENSDNOP00000011819_Dnov/1-1075	PQQQQGQPRCKQKVIFGSGTVTFSLSFDEPQKNAVA--------HRNSTHQNSLEAQRS-	1000
ENSSSCP00000041077_Sscr/1-1089	QPQLQQQPRCKQKVIFGSGTVTFSLSFDEPQKSATA--------HRNSTHQNSLEAQKN-	1014
ENSTTRP00000001326_Ttru/1-1091	QQQSQQQPRCKQKVIFGSGTVTFSLSFDEPQKSAMA--------HRNSTHQNSLEAQKN-	1016
ENSBBBP00000002858_Bbbi/1-1068	-PQSQQPPRCKQKVIFGSGTVTFSLSFDEPQKSAVA--------HRNSTHQTSLEAQKN-	993
ENSOARP00000021602_Oari/1-1032	----------------DRGTVTFSLSFDEPQKSAVA--------HRNSTHQTSLEAQKN-	957
ENSCHIP00000033385_Chir/1-1085	QPQSQQPPRCKQKVIFGSGTVTFSLSFDEPQKSAVA--------HRNSTHQTSLEAQKN-	1010
ENSBTAP00000059078_Btau/1-1085	QPQSQQPPRCKQKVIFGSGTVTFSLSFDEPQKTAVA--------HRNSTHQTSLEAQKN-	1010
ENSBIXP00000038534_Bthy/1-1085	QPQSQQPPRCKQKVIFGSGTVTFSLSFDEPQKTAVA--------HRNSTHQTSLEAQKN-	1010
ENSBMUP00000019115_Bmut/1-1081	QPQSQQPPRCKQKVIFGSGTVTFSLSFDEPQKSAVA--------HRNSTHQTSLEAQKN-	1006
ENSBIXP00005030570_Bihy/1-1085	QPQSQQPPRCKQKVIFGSGTVTFSLSFDEPQKSAVA--------HRNSTHQTSLEAQKN-	1010
ENSMUGP00000006365_Mung/1-1080	PQQQPQQPRCKQKVIFGSGTVTFSLSFDEPQKNAVA--------HRNSVRQNSLEAQKS-	1005
ENSRNOP00000069629_Rnor/1-1079	QQQQPQQPRCKQKVIFGSGTVTFSLSFDEPQKNAMA--------HRNSMRQNSLEAQRS-	1004
MGP_PahariEiJ_P0028852_Mpah/1-1003	QQQQPQQPRCKQKVIFGSGTVTFSLSFDEPQKNAMA--------HRNSVRQSSLEAQKS-	928
MGP_SPRETEiJ_P0043347_Mspr/1-1079	QQQQPQQPRCKQKVIFGSGTVTFSLSFDEPQKNAMA--------HRNSMRQNSLEAQKS-	1004
ENSMUSP00000069080_Mmus/1-1079	QQQQPQQPRCKQKVIFGSGTVTFSLSFDEPQKNAMA--------HRNSMRQNSLEAQKS-	1004
ENSMOCP00000010340_Moch/1-1076	KQQQPQQPRCKQKVIFGSGTVTFSLSFDEPQKSAMA--------HRNSTRHNSLEAQKS-	1001
ENSPEMP00000029995_Pmba/1-1076	QQQQPQQPRCKQKVIFGSGTVTFSLSFDEPQKSAMG--------HRNSIRQNSLEAQKS-	1004
ENSCGRP00015024036_Cgpi/1-1081	QQQPPQQPRCKQKVIFGSGTVTFSLSFDEPQKNAMA--------HRNSMRQNSLEAQKS-	1006
ENSCGRP00001016053_Cgch/1-1081	QQQPPQQPRCKQKVIFGSGTVTFSLSFDEPQKNAMA--------HRNSMRQNSLEAQKS-	1006
ENSCGRP00000017369_Cgcr/1-1081	QQQPPQQPRCKQKVIFGSGTVTFSLSFDEPQKNAMA--------HRNSMRQNSLEAQKS-	1006
ENSDORP00000002959_Dord/1-1078	QPLQQQPPRCTQKVIFGSGTVTFSLSFDEPQKNAMA--------HRNSTPQNSLEAQKS-	1002
ENSTSYP00000002643_Csyr/1-1015	QQHSRQQPRCKQKVIFGSGTVTFSLSFDEPQKNAMA--------HRNATHQNSLEAQKS-	940
ENSMLUP00000008249_Mluc/1-1075	ALQPQQQPRCKQ-VIFGSGTVTFSLSFDEPQKSAAA--------HRNSTHQNSLESQKS-	1000
ENSECAP00000022285_Ecab/1-1084	QQQAQQQPRCKQKVIFGSGTVTFSLSFDEPQKSAMA--------HRNSTHQNSLESQKS-	1009
ENSEASP00005022676_Eaas/1-1094	QQQAQQQPRCKQKVIFGSGTVTFSLSFDEPQKSAMA--------HRNSTHQNSLESQKS-	1019
ENSCAFP00000017381_Cfam/1-1075	TLPPQPQPRCKQKVIFGSGTVTFSLSFDEPQKSAAA--------PRNSTLQHSLEAQRS-	1000
ENSCAFP00020007154_Cldi/1-1075	TLPPQPQPRCKQKVIFGSGTVTFSLSFDEPQKSAAA--------PRNSTLQHSLEAQRS-	1000
ENSVVUP00000030826_Vvul/1-1075	TLPPQPQPRCKQKVIFGSGTVTFSLSFDEPQKSAAA--------PRNSTLQHSLEAQRS-	1000
ENSFCAP00000008080_Fcat/1-1081	QPQPQPQPRCKQKVIFGSGTVTFSLSFDEPQKSAMA--------HRNSMHQNSLEAQKS-	1006
ENSPPRP00000023800_Ppar/1-1081	QPQPQPQPRCKQKVIFGSGTVTFSLSFDEPQKSAMA--------HRNSMHQNSLEAQKS-	1006
ENSPTIP00000015042_Ptal/1-1081	QPQPQPQPRCKQKVIFGSGTVTFSLSFDEPQKSAMA--------HRNSMHQNSLEAQKS-	1006
ENSMPUP00000004759_Mpfu/1-1082	QPQPQPQPRCKQKVIFGSGTVTFSLSFDEPQKSAMA--------HRNSTHQNSLEAQRS-	1007
ENSNVIP00000018564_Nvis/1-1092	QPQPQPQPRCKQKVIFGSGTVTFSLSFDEPQKSAMA--------HRNSTHQNSLEAQRS-	1017
ENSUMAP00000035085_Umar/1-1071	-PQPQPQPRCKQKVIFGSGTVTFSLSFDEPQKSAM------------------------	998
ENSUAMP00000035930_Uame/1-1079	-PQPQPQPRCKQKVIFGSGTVTFSLSFDEPQKSAMA--------HRNATHQNSLETQRS-	1004
ENSAMEP00000015060_Amel/1-1089	-PQPQPQPRCKQKVIFGSGTVTFSLSFDEPQKSAMA--------HRNATHQNSLETQRS-	1014
ENSSDAP00000007437_Sdau/1-1079	QQQSKQQPRCKQKVIFGSGTVTFSLSFDEPQKNAMA--------HRNSTHQNSLEAQRS-	1004
ENSSTOP00000003945_Itri/1-1070	QQQQQQQQRCKQKVIFGSGTVTFSLSFDEPQKNAMA--------HRNSTHQNSLEAQRS-	995
ENSMMMP00000015298_Mmma/1-1083	QQQSQQQPRCKQKVIFGSGTVTFSLSFDEPQKNAMA--------HRNSTHQNSLEAQRS-	1008
ENSUPAP00010025947_Upar/1-1086	QQQSQQQPRCKQKVIFGSGTVTFSLSFDEPQKNAMA--------HRNSTHQNSLEAQRS-	1011
ENSSBOP00000036416_Sbbo/1-1078	QQQSQQQPRCKQKVIFGSGTVTFSLSFDEPQKNAMA--------HRNSTHQNSLEAQKS-	1003
ENSANAP00000028404_Anan/1-1078	QQQSQQQPRCKQKVIFGSGTVTFSLSFDEPQKNAMA--------HRNSTHQNSLEAQKS-	1003
ENSCCAP00000024327_Ccap/1-1077	QQQSQQQPRCKQKVIFGSGTVTFSLSFDEPQKNATA--------HRNSMHQNSLEAQKS-	1002
ENSCJAP00000062854_Cjac/1-1089	QQHSQQQPRCKQKVIFGSGTVTFSLSFDEPQKNAMA--------HRNSMHQNSLEAQKS-	1014
ENSNLEP00000000336_Nleu/1-1088	QQQSQQQPRCKQKVIFGSGTVTFSLSFDEPQKNAMA--------HRNSTHQNSLEAQKS-	1013
ENSPPYP00000015087_Pabe/1-1078	QQQSQQQPRCKQKVIFGSGTVTFSLSFDEPQKNAMA--------HRNSTHQNSLEAQKS-	1003
ENSP00000420194_Hsap/1-1088	QQRSQQQPRCKQKVIFGSGTVTFSLSFDEPQKNAMA--------HRNSTHQNSLEAQKS-	1013
ENSGGOP00000020287_Ggor/1-1078	QQQSQQQPRCKQKVIFGSGTVTFSLSFDEPQKNAMA--------HRNSTHQNSLEAQKS-	1003
ENSPPAP00000026371_Ppan/1-1078	QQQSQQQPRCKQKVIFGSGTVTFSLSFDEPQKNAMA--------HRNSTHQNSLEAQKS-	1003
ENSPTRP00000043342_Ptro/1-1078	QQQSQQQPRCKQKVIFGSGTVTFSLSFDEPQKNAMA--------HRNSTHQNSLEAQKS-	1003
ENSCSAP00000003635_Csab/1-882	QQQSQQQPRCKQKVIFGSGTVTFSLSFDEPQKNAMA--------HRNSTHQNSLEAQKS-	807
ENSMMUP00000061002_Mmul/1-1078	QQQSQQQPRCKQKVIFGSGTVTFSLSFDEPQKNAMA--------HRNSTHQNSLEAQKS-	1003
ENSPANP00000004316_Panu/1-1081	QQQSQQQPRCKQKVIFGSGTVTFSLSFDEPQKNAMA--------HRNSTHQNSLEAQKS-	1006
ENSCATP00000028615_Caty/1-1078	QQQSQQQPRCKQKVIFGSGTVTFSLSFDEPQKNAMA--------HRNSTHQNSLEAQKS-	1003
ENSMFAP00000042132_Mfas/1-1078	QQQSQQQPRCKQKVIFGSGTVTFSLSFDEPQKNAMA--------HRNSTHQNSLEAQKS-	1003
ENSMNEP00000024602_Mnem/1-1078	QQQSQQQPRCKQKVIFGSGTVTFSLSFDEPQKNAMA--------HRNSTHQNSLEAQKS-	1003
ENSMLEP00000008744_Mleu/1-1088	QQQSQQQPRCKQKVIFGSGTVTFSLSFDEPQKNAMA--------HRNSTHQNSLEAQKS-	1013
ENSTGEP00000008766_Tgel/1-1088	QQQSQQQPRCKQKVIFGSGTVTFSLSFDEPQKNAMA--------HRNSTHQNSLEAQKS-	1013

ENSCANP00000040262_Capa/1-1088	QQQSQQQPRCKQKVIFGSGTVTFSLSFDEPQKNAMA--------HRNSTHQNSLEAQKS-	1013
ENSPTEP00000016080_Ptep/1-1088	QQQSQQQPRCKQKVIFGSGTVTFSLSFDEPQKNAMA--------HRNSTHQNSLEAQKS-	1013
ENSRBIP00000006831_Rbie/1-1078	QQQSQQQPRCKQKVIFGSGTVTFSLSFDEPQKNAMA--------HRNSTHQNSLEAQKS-	1003
ENSRROP00000007818_Rrox/1-1088	QQQSQQQPRCKQKVIFGSGTVTFSLSFDEPQKNAMA--------HRNSTHQNSLEAQKS-	1013
ENSOGAP00000002850_Ogar/1-1077	QPPPQPQPRCKQKVIFGSGTVTFSLSFDEPQKNAPA--------HRNSAHQNSLEAQKS-	1002
ENSMICP00000050654_Mmur/1-1075	LQQPQPQPRCKQKVIFGSGTVTFSLSFDEPQKNATA--------HRNSTPRNSLEAQKS-	1000
ENSPCOP00000009624_Pcoq/1-1076	QQQPQPQPRCKQKVIFGSGTVTFSLSFDEPQKNATA--------HRNSTPQNSLEAQKT-	1001
ENSPSMP00000007909_Psim/1-1084	TLQPQPQPRCKQKVIFGSGTVTFSLSFDEPQKNATA--------HRNSTPQNSLEAQKG-	1009

ENSMEUP00000013310_Neug/1-956	--GDSLGRRQAL--	956
ENSTBEP00000002487_Tbel/1-914	--SDALTRHQALLPLQCGENDSEPATQ------DTGLQGP-VGGDHPPEME---DPEEMS	887
ENSPCAP00000000755_Pcap/1-1085	--NEMLTRHQALLPLQCTEPGSELTRQ------ETDLQGS-PGGDYQVEME---GPEKRA	1059
ENSCHOP00000011907_Chof/1-954	--	954
ENSVPAP00000001982_Vpac/1-1087	--NDALTRHQALLPLQCGEADSELSTQ------ETGLQGP-VGRDHQPEME---DPEEMS	1060
ENSEEUP00000009670_Eeur/1-946	--GDPLPRHQALLPLQCGDTDSDPIPP------ETGLQGP-VGGDCQAEMQ---DPEEMS	919
ENSOANP00000015961_Oana/1-1083	GGGDGPVRPRALLPLRDGNPGSDPGSDPGS--REACLPE--------SGGEEPRDPEEGP	1054
ENSOPRP00000000261_Opri/1-1082	--NETLTRHQALLPLQCGETEAELMMGQ-----EESMHG-PVGGDCRPDT---EAPEEMS	1055
ENSJJAP00000004721_Jjac/1-1084	--NDTLSRHQALLPLQCGDTDSETTIP------DAGLQRPIMVGDHQPEMQLEEEEEEMS	1051
ENSPCIP00000034747_Pcin/1-1018	--GDSLARRQALVPLQSSEAGSGLGSG------VLGSEE-SVVGDSQPDT--QIP-EEMS	991
ENSMODP00000022810_Mdom/1-1069	--GDSLARRQALLPLQSSEGGAGLGSG------DSGSNE-SVVGDSQSES--QIP-EEMS	1042
ENSVURP00010007344_Vurs/1-1070	--GDSLARRQALVPLQSSEAGSGLGSG------VLGSNE-SVVGDSQPET--QIP-EEMS	1043
ENSSHAP00000014890_Shar/1-1072	--GDSLARRQALLPLQSSEAGSELDSG------DLDSNE-SVVGDSQPET--QIP-EEMS	1045
ENSMAUP00000005394_Maur/1-954	--SDTVTTSFHLLRLCYSLDY-DPQGPHI---------------------------KRLV	940
ENSOCUP00000025672_Ocun/1-1063	--NDTLTRHQALPPLQRGETDSETDGPGDRPARAYGWGP-PARD--------------QG	1062
ENSPVAP00000011981_Pvam/1-1085	--NDTMTRHQALLPLQYGETDSELTTQ------ETGLQG-PVGGDCQPET--EDP-EELS	1058
ENSETEP00000011160_Etel/1-1024	--SETLVRHQALLPLQCAEMGSELAVP------ETDLQG-SAGGDRQPEM--EDP-EELT	997
ENSFDAP00000010532_Fdam/1-1108	--SDGLSRHQALLPLQYRDTDAGLATQ------ETGLQG-LVAGDHQPEV--EE-TEEGS	1080
ENSODEP00000013257_Odeg/1-1070	--NDSLTKHQALLPLQCRDTDSDLSTQ------DTGLQG-LGAGDHQPEM--EAP-EEMA	1051
ENSHGLP00000002019_Hgfe/1-1108	--NDGLSRHQALLPLQYRDTDSGLTTQ------ETGQQG-MVAGDHQPEL--EDP-EEMS	1081
ENSCLAP00000021334_Clan/1-1114	--NDGLTRHQGLLPLQGRDTDSDLTTQ------ETGMQG-SVAGDHQPEV--EDP-EEMS	1087
ENSCAPP00000014352_Cape/1-1068	--NDGLTRHQA---LQCRDADSDPSTQ------EKG----------QPEM--EDPEEEMS	1041
ENSCPOP00000011705_Cpor/1-1071	--NDGLTRHQA---LQCRDADSDPSTQ------EKG----------QPEM--EDPEEEMS	1044
ENSSARP00000006151_Sara/1-1085	--NDALTRHQALPPLQGEEADLELSPQ------EAGLPG-PV-GGEHQPE--MEGREEMS	1058
ENSLAFP00000018459_Lafr/1-1090	--NEALTKHQALLPLQCTEPGSELTMQ------DTDLQG-SA-GGDHQLE--TEGPEERA	1063
ENSDNOP00000011819_Dnov/1-1075	--NDTLGRPQAPLPLQCGEAGSELAVQ------ETGSPG-PV-AGDHQLE--TEDPEEMS	1048
ENSSSCP00000041077_Sscr/1-1089	--NDALTRHQALLPLQCGEADAELTAQ------ETGLQG-SV-GGDHHPE--MEDPEEMS	1062
ENSTTRP00000001326_Ttru/1-1091	--NDALTRHQALLPLQCGETDSELTVQ------ETGLQG-PV-GGDHQPE--MEDPEEMS	1064
ENSBBBP00000002858_Bbbi/1-1068	--NDALTKHQALLPLQCGETDSELTSQ------ETGLQG-PV-GEDHQLE--MEDPEEMS	1041
ENSOARP00000021602_Oari/1-1032	--NDALTKHQALLPLQCGETDSELTAQ------ETGLQG-PV-GEDHQLE--MEDPEEMS	1005
ENSCHIP00000033385_Chir/1-1085	--NDALTKHQALLPLQCGETDSELTAQ------ETGLQG-PV-SEDHQLE--MEDPEEMS	1058
ENSBTAP00000059078_Btau/1-1085	--NDALTKHQALLPLQCGETDSELTSQ------ETGLQG-PV-GEDHQLE--MEDPEEMS	1058
ENSBIXP00000038534_Bthy/1-1085	--NDALTKHQALLPLQCGETDSELTSQ------ETGLQG-PV-GEDHQLE--MEDPEEMS	1058
ENSBMUP00000019115_Bmut/1-1081	--NDALTKHQALLPLQCGETDSELTAQ------ETGLQG-PV-GEDHQLE--MEDPEEMS	1054
ENSBIXP00005030570_Bihy/1-1085	--NDALTKHQALLPLQCGETDSELTAQ------ETGLQG-PV-GEDHQLE--MEDPEEMS	1058
ENSMUGP00000006365_Mung/1-1080	--NDALSRHQALLPLQCADTDSEMTIQ------ETGLQG-PM-LGDHQPE--MESPEEMS	1053
ENSRNOP00000069629_Rnor/1-1079	--NDTLGRHQALLPLQCADADSEMTIQ------ETGLQG-PM-VGDHQPE--MESSDEMS	1052
MGP_PahariEiJ_P0028852_Mpah/1-1003	--NDALNRHQALLPLQCAEADSEMTIQ------ETGLQG-PM-VGDHQPE--MESADEMS	976
MGP_SPRETEiJ_P0043347_Mspr/1-1079	--NDTLNRHQALLPLQCAEADSETTIQ------ETGLQG-PM-VGDHQPE--IESPDEMS	1052
ENSMUSP00000069080_Mmus/1-1079	--NDTLNRHQALLPLQCAEADSEMTIQ------ETGLQG-PM-VGDHQPE--IESPDEMS	1052
ENSMOCP00000010340_Moch/1-1076	--NDTLSRHQALLPLQCADTDSEMTMQ------ETGLQG-PM-VGDHQPE--MESTEEMS	1049
ENSPEMP00000029995_Pmba/1-1076	--NDTLGRHQALLPLQCADTDSEMTTQ------E---TG-PM-VGDHQPE--MESPEEMS	1049
ENSCGRP00015024036_Cgpi/1-1081	--NDTLSRHQALLPLQCADTDSEMTIQ------ETGLQG-PM-VGDHQPE--MESPEEKS	1054
ENSCGRP00001016053_Cgch/1-1081	--NDTLSRHQALLPLQCADTDSEMTIQ------ETGLQG-PM-VGDHQPE--MESPEEKS	1054
ENSCGRP00000017369_Cgcr/1-1081	--NDTLSRHQALLPLQCADTDSEMTIQ------ETGLQG-PM-VGDHQPE--MESPEEKS	1054
ENSDORP00000002959_Dord/1-1078	--HEALTRHQALLPLPCEDTDSELTIQ------EPGKGG-GFAGGDCQPE--MEDPEEMS	1051
ENSTSYP00000002643_Csyr/1-1015	--NDTLTRHQALLPLQCGEIDSELTIQ------DPGLQG-PV-GGDHQPE--MEDPEEMS	988
ENSMLUP00000008249_Mluc/1-1075	--NDTLTRHQALLPLQCGEPDSELTAQ------ETGLQG-PV-GGDRQPE--AEDPEEMS	1048
ENSECAP00000022285_Ecab/1-1084	--NDTLTRHQALLPLQRGEIDSELSAQ------ETGLQG-PA-VGDRQPE--RKNPEEMS	1057
ENSEASP00005022676_Eaas/1-1094	--NDTLTRHQALLPLQRGEIDSELSTQ------ETGLQG-PA-VGDRQPE--RKNPEEMS	1067
ENSCAFP00000017381_Cfam/1-1075	--PEPPARPQALLPPQGGDTDAELPAQ------EPGLQG-PG-GADRRPE--MRDPEELS	1048
ENSCAFP00020007154_Cldi/1-1075	--PEPPARPQALLPPQGGDTDAELPAQ------EPGLQG-PG-GADRRPE--MRDPEELS	1048
ENSVVUP00000030826_Vvul/1-1075	--PEPPARPQALLPPQGGDADAELPAQ------EPGLQG-PA-GADRRPE--MRDPEELS	1048
ENSFCAP00000008080_Fcat/1-1081	--NETLTRHQALLPLQCGETDSELSAQ------ERGLQG-PV-DGDFRPE--MEDPEEMS	1054
ENSPPRP00000023800_Ppar/1-1081	--NETLTRHQALLPLQCGETDSELSAQ------ERGLQG-PV-DGDFRPE--MEDPEEMS	1054
ENSPTIP00000015042_Ptal/1-1081	--NETLTRHQALLPLQCGETDSELSAQ------ERGLQG-PV-DGDFRPE--MEDPEEMS	1054
ENSMPUP00000004759_Mpfu/1-1082	--NETLSRHQALLPLQCGETDSELTVQ------ETGLQG-PV-GGDHRPE--TEDPEEMS	1055
ENSNVIP00000018564_Nvis/1-1092	--NETLSRHQALLPLQCGETDSELTVQ------EAGLQG-PG-GGDHRPE--TEDPEEMS	1065
ENSUMAP00000035085_Umar/1-1071	----TLTRHQALLPLQCREIDSDLTAQ------ETGLQG-PV-GGDRRPE--TEDPEEMS	1044
ENSUAMP00000035930_Uame/1-1079	--NETLTRHQALLPLQCREIDSDLTAQ------ETGLQG-PV-GGDRRPE--TEDPEEMS	1052
ENSAMEP00000015060_Amel/1-1089	--NETLTRHQALLPLQCRETDSDLTAQ------ETGLQG-PV-GGGRRPE--TEDPEEMS	1062
ENSSDAP00000007437_Sdau/1-1079	--SDALTRHQALLPLQCGETDSELTTQ------ETGLQG-PM-AGAHQPE--MEDPEEMS	1052
ENSSTOP00000003945_Itri/1-1070	--SDALTRHQALLPLQCGETDSELTTQ------ETGLQG-PM-AGAHQPE--MEDPEEMS	1043
ENSMMMP00000015298_Mmma/1-1083	--SDALTRHQALLPLQCGETDSDLTTQ------ETGLQG-PM-AGAHQPE--MEDPEEMS	1056
ENSUPAP00010025947_Upar/1-1086	--SDALTRHQALLPLQGGETDSELTTQ------ETGLQG-PM-AGAHQPE--MEDPEEMS	1059
ENSSBOP00000036416_Sbbo/1-1078	--SDTLTRHQALLPLQCGETDSDLTVQ------ETGLQG-SV-GADHRPE--VEDPEEMS	1051
ENSANAP00000028404_Anan/1-1078	--SDTLTRHQALLPLQCGETDADLTVQ------ETGLQG-PV-GGDHRPE--VEDPEELS	1051
ENSCCAP00000024327_Ccap/1-1077	--SDTLTRHQALLPLQCGETDSDLTVQ------ETGLQG-PV-GGDHQPE--VEDSEELS	1050
ENSCJAP00000062854_Cjac/1-1089	--SDTLTRHQALLPLQCGETDSDLTVQ------ETGLQG-PV-GGDHRPE--VEDSEELS	1062
ENSNLEP00000000336_Nleu/1-1088	--SDTLTRHQPLLPLQCGETDSDLAVQ------ETGLQG-PV-GGDHRPE--VEDPEELS	1061
ENSPPYP00000015087_Pabe/1-1078	--SDTLTRHQPLLPLQCGETDLDLTVQ------ETGLQG-PV-GGDQRPE--VEDPEELS	1051
ENSP00000420194_Hsap/1-1088	--SDTLTRHEPLLPLQCGETDLDLTVQ------ETGLQG-PV-GGDQRPE--VEDPEELS	1061
ENSGGOP00000020287_Ggor/1-1078	--SDTLTRHQPLLPLQCGETDLDLTVQ------ETGLQG-PV-GGDQRPE--VEDPEELS	1051
ENSPPAP00000026371_Ppan/1-1078	--SDTLTRHQPLLPLQCGETDLDLTVQ------ETGLQG-PV-GGDQRPE--VEDPEELS	1051
ENSPTRP00000043342_Ptro/1-1078	--SDTLTRHQPLLPLQCGETDLDLTVQ------ETGLQG-PV-GGDQRPE--VEDPEELS	1051
ENSCSAP00000003635_Csab/1-882	--SDTLTRHQALLPLQCGEADSDLSVQ------ETGLQG-PV-GGDHRPE--VEDPEELS	855
ENSMMUP00000061002_Mmul/1-1078	--SDTLARHQALLPLQCGEADSDLSVQ------ETGLQG-PV-GGDHRPE--VEVPEELS	1051
ENSPANP00000004316_Panu/1-1081	--SDTLARHQALLPLQCGEADSDLSVQ------ETGLQG-PA-GGDHRPE--VEVPEELS	1054

```
ENSCATP00000028615_Caty/1-1078         --SDTLARHQALLPLQCGEADSDLSVQ------ETGLQG-PV-GGDHRPE--VEVPEELS   1051
ENSMFAP00000042132_Mfas/1-1078         --SDTLARHQALLPLQCGEADSDLSVQ------ETGLQG-PV-GGDHRPE--VEVPEELS   1051
ENSMNEP00000024602_Mnem/1-1078         --SDTLARHQALLPLQCGEADSDLSVQ------ETGLQG-PV-GGDHRPE--VEVPEELS   1051
ENSMLEP00000008744_Mleu/1-1088         --SDTLARHQALLPLQCGEADSDLSVQ------ETGLQG-PV-GGDHRPE--VEVPEELS   1061
ENSTGEP00000008766_Tgel/1-1088         --SDTLARHQALLPLQCGEADSDLSVQ------ETGLQG-PV-GGDHRPE--VEVPEELS   1061
ENSCANP00000040262_Capa/1-1088         --SDTLTRHQALLPLQCGEVDSDLSVQ------ETGLQG-PV-GGDHRPE--MEDPEELS   1061
ENSPTEP00000016080_Ptep/1-1088         --SDTLTRHQALLPLQCGEVDSDLSVQ------ETGLQG-PV-GGDHRPE--VEDPEELS   1061
ENSRBIP00000006831_Rbie/1-1078         --SDTLTRHQALLPLQCGEVDSDLSVQ------ETGLQG-PV-GGDHRPE--VEDPEELS   1051
ENSRROP00000007818_Rrox/1-1088         --SDTLTRHQALLPLQCGEVDSDLSVQ------ETGLQG-PV-GGDHRPE--VEDPEELS   1061
ENSOGAP00000002850_Ogar/1-1077         --NDTLTRHQALLPLQCGDTDSELTIQ------ETGLQG-PG-GGDHQRE--REDPEEMS   1050
ENSMICP00000050654_Mmur/1-1075         --SDTLSRHQALLPLQCGETDPDPTAQ------DAGLQG-PG-GGDLPPE--MEGPEEMS   1048
ENSPCOP00000009624_Pcoq/1-1076         --NDALSRHQALLPLQCGETDSELMIQ------DTGLQG-PG-GGDHPPE--MEDPEEMS   1049
ENSPSMP00000007909_Psim/1-1084         --SDPLGRHQALLPLQCGETDSELTVP------DTGLQG-PG-GGDHPPE--MEDPEEMS   1057


ENSMEUP00000013310_Neug/1-956          ---------------------------------    956
ENSTBEP00000002487_Tbel/1-914          PALVVSNSRSFVISSGSSTGTENILHS------    914
ENSPCAP00000000755_Pcap/1-1085         PALVVSKSQSFVISGGGGTGQRNVNS-------    1085
ENSCHOP00000011907_Chof/1-954          ---------------------------------    954
ENSVPAP00000001982_Vpac/1-1087         PALVVSSSRSFVISGGGSTVTENMLHS------    1087
ENSEEUP00000009670_Eeur/1-946          PALVVSNAHSFVISRGGSTITENILHL------    946
ENSOANP00000015961_Oana/1-1083         PALLVPNARSFVISGXXXXXXXXXXXXXX----    1083
ENSOPRP00000000261_Opri/1-1082         PGLVVSNSRSFVISGGGSAVAENRLHS------    1082
ENSJJAP00000004721_Jjac/1-1084         PALVMSKSGSFVISGGGSAVTEDVLRSQSQKSS    1084
ENSPCIP00000034747_Pcin/1-1018         PGLVVSNSRGFIISGGGSAITEKKIHS------    1018
ENSMODP00000022810_Mdom/1-1069         PALVVSNSRGFIISGGGSAITEKKLHS------    1069
ENSVURP00010007344_Vurs/1-1070         PALVVSNSRGFIISGGGSAVTEKKIHS------    1070
ENSSHAP00000014890_Shar/1-1072         PALVVSNSRSFIISGGGSAVTEKKIHS------    1072
ENSMAUP00000005394_Maur/1-954          PCMVLLE-------GHGAFSK------------    954
ENSOCUP00000025672_Ocun/1-1063         P--------------------------------    1063
ENSPVAP00000011981_Pvam/1-1085         PALVVSNSRSFVISGGGSTVTENILHS------    1085
ENSETEP00000011160_Etel/1-1024         PTLVVSTSRSLVISGGGSAARENMPNS------    1024
ENSFDAP00000010532_Fdam/1-1108         PTLVMAKSRSFVISGGASTAKESSPHSQ-----    1108
ENSODEP00000013257_Odeg/1-1070         QAFVVSKSQGFVIKNIWHS--------------    1070
ENSHGLP00000002019_Hgfe/1-1108         PALVMSKSRSFVISGGGSTVTENILHS------    1108
ENSCLAP00000021334_Clan/1-1114         PAFVVSKSQGFVISGGGSAVTENILHT------    1114
ENSCAPP00000014352_Cape/1-1068         PAFVMSKSQGFVFSGGGSTVTENILHS------    1068
ENSCPOP00000011705_Cpor/1-1071         PAFVMSKSQGFVFSGGGSTVTENILHS------    1071
ENSSARP00000006151_Sara/1-1085         PALVVSSSRSFVISGAGSTVTENIMHS------    1085
ENSLAFP00000018459_Lafr/1-1090         PALVVSNSRSFVISSGGSTVTENIPNS------    1090
ENSDNOP00000011819_Dnov/1-1075         PALMVSNSRGFVISGGGSTVTENMLRS------    1075
ENSSSCP00000041077_Sscr/1-1089         PALVMSNSRSFVISGGGSTVTENMLHS------    1089
ENSTTRP00000001326_Ttru/1-1091         PALVVSNSRSFVISGGGSTVTENMLRS------    1091
ENSBBBP00000002858_Bbbi/1-1068         PALVVSNSRSFVISGGGSTVTENMLRS------    1068
ENSOARP00000021602_Oari/1-1032         PALVVSNSRSFVISGGGSTVTENMLRS------    1032
ENSCHIP00000033385_Chir/1-1085         PALVVSNSRSFVISGGGSTVTENMLRS------    1085
ENSBTAP00000059078_Btau/1-1085         PALVVSNSRSFVISGGGSTVTENMLRS------    1085
ENSBIXP00000038534_Bthy/1-1085         PALVVSNSRSFVISGGGSTVTENMLRS------    1085
ENSBMUP00000019115_Bmut/1-1081         PALVVSNSRSFVISGGGSTVTENMLRS------    1081
ENSBIXP00005030570_Bihy/1-1085         PALVVSNSRSFVISGGGSTVTENMLRS------    1085
ENSMUGP00000006365_Mung/1-1080         PALVVSTSRSFVISGGGSSVTENILHS------    1080
ENSRNOP00000069629_Rnor/1-1079         PALVMSTSRSFVISGGGSSVTENVLHS------    1079
MGP_PahariEiJ_P0028852_Mpah/1-1003     PALVMSTSRSFVISGGGSSMSENILHS------    1003
MGP_SPRETEiJ_P0043347_Mspr/1-1079      PALVMSTSRSFVISGGGSSVTENILHS------    1079
ENSMUSP00000069080_Mmus/1-1079         PALVMSTSRSFVISGGGSSVTENILHS------    1079
ENSMOCP00000010340_Moch/1-1076         PALVMSTSRSFVISGGGSSVTENILHS------    1076
ENSPEMP00000029995_Pmba/1-1076         PALVVSTSRSFVISGGGSSVTENILHS------    1076
ENSCGRP00015024036_Cgpi/1-1081         PALVVSTSRSFVISGGGSSVTENILHS------    1081
ENSCGRP00001016053_Cgch/1-1081         PALVVSTSRSFVISGGGSSVTENILHS------    1081
ENSCGRP00000017369_Cgcr/1-1081         PALVVSTSRSFVISGGGSSVTENILHS------    1081
ENSDORP00000002959_Dord/1-1078         PALVMSKSRSFVISGGGSTVTENILHS------    1078
ENSTSYP00000002643_Csyr/1-1015         PARVVSSSRSFVISGGGSTVTENILHS------    1015
ENSMLUP00000008249_Mluc/1-1075         PALVVSNSRSFVISGGGSTVTENILHS------    1075
ENSECAP00000022285_Ecab/1-1084         PTLVVSNSRSFVISGGGSTVTENILHS------    1084
ENSEASP00005022676_Eaas/1-1094         PTLVVSNSRSFVISGGGSTVTENILHS------    1094
ENSCAFP00000017381_Cfam/1-1075         PALVVSSSQSFVISGGGSTVTENILHS------    1075
ENSCAFP00020007154_Cldi/1-1075         PALVVSSSQSFVISGGGSTVTENILHS------    1075
ENSVVUP00000030826_Vvul/1-1075         PALVVSSSQSFVISGGGSTVTENILHS------    1075
ENSFCAP00000008080_Fcat/1-1081         PALVVSSSQSFVISGGGSTVTENILHS------    1081
ENSPPRP00000023800_Ppar/1-1081         PALVVSSSQSFVISGGGSTVTENILHS------    1081
ENSPTIP00000015042_Ptal/1-1081         PALVVSSSQSFVISGGGSTVTENILHS------    1081
ENSMPUP00000004759_Mpfu/1-1082         PALVVSSSQSFVISSGGSTVTENILHS------    1082
ENSNVIP00000018564_Nvis/1-1092         PALVVSSSQSFVISSGGSTVTENILHS------    1092
ENSUMAP00000035085_Umar/1-1071         PALVVSSSQSFVISSGGSTVTENILHS------    1071
ENSUAMP00000035930_Uame/1-1079         PALVVSSSQSFVISSGGSTVTENILHS------    1079
ENSAMEP00000015060_Amel/1-1089         PALVVSSSQSFVISSGGSTVTENILHS------    1089
ENSSDAP00000007437_Sdau/1-1079         PALVVSSSRSFVISGGGSTVTENILHS------    1079
ENSSTOP00000003945_Itri/1-1070         PALVVSSSRSFVISGGGSTVTENILHS------    1070
ENSMMMP00000015298_Mmma/1-1083         PALVVSSSRSFVISGGGSTVTENILHS------    1083
ENSUPAP00010025947_Upar/1-1086         PALVVSSSRSFVISGGGSTVTENILHS------    1086
ENSSBOP00000036416_Sbbo/1-1078         PALVVSSSQSFVISGGGSTVTENVLHS------    1078
ENSANAP00000028404_Anan/1-1078         PALVVSSSQSFVISGGGSTVTENVLHS------    1078
ENSCCAP00000024327_Ccap/1-1077         PALVVSSSQSFVISGGGSTVTENVLHS------    1077
ENSCJAP00000062854_Cjac/1-1089         PALVVSSSQSFVISGGGSTVTENVLHS------    1089
ENSNLEP00000000336_Nleu/1-1088         PALVVSSSQSFVISGGGSTVTENVLNS------    1088
ENSPPYP00000015087_Pabe/1-1078         PALVVSSSQSFVISGGGSTVTENVLNS------    1078
ENSP00000420194_Hsap/1-1088            PALVVSSSQSFVISGGGSTVTENVVNS------    1088
ENSGGOP00000020287_Ggor/1-1078         PALVVSSSQSFVISGGGSTVTENVLNS------    1078
```

```
ENSPPAP00000026371_Ppan/1-1078         PALVVSSSQSFVISGGGSTVTENVLNS------    1078
ENSPTRP00000043342_Ptro/1-1078         PALVVSSSQSFVISGGGSTVTENVLNS------    1078
ENSCSAP00000003635_Csab/1-882          PALVVSSSQSFVISGGGSTVTENVLHS------    882
ENSMMUP00000061002_Mmul/1-1078         PALVVSSSQSFVISGGGSTVTENVLHS------    1078
ENSPANP00000004316_Panu/1-1081         PALVVSSSQSFVISGGGSTVTENVLHS------    1081
ENSCATP00000028615_Caty/1-1078         PALVVSSSQSFVISGGGSTVTENVLHS------    1078
ENSMFAP00000042132_Mfas/1-1078         PALVVSSSQSFVISGGGSTVTENVLHS------    1078
ENSMNEP00000024602_Mnem/1-1078         PALVVSSSQSFVISGGGSTVTENVLHS------    1078
ENSMLEP00000008744_Mleu/1-1088         PALVVSSSQSFVISGGGSTVTENVLHS------    1088
ENSTGEP00000008766_Tgel/1-1088         PALVVSSSQSFVISGGGSTVTENVLHS------    1088
ENSCANP00000040262_Capa/1-1088         PALVVSSSQSFVISGGGSTVTENVLHS------    1088
ENSPTEP00000016080_Ptep/1-1088         PGLVVSSSQSFVISGGGSTVTENVLHS------    1088
ENSRBIP00000006831_Rbie/1-1078         PALVVSSSQSFVISGGGSTVTENVLHS------    1078
ENSRROP00000007818_Rrox/1-1088         PALVVSSSQSFVISGGGSTVTENVLHS------    1088
ENSOGAP00000002850_Ogar/1-1077         PALVVSNSRSFVISGGGSTVTENVLHL------    1077
ENSMICP00000050654_Mmur/1-1075         PALVVSSSRSFVISGGGSTVTENVLHL------    1075
ENSPCOP00000009624_Pcoq/1-1076         PALVVSNSRSFVISGGGSTVTENVLHL------    1076
ENSPSMP00000007909_Psim/1-1084         PALVVPNSRSFVISGGGSTVTENVLHL------    1084
```

Supplementary Figure 3: *Mammalian CaSR multiple sequence alignment including the residue 91 (red arrows). The alignment was generated using the Clustal Omega server [1].*

In vitro screening of 159 predicted kokumi-active compounds on cCaSR

Compound ID	CAS number	EC_{50} Ago (M)	EC_{50} PAM (M)
(1R)-(+)-(1-Amino-2-methylpropyl)phosphonic acid	66254-56-6	-	-
(S)-α-Methylaspartic acid	3227-17-6	-	-
1,4,8,11-tetraazacyclotetradecane	295-37-4	0.000773242	-
1-isopropyl-2,3,4,9-tetrahydro-1H-pyrido[3,4-b]indole-3-carboxylic acid	436811-11-9	-	-
1s,3s-1-aminocyclobutane-1,3-dicarboxylic acid	73550-55-7	> 0.01	-
2,6-Diaminopimelic acid	583-93-7	-	-
2-Amino-4-phosphonobutyric acid	20263-07-4	> 0.01	-
2-aminododecanedioic acid	3721-90-2	-	-
2-Aminopimelic acid	627-76-9	-	-
2S,4S-g-Hydroxy-L-glutamic acid	3913-68-6	> 0.01	-
3,5-Dimethyl-4-methoxybenzoylacetonitrile	884504-21-6	-	-
3-Glutathionyl-S-methylindole	101038-93-1	0.003746383	-
3-O-Methyl-D-glucopyranose	13224-94-7	-	-
4-Fluoro-DL-glutamic acid	2708-77-2	> 0.01	-
9H-pyrido[3,4-b]indole-3-carboxylic acid	42438-90-4	-	-
AC-265347	1253901-26-6	-	5.11238E-08
Antibiotic G418	49863-47-0	-	-
$BaCl_2$	10361-37-2	0.001172392	-
$CaCl_2$	10035-04-8	0.001622123	-
Cadaverine	462-94-2	-	-
Calcium Ionophore	58801-34-6	-	-
Calhex 231	2387505-78-2	-	-
Calindol	729610-18-8	-	2.96569E-07
Cerium(III) carbonate	54451-25-1	-	-
ChemBridge CORE Library - 7126534	N/A	-	3.99941E-06
ChemBridge CORE Library - 7156222	N/A	-	3.68478E-06
Cinacalcet	226256-56-0	-	7.46105E-07
Cystine (disufide)	56-89-3	-	-
D-(+)-Glucose	2280-44-6	-	-
D-Arginine	157-06-2	-	-
D-Aspartic Acid	1783-96-6	-	-
D-Glutamic acid	6893-26-1	-	-
Dichloromethylenediphosphonic acid	22560-50-5	-	-
DL-Aspartic acid alpha-methyl ester	65414-77-9	> 0.01	-
D-Lysine	923-27-3	-	-
Enamine Screening - Z1362199910	N/A	-	5.88796E-05
Enamine Screening - Z1436888168	N/A	-	4.57134E-05
Enamine Screening - Z1437387381	N/A	-	9.00814E-06
Enamine Screening - Z1532507755	N/A	-	8.68191E-06
Enamine Screening - Z1563602968	N/A	-	-
Enamine Screening - Z1589915397	N/A	-	0.000122222
Enamine Screening - Z1592615858	N/A	-	1.92068E-06

Enamine Screening - Z1627256025	N/A	-	0.000796597
Enamine Screening - Z1857407854	N/A	-	5.5023E-06
Enamine Screening - Z317045236	N/A	-	2.41537E-06
Enamine Screening - Z336933272	N/A	-	1.21254E-05
Gd^{3+}	16056-77-2	0.000294595	-
Gentamicin	1403-66-3	0.000989847	-
Glutathione (γ-Glu-Cys-Gly)	70-18-8	0.006396298	-
Glycine	56-40-6	-	-
Hexamethylenetetramine	100-97-0	-	-
Hydroxyproline	51-34-4	-	-
Hygromycin B	31282-04-9	-	-
InterBioScreen Screening - STOCK1N-20329	N/A	-	9.87677E-07
InterBioScreen Screening - STOCK1N-29175	N/A	-	7.13774E-07
InterBioScreen Screening - STOCK1N-29290	N/A	-	4.49052E-06
InterBioScreen Screening - STOCK1N-32683	N/A	-	6.73283E-07
InterBioScreen Screening - STOCK1N-32983	N/A	-	7.51716E-07
InterBioScreen Screening - STOCK2S-09124	N/A	-	1.40781E-06
InterBioScreen Screening - STOCK2S-10026	N/A	-	4.57282E-07
InterBioScreen Screening - STOCK3S-12225	N/A	-	2.25131E-06
InterBioScreen Screening - STOCK3S-57962	N/A	-	1.44874E-06
InterBioScreen Screening - STOCK3S-69159	N/A	-	0.000135294
InterBioScreen Screening - STOCK4S-85937	N/A	-	0.000114962
InterBioScreen Screening - STOCK5S-00071	N/A	-	0.000171864
Kanamycin solution	25389-94-0	-	-
L-(+)-2-Amino-3-phosphonopropionic acid	23052-80-4	> 0.01	-
L-2-Amino-4-phosphonobutyric acid	20263-07-4	-	-
L-2-Aminoadipic acid	1118-90-7	> 0.01	-
L-Alanine	56-41-7	-	-
L-Arginine	74-79-3	-	-
L-Arginine methyl ester	2577-94-8	-	-
L-Asparagine	70-47-3	-	-
L-Aspartic acid	56-84-8	0.004114576	-
L-Aspartic acid β-methyl ester hydrochloride	16856-13-6	> 0.01	-
L-Cysteic acid	498-40-8	0.002339771	-
L-Cysteine	52-90-4	-	-
L-Glutamic acid	56-86-0	0.00915731	-
L-Glutamic Acid 5-Methyl Ester	1499-55-4	-	-
L-Glutamine	56-85-9	-	-
L-Histidine	71-00-1	-	-
L-Homocysteic acid	14857-77-3	> 0.01	-
L-Homoserine	672-15-1	-	-
L-Isoglutamine	636-65-7	> 0.01	-
L-Isoleucine	73-32-5	-	-
L-Leucine	61-90-5	-	-
L-Methinionine	63-68-3	-	-
L-Ornithine	70-26-8	-	-

L-Proline	147-85-3	-	-
L-Selenocystine monohydrate	29621-88-3	-	-
L-Serine	56-45-1	-	-
L-Taurine	107-35-7	-	-
L-Threonine	72-19-5	-	-
L-Tyrosine	60-18-4	-	-
L-Valine	72-18-4	-	-
Methylenediphosphonic acid	1984-15-2	0.001292506	-
Methylphosphonic acid	993-13-5	>0.01	-
Mg^{2+}	22537-22-0	0.006293176	-
N,N'-Bis(2-aminoethyl)-1,3-propanediamine	4741-99-5	-	-
Neomycin	119-04-0	0.001868047	-
NG,NG-Dimethylarginine dihydrochloride	220805-22-1	-	-
NG-methyl L Arginine	53308-83-1	-	-
NG-Nitro-L-Arginine Methyl Ester	2149-70-4	-	-
NPS 2143	284035-33-2	-	-
Nω-Nitro-L-arginine methyl ester hydrochloride	51298-62-5	-	-
O-Phospho-L-tyrosine	21820-51-9	> 0.01	-
Paromomycin sulfate salt	1263-89-4	0.001084496	-
Poly-L-arginine	26982-20-7	1.0135E-06	-
Poly-L-histidine	26062-48-6	-	-
Poly-L-lysine	25988-63-0	> 0.01	-
Poly-L-ornithine	26982-21-8	0.000239862	-
Praseodymium(III) trifluoromethanesulfonate	52093-27-3	0.000397784	-
Ribostamycin sulfate salt	53797-35-6	> 0.01	-
S-(2-Hydroxyethyl)glutathione	28747-20-8	0.002097055	-
S-1,1-dimethyl-2,3,4,9-tetrahydro-1H-pyrido[3,4-b]indole-3-carboxylic acid	N/A	-	-
S-2,3-diaminopropanoic acid	4033-39-0	-	-
Se-(Methyl)selenocysteine	863394-07-4	0.004468107	-
Sisomicin sulfate salt	53179-09-2	0.000299662	-
S-Lactoylglutathione	25138-66-3	0.006084892	-
S-Methylglutathione	2922-56-7	0.004418495	-
Sodium pyrophosphate tetrabasic decahydrate	13472-36-1	-	-
Spermidine	124-20-9	0.002495307	-
Spermine	71-44-3	0.012240719	-
S-piperazine-2-carboxylic acid	158663-69-5	-	-
Sr^{2+}	22537-39-9	>0.01	-
Sucralose	56038-13-2	-	-
Sucrose	57-50-1	-	-
Terbium(III) acetate hydrate	100587-92-6	0.000174627	-
Tobramycin	32986-56-4	-	0.024201762
Triethylenetetramine	112-24-3	-	
Vitas M Labs Express Pick - STK530456	N/A	-	2.61244E-05
β-Asp-Ala	13110-25-3	> 0.01	-
β-Asp-Gly	3790-52-1	> 0.01	-

**β-Asp-Leu**	**14650-26-1**	**> 0.01**	**-**
**β-Asp-Lys**	**56523-60-5**	**-**	**-**
**β-Asp-Phe**	**13433-10-8**	**> 0.01**	**-**
**γ-Carboxy-DL-glutamic acid**	**56271-99-9**	**> 0.01**	**-**
**γ-D-Glu-Trp**	**66471-20-3**	**0.004204093**	**-**
**γ-Glu-Abu**	**16869-42-4**	**> 0.01**	**-**
**γ-Glu-Abu-Gly**	**495-27-2**	**0.002658678**	**-**
**γ-Glu-Ala**	**5875-41-2**	**> 0.01**	**-**
**γ-Glu-Cys**	**636-58-8**	**> 0.01**	**-**
**γ-Glu-Cys-Gly-Oet**	**92614-59-0**	**-**	**-**
**γ-Glu-Gln**	**10148-81-9**	**> 0.01**	**-**
**γ-Glu-Glu**	**1116-22-9**	**0.002064774**	**-**
**γ-Glu-Glu-Gln**	**211430-99-8**	**> 0.01**	**-**
**γ-Glu-Glu-Glu**	**7446-63-1**	**0.002345792**	**-**
**γ-Glu-Gly**	**1948-29-4**	**> 0.01**	**-**
**γ-Glu-Gly-Gly**	**13640-39-6**	**-**	**-**
**γ-Glu-His**	**37460-15-4**	**-**	**-**
**γ-Glu-Leu**	**2566-39-4**	**> 0.01**	**-**
**γ-Glu-Lys**	**56523-60-5**	**-**	**-**
**γ-Glu-Met**	**17663-87-5**	**> 0.01**	**-**
**γ-Glu-Phe**	**13433-10-8**	**0.002316942**	**-**
**γ-Glu-Trp**	**66471-20-3**	**> 0.01**	**-**
**γ-Glu-Tyr**	**7432-23-7**	**0.003877693**	**-**
**γ-Glu-Val**	**2746-34-1**	**0.004518862**	**-**
**γ-Glu-Val-Gly**	**38837-70-6**	**0.004222828**	**-**
**γ-Glu-ε-Lys**	**17105-15-6**	**-**	**-**

Supplementary Table I: *List of all 159 tested compounds on cCaSR using in vitro cellular assays. The table contains the EC_{50} values for both agonists (EC_{50} Ago(M)) and positive allosteric modulators (EC_{50} PAM (M)). All compounds listed here were screened, using the HEK293/ T-REx/ natClytin cell line inducibly expressing cCaSR in an agonist assay**.** All measurements were repeated over at least three wells on the same assay plate. If the maximal response to a compound was not reached, the EC_{50} value could not be determined and is expressed as an estimation at > 0.01 M. -: compounds that showed no activity on cCaSR in the specific testing parameters used.*

References:

1 Sievers, F. *et al.* Fast, scalable generation of high-quality protein multiple sequence alignments using Clustal Omega. *Mol Syst Biol* **7**, 539, doi:10.1038/msb.2011.75 (2011).
